# Computational Derivation of Core, Dynamic Human Blunt Trauma Inflammatory Endotypes

**DOI:** 10.3389/fimmu.2020.589304

**Published:** 2021-01-18

**Authors:** Lukas Schimunek, Haley Lindberg, Maria Cohen, Rami A. Namas, Qi Mi, Jinling Yin, Derek Barclay, Fayten El-Dehaibi, Andrew Abboud, Ruben Zamora, Timothy Robert Billiar, Yoram Vodovotz

**Affiliations:** ^1^ Department of Surgery, University of Pittsburgh, Pittsburgh, PA, United States; ^2^ Department of Sports Medicine and Nutrition, University of Pittsburgh, Pittsburgh, PA, United States; ^3^ Center for Inflammation and Regenerative Modeling, McGowan Institute for Regeneration Medicine, University of Pittsburgh, Pittsburgh, PA, United State

**Keywords:** systems biology, inflammation, biomarker, critical illness, network analysis

## Abstract

Systemic inflammation ensues following traumatic injury, driving immune dysregulation and multiple organ dysfunction (MOD). While a balanced immune/inflammatory response is ideal for promoting tissue regeneration, most trauma patients exhibit variable and either overly exuberant or overly damped responses that likely drive adverse clinical outcomes. We hypothesized that these inflammatory phenotypes occur in the context of severe injury, and therefore sought to define clinically distinct endotypes of trauma patients based on their systemic inflammatory responses. Using Patient-Specific Principal Component Analysis followed by unsupervised hierarchical clustering of circulating inflammatory mediators obtained in the first 24 h after injury, we segregated a cohort of 227 blunt trauma survivors into three core endotypes exhibiting significant differences in requirement for mechanical ventilation, duration of ventilation, and MOD over 7 days. Nine non-survivors co-segregated with survivors. Dynamic network inference, Fisher Score analysis, and correlations of IL-17A with GM-CSF, IL-10, and IL-22 in the three survivor sub-groups suggested a role for type 3 immunity, in part regulated by Th17 and γδ 17 cells, and related tissue-protective cytokines as a key feature of systemic inflammation following injury. These endotypes may represent archetypal adaptive, over-exuberant, and overly damped inflammatory responses.

## Introduction

Trauma, with more than five million deaths annually, is one of the leading causes of death worldwide (1). The body reacts to trauma with an initial inflammatory response, which can drive multiple organ dysfunction (MOD). The pathophysiology leading to MOD involves multiple cell populations, and immune dysregulation and sepsis are major consequences (2, 3). Systemic inflammation ensues in many disease states as a consequence of innate immune activation (4), and this activation of innate immune responses impacts other facets of immunity in the context of severe traumatic injury (5). Traumatic injury triggers the activation of the complement system and the release of danger-associated molecular patterns (DAMPs), which activate the innate immune system (6). Key features of trauma-induced inflammation are a suppressed adaptive immune system secondary to reduced T helper (Th)1 activation and enhanced Th2 activation (7–9). In contrast, T helper 17 (Th17) (10, 11) function is enhanced following trauma. Of these, Th17 cells produce IL-17A, which activates neutrophils, have been associated recently with adverse trauma outcomes including mortality (11, 12), and presumably drive a feed-forward loop of inflammation → tissue damage/dysfunction → inflammation (5). This process is kept in check by Th2 cells, which suppress inflammation *via* the release of cytokines such as IL-10, as well as activating other arms of the adaptive immune system such as B cells (13). However, this same negative feedback can lead to hypo-inflammation, which, combined with immunosuppression driven by overly exuberant Th1 responses, can predispose to nosocomial infection, sepsis, and exacerbation of MODS (14). Many other cell types and cytokines are involved in the response to trauma (5), including natural killer (NK) cells (15), mast cells (16), and innate lymphoid cells (ILC) (17, 18) ILC and NK cells are activated early and also participate in the regulatory cytokine landscape that shapes the pro- and counter-inflammatory response in trauma (5, 13, 19).

While a balanced inflammatory and immune response is ideal for tissue regeneration and wound healing, most trauma patients exhibit either overly exuberant or overly dampened responses (5, 20, 21). These complex, dynamic, processes are likely a key aspect of the large variability observed in the systemic inflammatory responses within trauma patient populations (22–24), and thus defining inflammatory trauma endotypes has lagged in comparison to related fields such as sepsis (25). We and others have derived insights into the response to traumatic injury using dynamic network analyses in propensity-matched outcome sub-cohorts (26) and mathematically modeled “virtual trauma patients” (27); however, there is to date no unified definition of core inflammatory trauma endotypes. In a previous pilot study, we provided proof of concept for the derivation of trauma patient endotypes in the form of “inflammation barcodes,” by segregating the 5-day clinical outcomes of two small cohorts of trauma survivors based on their dynamic core inflammatory responses within the first 24 h post-injury. This was accomplished using patient-specific Principal Component Analysis (PCA) combined with unsupervised hierarchical clustering (28).

We therefore hypothesized that adaptive/balanced, over-exuberant, and overly damped inflammatory responses play a role in severe traumatic injury, and that the use of computational strategies such as patient-specific PCA could help define clinically distinct trauma endotypes. We show, in a large cohort of blunt trauma survivors and non-survivors, that three core inflammatory endotypes exist after trauma, which generally match the qualitative phenotypes of adaptive, overly exuberant, or overly damped post-injury systemic inflammation (5). In contrast to established dogma but supported by prior studies on trauma non-survivors (12, 29), type 3 immunity, in part regulated by Th17 cells, is a major hallmark of these endotypes, as are epithelium-derived protective cytokines.

## Methods

### Selection of Patients

The study cohort consisted of 236 patients who were studied following their admission to the Presbyterian University hospital emergency department (a Level 1 trauma center) in accordance with relevant guidelines and regulations and following approval by the Institutional Review Board at the University of Pittsburgh Medical Center. Informed consent was obtained from all participants in the study (cognitive impairment was not assessed). Of these, 227 were blunt trauma survivors: 148 were male and 79 were female. The mean age was 50.4 ± 1.3 years (min: 18 years, max: 90 years) and the mean ISS in this patient cohort was 17.7 ± 0.6 (min: 1, max: 50). A further 9 blunt trauma non-survivors were also included in this cohort. Of these 9 non-survivors, 7 were male and 2 were female. The mean age was 62.1 ± 7.2 years (min: 19 years, max: 86 years) and the mean ISS in this patient cohort was 19.9 ± 2.1 (min: 9, max: 33).

### Serial Analysis of Inflammatory Mediators

Inflammatory mediators were assayed as described previously (10). In brief, whole blood samples were withdrawn in heparinized tubes 3 times in the first 24 h after admission, and then daily for 7 days. The samples were kept on ice and centrifuged to obtain plasma, and then stored at −80°C until assayed for inflammatory mediators. The Luminex™ 100 IS analyzer (Luminex, Austin, TX) and Human Cytokine/Chemokine MILLIPLEX™ Panel kit (Millipore Corporation, Billerica, MA) were used to measure plasma levels of Eotaxin (CCL11), interleukin (IL)-1β, IL-1 receptor antagonist (IL-1RA), IL-2, soluble IL-2 receptor-α (sIL-2Rα), IL-4, IL-5, IL-6, IL-7, IL-8 (CCL8), IL-10, IL-13, IL-15, IL-17A, interferon (IFN)-α, IFN-γ, IFN-γ inducible protein (IP)-10 (CXCL10), monokine induced by gamma interferon (MIG; CXCL9), macrophage inflammatory protein (MIP)-1α (CCL3), MIP-1β (CCL4), monocyte chemotactic protein (MCP)-1 (CCL2), granulocyte-macrophage colony stimulating factor (GM-CSF) and tumor necrosis factor alpha (TNF-α). The human Th17 MILLIPLEX™ Panel kit (Millipore Corporation, Billerica, MA) was used to measure IL-9, IL-21, IL-22, IL-23, IL-17E/25, and IL-33. levels were measured by a Griess Reagent colorimetric assay (Cayman Chemical, Ann Arbor, MI). Soluble IL-1 receptor-like 1 (sST2) was measured by a sandwich ELISA assay (R&D Systems, Minneapolis, MN). All cytokine/chemokine mediator concentrations are given in pg/ml; concentrations are in µM. Experimental data are shown as mean ± SEM.

### Patient-Specific Principal Component Analysis

**Figure 1:**
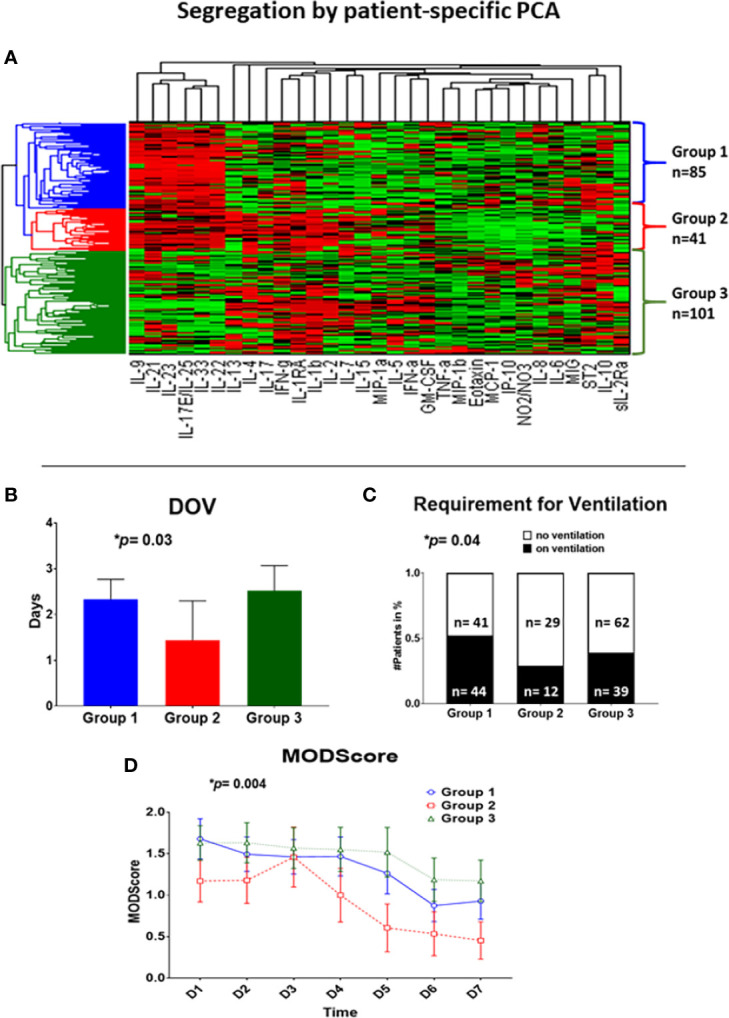
Patient-specific Principal Component Analysis followed by unsupervised hierarchical clustering of circulating inflammatory mediator data yields three distinct trauma survivor sub-groups. Patient-specific Principal Component Analysis was carried out for the Group of 227 trauma survivors using data on circulating inflammatory mediators obtained at three time points within the first 24 h of hospital admission. These data allowed patients to be clustered hierarchically using unsupervised methods as described in the *Materials and Methods* **(A)**, resulting in three patient sub-groups: Group 1 (blue; n= 85 patients), Group 2 (red; n= 41 patients), and Group 3 (green; n = 101 patients). **(B–D)** Significant clinical outcome differences among Groups 1–3. **(B)**: Group 2 (n = 41, 1.4 ± 0.9 days) showed significantly fewer days on ventilation over a time course of 8 days as compared to Group 1 (n = 85; 2.3 ± 0.4 days) and Group 3 (n = 101; 2.5 ± 0.6 days); *p =* 0.02. **(C)**: The requirement for mechanical ventilation was significantly different across Group 1 (n= 85; 44 on vs. 41 off ventilation), Group 2 (n = 41; 12 on vs. 29 off ventilation), and Group 3 (n = 101, 39 on vs. 62 off ventilation) over a time course of 8 days; *p =* 0.0127. **(D)**: Group 2 (n = 41) showed significantly lowered Marshall MODScores over a time course of 8 days as compared to Group 1 (n = 85), and Group 3 (n = 101); *p =* 0.0126.

The inflammatory mediators of the first 24 h (3 time points each patient) were analyzed by patient-specific Principal Component Analysis (PCA), followed by hierarchical clustering, which resulted in three subgroups (**Figure 1**). Group 1 (n= 85) exhibited a mean age of 45.3 ± 2.0 years (min: 18 years, max: 90 years), a mean ISS of 18.8 ± 1.0 (min: 1, max: 50) and a gender distribution of 51 males vs. 34 females. Group 2 (n = 41) showed a mean age of 54.6 ± 3.1 years (min: 18 years, max: 86 years), a mean ISS of 16.6 ± 1.3 (min: 2, max: 38) and a gender distribution of 30 males vs. 11 females. Group 3 (n = 101) had a mean age of 50.5 ± 1.9 years (min: 18 years, max: 89 years), a mean ISS of 17.1 ± 0.9 (min: 1, max: 50) and a gender ratio of 67 males vs. 34 females (**Table 1**).

**Table 1:** Demographics of Group 1 (n = 85), Group 2 (n = 41), and Group 3 (n = 101).

	Group 1 (n = 85)	Group 2 (n = 41)	Group 3 (n = 101)
**Age (years)**	48.3 ± 2.0	54.6 ± 3.1	50.5 ± 1.9
**ISS**	18.8 ± 1.0	16.6 ± 1.3	17.1 ± 0.9
**Gender**	Female: 34 Male: 51	Female: 11 Male: 30	Female: 34 Male: 64

### Statistical and Computational Analyses

To define if patient sub-groups differed with regard to demographics, clinical outcomes, or dynamic inflammatory responses, our analytic strategy was to apply a stepwise series of statistical and data-driven modeling techniques aimed at discovering significant differences, principal drivers, interconnected networks, and potential key regulatory nodes. We detail these analyses below:

1) D’Agostino & Pearson normality test was used to identify if the patient demographics and outcomes were distributed normally, using GraphPad Prism 7 (GraphPad Software, Inc., San Diego, CA). A *p*-value of less than 0.05 was considered significant.
2) One-way ANOVA followed by Tukey’s multiple comparison test was used to compare differences among groups of patients with regard to normally distributed demographics and outcomes, using GraphPad Prism 7. A *p*-value of less than 0.05 was considered significant.
3) Kruskal-Wallis test followed by Dunn’s multiple comparison test was used to compare differences among groups of patients with regard to non-normally distributed patient demographics and outcomes, using Graph Pad Prism 7. A *p*-value of less than 0.05 was considered significant.
4) Chi-Square was used to compare patient demographics and outcomes organized in contingency tables, using GraphPad Prism 7. A *p*-value of less than 0.05 was considered significant.
5) Two-Way ANOVA followed by Tukey’s multiple comparison test was used to determine time-dependent changes of circulating inflammatory mediators as a function of patient sub-group, using GraphPad Prism 7. A *p*-value of less than 0.05 was considered significant.
6) Principal Component Analysis (PCA) (30) was carried out to identify those inflammatory mediators that were the most characteristic of the overall dynamic, multivariate response of a given patient sub-group using MATLAB^®^ software (The MathWorks, Inc., Natick, MA). To perform this analysis, the data was first normalized for each inflammatory mediator (i.e., a given value divided by the maximum value for a given inflammatory mediator), so that all mediator levels were converted into the same scale (from 0 to 1). In this way, any artificial effects on variance due to the different ranges of concentration observed for different cytokines were eliminated. Only those components sufficient to capture at least 70% of the variance in the data were considered. From these leading principal components, the coefficient (weight) associated with each inflammatory mediator was multiplied by the eigenvalue associated with that principal component. This product represented the contribution of a given mediator to the variance accounted for in that principal component. The overall score given to each mediator is the sum of its scores in each component, depicted as a stacked bar graph. This gives a measure of a given inflammatory mediator’s contribution to the overall variance of the system. The mediators with the largest scores are the ones which contributed most to the variance of the process being studied (30).
7) Patient-specific PCA followed by hierarchical clustering (28) was used to differentiate the patients by their early inflammatory response. The goal of this analysis was to identify the subsets of mediators (in the form of orthogonal normalized linear combinations of the original mediator variables, called principal components) that are most strongly correlated with the inflammatory response in individual trauma patients, and that thereby might be considered principal characteristics of each response. We adapted an approach used previously (28, 31) to define patient-specific “inflammatory barcodes” using time course data and subsequent PCA. Hierarchical clustering is a simple and unbiased method for segregating series of numerical values by their similarity to each other (32). This analysis was performed using the Bioinformatics Toolbox in Matlab^®^ 8.1.0 for all inflammatory mediator data, following the patient-specific PCA described above. For better visualization, the colors of the resulting heat map were based on the standardized-transformed value (standardized along the rows of data, so that the mean is 0 and the standard deviation is 1 in the specified dimension), with red indicating higher values and green indicating lower values.
8) Inflammatory mediators that segregate each PCA/clustering-defined patient sub-group were defined using feature selection, a method used commonly in machine learning which can help reduce the dimensionality of the data, remove the irrelevant and redundant features, and directly select a subset of the relevant features in order to construct predictive models (33, 34). To carry out feature selection, we utilized the Fisher Score, a supervised feature selection method (35). The Fisher Score value indicates the degree that a given feature has similar values in the same group and dissimilar values in other groups. Since the groups were defined by hierarchical clustering following PCA, the Fisher Score analysis was carried out on data in PCA space, using the Python 3.6 scikit-feature library (35).
9) Dynamic Network Analysis (DyNA) (30) was used to define the central inflammatory network mediators as a function of both time and patient sub-group. Using inflammatory mediator measurements of at least three time-points per experimental group, networks were created over seven consecutive time periods (Admission-D1, D1–D2, D2–D4, D4–D5, and D5–D6) using MATLAB^®^ 8.1.0. Connections ([network edges] represent trajectories of inflammatory mediators [network nodes] that move in parallel; positive: same direction; negative: opposite direction) were created if the Pearson correlation coefficient between any two nodes (inflammatory mediators) at the same time-interval was greater or equal to a threshold of 0.7, as indicated. The network complexity for each time-interval was calculated using the following formula: Sum (N1 + N2 +…+ Nn)/(n − 1), where N represents the number of connections for each mediator and n is the total number of mediators analyzed. The total number of connections represents the sum of the number of connections across all time intervals for all patients in a given sub-group. In previous studies, we showed, that rising network complexity is associated with rising MODScores when comparing trauma survivors vs. non-survivors (10).
10) Spearman’s correlation was performed to measure the strength of the association between the Luminex™ data for two different mediators using a modified version of a MATLAB^®^-based toolbox described recently (10, 36). A *p*-value of less than 0.05 was considered significant.
11) Dynamic Bayesian Network (DyBN) inference was used to model the evolution of the probabilistic dependencies within a system over time. This analysis was carried out using MATLAB™ (The Math Works, Inc., Natick, MA), using an algorithm adapted from Grzegorczyk & Husmeier (37) and revised recently by our Group. In this analysis, inflammatory mediators were represented at multiple time points within the same network structure. In this approach, time was modeled discretely as in a discrete Markov chain. Each mediator was given a time index subscript indicating the time slice to which it belonged. Additional temporal dependencies were represented in a DyBN by edges between time slices. Each node in the network was associated with a conditional probability distribution of a variable that is conditioned upon its parents (upstream nodes). This particular network structure was used to assess the dominant inflammatory mediators and the probable interaction among various mediators, including possible feedback loops.

## Results

### Patient-Specific Principal Component Analysis/Hierarchical Clustering Segregates Blunt Trauma Patients Based on Core, Early Inflammatory Responses

The primary goal of the study was to define core, early (within the first 24 h) post-traumatic inflammatory endotypes of blunt trauma patients, and to determine if these inflammatory endotypes can predict the patients’ later clinical courses. Accordingly, we utilized patient-specific PCA followed by hierarchical clustering (28) to analyze the early post-traumatic inflammatory response of 236 blunt trauma patients (227 survivors and 9 non-survivors). Patients were grouped using hierarchical clustering based on inflammatory mediators assessed in plasma samples obtained at three timepoints within the first 24 h of admittance to the hospital. The sub-cohort of 227 survivors (148 males and 79 females; age = 50.4 ± 1.3 years [min: 18 years, max: 90 years]; ISS = 17.7 ± 0.6 [min: 1, max: 50]) was demographically similar though younger as compared to the sub-cohort of non-survivors (7 males and 2 females *p* = 0.7; age = 62.1 ± 7.2 years [min: 19 years, max: 86 years], *p* = 0.07; ISS = 19.9 ± 2.1 [min: 9, max: 33], *p* = 0.2).

We first analyzed time-course data from 31 circulating inflammatory mediators obtained over the first 24 h in the 227 survivors using patient-specific PCA followed by unsupervised hierarchical clustering, resulting in three main patient sub-groups (**Figure 1A**). We next sought to determine if non-survivors would co-cluster with these three sub-groups or form a separate cluster. Notably, the 9 non-survivors in the over cohort of 236 patients were segregated evenly across the three sub-groups defined using data from trauma survivors (**Figure S1**). Therefore, subsequent analyses were focused on the three sub-groups derived from the cohort of 227 trauma survivors, given the small number of non-survivors and the heterogenous causes of death typical of this population of trauma patients (10, 38, 39). These three groups had no statistically significant differences in their principal demographics, as follows (see details in **Table S1**):

1. Group 1 (n= 85): age: 45.3 ± 2.0 years (min: 18 years, max: 90 years), ISS: 18.8 ± 1.0 (min: 1, max: 50), gender: 51 males, 34 females
2. Group 2 (n = 41): age: 54.6 ± 3.1 years (min: 18 years, max: 86 years), ISS:16.6 ± 1.3 (min: 2, max: 38), gender: 30 males, 11 females
3. Group 3 (n= 101): age: 50.5 ± 1.9 years (min: 18 years, max: 89 years), ISS: 17.1 ± 0.9 (min: 1, max: 50), gender: 67 males, 34 females.

However, Group 1 was enriched for patients with head injury: (mean abbreviated injury scale 1 [AIS1, head injury]), where Group 1 had a significantly higher injury scale than Group 3 (Group 1: 1.14 ± 0.2, Group 2: 0.85 ± 0.2, Group 3: 0.22 ± 0.1 [*p*>0.99 Group 1 vs. 2; *p* = 0.0004 Group 1 vs. 3; *p*= 0.095 Group 2 vs. 3]) (**Figure S2A**). For the other body regions, there were no statistically significant differences across the three groups (**Figure S2B**).

### Significantly Different Clinical Outcomes in PCA/Clustering-Defined Trauma Sub-Groups

We next hypothesized that the three patient sub-group would differ in their clinical outcomes. In support of this hypothesis, we observed significant differences with regard to days on mechanical ventilation (Group 1: 2.3 ± 0.4 d vs. Group 2: 1.4 ± 0.9 d vs. Group 3: 2.5 ± 0.6 d; *p*= 0.03 overall; *p=* 0.02 Group 1 vs. 2), requirement for mechanical ventilation (Group 1: 44 on vs. 41 off ventilation vs. Group 2: 12 on vs. 29 off ventilation vs. Group 3: 39 on vs. 62 off ventilation; *p*= 0.04 overall; *p*= 0.02 Group 1 vs. 2), and Marshall MODscores over 7 days (*p*= 0.004 overall; *p* = 0.009 Group 1 vs. 2; *p*= 0.001 Group 2 vs. 3) across the three cohorts (**Figures 1B–D**). However, there were no significant differences in total hospital length of stay or ICU length of stay (**Figures S3A** **+** **S3B**). Additionally, there were no significant differences in other clinical parameters such as prevalence of nosocomial infection, prevalence and degree of hypotension, shock index, or comorbidities (**Figures S3C-E** **+** **S4** [see legend for exact values]).

### Distinct Dynamic Networks of Systemic Inflammation in Computationally Defined Trauma Patient Sub-Groups

**Figure 2:**
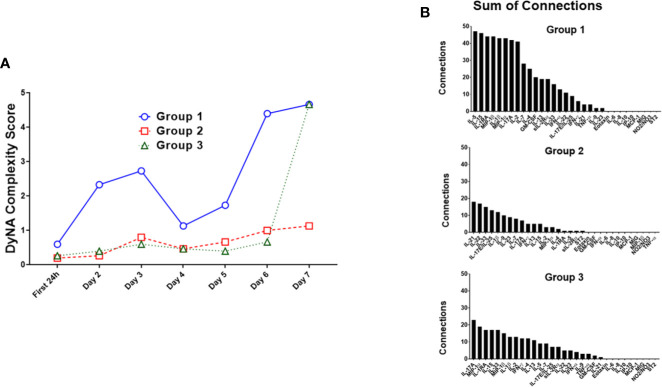
Dynamic Network Analysis (DyNA) suggests distinct dynamic inflammatory programs in trauma patient sub-groups. Dynamic Network Analysis was carried out on the systemic inflammatory mediator data of Groups 1–3, and network complexity was quantified as described in the *Materials and Methods*. **(A)** Group 1 had a consistently higher network complexity than the other two sub-groups, while Group 2 network complexity stayed consistently low without any peaks. Group 3 network complexity stayed low at the same level as Group 2 for the first 6 days before increasing towards the level of Group 1 at day 7. **(B)** The total network connections for each inflammatory mediator in each trauma survivor sub-group were tallied. Group 1 showed the highest degree of connectivity per mediator over a time course of 7 days, with the most connected mediators being IL-5, IL-15, IL-1RA, MIP-1β, and IL-1β. Group 2 and Group 3 showed similar degrees of individual mediator connectivity though at a lower level than in Group 1. The most connected mediators in Group 2 were IL-21, IL-22, IL-33, IL-17E/IL-25, and IL-1β, whereas the most connected mediators in Group 3 were IL-17A, MIP-1α, IL-1RA, IL-15, and IL-33.

We next characterized the dynamics of the circulating inflammatory mediators in each of the three computationally defined trauma patient subgroups. Out of the 31 measured inflammatory mediators, 23 were significantly different across the three sub-groups (**Table 2**). We next utilized Dynamic Network Analysis to define the dynamic inflammatory networks in the three sub-groups (**Figure 2A**). Group 1 had a consistently higher network complexity compared to the other two sub-groups, and this network complexity rose continually over the 7-day time course. Group 2 stayed consistently low without any peaks. During the first 6 days, the complexity of Group 3 remained low at the same level of Group 2, but then increased towards the level of Group 1 at day 7 (**Figure 2A**).

**Table 2:** Significantly different inflammatory mediators among Group 1 (n = 85), Group 2 (n = 41), and Group 3 (n = 101).

Inflammatory Mediator	p-value
Eotaxin	0.0256
GM-CSF	<0.0001
IFN-α	0.0012
IFN-γ	<0.0001
IL-1β	<0.0001
IL-1RA	<0.0001
IL-2	<0.0001
IL-4	<0.0001
IL-5	<0.0001
IL-7	<0.0001
IL-8	0.0426
IL-13	<0.0001
IL-15	<0.0001
IL-17A	<0.0001
IL-22	<0.0001
IL-23	0.0041
IL-17E/IL-25	0.0246
IL-33	0.0217
MIG	0.0419
MIP-1α	<0.0001
MIP-1β	<0.0001
sIL-2Rα	<0.0001
TNF-α	0.0102

We next investigated the degree of network connectivity of each mediator in each trauma patient sub-group over 7 days (**Figure 2B**). Group 1 had the highest degree of connectivity per mediator, with the most connected mediators being IL-5, IL-15, IL-1RA, MIP-1β, and IL-1β. Group 2 and Group 3 showed similar degrees of individual mediator connectivity though at a lower level than in Group 1. The most connected mediators in Group 2 were IL-21, IL-22, IL-33, IL-17E/IL-25, and IL-1β, whereas the most connected mediators in Group 3 were IL-17A, MIP-1α, IL-1RA, IL-15, and IL-33 (**Figure 2B**).

**Figure 3:**
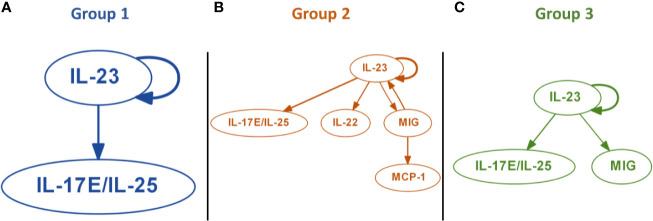
Dynamic Bayesian Network (DyBN) inference suggests distinct early inflammation programs in trauma patient sub-groups. Dynamic Bayesian Network inference was carried out on the systemic inflammatory mediator data of Groups 1–3 as described in the *Materials and Methods*. All groups contained a core motif consisting of IL-23 and IL-17E/IL-25, in which IL-23 was a central node. **(A)** Group 1 contained only the central motif of IL-23 and IL-17E/IL-25. **(B)** Group 2 showed the most complex inflammatory network, consisting of MCP-1 driven by MIG, along with of IL-22 driven by IL-23 and feedback by MIG. **(C)** Group 3 included MIG as a downstream mediator driven by IL-23.

We next sought to gain insight as to early drivers of inflammatory programs associated with each trauma endotype, using Dynamic Bayesian Network (DyBN) inference to define potential early feedback structures inherent in the initial 24-h data used to derive each of the three trauma sub-groups (**Figure 3**). All groups contained a core structure consisting of IL-23 and IL-17E/IL-25, in which IL-23 was a central node (meaning that it exhibited self-feedback as well as affecting downstream nodes). Group 1 contained only this central motif (**Figure 3A**), while Group 3 also included MIG as a downstream mediator driven by IL-23 (**Figure 3C**). Group 2 showed the most complex inflammatory network, being the only one with a third level of inflammatory mediators, consisting of MCP-1 driven by MIG. Another unique attribute was the presence of IL-22 driven by IL-23 and the feedback by MIG (**Figure 3B**).

**Figure 4:**
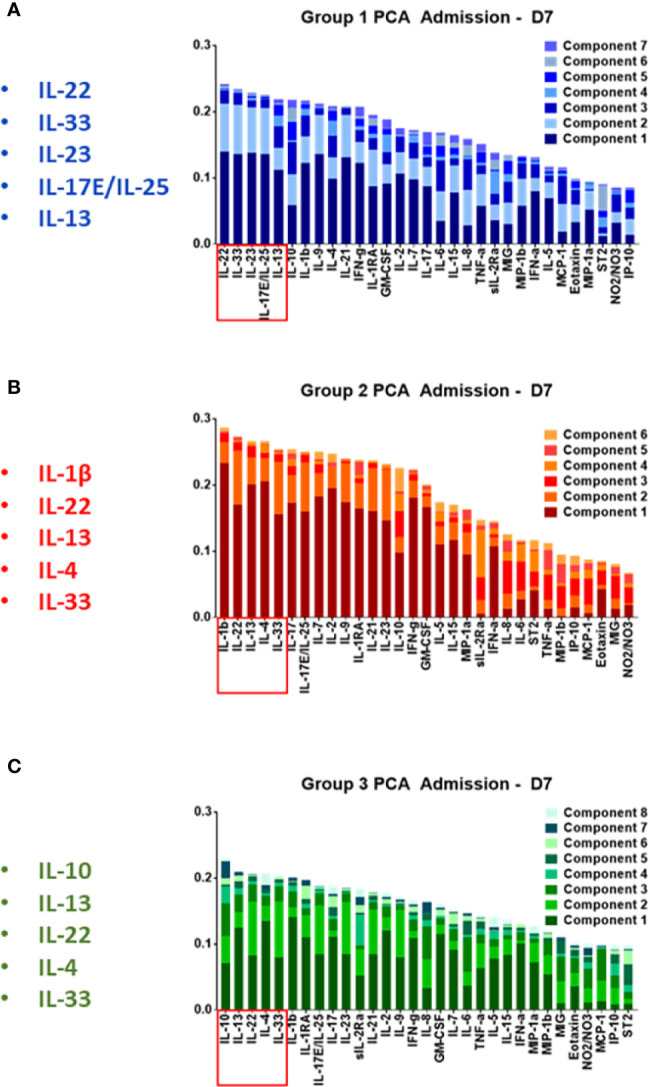
Principal component analysis of trauma patient sub-group data suggests a role for Type 3 immunity, in part regulated by Th17 cells, in the circulating inflammatory response to traumatic injury. Principal component analysis was carried out on the systemic inflammatory mediator data of Groups 1–3 as described in the *Materials and Methods*. **(A)** In Group 1, IL-22, IL-33, IL-23 IL-17E/IL-25, IL-13, and IL-10 were the principal characteristics. **(B)** In Group 2 patients, IL-1β, IL-22, IL-13, IL-4, IL-33, and IL-17A were the principal characteristics. **(C)** In Group 3, IL-10, IL-13, IL-22, IL-4, IL-33, and IL-1β were the principal characteristics.

Our next goal was to identify the principal characteristics, and thus possibly identify main differentiators, of the core, dynamic, systemic inflammatory responses in each trauma patient sub-group over the time course of the first 7 days after admission. We first utilized PCA to define principal characteristics of each trauma patient sub-group (**Figures 4A–C**). In Group 1, IL-22, IL-33, IL-23, IL-17E/25, and IL-13 were the most relevant inflammatory mediators (**Figure 4A**). In Group 2, IL-1β, IL-22, IL-13, IL-4, and IL-33 were the most relevant inflammatory mediators (**Figure 4B**). In Group 3, IL-10, IL-13, IL-22, IL-4, and IL-33 appeared as the principal characteristics of the inflammatory response (**Figure 4C**).

**Figure 5:**
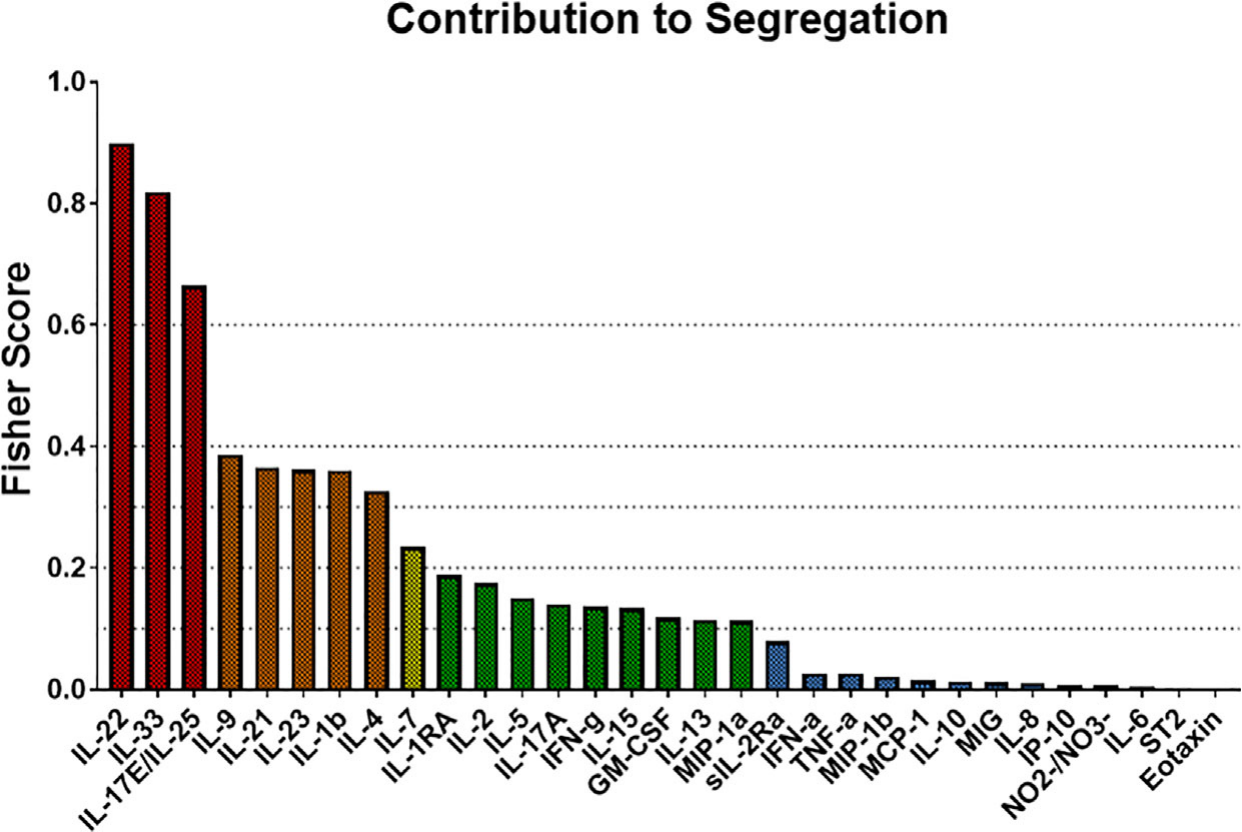
Fisher Score analysis points to Th17-related immune mediators the main differentiators among trauma patient sub-groups. Fisher Score analysis was carried out on the systemic inflammatory mediator data of Groups 1–3 as described in the *Materials and Methods*. The mediators IL-22, IL-33, and IL-17E/IL-25 were the mediators that best segregated among Groups 1–3, with IL-9, IL-21, IL-23, IL-1β, and IL-4 being the next best segregators.

We next used Fisher Score Analysis (35) to determine the inflammatory mediators that best segregated the three groups within the first 24 h. With a Fisher Score exceeding 0.6, IL-22, IL-33, and IL-17E/IL-25 were the most segregating mediators. The next most relevant mediators were IL-9, IL-21, IL-23, IL-1β, and IL-4, which exceeded a Fisher Score of 0.3 (**Figure 5**).

In line with the results of patient-specific PCA/hierarchical clustering, Fisher Score Analysis including the 9 non-survivors showed similar results in terms of contribution to the segregation but exhibited overall lower Fisher Scores (**Figure S5**). Taken together, these results point to differential Th17-related and epithelial-derived protective responses as the predominant early differentiators of systemic inflammation following blunt trauma.

### Spearman Correlation Suggests Distinct Dynamics of IL-17A–Producing T Cell Sub-Populations in Trauma Patient Sub-Groups

The cytokine IL-17A is a major component of innate immunity that contributes to systemic inflammation (40). Various cells produce this cytokine, including pathogenic Th17 cells that co-express IL-17A and GM-CSF and down-regulate IL-10 (in a manner that is potentiated by IL-23), as well as non-pathogenic Th17 cells, a reciprocal cell population that co-expresses IL-17A and IL-10 (41). We demonstrated recently that trauma non-survivors exhibit a positive correlation between IL-17A and GM-CSF—while exhibiting a negative correlation between IL-17A and IL-10—thus suggesting a shift toward pathogenic Th17 cells that characterize, and may be involved in, the systemic inflammation associated with mortality in trauma patients. In that study, matched survivors showed no correlation between IL-17A and either GM-CSF or IL-10 (10).

**Figure 6:**
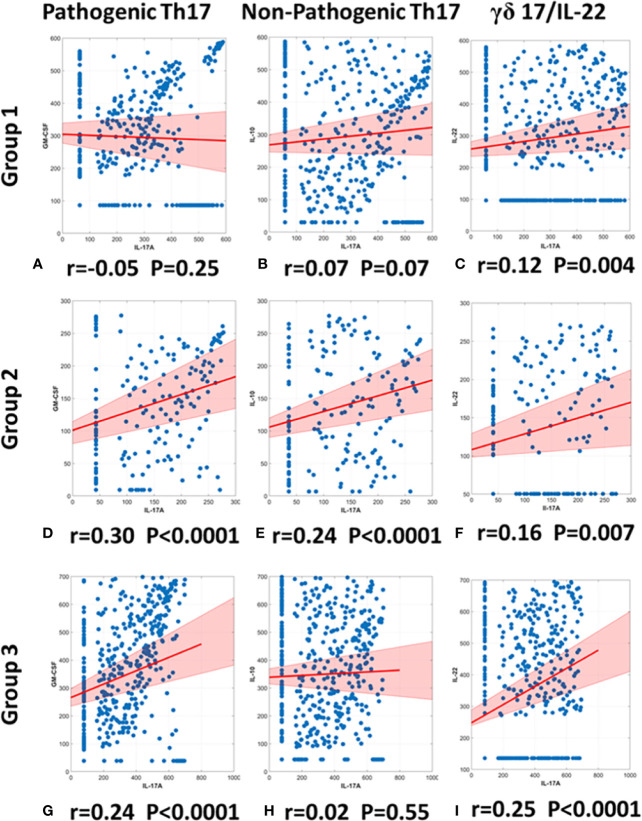
Spearman correlations of IL-17A vs. GM-CSF, IL-10, or IL-22 suggest differential presence of IL-17A–producing T cell subsets in trauma patient sub-groups. Spearman Correlations were carried out using the data on IL-17A, GM-CSF, and IL-10 from days 0 to 7 post-admission in Groups 1–3. Significant correlations between IL-17A and GM-CSF were inferred to suggest the presence of pathogenic Th17 cells, significant correlations between IL-17A and IL-10 were inferred to suggest the presence of non-pathogenic Th17 cells, and significant correlations between IL-17A and IL-22 were inferred to suggest the presence of γδ 17 T cells. Group 1 showed no correlation between either IL-17A and GM-CSF (r = −0.05, *p =* 0.25) **(A)** or between IL-17A and IL-10 (r = 0.0737, *p =* 0.0738) **(B)**. In contrast, Group 2 showed a positive correlation between IL-17A and GM-CSF (r = 0.30, *p <* 0.0001) **(D)** and a positive correlation between IL-17A and IL-10 (r = 0.24, *p <* 0.0001) **(E)**. Group 3 showed only a positive correlation between IL-17A and GM-CSF (r = 0.24, *p <* 0.0001) **(G)** and no correlation between IL-17A and IL-10 (r = 0.02, p = 0.55) **(F)**. Significant correlations between IL-17A and IL-22 suggest the presence of γδ 17 T cells in all groups: **(C)** Group 1 (r = 0.12, p = 0.004), **(F)** Group 2 (r = 0.16, p = 0.007), **(I)** Group 2 (r = 0.25, p < 0.0001).

We utilized the same methodology to gain insights into Th17 sub-populations in the three trauma patient sub-groups. Patients in Group 1 exhibited no significant correlation between IL-17A and GM-CSF (r= −0.05; *p*= 0.25; **Figure 6A**) nor between IL-17A and IL-10 (r= 0.07; *p* = 0.07 **Figure 6B**). In contrast, Group 2 patients showed a significant, positive correlation between IL-17A and GM-CSF (r= 0.30; *p*< 0.0001; **Figure 6D**), as well as a significant, positive correlation between IL-17A and IL-10 (r= 0.24 *p*< 0.0001; **Figure 6E**). Group 3 patients showed a significant, positive correlation between IL-17A and GM-CSF (r = 0.24; *p* < 0.0001; **Figure 6G**) but did not exhibit a significant correlation between IL-17A and IL-10 (r = 0.02; *p =* 0.55; **Figure 6H**). Thus, we suggest differential Th17 responses across the three sub-groups.

**Figure 7:**
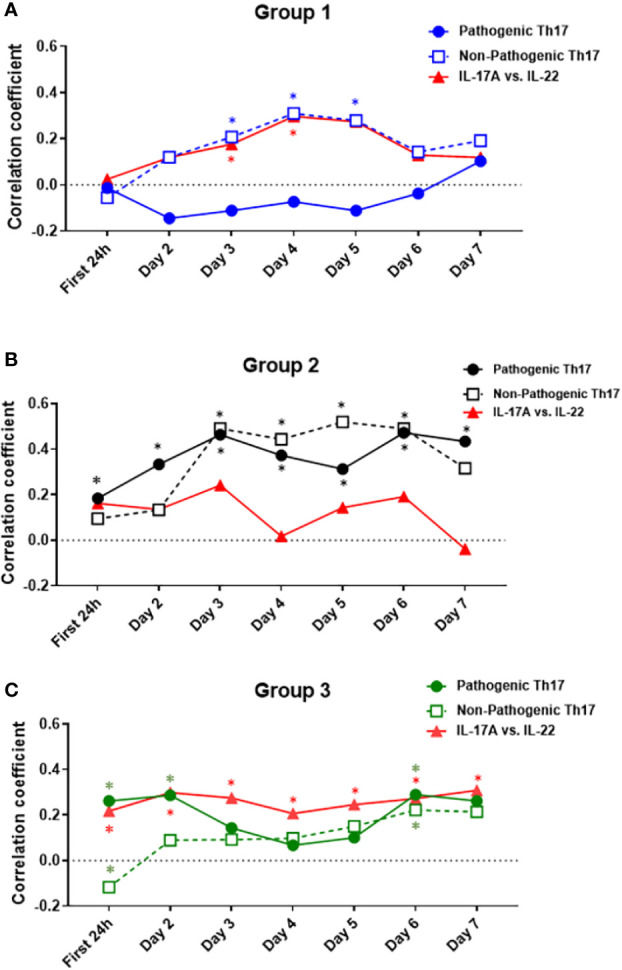
Dynamic Spearman correlations of IL-17A vs. GM-CSF, IL-10, or IL-22 suggest distinct trajectories of IL-17A–producing T cell subsets in trauma patient sub-groups. Spearman Correlations were carried out over 1-day time intervals using the data on IL-17A, IL-22, GM-CSF, and IL-10 in Groups 1–3. Significant correlations between IL-17A and GM-CSF were inferred to suggest the presence of pathogenic Th17 cells, significant correlations between IL-17A and IL-10 were inferred to suggest the presence of non-pathogenic Th17 cells, and significant correlations between IL-17A and IL-22 were inferred to suggest the presence of γδ 17 T cells. **(A)** In Group 1, non-pathogenic Th17 cells reached significant (*p* < 0.05) r values between days 3 and 5 post-admission and were inferred to predominate over pathogenic Th17 cells from day 2 to 7. **(B)** In Group 2, both pathogenic and non-pathogenic Th17 cell subsets showed similar r values, with a steady rise during the time course of 7 days after admission. **(C)** In Group 3, pathogenic Th17 cells appeared to predominate over non-pathogenic Th17 cells up to day 2. Thereafter, pathogenic and non-pathogenic Th17 cell subsets appeared to follow similar dynamics through day 7. Significant correlations between IL-17A and IL-22 were observed only in Group 1 (days 3–4) and Group 3 (all days). For exact r- and p-values, see **Table S1**.

γδ 17 T cells represent another IL-17A–producing immune cell population and are known to produce IL-17A and IL-22 (42, 43). To test for the potential presence of these cells, we carried out a Spearman correlation analysis for IL-17A vs. IL-22 in all three groups (**Figures 6C, F, I**). This analysis suggested the presence of γδ 17 T cells in Groups 1 and 3. We next segmented the correlation analyses over time by carrying out the same IL-17A/GM-CSF, IL-17A/IL-10, and γδ 17/IL-22 correlations over 1-day time intervals from hospital admission to 7 days post-admission (**Figure 7**, **Table S1**). This analysis suggested potentially differential dynamics of pathogenic and non-pathogenic Th17 cells as well as γδ 17 T cells. In Group 1, correlation values suggestive of the presence of non-pathogenic Th17 as well as γδ 17 T cells were highest from day 2 to day 7. In Group 2, inferred Th17 cell subset dynamics were similar for both pathogenic and non-pathogenic Th17 cell subsets, and γδ 17 T cells were not inferred. In Group 3, correlation values suggestive of γδ 17 T cells were inferred to predominate across all 7 days of observation. In contrast, pathogenic Th17 cells were apparent up to day 2, after which time this analysis suggested similar phenotypes of pathogenic and non-pathogenic Th17 subsets through day 7.

## Discussion

In the present study, we defined three early, dynamic systemic inflammatory endotypes in blunt trauma patients using individual-specific PCA combined with hierarchical clustering (28, 31), which also stratify the patients’ later clinical outcomes. These three endotypes are characterized by distinct dynamic inflammatory networks. In contrast to existing dogma in which early post-injury responses are dominated by classical innate immune cytokines such as TNF-α, IL-6, and IL-10, but supported by prior studies from our group and others (10, 11), our results suggest that pro-inflammatory type 3 (e.g., IL-17A and IL-23) and related, epithelium-derived, tissue-protective (IL-21, IL-22, and IL-25) responses might play a dominant role following severe blunt injury. The complex and dynamic interactions among these cytokines may result in distinct trajectories of Th17 cell subsets and γδ 17 cells, as inferred from a dynamic variant of correlation analysis utilized previously (10). These different core, early responses were also associated with distinct multiple organ dysfunction trajectories, reinforcing the concept that inflammation and organ dysfunction are intertwined processes (6, 26) and that organ dysfunction may be affected differentially depending on the presence of distinct cell subsets (10).

We have hypothesized previously that three states can emerge from the complex interplay of positive and negative feedbacks inherent in the dynamics of trauma-induced inflammation (5). An adaptive and properly regulated inflammatory response represent the optimal balance of positive and negative feedback and promotes tissue healing and regeneration. However, uncontrolled inflammation can become self-sustaining due to excessive positive feedback or insufficient negative feedback (4, 44). A third, less appreciated state likely involves excessive negative feedback, resulting in an inadequate/overly damped inflammatory response (5). The initial responses to trauma involve the local activation of tissue macrophages along with the influx and activation of monocytes and neutrophils (3, 45). Early studies in experimental animals and trauma patients also pointed to the impact of trauma on the lymphoid compartment, with a profound Th2 shift and immunosuppression following trauma/hemorrhagic shock (14, 46). Subsequent studies pointed to the early activation of regulatory T cells (Treg) following trauma, thereby providing one possible mechanism by which to account for this immunosuppression (46–48). These mechanisms may all contribute the well-established reduction in major histocompatibility complex class II expression and antigen presentation following trauma (7, 49–51).

A key inference from our studies is that differential dynamics of IL-17A–producing lymphocyte subsets may underpin the divergent systemic inflammatory responses of trauma patients. Th17 cells are generally pro-inflammatory, acting *via* the release of mediators such as IL-17A and IL-22 and play an important role in autoimmune diseases (e.g., Crohn’s disease) (52–54). Tregs release the generally anti-inflammatory and immunosuppressive mediators IL-10 and TGF-β1 (55, 56). Recent studies suggest that Th17 and Tregs play a major, early role in organ dysfunction (10, 11, 29, 46, 55), and point to the balance between so-called “pathogenic” (characterized by the release of IL-17A and GM-CSF) and “non-pathogenic” (characterized by the release of IL-17A and IL-10) Th17 cell subsets (41, 57) in the context of traumatic injury in humans (10, 29). Our results suggest that an upregulation of the “pathogenic” Th17 axis is associated with worse outcomes after trauma, in agreement with prior studies (10, 11). In extension of these previous findings (10, 11), our results further suggest a parallel upregulation of both “pathogenic” and “non-pathogenic” Th17 phenotypes as the optimal state following trauma, based on the trajectories of multiple organ dysfunction associated with each trauma endotype.

Our DyBN analyses implicated IL-23 as a core differentiator of the three computationally identified patient sub-groups. A key hallmark of these analyses was the differential impact on IL-22 in Group 2 but not Groups 1 or 3. This cytokine is produced predominantly by IL-23–polarized pathogenic Th17 cells. Notably, IL-23 is a major inducer of IL-22 by Th17 cells (58). Thus, our DyBN results further support a role for Th17 and γδ 17 T cells in the differential inflammatory and clinical trajectories of these patient sub-groups. Functionally, IL-22 is modulated by the transcription factors STAT3, RORγt, and aryl hydrocarbon receptor (58). Notably, Th17 cell differentiation and plasticity are regulated to a large extent by RORγt; thus, future studies could address the potential roles of these transcription factors in relevant tissues following severe traumatic injury.

IL-22 may also play a role as a product of γδ 17 T cells, which represent another IL-17A–producing immune cell population that has been implicated in inflammatory pathology (42, 43). Importantly, various studies suggest that these cells may respond more rapidly than Th17 cells and thereby accelerate Th17-mediated inflammatory responses (42). These cells are known to produce IL-17A and IL-22 (42). Our correlation analyses suggest the presence of these cells in Groups 1 and 3, and that these cells play a particularly important role in the responses of Group 3.

A surprising result, in line with our core hypothesis of three dynamic trajectories of inflammation (5), is that overly damped inflammation (Group 3), like overly exuberant inflammation (Group 1), is associated with prolonged multiple organ dysfunction and adverse clinical outcomes including longer ICU stays and days on mechanical ventilation. Our studies are in agreement with the notion of a dynamic process that starts with a pro-inflammatory phase, which in turn initiates a repair response in which key inflammatory cells switch their phenotypes from pro-inflammatory to repair (4). Studies in our group utilizing a larger trauma patient cohort that includes the 227 survivors studied herein further support this notion, finding that 8 inflammatory mediators (IL-22, IL-9, IL-33, IL-21, IL-23, IL-17E/25, IP-10, and MIG) were significantly suppressed during the initial 24 h and up to day 7 post-injury in survivors with severe injuries (ISS ≥25) vs. survivors with mild (ISS = 1–15) or moderate (ISS: 15–24) injury (Cai et al., submitted). Our suggestion that γδ 17 T cells, which produce IL-22, are present predominantly in Group 3 may indicate a potential role for IL-22 in dampening this inflammatory response. In addition, pathogenic Th17 cells can differentiate into regulatory T cells (59, 60), and it is intriguing to speculate that this plasticity might be involved in the phenotype of overly damped inflammation in trauma patients.

Our studies also point to a process for defining endotypes in other inflammatory disease settings. We employed a stepwise series of computational analyses involving individual-specific PCA combined with unsupervised hierarchical clustering to segregate core inflammatory response, defining pathways in each group by inferring dynamic networks, and delineating the mediators that segregate across groups using Fisher score analysis. A key aspect of this approach is that it encompasses a dynamic response in a single data vector [an “inflammation barcode” (28)] through the use of individual-specific PCA. This methodology is likely expandable to include other types of data (e.g., single-cell RNA sequencing, metabolomics, etc.) as well as being applicable to other complex disease settings. While data-driven analyses such as PCA are, strictly speaking, not amenable to direct biological interpretation (32), we have utilized PCA to suggest key pathways involved in porcine endotoxemia and used these insights to structure individual-specific mechanistic mathematical models of inflammation (61). We have also used PCA in concert with hierarchical clustering to segregate the core systemic inflammatory responses of patients suffering secondary to trauma (28) (Gruen et al., unpublished observations) as well as acute liver failure (31). In contrast to the present study, our prior studies used this approach strictly as a means of differentiating patients based on early inflammatory dynamics in settings in which the unprocessed data could not segregate patient subgroups, without addressing the specific pathways characteristic of each subgroup (28, 31).

While we have implicated type 17 immunity previously in the initiation and propagation of self-sustaining systemic inflammation associated with a small subset of patients that go on to die following admission to the ICU subsequent to traumatic injury, the computational strategy we employed now leads us to hypothesize that type 17 immunity and the protective cytokines that regulate this pathway are central to the inflammatory response of all blunt trauma patients. Despite limitations that include the inclusion of only blunt (not penetrating) trauma patients, the focus on a subset of possible inflammatory mediators, and the lack of a multi-center study design, our results suggest that greater diagnostic and therapeutic focus should be paid to type 17 and associated responses in the setting of traumatic injury.

## Data Availability Statement

All datasets presented in this study can be found in the **Supplementary Material**.

## Ethics Statement

The studies involving human participants were reviewed and approved by University of Pittsburgh School of Medicine. The patients/participants provided their written informed consent to participate in this study.

## Author Contributions

LS conceived the study, performed the analyses, wrote the manuscript, and edited the manuscript; HL, RN, QM, AA, and RZ performed the analyses and edited the manuscript. MC, JY, DB, and FE-D performed the analyses. AA and TB edited the manuscript. YV conceived the study, wrote the manuscript, and edited the manuscript. All authors contributed to the article and approved the submitted version.

## Funding

This work was supported by the Office of the Assistant Secretary of Defense for Health Affairs, through the Defense Medical Research and Development Program under Award No. W81XWH-18-2-0051 and W81XWH-15-PRORP-OCRCA. Opinions, interpretations, conclusions, and recommendations are those of the authors and not necessarily endorsed by the Department of Defense. Creation of the patient database and biobank was partially supported by NIH grant P50-GM-53789.

## Conflict of Interest

YV is a co-founder of, and stakeholder in, Immunetrics, Inc.

The remaining authors declare that the research was conducted in the absence of any commercial or financial relationships that could be construed as a potential conflict of interest.
