# Supplementary material for: Computational Derivation of Core, Dynamic Human Blunt Trauma Inflammatory Endotypes
Source: Front Immunol. 2021 Jan 18;11:589304. doi: 10.3389/fimmu.2020.589304 (PMC7848165; doi:10.3389/fimmu.2020.589304)
Supplement: Supplementary file 1 [file DataSheet_1.pdf]

Supplementary Material

Supplementary Figures

Suppl. Fig. 1

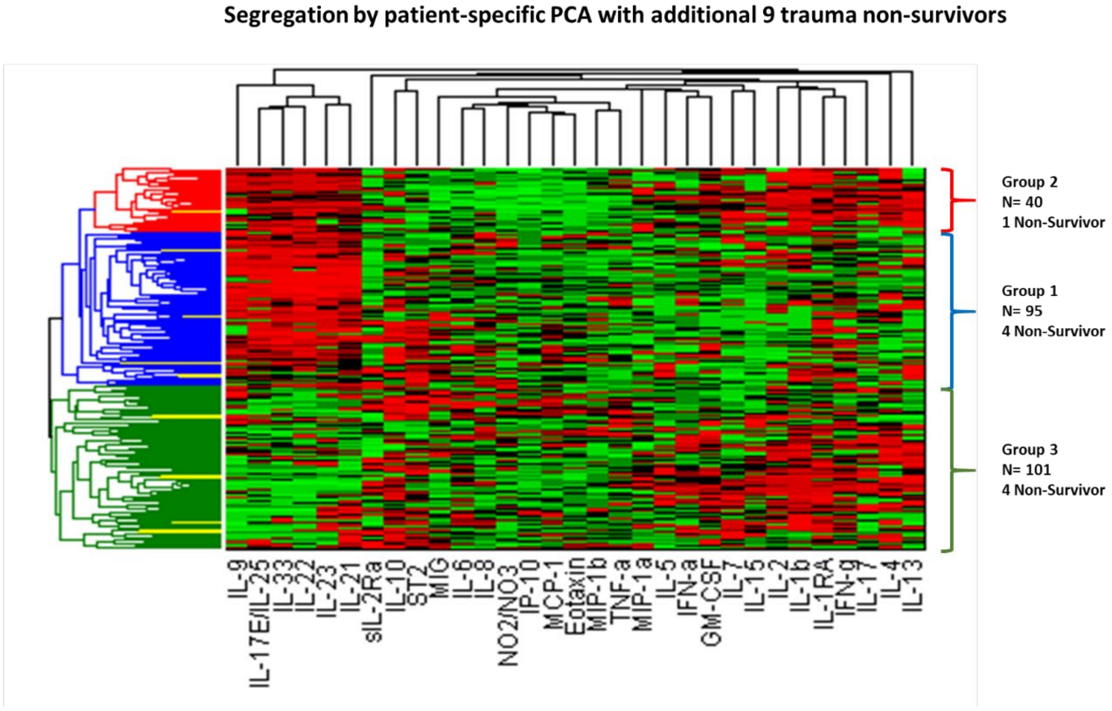

Supplementary Figure 1. Patient specific Principal Component Analysis with additional 9 blunt trauma non-survivors yielded similar groups. The 9 non-survivors (NS) were segregated evenly across the three sub-groups defined using data from trauma survivors (4 NS in Group 1, n= 95; 1 NS in Group 2, n=40; 4 NS in Group 3, n=101). NS are highlighted in yellow.

Suppl. Fig. 2

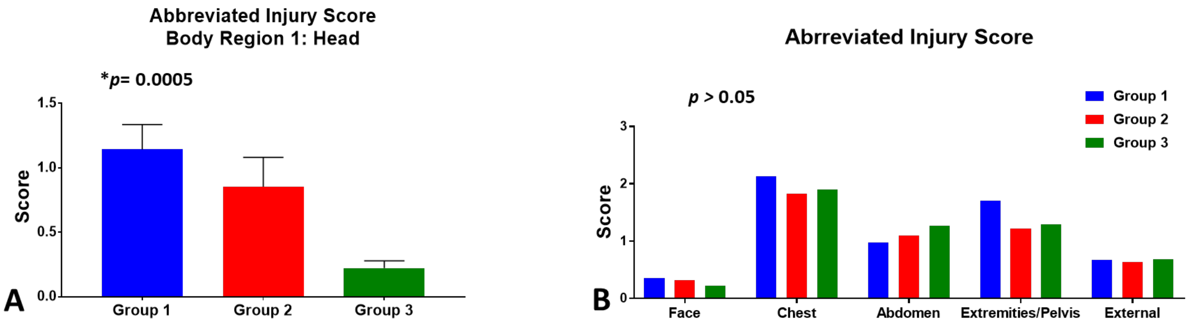

Supplementary Figure 2. Group 1 showed a significantly higher mean abbreviated injury score 1, but similar mean abbreviated injury scores for the remaining body regions. A) Group 1 had a significantly higher mean abbreviated injury score 1 (AIS1, head injury) than Group 3 (Group 1: 1.14

± 0.2, Group 2: 0.85 ± 0.2, Group 3: 0.22 ± 0.1 (p>0.99 Group 1 vs. 2; p =0.0004 Group 1 vs. 3; p= 0.095 Group 2 vs. 3). B) Group 1, Group 2, and Group 3 showed similar AIS scores for the remaining 5 body regions (face, chest, abdomen, extremities/pelvis, and external).

Suppl. Fig. 3

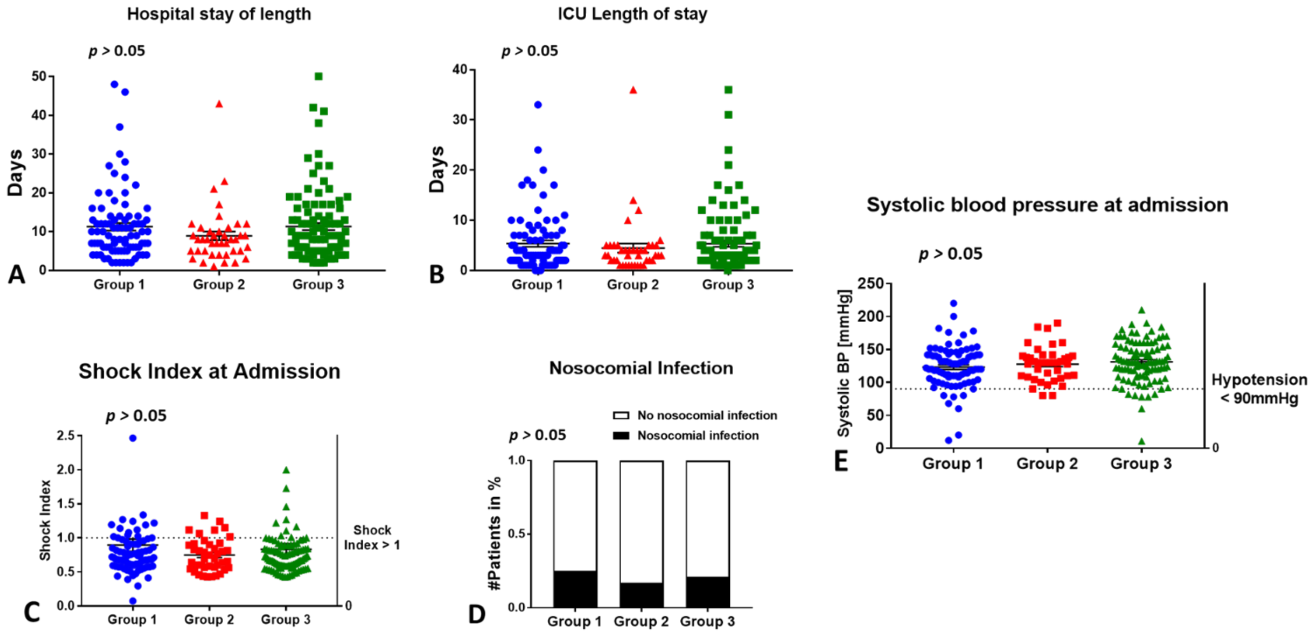

Supplementary Figure 3. No significant differences in additional clinical outcomes across Group 1, Group 2, and Group 3. Analyses of additional clinical outcomes such as hospital length of stay (Group 1: 11.3 ± 1 d vs. Group 2: 8.9 ± 1.1 d vs Group 3: 11.3 ± 0.9; p= 0.27 [A] , ICU length of stay (Group 1: 5.4 ± 0.6 d vs. Group 2: 4.5 ± 0.9 d vs Group 3: 5.3 ± 0.6; p= 0.88 [B]), shock index at admission (Group 1: 0.89 ± 0.09 vs. Group 2: 0.75 ± 0.04 vs. Group 3: 0.83 ± 0.09; p= 0.58 [C]), prevalence of nosocomial infections (Group 1: 21 with vs. 64 without nosocomial infection vs. Group 2: 7 with vs. 34 without nosocomial infection vs. Group 3: 21 with vs. 80 without nosocomial infection; p= 0.6 [D]), and prevalence and degree of hypotension (Group 1: 7 with vs. 78 without hypotension vs. Group 2: 2 with vs. 39 without hypotension vs. Group 3: 7 with vs. 94 without hypotension; p= 0.79 [E]) did not show significant differences.

Suppl. Fig. 4

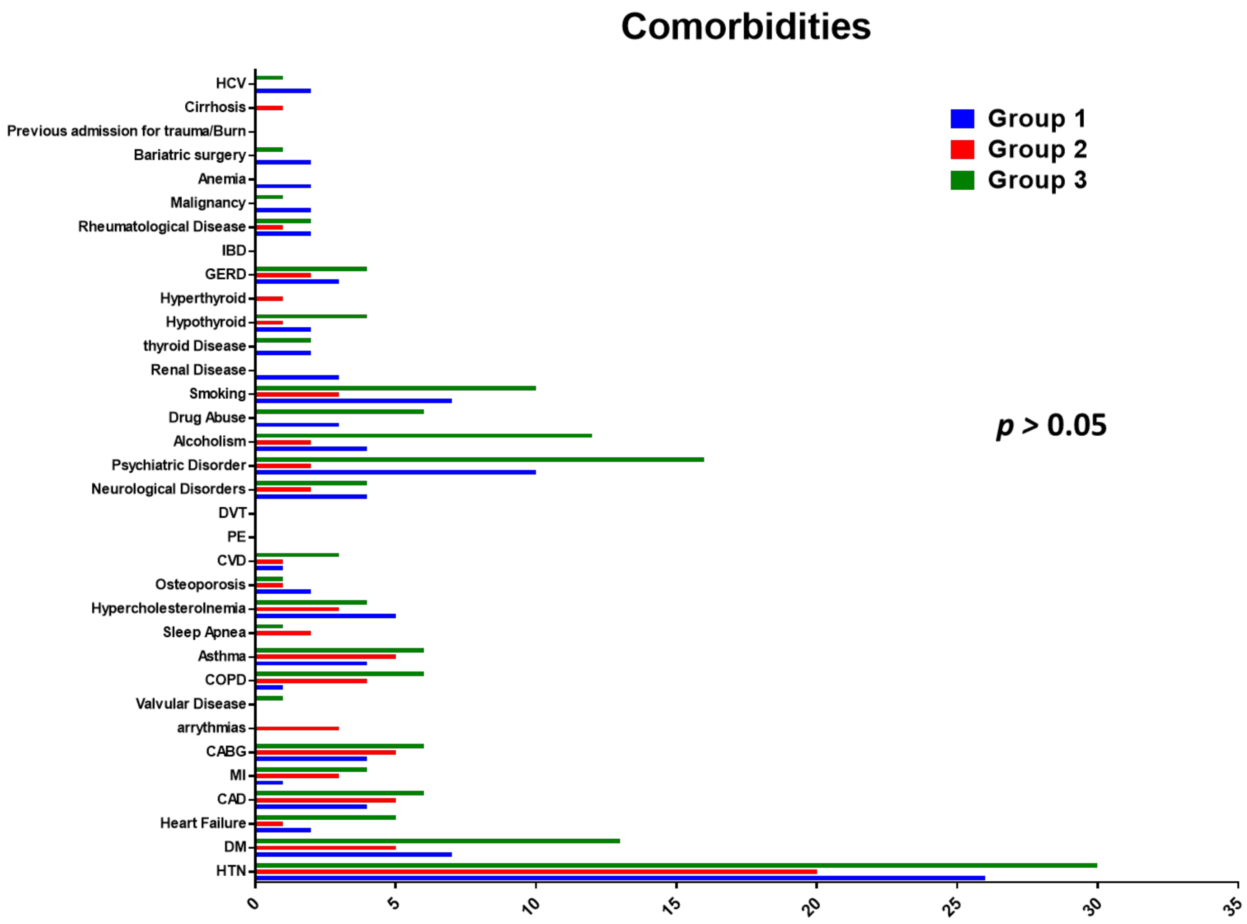

Supplementary Figure 4. Comparable comorbidities across Group 1, Group 2, and Group 3. Abbreviations: Hypertension (HTN), Diabetes mellitus (DM), Coronary artery disease (CAD), Myocardial infarction (MI), coronary artery bypass graft (CABG), Chronic obstructive pulmonary disease (COPD), Cardivascular disease (CVD), Pulmonary embolism (PE), Deep vein thrombosis (DVT), Gastroesophageal reflux disease (GERD), Inflammatory bowel disease (IBD), Hepatitis C Virus (HCV).

Suppl. Fig. 5

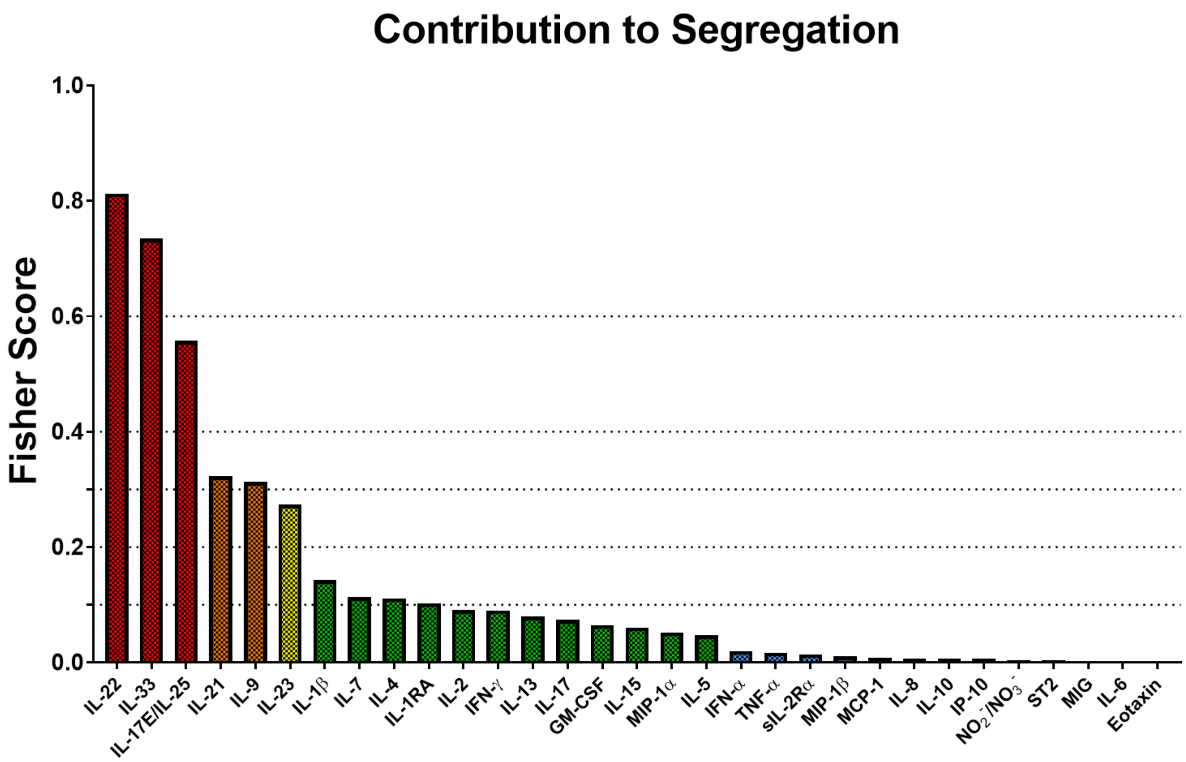

Supplementary Figure 5. Fisher Score analysis including nine non-survivors does not change the overall PCA/clustering results. Patient-specific PCA and subsequent unsupervised hierarchical clustering were carried out as in Fisher Score Analysis of the full trauma patient cohort, including 226 survivors and 9 non-survivors, showed similar results in terms of contribution to the segregation but exhibited overall lower Fisher Scores. In this analysis, the mediators IL-22 and IL-33 and exhibited a Fisher Score greater 0.6 and were therefore the most segregating. The next most relevant mediators were IL-17E/IL-25, IL-21, and IL-9, exceeding a Fisher Score of 0.3.

Supplementary Tables

Supplementary Table 1. Dynamic Spearman correlations scores of IL-17A vs. GM-CSF or IL-10 and γδ 17 vs. IL-22. Exact r-values and p-values of Dynamic Spearman correlations (see Fig. 7).

Group 1	Pathogenic Th17	p-value	Non-Pathogenic Th17	p-value	γδ 17/IL-22	p-value
First 24h	-0.01285	0.8367	-0.05589	0.3694	0.0253	0.6859
Day 2	-0.1443	0.0867	0.1199	0.1551	0.1191	0.1594
Day 3	-0.1113	0.2679	0.2074	*0.0374	0.1761	0.0798
Day 4	-0.07285	0.4853	0.3097	*0.0024	0.2972	*0.0038
Day 5	-0.1107	0.2991	0.2791	*0.0077	0.2738	*0.0090
Day 6	-0.03677	0.7541	0.1431	0.2207	0.1291	0.2696
Day 7	0.1032	0.4802	0.1918	0.1867	0.1190	0.4204

Group 2	Pathogenic Th17	p-value	Non-Pathogenic Th17	p-value	γδ 17/IL-22	p-value
First 24h	0.1845	*0.0349	0.09531	0.2788	0.1622	0.0685
Day 2	0.3334	*0.0058	0.1339	0.2801	0.1358	0.2769
Day 3	0.4637	*0.0015	0.4907	*0.0007	0.2416	0.1232
Day 4	0.3732	*0.0149	0.4436	*0.0033	0.0174	0.9151
Day 5	0.3129	*0.0411	0.5195	*0.0004	0.1440	0.3691
Day 6	0.4725	*0.0027	0.4902	*0.0018	0.1916	0.2629
Day 7	0.4341	*0.0493	0.3162	0.1626	-0.0381	0.8771

Group 3	Pathogenic Th17	p-value	Non-Pathogenic Th17	p-value	γδ 17/IL-22	p-value
First 24h	0.2626	*<0.0001	-0.1168	*0.0373	0.2175	*<0.0001
Day 2	0.2871	*0.0003	0.08985	0.2694	0.2993	*0.0002
Day 3	0.1437	0.1205	0.09266	0.3183	0.2758	*0.0027
Day 4	0.06759	0.4595	0.09828	0.2815	0.2071	*0.0227
Day 5	0.1011	0.2933	0.1502	0.1174	0.2463	*0.0098
Day 6	0.2904	*0.0052	0.2222	*0.0342	0.2732	*0.0092
Day 7	0.2626	0.0505	0.2145	0.1124	0.3087	*0.0206
