# Supplementary material for: Computational Derivation of Core, Dynamic Human Blunt Trauma Inflammatory Endotypes
Source: Front Immunol. 2021 Jan 18;11:589304. doi: 10.3389/fimmu.2020.589304 (PMC7848165; doi:10.3389/fimmu.2020.589304)
Supplement: Supplementary file 2 [file DataSheet_2.pdf]

PatientID	time	Eotaxin	GM-CSF	IFN-a	IFN-g	IL-1b	IL-1RA	IL-2	IL-4	IL-5	IL-6	IL-7	IL-8	IL-9	IL-10	IL-13	IL-15	IL-17	IL-17E/IL-2	IL-21	IL-22	IL-23	IL-33	IP-10	MCP-1	MIG	MIP-1a	MIP-1b	NO2/NO3	sIL-2Ra	ST2	TNF-a
PatientID	time	Eotaxin	GM-CSF	IFN-a	IFN-g	IL-1b	IL-1RA	IL-2	IL-4	IL-5	IL-6	IL-7	IL-8	IL-9	IL-10	IL-13	IL-15	IL-17	IL-17E/IL-2	IL-21	IL-22	IL-23	IL-33	IP-10	MCP-1	MIG	MIP-1a	MIP-1b	NO2/NO3	sIL-2Ra	ST2	TNF-a
p95	Admission	18.3938	133.3318	146.8346	35.8086	121.884	3401.887	14.1268	18.8711	13.8391	834.0011	45.0276	28.9312	0	0	27.8035	45.5688	56.8247	0	6.11	0	0	3.67	37.1974	374.8643	124.4541	62.0155	769.1945	28.103	516.7801	94537.38	28.3116
p95	D1	25.9481	39.2391	0	12.2932	123.1483	0	14.1268	7.0313	1.5213	664.2194	15.9921	56.0757	0	0	14.2535	0	0	0	0	0	0	0	23.5036	543.2169	0	22.5198	2320.233	28	414.4592	174086.7	20.8178
p95	D4	34.3296	288.3016	178.9395	57.3726	275.5819	85.4821	58.6669	37.8489	29.4027	795.6653	122.2914	28.9312	0	0	29.5595	75.1748	96.7203	120	10.78	0	0	16.59	44.1631	564.0499	215.8858	106.0876	1498.337	12	1245.087	170659.7	48.0341
p95	D7	66.4979	264.3564	193.3517	67.5545	312.7961	0	58.6669	34.8944	29.1408	204.6362	151.5809	15.4533	0	0	30.8486	80.3101	99.2922	0	4.88	0	0	8.2	33.4119	415.9288	212.6527	99.9391	1708.862	27	1043.573	76061.69	39.4266
p70	Admission	102.8528	481.4356	332.0502	148.3343	287.9232	3544.008	68.8729	96.9783	65.3028	629.6354	172.375	24.2194	42.85	57.7711	55.7783	206.7635	258.2099	960	77.53	788	3410	124	18.0336	478.7985	485.7829	208.4076	2186.255	32.25	1187.379	49968.72	68.9603
p70	D1	66.9928	666.4607	390.885	170.2692	349.8072	2007.406	137.9562	128.8643	78.8564	237.3494	223.3302	18.9088	35.73	42.7906	75.8776	225.6124	299.2261	660	63.84	539	2330	77.4	23.5433	371.2483	525.3345	260.2332	2267.063	31	1485.404	72539.27	120.1885
p70	D4	66.4979	302.9498	239.6622	106.5195	183.992	200.3697	30.6876	52.5525	37.2118	119.9754	88.5409	13.8193	51.76	6.3501	37.782	105.3866	146.0373	1720	87.62	1220	6120	179	13.8034	313.4499	309.7757	106.0876	1463.795	25	822.1938	17189.03	41.8873
p70	D7	58.2078	927.0447	555.5522	280.5608	494.6626	5335.32	203.9992	206.0296	141.0959	328.4613	299.1099	32.4362	47.8	68.3564	85.7343	394.0356	452.1784	1350	73.93	1181	4260	126	23.1465	512.6405	868.328	312.4231	4273.972	12	2010.009	19087.79	125.8977
p62	Admission	34.5952	252.8197	213.4865	86.0695	189.2578	1261.49	29.0092	45.6986	31.2419	886.9442	90.3632	26.3629	27.56	8.725	27.8035	62.3309	144.054	1260	81.86	1076	3470	155	17.4693	584.8386	253.64	107.5927	1390.193	17	998.0638	140379.8	41.8873
p62	D1	42.2971	443.8169	338.8442	179.6207	299.1827	475.025	93.419	99.4811	77.8608	2876.127	170.7529	25.6165	49.46	39.2224	66.128	185.7496	240.7142	1260	114	981	4340	135	78.5594	1290.424	426.3138	210.5269	2256.43	13.6	1442.129	218509.3	103.7971
p62	D4	43.7145	499.0356	339.9664	177.8414	343.3537	845.9023	130.2217	107.2691	75.4476	604.0781	163.9205	18.7825	19.44	25.8339	50.3113	171.188	268.3483	420	56.28	220	1430	50.88	120.3817	1224.581	477.9066	210.5269	2335.123	21	1665.404	33691.04	110.0838
p59	Admission	38.7998	52.0187	75.4751	20.3251	83.6097	10520.1	8.4263	5.8471	7.0973	1526.589	41.9002	22.2728	0	73.2464	26.0001	27.2635	22.6841	0	7.4	0	0	10.36	17.1462	495.9388	34.3242	51.7217	411.4072	25	501.643	217260.4	24.5778
p59	D1	39.7292	489.7305	329.7617	112.0358	221.5947	3633.184	58.2266	91.4876	60.1403	533.6761	177.2027	18.6142	31.09	72.9746	45.3244	173.8288	210.4219	860	59.01	646	2930	75.48	22.352	548.6228	447.2039	181.3704	2156.483	24	1528.511	389554.9	64.0266
p59	D2	41.7657	872.9189	463.5175	191.609	381.3515	3225.232	145.7514	181.7325	105.8329	397.7167	228.6348	22.9625	20.7	45.2524	79.1173	257.9705	305.6454	280	31.88	52.62	660	36.05	45.2759	578.6831	628.1933	287.6955	3105.177	12	1918.327	44151.43	139.2919
p59	D5	71.9535	1062.203	557.1091	260.374	474.3551	4155.438	188.6515	191.9342	142.2974	258.6109	300.4104	37.0118	41.67	60.214	92.9901	326.6865	374.4772	1300	66.22	830	3440	102	27.3015	473.333	698.1186	296.6319	3426.148	16.784	2043.223	81536.76	150.8547
p56	D2	23.4096	156.4141	118.3865	50.4753	112.8663	1277.242	12.4952	25.9595	22.7856	357.1263	50.9765	6.8439	11.85	11.9393	21.239	69.3971	88.0377	410	35.58	180	660	45.36	28.8008	351.8761	196.9145	83.4794	922.1954	11	669.6883	106947.3	30.7905
p56	D3	15.7294	128.104	113.0909	34.2536	102.1589	1308.816	10.8664	19.8955	16.8225	311.1828	41.3674	5.4323	21.18	7.0429	21.239	57.1853	67.2769	720	40	717	1610	70.62	21.9543	386.4834	135.1136	70.0495	771.5046	16.4	616.0997	104084.6	27.0694
p54	Admission	49.6579	106.7639	37.0623	35.2912	172.169	2141.031	40.0099	15.2408	11.878	466.1225	51.9337	3.3515	0	20.6595	15.9757	44.9212	59.2377	100	9.74	0	0	18.61	15.3602	239.1029	113.47	82.3144	602.5706	24.659	746.5203	165311.4	30.1714
p51	Admission	38.5785	58.1784	71.6357	19.2088	137.9102	440.1602	11.6805	7.6109	5.0209	644.6838	45.0276	79.4888	0	0	21.239	25.2707	18.9192	0	0	0	0	0	14.7066	1194.271	47.1049	62.7091	612.1129	47.558	645.4127	361588.6	28.3116
p51	D1	64.0272	382.0685	295.0666	92.1526	225.4907	1825.406	39.5829	60.3881	42.4507	1555.555	117.5305	45.2308	5.84	41.1457	44.5942	118.9463	155.8829	290	16.81	154	260	36.99	24.2568	1472.407	321.3525	145.9258	1755.924	24	1222.937	392446.1	57.8699
p455	Admission	49.12	6.71	23.39	12.06	14.6	113.41	7.14	15.71	4.24	8.93	14.52	8.16	0	14.49	13.23	11.79	4.45	530	10.13	0	0	17.51	70.57	263.45	100.75	0	10.97	26.803	24.53	38179.12	4.77
p455	Admission	59.91	5.84	21.21	8.85	8.71	71.9	5.13	15.22	3.23	14.39	10.59	9.62	0	12.02	9.2	9.24	3.4	250	7.04	0	0	0	204.3	328.43	109.65	0	7.74	46	26.46	36599.93	2.98
p455	Admission	47.04	5.37	7.11	6.59	7.85	61.04	4.73	3.51	2.82	10.33	8.66	6.45	6.98	12.33	6.61	9.33	3.5	1100	30.06	148	900	81.65	162.93	221.94	130.76	0	5.28	8.556	29.02	52430.3	1.86
p455	D1	31.8	3.05	0	0	0.67	15.38	2.12	0	1.82	19.44	2.31	9.67	8.37	10.17	0	7.65	1.09	1370	29.64	123	1000	81.65	242.2	367.92	270.36	0	0	13.603	21.28	52406.3	1.27
p455	D2	36.57	3.36	0	1.66	3.46	37.51	2.85	0	2.04	135.43	3.01	16.33	16.1	10.48	2.33	8.87	1.66	1880	36.8	197	1460	110	649.15	465.58	277.07	0	8.16	9.263	43.11	126691	2.71
p455	D3	22.1	3.01	0	0	0	20.52	2.57	0	1.89	7.17	3.64	1.95	0	3.29	0.65	6.4	1.09	620	9.34	0	220	38.79	174.37	154.23	190.57	0	0	8.315	31.75	48302.31	0
p455	D4	13.58	2.67	0	0	1.47	15.38	2.12	0	1.68	10.4	0	5.37	0	6.09	0	8.31	0.88	250	2.27	0	0	0	57.99	90.15	188.92	0	0	6.816	24.75	103377.4	0
p455	D5	24.99	3.33	0	6.27	3.76	42.57	4.04	2.26	2.41	3.56	8.66	3.09	0	8.05	5.22	7.17	2.66	0	1.19	0	0	0	142.55	121.58	169.18	0	0	4.592	29.23	32495.93	0.11
p455	D6	27.41	3.08	0	0	1.63	17.96	2.91	0	1.87	6.84	2.31	2.67	0	4.76	1.12	6.2	1.29	0	0	0	0	0	103.6	91.31	181.51	0	0	8.073	27.32	17889.55	0
p454	Admission	34.16	5.53	18.98	13.98	8.42	139.82	5.47	10.36	2.97	39.62	11.14	11.61	0	63.49	8.92	8.78	4.13	510	86.69	500	7160	66.24	173.08	264.8	497.37	0	21.98	20.91	53.37	36049.2	5.97
p454	Admission	17.36	4.31	3.89	9.82	4.21	30.74	4.1	3.11	2.56	58.6	10.41	8.37	0	30.02	6.3	8.22	2.55	490	72.65	367	5080	35.5	134.49	232.17	247.8	0	10.19	12.354	66.19	219750.4	3.39
p454	D1	20.74	2.53	0	6.75	0	10.21	1.9	0	1.87	106.06	7.62	15.96	0	14.34	15.53	7.74	1.29	0	0	0	0	0	222.73	290.54	463.59	0	9.4	8.794	111.95	196201.8	4.44
p454	D1	19.07	2.8	0	3.65	0	9.34	2.68	0	1.77	79.11	4.77	12.22	0	15.27	4.09	7.17	1.19	0	26.2	0	2350	0	222.21	247.93	307.34	0	12.67	9.494	117.18	198961.4	3.55
p454	D2	19.21	2.53	0	1.5	0	9.34	2.06	0	1.7	100.06	3.64	15.21	0	11.71	6.9	7.93	0.98	0	0	0	0	0	335.53	282.93	483.49	0	12.11	8.794	141.98	194989.2	6.32
p454	D4	19.35	3.4	13.42	7.88	2.25	23.94	5.3	6.59	2.51	20.25	10.41	8.68	0	7.44	5.22	7.65	2.97	0	34.21	0	2770	0	153.46	144.09	303.97	0	0	8.315	102.38	72212.6	2.15
p454	D6	34.97	3.19	0	6.59	0	35.82	2.68	0	2.06	17.75	8.46	8.7	0	12.17	6.3	6.4	1.92	0	0	0	0	0	237.84	268.84	301.44	0	5.28	5.466	134.24	57917	3.52
p453	Admission	29.02	11.88	42.93	16.53	13.21	82.73	8.35	22.57	4.05	10.95	18.59	7.28	34.75	37.95	10.31	11.34	5.4	3550	85.09	635	4080	229	219.57	173.36	95.91	0	25.82	19.433	43.92	7358.363	4.35
p453	Admission	11.8	8.36	34.85	11.74	11.67	74.4	6.56	19.68	3.29	9.02	17.28	5.71	48.91	15.12	10.72	10.07	4.87	3240	143	552	4400	250	126.9	68	70.96	0	15.36	7.827	58.73	76838.27	1.79
p453	Admission	7.95	6.07	16.66	6.27	4.06	32.43	5.01	8.68	2.43	5.99	9.26	5.89	29.91	8.96	5.38	8.87	3.03	2670	86.69	486	3810	195	129.77	94.74	67.75	0	10.97	8.794	90.46	146620.6	1.34
p453	D1	7.57	8.44	0	0	0	6.73	1.56	0	1.59	6.91	0	4.3	2.01	7.89	0	5.71	0.56	790	14.16	0	290	43.37	245.01	173.84	88.65	0	27.32	24.077	77.64	146620.6	0
p453	D2	10.8	5.29	0	0	0	13.66	2.57	0	1.68	7.04	1.5	4.66	27.46	4.17	0	6.98	0.88	1720	71.44	363	2620	171	151.36	172.75	69.36	0	5.98	9.029	62.86	140985.4	0
p453	D3	8.13	4.72	7.11	1.33	0.34	22.23	3.76	0	1.82	22.16	6.27	4.91	39.06	9.87	0.06	8.5	1.5	3570	102	654	5560	256	313.2	180.5	78.19	0	6.88	8.315	90.65	131860.6	0
p453	D4	9.94	5.37	15.07	1.5	0.67	20.52	3.99	0	1.92	8.83	5.29	4.3	22.99	5.05	1.12	6.4	1.35	2870	59.22	470	2960	222	346.52	150.34	128.32	0	6.88	8.556	67.76	24033.54	0
p453	D5	22.63	3.83	3.89	0	0.67	29.04	2.46	0	1.63	2.28	0	21.98	0	5.64	0.06	4.49	0.98	0	2.87	0	0	0	516.45	464.6	209.57	0	19.74	37.051	53.97	8227.162	1.93
p452	Admission	36.35	2.98	0	0.99	1.32	21.38	2.01	0	1.77	139	0.33	55.8	0	69	2.33	10.25	0.98	700	7.53	0	430	7.99	94.36	1051.97	353.89	0	16.06	39.007	105.58	201832.7	4.18
p452	Admission	53.8	3.15	8.09	1.33	0.67	14.52	1.78	6.28	1.77	224.71	1	93.26	0	91.4	0	9.7	0.93	90	0	0	310	0	190.58	1941.63	325.91	0	30.66	31.31	123.88	201637.4	7.03
p452	D1	60.05	4.22	22.2	0.84	0.84	29.24	1.48	0	1.35	202.89	2.03	60.74	0	51.66	0	5	1.49	0	0	0	290	0	1261.94	457.76	1117.24	5.88	75.11	23.8	27.4	200819.6	6.48
p452	D1	47.77	3.22	14.25	0	1.16	11.94	1.67	2.26	1.73	222.66	1.5	97.78	0	105.34	1.12	11.43	0.67	0	0	0	330	0	291.67	2526.69	374.31	0	34.32	26.978	155.55	196077.1	7.09
p452	D2	46.2	3.36	19.73	0.31	1.94	18.81	1.78	5.95	1.92	212.92	4.22	86.35	0	75.87	1.55	14.79	1.09	710	10.25	106.8	480	22.37	442.57	2200.02	432.55	0	28.93	24.689	155.91	200386.2	8.43
p452	D3	23.43	3.08	0	0.31	2.56	30.74	2.51	2.26	2.31	25.68	4.5	39.49	0	24.58	2.7	14.61	0.98	0	0	0	260	0	655.04	980.95	829.97	0	13.94	9.951	122.76	189418.2	5.66
p452	D4	34.62	2.91	8.09	0.31	2.4	23.94	2.35	3.11	2.36	18.22	3.64	27.3	0	12.17	3.76	14.17	0.67	0	0	0	270	0	587.68	1116.17	1234.3	0	13.94	10.177	125.73	122822.9	3.39
p452	D5	14.05	2.87	0	0	1.94	25.64	1.9	0	2.06	31.88	1.5	16.6	0	16.05	2.33	14.61	0.77	1290	17.32	394.48	830	42.74	597.42	599.11	2375.37	0	1.77	19.619	62.67	94759.03	2.91
p452	D6	24.48	3.15	0	0	2.56	25.64	2.35	0	2.16	17.54	3.01	11.88	0	12.02	3.41	11.79	1.71	800	17.32	184.18	620	30.4	666.23	469.06	1112.64	0	14.66	16.781	75.52	58113.61	5.91
p451	Admission	21.42	8.11	40.18	18.11	15.02	110.93	7.94	18.99	4.05	34.95	14.84	15.27	0	15.43	14.77	13.56	5.88	0	13	0	1700	6.5	133.55	290.83	121	0	17.76	11.283	24.75	361781.6	7.31
p451	Admission	9.41	2.94	0	0	0.34	17.1	1.78	0	1.75	46.04	0	16.12	0	5.35	2.88	8.31	1.35	0	5.7	0	900	0	213.45	427.77	182.33	0	8.99	11.93	6.1	293427.9	2.29
p451	D1	11.14	2.77	0	2.99	0.67	11.94	1.56	0	1.92	44.71	0	17.59	0	5.94	3.06	9.79	1.82	0	0	0	0	0	219.83	492.23	212.89	0	6.65	12.143	7.09	287601.8	2.46
p451	D1	13.73	3.05	0	0	0.34	15.38	1.67	0	2.31	62.85	0	20.03	0	13.56	3.06	8.87	2.66	0	1.6	0	100	0	305.65	584.03	211.23	0	10.19	15.017	6.6	188461.5	2.5
p451	D2	13.58	4.01	0	3.98	0.67	132.4	2.35	0	2.74	802.9	0	119.64	0	60.83	2.52	10.16	5.08	0	2.2	0	400	0	2511.91	1230.17	627.01	0	30.09	35.763	41.07	262393.4	15.86
p451	D3	7.95	3.87	0	8.85	1.32	56.01	2.8	0	2.71	262.18	3.01	55.63	0	12.33	3.76	10.07	6.83	0	5.7	0	1100	0	2003.84	880.47	544.41	0	16.4	51.251	39.84	197185.1	7.4
p451	D4	21.56	3.58	0	12.38	0.84	23.94	2.35	0	2.66	67.72	0	74.79	0	7.74	3.76	10.16	7.94	0	0	0	0	0	1685.84	1044.07	880.76	0	23.38	97.746	36.13	125631.9	7.49
p451	D5	31.44	3.94	0	11.74	1.94	30.74	2.8	0	2.01	40	3.01	63.55	0	10.33	3.41	9.43	11.08	0	3.5	0	1200	3.6	1133.87	1160.25	859.87	0	18.75	112.376	36.13	72463.66	6.6
p451	D6	45.03	4.16	3.89	8.37	2.86	44.25	3.08	0	2.33	57.12	4.77	60.52	0	13.41	5.07	12.5	12.25	0	1.1	0	400	0	1054.13	1553.03	1610.9	0	26.57	295.722	36.54	85897.37	10.53
p451	D7	66	3.33	0	7.88	0	39.2	2.18	0	2.26	108.18	0	86.26	0	10.79	0.65	13.03	6.78	0	0	0	0	0	1505.41	2013.47	2186.38	0	23.69	292.097	22.81	52492.91	8.79
p449	Admission	227.47	2.67	0	3.65	1	29.04	2.4	0	1.82	14.78	3.01	18.31	0	12.02	0	7.74	1.09	0	45.35	483	3030	24.8	253.51	690.87	1199.54	0	19.41	47.718	30.7	5891.7	2.71
p449	Admission	47.14	6.79	24.1	19.06	8.42	72.73	7.02	27.27	3.39	9.59	15.47	11.88	0	10.17	9.76	9.79	3.92	500	51.31	503	4240	52.15	128.18	147.35	468.77	0	23.69	15.611	47.16	35645	7.4
p449	Admission	45.03	6.71	31.6	20.65	12.09	79.4	8.29	20.58	3.53	27.71	16.08	11.4	0	11.86	9.76	10.43	3.4	520	79.67	922	7160	93.26	112.55	146.48	277.91	0	13.22	13.397	39.84	31632	7.4
p449	D1	25.25	3.58	0	3.98	3.61	25.64	4.16	0	2.11	6.97	5.29	5.17	0	5.64	2.52	9.24	1.82	0	46.01	464	4240	29.74	91.69	118.72	250.3	0	1.43	11.93	24.32	29940.2	0.8
p449	D2	93.7	2.87	0	2	0.51	15.38	2.68	0	2.11	9.65	1.5	9.62	0	4.9	0.06	8.12	1.4	0	0	0	0	0	186.68	378.67	632.31	0	20.38	11.283	29.44	10710.6	1.97
p449	D3	63.36	2.87	0	0.99	0	23.09	2.57	0	2.18	5.73	0.33	6.04	0	4.76	0	7.55	1.4	0	0	0	0	0	245.01	254.87	421.38	0	10.19	17.358	24.32	9887.2	2.39
p449	D4	41.59	2.8	0	0.13	1.63	18.81	2.57	0	2.66	5	1.92	5.53	0	4.46	0.06	7.27	1.19	0	0	0	0	0	232.28	239.41	532.19	0	10.58	17.929	28.17	8750.3	2.15
p449	D5	35.89	3.22	0	2.99	0.67	26.49	2.57	0	3.5	3.53	2.31	6.66	0	6.24	0	6.69	0.98	0	0	0	0	0	349.36	254.03	463.59	0	10.97	14.416	40.66	7793.9	3.32
p448	Admission	105.18	2.84	1.22	14.62	16.53	140.64	2.68	0	4.5	63.83	23.32	107.26	20.7	33.9	17.02	19.33	7.1	550	34.6	800	4900	95.8	98.93	796.05	232.81	0	15.01	11.842	28.59	35207.89	0.47
p448	Admission	78.21	2.77	12.16	9.17	10.97	109.27	1.84	0	3.5	20.14	15.78	14.63	8.5	26.02	12.19	18.74	3.61	0	8	0	1100	2.1	92.05	533.66	167.54	0	18.92	4.98	30.7	22408.1	0
p448	D1	90.34	3.36	0	47.07	17.63	153.81	2.57	0	3.77	26.08	11.85	18.96	5.7	37.3	16.28	20.09	17.1	0	0	0	0	0	172.51	210.24	110.46	0	26.27	2.9	51.77	16786.73	0.11
p447	Admission	4.5	3.12	28.28	8.18	4.88	47.03	1.83	4.5	1.76	44.03	5.6	22.14	49.4	29.06	3.25	11.83	2.17	1420	101	1430	11600	147	71.76	305.31	233.49	0	14.29	10.878	41.16	376436.6	3.51
p447	D1	7.96	1.8	37.51	1.65	3.54	38.19	1.25	1.37	1.47	48.39	2.69	29.18	0	49.82	1.4	10.69	0.66	150	19	0	3000	22.8	103.36	276.69	287.56	0	13.14	9.94	49.75	332638.7	2.14
p447	D1	4.29	1.97	43.68	0.51	3.35	24.68	1.32	1.79	1.42	47.69	1.4	22.77	0	28.19	0.73	11.33	0.66	0	13	0	2400	25.2	131.93	385.81	276.17	0	20.88	9.471	37.36	221736.6	3.38
p447	D1	9.24	1.89	41.78	3.02	3.82	36.41	1.39	1.79	1.54	40.61	2.81	20.12	2.6	27.33	2.29	11.91	0.94	200	20.7	148	3800	37.2	162.56	426.41	274.54	0	20.03	9.158	22.51	270851.1	4.12
p447	D2	15.96	2.24	34.26	2.72	2.97	43.51	1.44	3.11	1.51	11.88	2.21	8.58	0	15.43	2.57	9	1.04	160	18.1	0	3000	26.4	122.8	333.31	188.5	0	14.61	6.335	40.53	133055	2.67
p447	D3	21.8	3.21	26.48	7.06	3.82	54.02	1.83	4.98	1.69	8.99	4.43	11.18	3.9	13.62	2.84	10.41	2.08	180	14.7	0	3800	31.6	167	305.47	164.83	0	15.4	11.502	27.3	193566.1	3.21
p447	D4	37.4	1.97	39.66	0.51	2.78	66.14	1.39	1.37	1.34	4.61	2.45	10.03	13.3	8.26	1.08	8.86	1.04	550	41.9	635	6500	78.5	185.22	316.02	259.82	0	5.78	3.491	11.35	126443.8	3.13
p447	D5	54.48	2.68	39.23	3.47	4.5	75.57	1.68	4.27	1.56	12.45	3.38	10.31	16.3	14.45	2.29	9.7	1.74	800	55.1	922	6800	104	141.68	316.96	251.62	0	7.9	1.893	22.07	113486.9	2.46
p447	D6	40.68	2.5	35.57	4.2	3.73	83.25	1.63	3.11	1.6	7.9	2.93	8.52	11.2	11.54	2.29	9.21	1.79	600	32.4	628	6300	83.6	96.86	160.34	203.58	0	0	2.214	28.59	90969.52	1.48
p447	D7	193.4	2.41	25.79	2.11	3.63	90.03	1.49	2.66	1.37	8.85	3.92	21.13	2.6	11.82	2.84	9.56	1.79	210	19.4	109	3700	33.3	169.28	478.96	243.39	0	4.86	2.374	14.52	86368.33	2.88
p446	Admission	48.18	3.84	30.08	6.78	5.65	52.28	2.39	7.29	1.94	5.62	5.79	7.6	0	9.9	4.66	5.44	2.6	0	27.78	240	2200	21.4	248.88	224.76	529.51	0	8.3	16.25	18.55	26263.2	3.74
p446	D1	26.79	5.49	29.18	6.21	6.14	64.42	2.7	4.62	1.98	11.11	3.81	5.7	0	13.48	4.41	6.11	3.62	510	71.98	717	7040	79.1	254.42	199.29	437.56	0	0	9.784	16.77	36963.2	3.07
p446	D1	20.53	3.12	33.61	1.8	3.35	27.41	1.88	3.23	1.54	4.32	2.21	4.45	0	8.26	0.91	4.77	2.03	530	79.34	1129	8240	104	237.71	165.25	580.36	0	0	3.967	9.04	28710.7	2.25
p446	D1	44.93	2.15	45.98	0.15	2.4	32.83	1.44	3.8	1.41	2.64	4.23	12.59	0	17.81	0.35	4.31	0.85	0	0	176	0	22.95	240.76	184.1	1341.31	0	15.08	7.905	11.8	26312.7	2.71
p446	D2	18.33	3.03	21.4	4.49	4.3	41.74	1.98	4.5	1.62	4.38	4.83	5.72	0	7.72	2.57	4.64	1.6	0	0	0	0	0	97.85	95.82	303.77	0	0	4.916	13.17	191361.6	2.35
p446	D3	21.63	1.97	27.83	0	2.59	14.45	1.29	1.58	1.27	12.8	0.59	3.36	0	5.84	0	4.64	1.32	0	0	0	0	0	197.07	236.01	345.62	0	0	16.021	3.79	41330.9	1.2
p446	D4	9.86	2.15	10.55	0.68	2.4	26.5	1.54	2.22	1.27	7.96	0.77	2.8	0	7.99	0	4.64	1.08	0	21.82	0	1900	0	131.35	111.39	509.37	0	0	9.94	18.77	27313.6	0.93
p445	Admission	39.95	6.23	35.13	20.03	45.28	113.61	3.4	8.67	2.88	22.35	8.57	18.02	0	19.5	9.98	10.9	6.97	510	95.05	919	9690	81.15	122.5	173.23	171.62	0	11.26	7.65	24.91	24684.5	4.06
p445	D1	49.85	3.3	28.73	10.93	36.22	47.03	1.54	1.16	1.47	41.06	3.38	27.43	0	15.84	2.84	8.3	7.17	0	0	0	0	0	156.57	241.75	191.86	0	5.78	9.628	14.07	22118.6	1.91
p445	D1	37.03	3.84	26.48	7.2	20.71	64.42	1.63	1.16	1.56	26.08	6.43	12.66	0	17.39	3.38	10.69	6.03	0	23.68	0	2480	0	120.7	128.41	140.88	0	3.86	16.176	9.04	21052.4	0.42
p445	D1	40.18	3.75	22.8	11.48	26.32	55.76	1.41	0.38	1.54	19.09	5.97	10.49	0	17.1	3.25	9.28	5.98	0	0	0	0	0	283.69	191.36	274.54	0	0	13.062	9.97	13265.8	1.98
p444	Admission	41.68	2.41	4.43	95.33	2.68	27.41	1.88	3.34	1.83	285.18	0.59	52.39	52.59	20.91	2.29	9.56	19.74	2310	126	1809	6520	298	85.36	449.98	358.41	23.93	20.03	10.409	38.84	85456.69	1.37
p444	Admission	35.56	1.62	0	78.29	1.38	16.34	1.34	0.76	1.87	296.9	0	68.48	0	37.47	1.4	12.55	19.23	0	7.96	0	0	19.58	151.2	810.57	535.69	0	25.75	11.814	21.2	128875	1.39
p444	Admission	27.31	2.15	4.43	67.75	2.12	27.41	1.78	1.58	2.56	300.32	1.82	52.11	8.26	20.91	3.11	11.33	21.29	740	42.98	646	1580	100	171.01	631.58	555.75	0	21.71	9.158	41.79	178560.6	2.06
p444	D1	22.36	2.06	5.9	114.6	2.21	18.21	1.54	1.89	1.98	113.28	1.68	40.21	5.74	14.59	2.57	11.12	34.96	790	39.27	741	1560	110	209.99	367.16	336	23.46	19.74	10.565	50.59	160672.9	1.25
p444	D2	27.42	3.08	8.95	110.85	2.59	33.73	2.06	2	2.77	60.72	1.95	32.71	12.54	10.58	2.98	8.44	43.48	1170	62.85	1034	2510	169	230.61	295.6	592.64	64.62	41.91	7.749	48.5	44370.89	2.44
p444	D3	12.21	3.84	21.16	44.39	6.72	88.34	3.5	8.42	4.28	31.75	5.6	16.99	40.58	10.17	6.58	9.7	15.03	1880	104	1517	5690	227	302.2	261.47	356.81	0	13.14	4.916	96.32	19018.63	3.74
p444	D4	25.18	2.86	16.85	65.49	2.97	84.94	2.03	3.57	6.86	656.28	3.16	84.51	8.78	77.73	3.64	14.58	21.39	880	36.78	704	1630	117	455.07	678.94	911.61	0	20.88	5.39	98.33	81059.85	2.94
p444	D5	6.94	3.08	23.5	33.28	2.78	41.74	2.68	5.1	4.77	52.78	2.69	21.2	5.98	11.13	2.29	9	9.67	750	27.16	537	1180	93.48	287.69	230.65	404.47	0	0	2.534	121.12	78468.23	2.46
p444	D6	27.78	4.84	27.61	17.39	6.92	88.34	3.42	7.17	7.89	14.56	4.63	11.49	60.8	19.07	6.82	9.7	10.07	2950	160	2307	7370	377	286.51	291.17	448.55	0	13.96	2.374	87.45	35512.65	3.76
p444	D7	10.17	2.06	9.49	19.77	3.54	38.19	1.91	5.94	5.16	9.79	2.45	10.03	0	13.75	2.84	5.7	6.52	420	10.39	266	300	48.58	318.91	176.02	350.42	0	0.58	4.125	53.91	19507.04	2.63
p443	Admission	63.8	3.48	226	37.76	4.21	49.66	3.02	4.98	1.87	312.13	3.6	31	43.2	27.04	14.97	8.86	31.77	5820	279	2992	15280	500	138.55	481.11	193.54	0	30.92	15.087	30.74	45435.8	2.92
p443	Admission	64.19	4.94	322.7	50.23	4.3	54.02	3.29	4.03	2.5	377.26	2.21	49.32	38.11	24.18	16.82	10.34	36.17	8180	272	3676	15130	614	401.19	770	367.98	6.77	44.52	17.12	37.99	44673.37	4.77
p443	Admission	10.69	2.15	27.16	6.78	2.78	36.41	1.93	2.44	1.63	154.54	0.77	11.64	24.78	379.9	2	9.98	3.62	960	88.15	845	3870	180	230.98	250.97	211.93	0	1.44	10.722	21.85	58242.64	1.93
p443	D1	0	1.89	81.11	16.85	1.84	12.54	1.49	1.37	1.44	45.19	0	11.82	10.68	27.33	2.29	7.06	10.67	2140	116	1910	5390	317	156.16	139.05	151.18	0	3.32	13.685	22.07	165012.8	1.25
p443	D2	8.12	2.68	70.51	14.05	2.59	33.73	2.24	3.11	1.44	207.17	0.59	33.95	42.18	51.6	3.38	7.54	8.66	2220	171	1848	8440	309	114.32	293.86	4.19	0	19.45	30.888	37.57	180964	2.33
p443	D5	21.28	5.3	142.88	26.04	6.24	85.79	3.29	7.17	2.24	42.67	4.63	18.68	36.07	17.67	9.76	8.72	21.39	3340	194	2758	10820	423	301.73	339.1	268	0	12.64	4.916	46.83	57634.69	3.64
p443	D6	14.26	2.99	108.85	13.37	2.03	55.76	2.03	1.47	1.3	28.9	0	13.04	7.48	10.72	6.35	5.97	19.08	1190	59.71	791	2940	191	261.38	183.66	287.56	0	4.86	3.331	24.26	46841.07	2.79
p443	D7	29.64	4.57	150.35	19.24	4.5	83.25	2.7	4.5	1.76	31.99	3.81	16.36	15.43	16.82	9.98	8.16	18.1	2690	126	1785	6770	323	311.03	272.21	396.56	0	20.17	3.967	44.32	41185.13	3.64
p442	Admission	17.03	2.46	0	27.34	3.06	30.13	2.89	0.76	1.25	44.26	2.69	24.8	0	33.68	42.68	6.38	15.44	0	0	0	0	0	46.09	309.24	279.43	0	83	6.649	29.02	161710.9	1.5
p442	Admission	31.61	2.15	0	33.28	6.24	38.19	3.1	0.96	1.24	73.59	1.82	30.57	0	18.51	63.54	6.11	15.85	0	0	0	0	0	70.43	400.8	344.02	0	94.5	6.335	28.16	167535.2	1.66
p442	D1	39.4	2.77	0	35.84	4.69	34.62	2.26	1.79	1.27	67.55	1.95	28.4	0	25.9	33.32	7.4	14.57	0	0	0	0	0	69.66	341.08	521.77	0	8.3	6.178	38.2	118602.8	1.6
p442	D2	0	2.24	0	22.65	3.73	31.93	2.34	1.79	1.34	243.05	2.21	23.06	0	23.75	27.78	8.65	8.11	0	0	0	0	0	128.57	228.68	1102.59	0	0	14.464	57.64	86759	1.2
p442	D3	27.37	2.46	0	21.61	4.78	66.14	2.19	4.15	1.54	113.57	7.73	33.17	0	29.63	9.54	10.27	4.74	0	0	0	0	0	111.24	456.35	969.21	0	24.3	12.126	92.29	49687	4.1
p442	D5	17.72	2.24	1.89	15.25	3.73	60.96	1.76	2	1.62	118.28	4.43	21.03	0	21.48	6.82	9.14	4.99	0	0	0	0	0	162.42	273.65	243.39	0	0	8.845	90.27	49721.1	2.44
p442	D6	22.19	2.9	5.61	21.87	6.04	73.01	1.98	4.5	1.76	143.9	4.53	24.24	0	30.07	10.85	10.83	5.58	0	0	0	0	0	185.48	260.23	394.98	0	0	4.916	111.35	34684.5	2.42
p442	D7	29.59	3.03	6.19	18.97	5.07	91.73	3.05	3.11	1.78	613.83	6.24	69.85	0	63.84	11.39	13.13	5.68	0	0	0	0	0	457.09	374.76	421.83	0	11.61	1.893	140.97	44990.5	2.77
p441	Admission	40.13	3.3	13.13	10.25	5.75	36.41	2.29	10.07	1.94	4.38	5.22	4.64	0	8.26	2	7.96	3.52	0	66.63	373	4910	21.66	177.1	213.49	374.34	0	5.33	8.532	39.68	21795	2.82
p441	Admission	13.59	2.99	3.2	16.59	5.27	39.97	2.03	12.54	1.96	5.16	3.27	6.56	0	8.53	1.4	7.89	3.67	0	30.98	0	2770	0	372.92	187.49	190.18	0	0	11.814	46.83	26676.2	3.51
p441	D1	11.71	1.8	0	7.62	2.31	18.21	1.49	6.8	1.32	8.96	0.38	4.49	0	6.38	0	6.11	1.98	0	0	0	1120	0	179.73	138.96	215.25	0	0	37.967	42.85	534.4	1.33
p441	D1	20.7	1.28	0	2.11	1.28	183.24	1.13	6.43	1.27	7.68	0.14	67.96	0	5.84	0	6.24	1.13	0	0	0	0	0	383.8	303.19	1036.81	0	8.69	13.47	53.91	16667.4	3.11
p441	D2	6.41	1.76	0	5.22	2.21	16.34	1.29	4.98	1.34	9.57	0	4.36	0	4.52	0	6.52	1.32	0	0	0	0	0	209.49	156.99	220.24	0	0	19.287	65.26	21633.3	1.12
p440	Admission	19.65	5.67	18.07	10.66	8.09	86.64	3.18	7.42	2.67	6.89	6.52	4.68	38.67	14.73	7.74	7.61	3.82	1450	198	1276	17250	171	128.13	153.18	624.76	0	6.23	9.628	33.74	34257	3.07
p440	D1	16.78	2.33	10.02	1.17	3.54	23.76	1.49	3.23	1.53	9.18	2.21	4.82	0	8.53	2.43	5.57	1.32	0	26.51	0	1850	0	107.96	142.28	557.3	0	0	6.02	43.69	34735.9	0.91
p440	D1	22.08	0	11.07	2.27	2.12	42.62	1.63	4.74	1.91	8.49	3.81	4.26	0	11.82	1.7	5.84	2.03	500	61.96	657	5910	37.87	127.84	147.57	326.36	0	0	11.346	26.86	25028.8	0.62
p440	D1	53.37	5.12	13.63	6.78	5.75	84.94	2.78	4.98	2.48	7.01	4.53	5.26	0	14.31	4.66	7.2	3.62	650	149	1479	16580	219	175.52	314.49	992.76	0	6.87	7.278	27.08	21342.7	3.47
p440	D2	0	1.71	3.83	0	1.84	18.21	1.13	1.47	1.41	29.96	0	4.03	0	14.59	0	4.24	0.48	0	27.78	0	2640	0	121.45	110.17	352.02	0	0	4.916	30.31	26643.1	0.87
p440	D3	0	2.15	7.31	1.33	2.4	20.07	1.46	2.11	1.8	8.71	0.38	4.24	0	8.81	0	4.11	0.94	0	25.88	0	2690	0	183.26	142.65	382.29	0	0	15.554	35.44	47070.1	1.29
p440	D4	149.87	1.8	45.15	0	3.35	41.74	1.2	3.57	3.37	2.13	2.93	25.76	0	7.18	0	4.57	0.57	0	27.78	0	1140	0	185.48	874.25	973.63	0	14.61	51	50.38	35198.6	3.13
p440	D5	6.41	2.11	17.34	0	1.65	27.41	1.34	1.79	2.67	3.27	0.94	5.52	0	5.58	0	4.11	0.71	500	69.64	324	6690	41.8	201.66	183.8	811.95	0	0	13.529	27.3	33924.1	2.08
p440	D6	28.46	1.84	28.73	0	2.78	71.3	1.2	3.57	3.08	5.22	1.95	39.78	0	7.32	0	5.04	0.57	0	0	0	0	0	300.56	376.88	2179.96	0	27.8	18.247	88.26	32283.4	4.67
p439	Admission	0	4.21	19.5	8.18	7.6	86.64	2.39	7.67	1.91	64.39	4.83	7.85	86.06	36.88	8.19	7.4	4.55	3710	206	2451	11400	391	76.06	173.17	181.76	0	5.33	20.065	34.59	75877.52	2.67
p439	Admission	8.45	5.4	25.34	5.36	8.78	90.03	2.65	6.67	2.32	64.54	4.23	11.14	38.85	187.72	9.09	9.56	5.93	2140	127	1613	5690	283	73.83	289.43	383.88	0	20.31	8.062	28.59	205893.5	4.31
p439	Admission	2.2	5.72	28.28	7.9	8.88	92.57	3.34	6.67	2.67	44.26	5.79	10.44	62.2	27.33	7.28	8.58	6.57	4360	188	2747	10720	477	63.37	159.06	287.56	0	15.87	3.491	33.53	167804.4	3.68
p439	D1	14.6	2.77	23.26	0.15	4.5	47.91	1.49	2.44	1.51	91.43	2.08	11.91	24.43	20.06	3.38	7.75	2.46	1150	79.84	1053	3690	174	59.26	256.72	323.14	0	4.62	22.138	19.43	204505	2
p439	D2	9.16	3.48	21.4	5.93	6.43	61.83	2.06	4.74	1.81	7.9	3.6	4.22	36.55	14.87	6.35	7.61	3.23	1660	96.89	1436	5140	229	217.38	96.98	451.68	0	4.37	2.694	37.57	82427.9	2.29
p439	D3	8.45	2.81	13.63	2.42	4.69	39.97	1.39	3.57	1.47	224.99	2.45	5.66	0	14.45	2.29	6.99	2.65	530	21.26	364	990	82.26	302.78	172.3	963.32	0	5.33	4.6	25.56	32668.9	2.17
p437	Admission	4.4	3.03	32.73	3.62	3.92	39.97	1.76	3.23	2.59	11.93	3.16	6.06	15.4	8.4	2.57	4.64	3.33	520	63.7	336	5800	30.38	197.97	196.1	272.9	0	7.49	7.905	30.1	37646.6	1.79
p437	Admission	6.67	2.33	35.79	4.78	2.97	23.76	1.86	2.66	2.71	16.3	0	6.58	19.19	7.86	0.73	4.11	5.48	1460	112	1347	11640	136	211.63	233.8	272.9	0	16.48	9.002	22.07	33296.8	2.35
p437	D2	0	1.71	32.73	0.15	2.21	31.03	1.29	1.58	2.05	21.44	0	5.88	0	9.08	0	4.11	3.38	0	7.93	0	290	0	308.25	197.11	348.82	0	3.32	8.532	20.98	15831.1	1.52
p437	D4	0	1.54	16.12	1.65	1.84	55.76	1.34	1.26	2.56	57.64	0	9.3	0	8.81	0	4.51	3.91	0	6.08	0	560	0	208.23	196.59	360	0	6.23	4.125	23.82	42834.1	2.04
p437	D5	85.43	1.8	86.99	1.8	2.21	25.59	1.39	1.58	1.89	31.88	0	19.65	0	10.03	0	5.3	3.96	0	9.86	0	430	0	185.48	602.81	527.96	0	15.24	23.74	48.5	38691.1	3.7
p437	D6	19.59	1.36	69.93	3.47	2.03	44.39	1.25	1.16	2.09	18.24	2.69	26.06	0	7.99	0	5.24	5.68	0	0	0	0	0	476.54	417.14	4282.98	0	20.74	16.657	60.74	30474.5	4.33
p437	D7	8.45	1.84	39.44	2.72	3.73	27.41	1.41	2.89	1.65	13.29	0	6.5	0	9.76	0	5.17	2.08	0	1.31	0	0	0	327.22	169.16	607.95	0	0	3.331	57.02	27697.6	1.45
p436	Admission	38.26	0.22	25.44	5.56	0.26	25.53	0	0	0	56.57	0.01	21.27	0	6.93	0.77	5.29	0	0	7.2	0	1800	6.5	253.61	352.36	672.23	0	8.4	34.5	9.67	119578.2	1.81
p436	Admission	48.7	0	23.61	6.26	1.2	15.47	0	0	0.12	58.39	1.19	67.24	0	7.23	0.06	5.16	0	0	0	0	0	0	306.35	456.21	1001.28	0	13.63	32.2	10.3	76430.6	3.14
p436	D1	23.49	0.22	28.65	6.26	1.62	7.29	0.17	0	0	10.99	0.52	7.96	0	11.84	0.54	5.98	0	0	0	0	0	0	402.96	313.16	1285.52	0	0	81.6	12.15	48972.42	2.7
p436	D2	27.69	0	27.24	2.33	0.26	25.53	0	0	1.2	14.44	0	6.76	0	10.27	0.54	5.84	0	0	0	0	0	0	549.54	352.87	928.81	0	0	42.7	12.77	26895.93	4.36

p433	Admission	153.49	177.59	174.08	34.4	121.26	806.49	40.9	244.84	7.24	58.6	2.13	22.82	10.99	40.09	5.5	116.57	6.33	670	33.75	533	4790	57.04	430.75	370.54	1039.52	0	183.17	71.4	797.22	394898	43.48
p433	Admission	117.79	144.22	141.16	26.72	135.01	720.43	59.03	207.16	4.03	55.65	3.43	22.94	11.54	53.8	5.5	112.88	5.95	680	28.23	450	5140	49.48	394.17	458.74	737.96	0	146.93	65.1	713.39	300335	32.56
p433	D1	113.87	90.19	136.73	19.99	90.59	520.62	41.54	202.92	3.53	50.11	3.92	24.69	4.29	32.63	4.09	56.27	4.75	620	16.53	421	6030	47.37	390.14	591.64	1986.24	0	146.14	67.6	664.21	250337.2	26.16
p433	D1	103.51	57.5	99.81	26.72	82.27	289.38	30.07	114.56	2.27	32.57	1.61	19.84	0	28.44	3.66	29.43	3.89	0	6.69	0	930	0	362.12	586.55	1651.79	0	102.65	50.3	454.58	78105.5	23.24
p432	Admission	47.65	11.64	60.8	80.19	20.52	207.45	5.72	30.75	7.47	31.5	31.67	21.7	7.4	25.35	19.76	8.36	14.71	230	22.4	0	3500	35.6	145.27	258.62	145.32	46.1	39.95	17.6	100.07	109098.9	16.77
p432	D1	39.8	2.28	29.35	61.49	10.39	35.05	2.51	0	1	135.84	27.45	34.75	0	31.75	3	6.41	3.58	0	0	0	400	0	196.59	312.32	183.46	47.58	42.84	18	85.04	79237.91	8.08
p432	D1	44.8	1.93	38.05	56.56	3.53	44.22	1.07	0	1.3	157.4	30.38	32.99	0	30	5.18	6.88	5.29	0	0	0	0	0	218.59	312.32	494.02	31.12	39.58	13.7	92.7	77371.1	7.73
p432	D1	39.55	1.75	32.1	44.02	3.16	23.07	1.07	0	1.2	76.67	20.84	22.79	0	17.3	3	6.48	3.58	0	0	0	0	0	164	315.74	753.03	0	31.55	14.7	113.48	87666.51	4.31
p432	D6	24.77	2.28	27.6	87.39	7.25	68.31	0.82	8.98	2.17	42.55	36.49	26.55	0	16.32	5.83	6.16	15.37	0	0	0	0	0	186.43	153.84	599.07	18.28	44.82	22.4	154.63	39717.87	13.18
p429	Admission	35.24	4.55	33.45	45.18	8.53	70.44	3.66	9.5	2.74	22.78	5.64	18.85	0	14.7	3.44	6.44	5.29	0	0	0	0	0	390.91	418.61	876.89	0	39.58	18.8	161.37	346531.8	4.95
p429	D1	45.92	1.02	22.11	26.05	0	20.59	4.11	0	0.24	22.81	5.73	19.87	0	12.47	0.65	6.19	0	0	9.86	0	990	0	819.27	450.91	876.89	0	39.21	31	146.13	232106.4	2.01
p429	D1	40.3	1.93	16.61	17.96	0	16.77	3.32	0	0.18	25.32	2.84	23.91	0	13.58	0.3	5.77	0	0	3.76	0	770	0	742.01	426.64	1141.81	0	42.72	32.2	125.07	203422.6	1.91
p429	D1	28.6	0.93	23.24	28.4	0	15.47	2.63	0	0.24	29.24	5.64	23.38	0	17.47	0.3	5.84	0	0	3.22	0	930	0	608.87	416.57	2120.53	0	37.83	21.4	188.56	113538.1	1.5
p429	D2	24.77	0.64	22.87	23.36	1.2	20.59	2.86	0	0.12	13.86	3.82	14.58	0	10.9	0	6.26	0	0	6.08	0	850	0	545.21	354.69	692.93	0	36.16	29.5	157.12	132665.5	1.5
p429	D3	40.55	3.64	28.3	26.72	2.41	46.47	3.43	0	0.69	26.01	5.16	14.64	0	11.52	0.77	6.99	0	0	9.86	0	1430	0	463.69	317.6	642.42	0	46.07	26.1	178.59	91543.1	1.86
p429	D4	73.78	3.22	22.87	16.26	2.98	43.09	1.56	0	1.1	28.67	3.43	27.24	4.87	11.84	2.56	6.26	1.91	770	54.94	508	5860	75.83	266.69	409.39	1412.53	0	34.04	17.8	172.1	121146	1.6
p429	D5	51.14	1.84	24.72	27.39	0.26	29.14	4.33	0	0.36	25.06	6.29	21.42	0	9.19	0.99	6.44	0.59	0	4.89	0	400	0	252.14	336.03	504.12	0	45.84	10.8	206.51	77575.6	0.26
p429	D6	18.8	1.57	31.08	12.16	0.98	32.7	0.63	0	0.24	54.54	3.63	12.58	0	11.36	0.3	6.26	0	0	0	0	0	0	497.48	283.62	571.81	0	30.69	15.7	180.58	58393.6	0.05
p429	D7	43.66	2.71	131.81	62.15	0	44.22	5.11	0	0.12	29.99	21.09	18.29	0	8.89	0.3	6.16	0	0	9.21	0	210	0	407.4	294.88	1685.99	0	44.93	17.6	444.47	17893	1.96
p427	Admission	62.05	9.28	47.1	35.06	17.72	146.92	3.66	23.71	5.89	138.89	11.52	70.28	32.64	89.24	10.53	6.73	13.35	2300	152	1163	5140	257	221.52	823.91	599.07	0	100.66	63.1	197.04	249696.3	20.88
p427	Admission	61.88	0	56.83	4.14	3.16	145.95	0	0	0.3	172.93	2.03	79.1	0	35.45	2.12	7.4	1.09	0	8.39	0	0	0	282.33	991.67	437.08	0	66.23	33.6	220.48	248601.1	8.12
p427	Admission	42.02	0	40.92	1.96	1.62	31.52	0	0	0	129.84	2.34	50.98	0	39.01	1.89	6.77	0	0	23.05	0	0	13.89	226.36	876.94	439.3	0	40.44	32.1	158.12	248127.7	5.59
p427	D1	38.78	0.33	45.89	5.56	1.82	53.14	0	0	0.24	143.95	3.34	42.36	0	52.5	1.67	8.28	0	720	45.51	572	1600	94.93	310.08	989.13	245.53	0	58.27	52.1	206.01	238596	9.9
p427	D2	44.35	0.64	54.52	3.24	2.02	44.22	0	0	0.36	88.22	1.4	34.37	0	22.8	1.89	6.84	0.59	470	62.89	517	2020	58.4	281.28	921.85	159.06	0	33.09	36.3	164.12	193155.8	5.59
p427	D3	41.29	0.74	48.61	3.42	2.79	61.87	0	0	1.55	30.31	1.82	22.51	0	14.38	2	7.36	1.91	320	53.52	572	550	38.1	432.25	602.68	3265.52	0	28.02	57.1	182.08	126475.9	7.17
p427	D4	76.22	1.93	44.35	6.96	3.53	85.11	0	0	2.92	19.11	3.82	23.5	14.8	11.52	2.89	6.05	2.95	1170	111	1094	4010	155	306.52	712.96	424.86	0	29.82	28.4	104.38	63795.16	4.13
p427	D5	52.13	6.58	52.18	15.92	10.69	146.92	1.86	11.5	5.66	37.26	9.39	23.57	30.76	18.79	7.12	6.99	7.56	2490	166	1308	6730	252	722.77	619.3	773.94	0	42.6	31.9	125.07	58297.96	12.82
p427	D7	39.42	4.14	47.71	10.44	5.61	129.38	1.14	0.88	2.64	20.04	8.49	25.68	4.82	17.3	4.09	6.48	5.43	800	68.03	617	1490	101	648.28	576.12	939.47	0	52.3	33.8	148.63	67525.9	16.45
p426	Admission	52.9	2.1	259.54	8.01	4.24	41.95	0.44	8.17	1.45	99.11	0	28.8	0	17.96	3	5.77	5.02	0	2.71	0	0	0	310.93	505.61	804.23	0	29.82	39.8	94.23	22175.8	8.04
p426	Admission	20.15	1.93	238.49	1.78	2.02	41.95	0	0	0.69	74.18	0	27.74	0	15.35	1.45	5.63	1.52	0	9.21	0	670	0	450.13	419.85	374.02	0	23.83	28.6	70.63	28644.1	4.41
p426	Admission	41.05	1.21	281.95	1.78	2.02	37.37	0	0	0.95	53.19	0.01	32.21	0	11.84	1.45	5.77	1.91	0	11.82	0	720	0	354.75	515.74	468.28	0	14.88	38.3	72.18	24943.6	4.59
p426	D1	25.59	2.54	285.58	5.91	5.61	50.93	0.02	0.88	2.13	79.68	1.3	25.06	0	22.29	3.22	6.05	3.58	340	35.48	130	3420	3.92	354.1	383.19	502.99	0	23.13	26.9	84.79	23691.4	5.41
p426	D3	37.19	11.8	276.49	30.4	19.13	127.42	4.61	26.5	4.76	5.02	7.94	10.94	0	15.67	12.11	7.25	9.17	0	4.32	0	0	0	130.34	211.68	150.6	0	32.67	16.8	144.12	52250.6	12.66
p425	Admission	114.08	6.09	55.68	27.22	10.99	133.3	3.14	14.33	2.92	38.51	7.21	23.75	204	19.95	5.83	6.26	7.44	44470	74.8	3967	43170	1524	339.84	245.89	1979.75	0	44.7	29.5	87.09	27838.21	11.68
p425	Admission	140.4	9.91	57.4	40.71	18.28	141.1	4.89	28.45	4.49	23	9.65	22.57	153	20.28	8.83	7.51	8.72	40990	137	5958	70150	1362	392.62	257.97	2667.43	0	46.64	28.9	90.92	28075.92	16.61
p425	Admission	74.87	2.28	37.4	8.36	5.61	50.93	1.01	3.21	1.2	69.43	2.64	21.86	214	35.63	1.89	7.06	1.09	50920	71.09	5276	57170	1510	324.01	219.16	2269.31	0	30.98	50.1	98.04	33926.4	4.95
p425	D1	64.52	1.39	44.66	5.91	4.06	59.71	0.17	0	0.48	31.25	3.63	27.55	257	59.59	0.77	7.14	0	73560	48.52	5273	59290	1913	463.69	320.05	3146.5	0	40.93	39.4	122.8	71482.11	9.06
p425	D1	113.33	2.1	32.77	7.31	5.27	75.72	0.44	0	1.98	27.72	2.34	15.57	118	14.7	2.12	6.73	2.27	39530	77.27	4267	45370	1307	328.65	249.44	902.81	0	28.02	48.1	99.82	45032.23	6.82
p425	D5	126.01	1.75	56.25	11.13	4.41	53.14	0.95	6.19	1.98	36.89	2.44	19.9	127	13.74	0.77	6.88	0	47680	27.71	4568	47450	1491	372.91	205.39	1144.23	0	21.71	26.9	128.58	35545.25	6.3
p425	D6	125.2	1.57	34.11	6.96	3.53	58.62	0.17	5.89	1.15	25.19	1.82	23.57	101	14.38	1.11	7.32	0	41100	47.25	4245	48400	1372	211.11	177.44	960.82	0	20.59	21.6	109.19	42034.76	5.18
p424	Admission	58.08	15.39	82.8	58.69	42.48	305.45	6.65	48.62	12.68	11.77	14.3	10.02	79.1	43.16	28.46	9.15	22.67	2620	135	2386	20300	272	176.67	162.53	238	12.96	41.41	22.4	74.5	15186.28	25.04
p424	Admission	45.7	0.43	35.44	8.71	9.77	76.77	0	0	2.36	22.78	3.82	6.3	0	25.01	9.68	6.02	10.59	0	7.2	0	1700	13	165.77	255	226.19	0	0	20.7	27.31	15112.3	2.36
p424	Admission	41.05	0.93	38.69	9.23	11.45	66.17	0	0	2.27	21.82	2.84	4.55	0	18.95	7.76	5.91	11.54	0	3.5	0	400	0	160.42	226.94	224.05	0	9.49	23.7	24.49	11126.86	2.46
p424	Admission	25.98	0	38.69	6.26	8.21	64.03	0	0	2.46	18.08	0.97	8.97	0	19.28	7.55	5.67	14.71	0	0	0	0	0	150.86	272.3	315.95	0	11.71	24.3	24.2	13734.59	2.41
p424	D4	17.32	0.22	73.12	4.85	12.5	94.37	0	0.88	5.85	15.41	0.86	10.54	0	25.01	8.19	6.41	10.7	0	0	0	0	0	359.24	311.86	457.12	0	18.22	28.9	63.35	13623.65	5.09
p424	D5	0	1.66	48.01	9.06	9.77	78.86	0	5.89	2.83	9.81	1.4	7.84	12.5	20.28	7.97	6.26	11.33	330	20.7	204	4200	37.8	285.84	209.74	260.62	0	7.6	29.7	62.82	8477.467	4.77
p423	Admission	60.18	4.55	39.97	110.27	4.24	39.67	0	7.9	1.15	46.4	6.29	13.84	3.89	13.1	2.12	5.98	6.83	1650	46.71	348	1150	126	78.62	102.75	212.27	236.44	11.42	22.4	88.88	136213.8	1.5
p423	Admission	57.9	1.02	41.86	165.2	0	158.46	0	0	0	33.7	5.35	65.05	0.46	9.35	0.3	5.77	1.09	340	20.73	89.2	90	16.38	86.9	143.52	276.84	267.31	20.78	19.3	85.55	129702.5	0
p423	Admission	52.71	6.74	31.42	129.22	2.41	25.53	0	0	0.69	45.84	9.21	14.67	23.66	9.96	1.67	6.12	0.59	3520	157	847	4720	303	90.76	119.36	173.9	240.6	7.17	20.7	90.66	125517.5	0
p423	D1	60.86	4.63	41.23	119.91	0	15.47	0	0	0.04	33.04	5.16	11.07	0.23	7.53	0.18	5.95	0	0	0	0	0	0	140.54	114.37	187.72	273.75	0	19.7	82.48	96943.64	0
p423	D2	70.6	5.61	51	98.83	2.02	31.52	0	0	0.58	18.43	7.03	6.3	10.68	8.13	0.77	6.26	1.09	1450	67.05	391	1900	155	222.68	96.13	477.22	227.37	1.65	17.2	65.43	55275.47	0
p423	D3	73.85	4.63	0.4	176.66	0	23.07	0	0	0.36	14.63	11.96	8.27	0.46	5.75	0.3	4.82	0	0	0.37	0	0	0	259.82	98.97	559.35	353.6	21.34	21.2	81.97	27194.65	0
p423	D4	66.6	6.41	47.1	82.48	2.6	41.95	0	0	0.95	14.37	12.48	8.33	9.62	9.35	1.67	5.91	3.27	1510	64.99	357	1590	152	254.53	83.69	341.08	235.71	11.71	21.8	66.47	27194.65	1.23
p422	Admission	17.63	0	6.48	3.06	0	4.29	0	0	0	9.28	1.19	12.42	0	12.15	0.3	5.91	8.72	0	0	0	240	0	192.26	150.29	602.48	0	64.14	24.5	30.39	73894.3	1.28
p422	Admission	0	0.43	62.49	2.33	2.02	15.47	0	0	0.12	7.23	0.4	9.53	0	11.68	0.99	5.84	1.09	0	0.58	0	400	0	121.04	86.46	259.54	0	10.81	22.2	22.78	121989.2	0
p422	Admission	0	0.22	47.1	2.7	2.22	15.47	0	0	0	2.91	1.61	6.76	0	9.04	1.45	6.05	1.09	0	0	0	400	0	87.8	74.97	195.18	0	10.17	18.6	20.47	174978.8	0.12
p422	D1	0	0	41.23	0.84	0.26	12.83	0	0	0	2.7	0	5.32	0	6.93	0.65	6.12	0	0	0	0	350	0	79.87	64.6	109.57	0	0	18.4	15.18	194147.1	0
p420	Admission	25.77	0	65.65	2.49	0	21	0	0	1.31	79.14	6.13	28.42	0	33.58	0	2.34	0	180	21.33	50.46	1870	0	311.56	772.31	241.6	0	4.65	10.916	355.61	167704.7	3.95
p420	Admission	21.14	11.27	68.03	6.05	3.78	67.79	1.73	1.76	1.9	76	8.38	23.41	19.71	46.5	0	6.2	0	970	80.98	733	7730	89.1	331.58	538.09	179.29	0	11.92	8.15	221.8	187172.6	4.58
p420	D1	0	0	62.55	2.25	0	12.11	0	0	1.23	34.28	1.73	13.42	1.55	10.27	0	2.84	0	130	22.02	0	2060	0	230.56	250.08	165.33	0	0	6.946	256.25	181896.2	1.69
p420	D1	0	3.06	65.31	3.71	0.86	49.07	0.52	0	1.75	41.01	7.37	21.17	15.93	10.27	0	4.04	0	780	54.27	572	6970	69.16	311.56	548.15	180.36	0	0	8.932	259.61	77326.4	4.01
p420	D4	3.73	0.82	60.45	3.47	7.62	103.64	0	0	1.84	52.63	6.13	9.37	0	11.85	0	2.56	0	0	0.58	0	0	0	311.95	422.39	456.95	0	0	26.898	324.64	33082.3	2.64
p420	D5	40.02	0	49.6	0	0	150.45	0	0	1.23	8.47	4.01	101.18	0	3.91	0	1.59	0	0	0.58	0	0	0	470.7	585.14	577.28	0	9.67	30.091	268.22	11612.9	4.98
p420	D6	10.89	11.14	71.71	11.67	4.09	102.04	1.87	0.38	2.02	13.49	10.47	10.54	15	16.34	0	3.93	0	590	50.88	435	5530	47.37	1325.15	356.79	275.08	0	23.38	7.542	228.34	24282.3	7.86
p420	D7	13.04	7.5	114.01	15.14	0	40.3	0	0	1.39	20.73	5.57	12.11	0	7.45	0	2.45	1.85	0	0	0	0	0	1870.33	667.46	478.73	0	31.32	9.726	416.49	13555.8	5.63
p419	Admission	56.15	13.24	70.05	5.36	9.28	95.63	4.2	5.46	2.7	10.5	15.32	9.43	4.58	15.21	0	4.63	0.23	300	43.37	43.52	3050	0	475.14	338.09	586.94	0	35.84	13.891	33.14	34877.4	6.99
p419	Admission	39.73	9.54	46.96	0	2.83	49.07	1.32	0.02	1.78	11.15	5.94	5.98	8.54	6.51	0	3.18	0	960	80.98	862	8160	90.75	658.69	529.47	619.38	0	33.51	32.734	23.23	32555.9	5.07
p419	D1	6.97	0.82	25.83	0	0	14.14	0	0	1.43	8.84	1.73	5.8	0	3.76	0	1.48	0	180	35.13	0	2400	0	626.44	347.88	546.64	0	15.95	28.578	6.84	19818.1	2.32
p417	Admission	219.54	591.35	181.38	9	555.26	415.36	429.59	22.07	3.27	86.88	14.62	4.9	64.38	20.23	10.54	59.84	1.25	350	133	273	2230	23.18	645.54	348.42	313.6	0	551.79	17.261	597.35	93398.3	17.38
p417	Admission	362.57	766.58	259.84	5.82	87.71	457.16	12.94	13.99	2.32	113.93	15.6	6.01	78.16	14.73	0	17.31	0	770	145	703	4490	71.31	1106.54	589.16	282.77	0	1172.22	15.843	1037.47	67446.1	18.19
p417	D1	237.21	601.7	165.35	0	492.65	264.8	282.67	3.62	1.47	102.83	9.68	3.95	75.78	12.49	0	529.13	0	680	143	564	3820	49.07	1014.78	601.2	261.63	0	581.6	14.099	635.73	78847.1	13.55
p417	D1	145.89	296.13	131.09	0	268.31	215.8	179.57	7.12	1.47	79.45	8.22	4.25	58.71	14.41	0	266.37	0	750	105	674	4390	57.19	1105.17	541.2	250.11	0	334.3	20.506	408.86	58672.7	8.41
p417	D2	261.96	761.09	128.62	0	550.14	241.51	436.98	2.1	1.44	86.67	5.57	0.48	41.97	8.38	0	736.37	0	190	81.47	48.2	1570	8.72	1029.65	504.02	291.44	0	601.27	6.747	568.05	22044	9.27
p417	D3	270.98	882.68	143.51	0	428	141.23	593.39	0	1.48	64.36	5.76	1.8	30.84	9.48	0	723.03	0	390	58.34	227	2290	26.07	1837.56	629.82	381.09	0	585.1	8.336	402.15	19683.7	8.1
p416	Admission	9.82	20.63	110.27	25.69	14.14	121.02	5.2	17.88	3.58	8.57	18.58	5.86	35.93	22.84	0	5.35	8.83	1560	75.8	401	2200	116	233.31	214.42	186.39	0	22.92	56.356	109.95	139022.9	10.5
p416	Admission	7.65	0	60.1	26.11	0	3.42	0	0	1.2	11.94	0.54	4.92	2.91	101.67	0	1.8	1.25	170	11.56	49.25	120	18.4	362.66	300.42	254.52	0	20.55	22.018	155.06	157907.6	3.64
p416	Admission	0	0	54.02	6.97	0	0	0	0	1.24	8.77	2.24	2.51	19.29	9.95	0	1.69	0	560	31.62	176	1230	57.23	111.86	218.61	167.49	0	0	21.82	116.07	208021.4	1.75
p416	D1	19.48	0	59.04	6.28	0	3.42	0	0	1.28	28.65	1.73	11.17	26.65	13.45	0	2.78	0	740	36.71	235	1800	70.65	200.01	535.44	134.68	0	0	21.423	183.01	211391.2	3.14
p416	D2	0	0	46.96	0	0	0	0	0	1.15	9.59	0.54	2.83	0.7	3.91	0	2.34	0	120	7.19	0	180	15.44	200.51	234.25	79.14	0	0	16.666	100.99	176385.6	1.15
p416	D3	17.86	0	44.27	0	0	35.83	0	0	1.69	33.21	1.73	5.3	0.25	8.7	0	2.95	0	120	6.44	0	200	15.44	336.11	467.14	140.22	0	0	25.985	81.55	59518.65	2.13
p416	D4	9.2	0	44.65	0	0	40.3	0	0	1.84	27.2	1.46	7.6	0.7	10.43	0	2.73	0	120	9.04	0	150	10.27	431.78	487.65	120.5	0	0	6.747	35.93	38507.61	2.26
p416	D5	0	0	58.33	0	0	42.07	0	0	1.5	13.56	1.99	4.55	3.77	7.45	0	2.23	0	120	10.13	0	350	19.66	345.07	362.82	125.38	0	0	9.924	18.3	26614.72	1.82
p416	D6	0	0.05	67.35	0	0	45.58	0	0	1.61	12.13	3.38	4	17.23	4.98	0	2.12	0	120	44.23	307	1990	71.79	444.28	349.4	141.33	0	0	5.952	12.37	27779.8	2.57
p415	D5	3.55	0	10.19	1.49	0	18.1	0	0	1.31	33.77	7.37	9.53	13.5	15.37	0	2.45	0	590	28.39	624	4350	85.26	278.94	630.93	166.77	0	92.32	11.511	186.45	23747	3.39
p415	D5	0	0.05	16.47	2.98	0	20.04	0	0	1.29	33.55	3.59	13.67	9.53	18.93	0	3.58	0	300	18.01	215	2770	34.94	553.76	684.29	274.74	0	51.95	11.908	151.82	23866.4	4.01
p415	D6	0	0	28.55	0	0	29.45	0	0	1.33	180.79	3.59	19.94	44.91	37.42	0	2.56	0	1330	76.68	1114	9670	123	414.37	461.79	431.24	0	33.12	16.27	138.31	242443.2	2.48
p415	D7	0	0	32.05	0	0	27.6	0	0	1.33	133.31	1.99	16.85	13.01	18.93	0	3.58	0	350	26	296	2810	31.96	220.79	391.82	206.76	0	15.13	7.049	165.74	105976.5	1.88
p414	Admission	44.36	5.97	34.18	0	6.1	66.11	0	4.12	1.72	16.08	15.04	5.1	27.32	6.51	0	4.4	0	1280	51.85	1139	8670	145	129	310.13	79.14	0	22.69	16.64	71.05	43595.9	6.81
p414	Admission	38.58	3.51	24.43	0	3.15	46.46	0	1.42	1.61	18.65	7.71	5.05	12.03	3.76	0	3.64	0	620	20.4	492	3890	70.31	130.27	284.13	73.13	0	23.61	15.045	72.63	22720.1	5.32
p414	Admission	19.39	0	24.9	0	2.51	18.1	0	0	1.35	15.56	4.81	2.02	39.01	4.06	0	3.41	0	1220	65.88	1105	9530	119	113.91	125.85	80.34	0	0	5.156	39.81	35011.6	1.11
p414	D1	43.81	0	11.41	0	1.2	10.04	0	0	1.35	19.93	5.94	2.3	8.76	3.46	0	3.93	0	250	12.46	71.97	2130	19.42	233.31	186.65	98.77	0	0	17.855	46.12	18791.2	1.34
p413	Admission	10.06	1.16	32.91	0	0	23.86	0	0	1.55	7.95	8.55	8.95	7.4	5.9	0	2.56	0	290	31.6	176	3400	57.5	327.77	303.04	497.91	0	0	14.85	48.84	170989.6	5.57
p413	D1	17.96	0	27.65	0	0.15	17.12	0	0	1.52	12.45	9.04	8.25	6.3	12.17	0	4.16	0	300	27.7	167	3200	49.9	311.95	313.62	485.55	0	6.38	13.296	67.34	121718.3	6.37
p413	D1	26.61	2.14	32.05	0	0	18.1	0	0	1.52	12.81	4.81	14.21	15.8	11.06	0	3.35	0	570	44.5	533	5700	71.2	505.06	421.41	567.6	0	4.18	20.624	45.3	105056	5.35
p413	D1	26.7	0.05	26.29	0	0	17.12	0	0	1.47	10.11	5.57	7.68	18	8.7	0	3.46	0	680	53	650	4700	94.3	644.17	486.42	611.29	0	0	26.183	51.82	71534.81	5.69
p413	D2	19.85	0.46	34.6	4.42	0	12.11	0	0	1.55	7.62	5.2	13.59	5.7	9.48	0	4.28	0	200	19	0	2700	36.7	703.87	671.26	56.72	0	18.06	41.483	55.86	44307.86	6.87
p413	D3	0	1.82	23.01	0	0	0.87	0	0	1.67	4.94	4.01	6.85	5.1	8.07	0	2.73	0	170	18.1	0	2900	25.2	775.4	497.55	647.72	0	0	41.882	46.39	28066.96	4.89
p412	Admission	26.2	23	54.02	12.54	15.89	164.17	6.41	20.46	3.93	19.8	19.37	14.81	33.2	89.45	0	6.33	8.45	1070	74.2	839	7100	95.8	302.88	1145.41	242.97	0	31.12	27.77	171.92	242046.3	13.79
p412	Admission	0	0	13.73	0	0	10.04	0	0	1.23	14.43	1.18	11.65	0	16.34	0	2.78	0	130	11.3	0	2400	13	376.99	871.7	209.2	0	0	24.001	183.5	310764.5	2.64
p412	Admission	0	0	12.59	0	0	4.59	0	0	1.25	14.4	0	13.96	0	14.57	0	2.78	0	200	14.7	0	3400	28.7	393.13	1068.29	240.58	0	0	26.381	210.39	380826.9	3.33
p412	D1	14.12	2.6	33.76	0	0	27.6	0.67	0.73	1.39	36.2	8.38	15.84	8.5	40.1	0	4.87	0	470	37.6	327	4900	63.9	444.28	2775.99	363.42	0	15.13	24.993	438.63	241716.8	6.44
p412	D2	0	3.21	17	0	0	10.04	0	0	1.35	65.35	1.73	17.51	0	29.76	0	3.52	0	210	17.2	0	3200	38.9	751.25	2404.54	229.61	0	0	18.053	302.45	227942.7	4.2
p412	D4	15.78	7.36	44.27	0	2.67	106.82	0.6	2.78	1.96	10.96	12.74	7.24	26.9	25.3	0	3.81	0	840	59.3	878	7100	91.8	589.74	1497.93	319.84	0	17.54	5.952	284.42	34742.9	8.9
p412	D5	5.82	2.6	23.01	0	0	113.95	0	0	1.75	10.96	0	8.51	0	24.48	0	3.01	0	130	10.5	0	2300	20.4	1033.36	1683.52	212.33	0	0	9.924	271.08	27420.29	6.81
p412	D6	0	0	29.44	0	0	83.48	0	0	1.39	6.1	0.54	6.88	15.8	18.93	0	2.23	0	490	36.3	417	6100	57.5	575.06	971.1	237.84	0	0	13.891	176.85	22156.96	4.33
p412	D7	16.38	11.67	60.1	6.74	5.03	128.06	2.27	10.73	2.43	9.39	17.24	10.06	19.3	36.75	0	4.16	0.23	690	43.2	760	7100	74.9	608.49	1264.26	234.08	0	32.33	18.648	328.64	25596.17	11.96
p411	Admission	46.8	69.59	67.01	29.81	30.23	203.95	20.64	226.15	109.46	100.11	28.94	28.79	75.2	54.13	0	7.63	19.83	480	111	1357	6000	204	324.72	5086.59	144.27	52.37	1008.33	15.08	2080.06	97628.65	23.42
p411	D1	38.44	38.91	67.35	31.65	28.83	305.12	15.08	37.56	62.19	89	25.14	26.97	43.5	61.47	9.13	12.36	22.2	400	67.4	991	4000	159	546.08	792.25	132.09	20.51	66.43	20.624	261.04	100831.6	19.65
p411	D1	19.29	1.82	6.19	0	0	55.1	0	0	48.25	58.78	5.2	19.11	5.8	21.37	0	3.52	0	0	0.3	0	0	8.4	567.52	1072.06	140.22	0	15.95	3.362	203.57	84404.4	7.24
p411	D2	0	14.66	8.93	0	0	61.06	4.33	0	29.03	250.82	6.31	16.66	9	53.11	0	7.95	0	90	5.1	17.7	300	22.8	2923.75	731.26	731.45	0	0	7.145	262.48	49726.2	6.44
p411	D3	0	27.07	32.05	6.51	3.15	79.38	3.89	1.07	16.48	67.76	9.04	9.74	16.6	47.52	0	7.07	0	110	13.9	78.6	600	38.1	3733.62	576.89	2567.07	0	18.06	14.247	342.96	50294.2	8.41
p411	D6	51.49	55	95.46	31.86	36.62	340.75	12.94	48.49	14.48	18.55	34.38	19.56	27.2	51.24	4.15	6.82	28.19	270	43	540	2800	106	2493.66	1077.34	1158.38	37.14	76.39	15.278	356.54	22630.3	27.63
p411	D7	0	11.4	32.05	3.94	7.77	100.44	4.45	9.26	3.86	13.43	13.62	5.45	22.9	16.66	0	1.59	0	230	33.6	457	2100	94.8	1051.61	562.63	440.04	0	20.06	15.08	303.4	30621.8	7.36
p409	Admission	0	0	0	0.75	0	0.39	0	0	0.01	11.37	0	2.75	0	2.13	0.4	0.86	0	0	5.62	0	0	0	89.22	325.42	22.23	0	0	14.758	6.8	27501.25	0
p409	Admission	30.72	0.38	0	1.12	0.19	10.21	0.15	0	0.2	71.63	2.1	82.46	23.75	8.51	0.67	1.28	0	1450	97.53	1082	3910	203	246.61	257.07	25.54	0	8.17	0	19.18	30715.42	0.22
p409	Admission	55.22	0.53	0	1.38	0.41	13.05	0.41	0	0.53	48.25	14.19	5.22	0	19.55	0.94	1.84	0	470	26.65	215	320	54.82	193.36	330.64	23.59	0	0.82	18.428	17.59	35524.21	0.41
p409	D1	0	0	0	0.76	0	0	0	0	0	0	0	3.61	0	0.92	0.34	0.71	0	330	17.56	109	130	27.1	153.13	260.99	55.58	0	0	35.733	4.32	26385.01	0
p408	Admission	30.09	0	0	0.88	0	0	0.05	0	0	36.24	0	4.72	0	8.78	0.42	1.78	0	80	2.91	0	660	10.69	87.23	276.19	13.25	0	7.48	18.766	4.85	200100.2	0.1
p408	D1	0	0	0	0.93	0	0	0	0	0	20.04	0	3.48	0	15.78	0.49	2.42	0	20	0	0	370	0	189.77	291.19	9.84	0	1.49	28.508	1.6	121285	0
p408	D1	0	0	0	0.83	0	0	0	0	0	0	0	1.41	0	7.7	0.42	1.43	0	20	0	0	240	0	147.16	214.27	15.33	0	0	25.676	1.54	92698.5	0
p408	D2	0	0	0	0.75	0	0	0	0	0	0	0	0.5	0	7.16	0.41	1.77	0	70	3.56	0	770	8.35	89.46	162.6	10.02	0	0	25.676	4.85	33117.2	0
p408	D3	16.38	0	0	0.81	0	0.19	0	0	0	0	0	0.52	0	7.56	0.46	1.03	0	160	7.09	60.04	1370	18.5	166.7	217.51	7.4	0	0	24.974	3.8	25628.5	0
p407	Admission	0	0.49	0	1.4	1.2	8.36	0.57	28.02	0.24	44.9	11.85	8.18	0	23.92	1.83	5.69	0	0	6.4	0	500	9.2	129.87	139.06	34.78	0	15.08	62.699	21.8	327776.2	3.62
p407	D1	0	0	0	0.76	0	0.15	0	0.87	0	50.31	0	10.05	0	21.37	0.45	3.8	0	0	2.2	0	100	0	235.33	240.15	29.54	0	13.7	126.325	11.74	255881.9	0
p407	D1	0	0	0	0.76	0	0	0	0	0	51.72	0	6.84	5.1	36.24	0.41	3.66	0	230	19	0	3600	35.6	372.45	379.37	24.33	0	13.45	50.705	2.77	196941.7	3.12
p407	D1	0	0	0	0.67	0	0	0	0	0	39.07	0	5.79	0	13.22	0.36	3.35	0	180	16.4	0	2500	35.6	352	376.83	30.77	0	11.41	86.282	1.78	177442.4	3.07
p407	D2	0	0	0	0.66	0	0.19	0	0	0	44.15	0	3.15	0	23.99	0.39	3.27	0	0	1.1	0	0	0	405.16	515.82	13.18	0	8.47	15.418	3.15	75221.74	3.07
p407	D3	0	0	0	0.7	0	1.06	0	0	0.03	17.89	0	2.94	0	11.98	0.46	2.09	0	0	0	0	0	0	527.87	422.66	23.47	0	1.49	12.8	2.77	49578.43	2.42
p407	D4	0	0	0	0.75	0	2.94	0	0	0.03	0	0	2.39	0	8.1	0.46	1.47	0	0	0	0	0	0	99.77	53.09	35.86	0	3.24	12.477	8.25	29832.27	0
p407	D5	0	0	0	0.89	0	0.74	0	0	0	17.11	0	3.23	0	8.1	0.36	1.9	0	0	0	0	0	0	317.36	299.65	42.38	0	0	13.775	5.11	26063.43	0
p407	D6	0	0	0	0.77	0	0.65	0	0	0.02	7.94	0	3.47	0	11.23	0.49	1.64	0	0	0	0	0	0	350.24	220.95	27.08	0	0	17.417	3.15	17860.66	0.88
p407	D7	0	0	0	0.8	0	2.58	0	0	0.01	0	0	2.59	0	9.18	0.39	1.25	0	0	0	0	0	0	347.26	158.67	19.47	0	0	15.088	4.32	14354.05	0
p35	Admission	2.9261	0	0	3.3209	108.905	114.5523	10.8664	1.1059	1.1129	177.9846	0	7.1452	0	35.3587	6.867	2.7313	0	0	0	0	0	7.1	3.8292	130.2068	0	16.606	43.0157	42	91.0737	11579.37	10.5373
p341	Admission	78.8	0	50.9	299.8	0	732.8	7.6	59.8	50	158	59.5	29.2	49.7	8.4	0	19.6	0	190	47.1	733	3700	53.1	228.8	509.1	139.5	106.5	78.6	120.8	388.1	90091.54	0
p341	D1	96.7	0	55.1	288.1	10.4	767	7.6	54.3	50.1	158	60.5	32.5	68.4	11.8	0	10.3	0	290	68.9	1019	4200	101	188.1	655.6	123	102.3	68.5	117.8	424.5	121482.6	0
p341	D1	72.5	0	41.8	186.1	12	538.9	7.6	45	49.7	122.8	59.5	44.9	41	11.8	0	15	0	130	38	535	2300	32.9	180.9	588.2	74.3	87.6	56.1	98.9	432.5	178112.9	0
p341	D2	54.6	0	41.8	217.6	0	538.9	6.9	46.9	49.7	54.6	58.4	18.3	48.5	6.6	0	7.1	0	90	25.6	422	2100	13.1	178.3	368.8	85.3	83.3	56.1	100.3	550.6	110804.9	0
p341	D3	61.8	0	46.5	233.3	0	538.9	6.9	56.2	49.9	49.3	58.4	15.7	50.4	9.4	0	10.3	0	140	38.3	637	2900	34.3	177.9	427.5	95.5	91.8	54.1	98.4	554.5	92376.91	0
p341	D4	73.1	0	36.7	209.8	0	447.9	6.6	39.3	49.4	43.3	61.6	23	41.5	9.4	0	8.7	0	110	31.7	522	2300	22.2	214.1	504.9	79.9	81.2	45.7	74.7	538.9	70502.21	0
p341	D7	77.8	0	41.8	201.9	0	516.1	4.6	38.4	49.7	35.1	57.4	18.3	52.9	5.5	0	0	0	170	50.2	855	3500	50.3	183.3	425.2	74.3	81.2	56.1	45.6	492	45666.61	0
p338	Admission	42.5	0	57.2	307.5	7.8	812.8	7.2	63.5	50.4	419.5	66.8	87.4	157	20.6	0	10.3	0	740	75.4	791	5970	97.04	54.4	496.3	85.3	108.6	64.4	19.4	254.4	45078.2	0
p338	D1	19.9	0	36.7	194	5.9	357.2	9	41.2	49.4	468.1	64.2	18.3	109	9.4	0	0	0	140	22.27	0	1510	0	46.7	215.4	32.4	74.7	58.2	3.2	245.7	112420.7	0
p338	D1	21.4	21.3	25.1	162.3	0	311.9	8.6	56.2	49.7	405.6	54.2	24.7	106	8.9	0	0	0	0	2.76	0	30	0	50.4	172.2	0	72.6	56.1	81.6	201.8	64153.35	0
p338	D3	40.6	0	59.1	400.6	24.5	584.4	11.2	63.5	50.4	136.5	63.6	26.4	81.06	8.4	15.9	28.6	0	760	63.32	740	5540	126	89.8	388	123	106.5	76.6	2.1	380	15499.04	0
p338	D4	43.3	70.5	107.7	419.9	37.5	904.4	14	67.1	51.1	32.4	69.8	23.5	7.66	16.4	38.8	67.6	12.2	170	34.15	65.67	2450	0	299.2	326.9	147.4	129.1	129	6.1	371.8	35915.26	2.4
p331	Admission	98.3	24.2	256	504.6	119.6	10773.3	32.8	179.2	54.4	1806.6	102.9	565.3	0	139.9	73.3	508.1	410.5	450	15.28	11.73	330	38.33	53	3489.9	262.8	350.5	402.3	23.9	1286.1	85043.6	41.5
p331	Admission	38.9	0	126.2	260.7	78.1	3288.3	15.1	83.2	51.1	989.2	69.8	358.5	0	63.1	28.8	114.9	93.3	430	16.97	0	470	56.99	79.4	1715.4	131.4	159.4	118.3	18.8	821.4	89149.74	11.5
p331	Admission	25.3	1.7	73.9	299.8	37.5	835.7	14.6	70.7	50.9	331.3	70.9	48.9	0	15.3	25	54.8	55.8	0	11.95	0	80	31.8	72.3	592.2	105.1	106.5	71.5	14.3	638.7	87840.47	5.4
p331	D1	29.4	11.2	90.2	431.4	21.7	858.6	11.2	86.7	51.1	368.3	69.8	45.5	12.92	13.3	28.8	57.7	61.1	930	44.74	413	1720	108	59.5	834	131.4	139.3	98.6	5.8	676.5	85290.55	3.9
p331	D2	43.9	0	143.2	225.5	95.6	1432.9	30	86.7	50.9	440.3	79	46.6	7.61	9.9	35.6	173	108.3	570	23.78	120	770	73.11	123.9	1401.2	105.1	163.4	127.1	12.8	780.9	82171.65	12.3
p331	D3	55.3	0	214.5	209.8	120.1	2079.7	34.6	128.3	52.1	663.7	86.1	28.9	11.93	10.3	62.3	321.2	242.8	810	36.63	230	1310	102	141.2	1368.3	131.4	270.1	218.4	7.4	1310.2	81433.43	21.9
p331	D4	33.7	0	117.1	466.1	85.4	1283.2	25.2	93.7	52.3	185.4	85.1	48.3	0	8.9	28.8	131.2	178.6	0	13.19	0	230	30.5	107.5	900.7	180.1	177.3	148.4	8.2	1090.8	77943.31	6.9
p331	D5	49.5	0	114.5	342.5	0	6458.7	17.3	96.4	51.1	263.5	67.8	321.4	0	8.9	38.8	97	130.5	0	1.09	0	40	0	91.6	624.2	162.4	157.4	273.9	11.2	1090.8	72020.25	2.2
p331	D6	63.9	35.1	335.1	535.3	190.9	4012.4	60.8	174.1	56	137.1	111.6	41.6	0	11.8	59.9	252.4	291.8	0	11.95	305	860	54.98	73.8	855.3	420.9	376.3	357.5	3.4	1202.9	75678.11	22.9

p331	D7	163.7	164.5	599	868.1	369.6	11893.3	186	531.8	82.1	158.3	373.3	64.7	7.84	47.3	217.8	1114.2	973.9	620	19.51	494	2240	117	75.6	1226.3	1282.5	878.5	1169.7	2.7	2313	69557.93	99.4
p329	Admission	39.8	352.1	83.9	650.3	46	618.6	11.5	68.9	51.1	418.3	63.6	43.2	21.02	72.2	0	3.8	0	700	68.58	439	7100	65.86	65.2	380.8	176.7	120.9	1121	11.9	228.3	74860.13	0
p329	Admission	31.7	733.6	90.2	256.8	123	721.3	31.3	64.4	49.7	555.2	60.5	18.8	0	237.2	0	0	0	80	5.32	0	1020	5.04	101.4	221.3	114.3	108.6	102.6	5	228.3	172448.2	0
p329	D1	19.4	270.2	46.5	162.3	57.5	311.9	12.8	39.3	49.4	327.1	59.5	13.9	5.31	60.2	0	0	0	170	13.24	27.27	2530	21.24	96.5	131.6	48.7	76.9	52	5.4	174.8	90274.17	0
p329	D2	21.7	215.4	36.7	186.1	39.9	334.5	9.4	35.5	49.2	148.8	58.4	13.5	7.42	39.4	0	0	0	250	19.36	79.38	3250	34.03	128.6	131.6	48.7	76.9	47.8	10.6	237	57776.64	0
p329	D3	30.1	191.9	73.9	264.7	0	1640.4	5.5	74.3	49.7	53.9	57.4	101.2	0	27.2	0	0	0	10	0.26	0	0	0	84.4	159.1	85.3	131.2	214.7	5.9	275.8	29614.43	0
p329	D4	30.1	359	70.4	272.5	60.8	675.7	16.9	59.8	49.9	60.5	61.6	17.9	12.36	58.6	0	0	0	410	32.74	282	4520	40.91	164.1	203.4	100.4	100.2	66.4	7.8	263	20130.74	0
p329	D5	59.9	466.9	106.3	673.2	69.6	1133.8	20.1	90.2	51.1	87.3	70.9	40.5	18.28	101.3	0	19.6	0	790	56.32	717	8010	96.05	75.6	411.4	251.6	147.4	178.9	8.7	363.6	26869.02	0.4
p328	Admission	44.6	44.8	70.4	466.1	0	1317.7	7.2	67.1	50.4	72.9	79	57.6	2.78	10.3	41.7	13.4	0	710	78.93	643	5650	105	154.4	515.5	176.7	124	162.7	28.78382	1034.1	68469.28	0
p328	D1	39.5	24.2	55.1	233.3	0	1179.7	5.2	56.2	49.9	43.3	72.9	35.1	0	8	28.8	0.3	0	0	2.28	0	0	0	105.7	429.8	114.3	109.6	165.6	186.89	1083.7	55940.96	0
p328	D2	65.3	40	66.8	396.7	0	1317.7	5.2	63.5	50.3	42.6	70.9	70.6	0	8.9	30.6	10.3	0	300	49.69	296	3080	25.13	87.4	914.6	147.4	129.1	159.9	249.7	969.9	25937.25	0
p328	D3	32.1	24.2	46.5	186.1	0	744.2	7.2	35.5	49.7	21.9	66.8	19.7	0	7.7	32.4	13.4	0	0	12.48	0	680	0	172.6	238.7	62.2	79	90.6	135.7	879.7	27527.86	0
p328	D4	39.3	58.1	55.1	249	0	1386.8	6.2	56.2	50.4	32.7	75.5	40	0	11.1	41.7	16.5	0	320	41.75	263	3080	28.02	259.6	526.1	131.4	104.4	110.5	158.7	1143.5	45280.59	0
p327	Admission	43.3	1.7	73.9	761.2	0	698.5	7.9	77.9	52.9	32.4	70.9	52	26.87	8	9.7	40.4	21.2	850	96.82	666	7920	84.05	75.6	467.9	216	137.3	205.3	23.9	219.5	78292.21	0.1
p327	Admission	41.2	1.7	73.9	657.9	0	698.5	7.1	76.1	52.9	42.6	68.3	42.1	7.17	7.1	9.7	34.5	9.1	540	45.36	432	4290	56.8	73.1	314.1	176.7	129.1	257.4	22	201.8	60261.05	0
p327	D1	48.2	0	73.9	427.6	0	1987.1	4.6	72.5	51.9	43.9	62.6	215.2	0	6.6	9.7	3.8	0	0	1.05	0	0	0	85.6	359.1	85.3	125	491.6	15.2	183.9	25558.76	0
p325	Admission	47.1	18.2	66.8	535.3	45.4	881.5	11.9	68.9	52.1	196.9	67.8	87.4	98.52	9.4	32.4	34.5	0	4980	173	2782	10610	463	85.5	763.4	176.7	129.1	150.3	14.9	424.5	101123.1	0.4
p325	Admission	32	1.7	50.9	264.7	38.7	630	9.5	61.7	50.4	137.7	67.8	22.5	42.18	8.4	32.4	13.4	0	2550	105	1869	4760	335	116.2	402.1	85.3	104.4	80.6	8.5	388.1	91789.58	0
p325	Admission	26.4	1.7	39.3	225.5	20.3	984.6	10.1	57.1	50.4	235.4	67.8	418.6	26.84	7.5	25	0.3	0	1970	85.13	1578	3940	283	86.5	730	74.3	96	80.6	16.1	404.3	70919.91	0
p325	D1	27.9	0	50.9	249	27.2	584.4	9	52.5	50.4	74.2	67.8	19.7	11.74	6.2	25	13.4	0	690	35.69	525	1160	114	93.7	487.6	62.2	96	78.6	11.3	468.3	81434.48	0
p325	D3	94.5	0	61.1	342.5	0	698.5	4.9	63.5	50.4	14.7	62.6	41	15.43	5.7	20.8	0.3	0	1550	93.42	1322	2460	189	46.7	893.8	85.3	116.8	86.6	30.8	412.4	16533.22	0
p324	Admission	52.9	0	52.4	295.5	0	608.2	0	57.9	24.5	416.8	0	27.4	2.86	18.2	63.6	15.8	41.2	140	28.44	0	1980	0	131.2	431.4	93	110.9	110.5	13	377.7	45173.06	2.3
p324	Admission	58.2	0	42.3	256	0	470.2	0	52.8	24	480.4	0	26.7	0	17	63.6	6.2	30.4	0	10.92	0	690	0	134.8	504.3	61.4	108.5	80	33.8	350.8	49365.82	1.5
p324	D1	63.1	0	45.8	216.2	13	568.6	0	47.7	24	658	0	28.8	0	17.8	62.6	8.6	21.9	0	10.17	0	520	0	192.6	621.4	61.4	91	72	41.3	346.9	42408.16	1
p324	D1	42	0	34.8	159	0	431	0	40.1	23.6	488.3	0	21.2	0	16.7	63.6	3.8	9.8	0	3.73	0	90	0	191.9	348.4	45	80.2	63.8	10.7	470.3	41689.55	0.6
p324	D2	89.5	0	52.4	209.5	0	1171.9	0	47.7	24	77.9	0	131.8	0	16.7	64.6	0	21.9	0	0	0	0	0	43.9	418	38.9	98.6	929.5	21.9	576	93530.21	1.5
p324	D3	95.7	18.8	70	1007	0	647.9	0	63	30.6	42.7	25.6	80.6	0	17	64.6	56.7	21.9	0	10.54	0	920	0	59.7	357.5	171.7	139.1	466.5	8	407.9	63997.38	2.3
p324	D4	116.3	0	55.5	331.5	17	588.4	0	60.4	24	15.4	0	67.1	0	16.1	64.6	20.6	46.5	0	0	0	0	0	52.6	293.1	50.8	127	656.6	35.3	576	58180.32	3.6
p322	Admission	22.8	567.2	61.5	1244.2	23	787.6	0	72.1	31.2	166.4	120.7	90.8	0	97.4	138.5	102.7	114	210	24.25	62.4	3080	0	52.1	271.3	264	159.1	308.7	30.3	513.2	574635.2	5.2
p322	Admission	17.6	476.6	40.5	196.1	30	295.3	0	40.1	24.7	173.4	98.4	9.1	0	79.7	124.7	3.8	115.2	0	0	0	0	0	73	173	50.8	83	63.8	29.7	433.8	595568.8	0.6
p322	D1	34.3	475.8	38.7	189.4	35	392	0	45.2	25	153.7	82.9	6	0	69.4	114.2	8.6	131.4	0	0	0	0	0	98.5	476.2	45	91	66.9	24.1	381.5	157921.7	1.5
p322	D2	26.9	431.5	52.4	295.5	61	3157.2	3.2	56.6	24.7	134.2	87.9	376.3	0	62	111	6.2	145.1	0	0	0	0	0	82.9	574.8	50.8	115.6	157.7	22.6	452.2	81999	2.7
p322	D3	25	325.5	52.4	341.3	65	888.1	8.3	54	25.5	269.5	67.5	24	0	57.8	103.7	23	196.3	0	6.92	0	630	0	93.9	290	84.7	103.6	87.8	7.8	433.8	237683.4	2.3
p322	D4	29.5	511.5	34.8	202.8	27	767.6	0	41.4	25	222.3	71.3	22.6	0	68.8	104.7	15.8	78.1	0	0	0	0	0	83.3	276	32.1	84.3	59.7	6.7	315.5	133655.3	1
p322	D5	27.8	535.7	49.2	272.5	34	787.6	0	50.2	25.2	210.3	74.9	22.6	0	80.3	105.8	23	124.5	140	17.64	0	2070	0	130.5	283.8	80.3	96.1	72	4.8	373.9	60483.62	2.5
p322	D7	34	419.1	83.1	341.3	16	1171.9	0	67.5	28.7	94.6	88.8	39.6	0	62.8	113.1	42.2	149.6	0	5.58	0	300	0	172.3	293.1	88.9	133.7	123.3	5.2	534.3	37370.26	10
p319	Admission	23.4	0	16.2	121.4	0	181.1	0	27.7	23.1	36.6	0	0	11.6	15.9	61.6	0	0	690	69.9	751	2800	86.49	53.8	125.3	50.8	56.3	42.2	13.5	245.6	87161.76	0
p319	Admission	26.4	0	0	0	0	125	0	13.1	22.9	76.3	0	3.9	0	13.3	61.6	0	0	0	35.45	166	700	15.61	81.3	208.3	0	25.2	34.1	12.7	155.8	106034.5	0
p319	Admission	39.1	0	9.6	20.6	26	162.3	0	14.9	22.9	89.9	0	0	0	13.7	61.6	0	0	420	53.84	260	1030	42.7	161.1	240	0	34.3	44.5	10	183.9	77071.79	0
p319	D1	39.2	0	34.8	148.8	13	314.6	0	42.6	23.6	84.5	0	8.3	37.3	14.4	62.6	0	33.1	1650	155	1145	6140	191	173.7	255.7	32.1	80.2	59.7	6.7	254.1	41771.06	0
p319	D2	44.1	0	42.3	216.2	22	568.6	0	50.2	23.8	51.5	0	2.6	34.82	14.4	62.6	6.2	51.7	1770	216	1511	6110	190	201	201.9	38.9	93.6	57.5	6.7	327.4	16849.98	0.1
p319	D3	44.4	0	55.5	249.4	29	548.8	0	50.2	24.3	52	0	8.3	66.21	14.8	62.6	2.7	27.6	2930	167	972	5190	284	220.3	182.6	61.4	103.6	63.8	5.4	295.4	15083.53	0.4
p319	D4	84.2	0	64.4	585.6	34	827.8	0	59.2	27.5	46.2	28.7	51.7	77.32	16.7	69.7	33.8	38.5	3180	405	2487	13680	329	154.9	425.5	116.1	128.1	101.1	7.4	343	25116.97	1.3
p319	D5	42.4	0	42.3	275.8	34	647.9	0	50.2	24.5	67.1	0	6	47.37	15.5	62.6	11	34.5	1980	194	1355	7970	205	249.1	200.3	80.3	103.6	70	16.1	373.9	26713.84	0.6
p318	Admission	127.4	0	110.9	282.4	0	588.4	0	63.6	25.1	63.9	0	16.3	0	15.2	69.7	119.7	0	0	4.89	93.09	0	2.39	233	432.9	66.4	145.5	177.4	18.7	370.1	379278	13.7
p318	Admission	34.4	0	47.5	249.4	12	868	0	49	23.8	216.9	0	345	6.3	25.4	62.6	13.4	0	810	45.81	1068	1570	121	82.1	1784.6	68.8	93.6	85.9	55.4	279	418203	2.3
p318	Admission	28	0	9.6	0	0	314.6	0	10.8	22.6	273.5	0	198.4	0	25.4	62.6	0	0	0	0	0	0	10.73	78.5	1345	15.5	20	65.9	59.7	193	439407.8	0
p318	D1	44.9	0	61.5	556.9	5	647.9	0	61.7	24.7	224.6	0	98.6	40.44	24.6	63.6	56.7	0	1890	91.26	1696	6530	260	109.8	1080.7	137.1	115.6	162.9	45.5	303.4	331190.4	1.5
p318	D2	37.5	0	9.6	86.5	0	509.4	0	20.4	22.9	97.1	0	26.4	0	20.9	62.6	20.6	0	0	6.72	36.57	0	11.87	521.1	1845.4	50.8	53	72	7.4	270.8	103726.4	0
p318	D3	51.2	0	38.7	182.7	9	807.7	0	37.6	23.6	77.4	0	30.8	0	21.7	61.6	32.6	0	630	25.08	632	790	81.81	496.8	1635.7	64	80.2	78	6.5	358.6	69637.66	0
p318	D4	41.2	0	44.1	282.4	0	4660.4	0	60.4	24	46.2	0	889.1	0	22.1	64.6	14.6	3	0	0	0	0	0	178.6	870	45	113.2	155.1	30.9	565.7	45394.27	1.5
p318	D5	58.6	0	67.3	399.6	0	1356	0	65.6	24.5	434.1	0	89.4	0	32.4	64.6	35	0	0	8.24	36.57	0	9.58	116.2	934.6	93	127	184.2	11.1	684.2	69087.55	1.9
p318	D6	73.1	0	84.3	351	12	1253.6	0	66.9	27.2	214.9	0	100.6	0	30.4	66.6	49.4	0	380	22.98	384	320	40.23	120.4	1126.3	101	131.5	81.9	13.7	562.2	75818.61	4
p318	D7	54.9	33.5	104.3	415.8	23	1685.8	0.6	81.2	29.8	52	15	54.3	0	29.7	71.7	73.6	3	0	9.15	234	0	36.61	93.6	498.4	112.5	167.1	228.9	11.7	506.1	61799.24	8.4
p309	Admission	116.6288	61.0084	133.5987	158.5409	72.911	921.0172	36.6983	271.8993	47.0044	63.532	107.5578	39.138	72.25	21.0177	54.79	106.6921	261.9103	1550	119	1186	10230	256	253.7202	393.4791	105.4611	409.7349	201.4025	16.8	1425.478	62441.99	33.3181
p309	Admission	105.4513	0	82.4607	64.1345	41.3215	336.3029	2.1227	141.0315	39.8735	51.9922	89.2692	26.3119	39.12	22.6161	0	22.5941	160.5886	880	53.66	943	5080	173	216.0596	461.7858	59.286	104.5853	220.418	19.1	728.4998	48789.58	0
p309	Admission	83.5342	0	98.7854	75.9022	50.7012	552.7095	14.9961	201.8646	41.6322	38.1927	96.3606	29.8777	47.87	18.2125	0	33.1365	178.4945	1530	76.8	1108	7180	238	164.3017	372.5497	62.293	141.0294	126.4577	21.5	994.5215	43299.78	5.7326
p309	D1	80.5618	0	108.9957	92.8639	62.7012	552.7095	25.2061	229.4842	42.8137	37.0466	96.3606	17.2556	52.99	18.2125	0	60.4361	191.1684	1020	103	1228	6600	213	180.0905	296.9555	68.1632	200.0995	142.0963	17.1	1162.862	49832.58	13.3636
p309	D2	130.0489	0	71.5289	49.9851	32.7689	220.4155	0	128.9904	39.291	19.5461	84.3561	27.9513	30.07	17.6023		0	142.4695	730	41.76	758	4280	142	94.9004	366.735	24.9369	92.5328	119.5316	17.4	525.5567	27830.76	0
p309	D3	206.8836	26.7106	123.6366	122.8982	64.5349	790.1595	26.4049	281.0257	44.5993	27.6865	107.5578	48.3623	68.72	17.9068	75.9434	64.6113	218.7515	7660	247	1997	11660	508	177.113	627.2979	71.0332	415.7178	152.8843	19.7	1325.859	20456.5	17.3209
p305	Admission	57.5882	0	52.9571	44.4104	0	827.7135	11.2366	88.0193	38.8451	317.5468	0	82.4657	0	30.1527	77.3958	0	113.1702	0	5.53	0	0	0	117.7415	1027.867	177.0114	118.2486	122.018	14.8	1036.107	78035.86	16.0933
p305	Admission	63.1955	0	49.9004	35.8545	0	802.4891	10.2052	86.1784	39.2186	316.703	0	58.1116	0	28.6758	77.0121	0	103.0549	0	7.65	0	70	9.4	133.4706	722.444	139.4534	96.8358	88.2266	11.6	843.0532	80711.86	15.5561
p305	Admission	46.2294	0	48.8743	34.9876	0	650.1988	9.1668	103.0336	39.2186	148.6521	0	53.438	0	27.6382	76.6291	0	107.1112	470	26.35	519	600	57.55	97.9232	458.4802	101.504	105.1126	78.5132	11.6	925.956	73329.78	14.755
p305	D1	27.7981	0	49.9004	44.4104	0	534.7364	9.1668	108.7896	38.473	158.5711	0	10.8836	0	26.4125	77.3958	0	108.1231	260	13.56	108	140	20.4	96.8826	277.5303	82.3975	90.1392	69.2261	14.1	925.956	35646.61	15.8244
p305	D2	22.1355	0	48.3598	27.0724	0	611.8433	8.3826	86.1784	38.473	142.3703	0	1.8675	0	27.2273	76.6291	0	94.899	300	16.57	174	260	28.8	64.5241	260.1153	51.7633	87.6834	70.7807	6.6	953.5331	53271.31	13.4327
p305	D3	22.0362	0	44.7309	16.081	0	827.7135	5.9935	71.7586	36.9998	202.9213	0	0	0	26.2103	76.2467	0	78.391	0	7.29	0	110	0	140.2396	350.6281	24.1136	70.3665	67.1486	7.6	891.4453	80977.15	11.6106
p305	D4	20.7072	0	40.5192	12.2436	0	285.9704	2.6613	47.1825	36.6353	59.8465	0	0	0	27.2273	75.865	0	63.6879	0	4.17	0	0	0	126.4193	419.9127	21.5629	54.0102	58.7775	8.8	606.5165	41586.88	9.3233
p305	D5	31.151	0	49.9004	62.6867	0	391.731	6.53	87.0979	38.8451	76.4337	0	5.2063	0	26.4125	77.3958	0	111.1538	430	18.83	225	490	47.04	159.9041	455.1883	58.1405	84.181	72.3325	10.7	1001.729	17122.23	15.8244
p305	D6	26.3211	0	44.7309	25.2811	0	285.9704	5.9935	79.3497	37.7334	96.6216	0	3.2904	0	25.8076	76.2467	0	82.5448	0	5.53	0	0	0	106.1069	304.0115	63.3452	66.951	56.6673	9.7	898.351	8713.586	12.1275
p305	D7	34.3868	0	50.9228	131.1229	0	470.0262	10.7217	131.4425	40.728	75.5005	16.2205	31.3013	5.21	27.0227	83.6215	0	189.8114	400	16.57	450	460	54.96	80.5502	417.6978	68.4625	105.1126	80.565	9.5	1022.36	6648.913	25.699
p298	Admission	30.3841	39.3633	75.0382	110.3197	0	1127.563	15.8142	137.4971	45.7986	77.3654	42.7701	22.3522	65.92	32.202	85.6002	18.8005	185.9583	2720	144	842	4750	290	27.8692	348.3701	93.9594	112.2091	110.4776	16.2	932.8529	230802.4	25.2694
p298	Admission	32.9806	0	67.8225	51.9648	0	827.7135	9.9463	99.7068	42.2614	69.8668	18.0314	15.3402	29.18	30.5798	82.2457	0	119.2004	2020	153	1502	5030	213	25.132	386.5534	66.4254	91.1116	71.2983	12.6	662.4214	216310.4	15.2884
p298	Admission	38.064	0	60.9648	42.7141	0	1003.214	11.2366	82.9768	42.2614	90.2626	29.9678	16.8696	7.21	29.7278	81.463	8.2655	115.1834	750	101	792	3300	77.96	26.502	448.5969	63.3452	77.9802	82.1013	11.6	543.4069	214197.8	14.4892
p298	D1	35.5813	0	64.8991	47.783	0	903.1476	11.2366	86.6379	42.2614	91.1743	29.9678	3.2904	36.99	30.5798	85.6002	0	116.1888	2020	218	1645	8510	222	31.3523	466.1515	56.0317	87.1877	73.3654	14.1	690.3116	169506.6	15.5561
p298	D4	31.7022	86.0231	102.3804	102.5351	40.0914	1860.867	26.7718	139.528	56.2507	36.3569	62.7984	3.9496	35.13	35.3146	99.0461	80.8155	185.9583	1650	223	1121	4890	171	39.1808	317.7294	112.609	127.4678	97.8696	14.3	1049.847	42371.65	26.1296
p298	D5	29.0288	84.1952	96.4778	141.0452	0	1521.011	25.2886	134.4626	54.9483	54.0106	62.7984	6.4057	27.92	34.9759	96.1388	87.28	208.023	2020	173	1124	8010	208	34.3701	327.9765	108.9337	116.1084	101.9128	17.7	987.9668	40548.68	26.4171
p298	D6	36.6722	108.435	108.6713	121.1425	40.0914	1812.529	27.7594	148.7441	58.2289	52.537	72.6297	3.9496	20.59	37.0268	101.1407	104.2781	191.7354	1730	155	1083	5710	168	42.1784	352.8845	123.4908	122.4774	101.4079	13.8	977.641	40039.94	27.5717
p298	D7	36.6722	110.8875	105.0845	119.6013	38.0058	1788.335	26.0305	144.6326	56.0327	53.0289	77.6695	6.4057	24.74	38.1853	102.1936	106.3808	201.3304	1550	124	823	4150	164	34.6713	337.0565	119.8865	119.0998	98.8812	14.5	908.7062	47084.54	26.2733
p296	Admission	66.6734	0	61.9524	172.7791	0	521.8295	10.2052	182.477	41.8758	65.123	18.0314	50.6318	0	30.1527	83.8186	8.2655	210.8856	0	42.59	464	930	4.83	42.1784	481.4542	68.4625	109.5738	130.5215	28.78382	953.5331	112930.7	33.143
p296	Admission	42.1486	0	54.9778	77.2455	0	417.9151	8.12	141.5652	39.2186	56.4531	0	16.6159	5.41	31.0093	78.5507	0	137.1355	800	91.82	754	3200	93.53	46.6641	533.5413	58.1405	106.0121	65.0654	0	884.5377	143970.8	19.3651
p296	Admission	58.9094	0	53.9691	65.9479	0	417.9151	8.12	117.5454	38.8451	67.0262	0	27.4614	0	30.366	77.3958	0	123.2056	0	45.3	205	970	27.13	51.7355	944.9077	53.9063	94.4727	57.7234	4.26	894.8984	145949.2	17.4466
p296	D1	44.4495	0	54.9778	39.3001	0	417.9151	8.12	118.5271	38.6588	79.6881	0	10.3432	0	28.4671	77.0121	0	103.0549	600	74.45	878	3490	76.53	56.4993	620.2551	47.4205	94.9478	74.3972	13.4	877.6283	119184	15.8244
p296	D2	48.8431	0	53.4635	64.3193	0	404.8344	8.6446	113.6363	38.8451	88.4355	0	4.5866	16.73	27.0227	77.3958	0	121.2045	1170	127	1132	5710	133	83.0726	559.3803	51.7633	96.8358	70.2628	11.7	901.8032	84667.53	17.0391
p296	D3	32.0897	0	50.9228	34.1185	0	312.5822	5.9935	97.8163	38.1024	21.7085	0	0	0	25.6071	76.4378	0	98.9845	340	61.17	521	1890	38.1	62.4449	255.4506	40.7444	82.1433	58.2507	8.6	770.2845	79127.94	14.2241
p296	D4	28.975	0	55.4809	78.8462	0	470.0262	8.6446	133.4543	38.8451	53.0289	0	0	20.59	26.8187	79.7113	0	147.0084	2020	91.82	682	3490	226	54.7137	229.6266	56.0317	100.5566	64.0215	9.3	932.8529	54348.18	19.918
p295	Admission	21.2949	0	53.9691	52.7969	0	483.0044	8.12	112.6634	38.8451	17.6547	0	5.8119	0	37.7203	77.0121	0	117.1934	0	4.32	0	190	0	28.3244	183.0454	58.1405	93.9964	72.3325	15.8	953.5331	331273.8	16.3628
p295	Admission	18.6418	0	51.9417	39.3001	0	312.5822	6.53	103.9883	38.1024	26.1865	0	0	0	26.8187	77.0121	0	105.0848	70	12.07	0	970	0	27.4137	158.6758	33.8316	79.0325	55.6092	14.8	725.1197	267604.4	14.755
p295	D1	19.0688	0	45.7728	21.655	0	150.5229	2.6613	56.6664	36.6353	28.1039	0	0	0	26.8187	76.2467	0	82.5448	0	3.17	0	140	0	19.1583	146.3433	13.4658	52.6744	49.2144	14.5	487.094	300443.6	10.8408
p295	D1	27.5856	0	45.7728	16.081	0	178.0082	2.0742	58.7755	36.2723	52.2908	0	0	0	26.6153	75.865	0	67.917	0	0	0	0	0	27.7174	170.9069	9.0292	47.8077	52.4223	19.9	465.9191	204860.6	10.5858
p295	D3	32.7014	0	51.9417	46.0999	0	470.0262	5.9935	97.8163	38.1024	51.3043	0	3.9496	0	26.0086	76.2467	0	98.9845	290	22.17	304	2340	28.4	37.9799	220.1606	40.7444	89.1612	72.3325	33.8	725.1197	26533.83	13.9596
p295	D4	35.4099	0	52.9571	48.6222	0	444.0116	6.53	111.6923	37.7334	63.6903	0	8.6937	4.62	26.0086	77.0121	0	105.0848	350	31.39	266	3390	31.77	66.8996	279.8433	49.6018	87.1877	72.8491	23.3	787.631	16827.86	15.8244
p294	Admission	40.417	0	55.9832	93.9127	0	444.0116	7.5929	129.4372	39.9703	27.8313	0	49.2277	28.2	27.2273	79.9053	0	141.092	210	29.5	381	2300	89.2	53.0761	698.3601	57.0881	114.8155	128.0224	11.4	843.0532	145577.8	20.1953
p294	Admission	29.6771	0	50.9228	42.7141	0	444.0116	5.453	103.9883	38.473	20.5649	0	24.7969	12.8	27.0227	76.6291	0	101.0215	130	12.5	164	1200	45.4	41.2798	484.7266	38.4693	86.1919	75.4278	11.2	711.2036	169647.3	14.4892
p294	D1	32.5342	0	48.8743	46.0999	0	325.8419	5.453	105.9033	38.1024	12.179	0	6.4057	6.5	25.6071	76.4378	0	96.9436	80	3.8	0	500	20.2	42.4778	391.0185	36.1659	74.2304	69.2261	9.2	655.4426	79915.4	13.9596
p294	D2	32.7014	0	45.7728	14.177	0	178.0082	3.2357	63.0419	36.6353	15.567	0	0	3.5	25.6071	75.865	0	72.1228	0	2.5	0	300	15.5	38.5805	307.447	21.5629	44.8691	54.5491	9	437.6329	51867.5	11.3532
p293	Admission	51.2438	0	58.4833	61.0499	0	12357.88	10.2052	119.5105	39.2186	350.7341	0	225.0814	8.07	34.9759	77.7801	0	105.0848	680	51.12	464	1230	118	30.8988	2030.625	66.4254	114.8155	123.0195	11.9	960.4232	78440.12	16.6328
p293	Admission	41.9685	0	43.16	4.0326	0	417.9151	3.2357	52.4979	36.6353	378.7715	0	185.9653	0	38.3019	75.4839	0	59.4335	0	16.12	0	330	40.97	21.0036	1835.556	16.255	50.6274	93.8168	10.9	458.8534	85580.96	9.5741
p293	Admission	26.5307	0	47.8444	30.6174	0	218.8203	5.453	77.1003	36.4536	163.2976	0	56.7098	12.42	31.0093	75.865	0	78.391	810	51.12	274	1720	114	25.4367	1037.936	29.0574	70.3665	76.4574	16.5	683.3428	90182.27	12.9085
p293	D1	30.7121	0	57.9848	166.015	0	312.5822	10.7217	154.4368	39.9703	126.8806	0	18.633	45.82	34.3022	81.2678	10.9923	152.9039	1830	158	851	5310	199	32.4096	680.5026	74.5005	118.2486	128.0224	10.5	1036.107	90588.2	29.7541
p293	D2	41.1906	0	57.9848	74.0348	0	391.731	9.687	114.6109	39.5937	106.2907	0	22.1064	0	29.3053	78.1651	0	117.1934	0	20.36	0	230	19.19	51.7355	1844.115	60.2335	111.334	161.921	14.6	994.8486	86058.5	17.992
p293	D2	33.8786	0	46.8106	23.4758	0	312.5822	3.2357	64.766	36.9998	216.0345	0	38.8939	0	35.6546	75.4839	0	72.1228	0	22.07	0	360	31.15	37.3791	2485.087	31.4633	62.2234	90.7703	13.3	546.9194	80742.3	10.8408
p293	D4	41.3697	0	56.9855	87.5979	0	701.1538	9.1668	131.4425	39.5937	59.8465	11.8446	32.2559	31.36	27.2273	79.5175	0	129.1918	1340	87.24	817	3410	205	52.0334	1975.874	62.3116	117.3947	95.8444	20.3	905.2549	75080.56	19.3651
p293	D4	63.3308	0	55.9832	44.4104	0	1053.046	5.7238	73.9745	38.1024	68.4486	0	45.9479	5.14	29.94	77.7801	0	96.9436	520	32.35	205	860	75.8	55.9042	2539.827	40.7444	74.2304	81.0773	18.2	665.9099	59331.93	13.6958
p293	D6	55.3625	0	50.9228	37.5815	0	650.1988	5.453	84.3458	37.3658	54.9896	0	32.7326	0	28.6758	76.2467	0	86.6801	0	5.61	0	140	17.98	53.8206	1378.866	36.1659	69.2393	70.2628	25.7	669.3977	77512.96	12.6475
p292	Admission	37.9474	0	56.9855	56.1126	0	777.2226	8.906	116.5655	39.5937	844.3941	22.6575	105.6734	38.36	32.0928	77.3958	0	117.1934	1510	61.27	1147	2570	196	43.0765	581.8858	61.2744	106.9079	103.9312	29.2	867.2608	121414.7	15.0214
p292	Admission	27.9045	0	821.0346	39.3001	0	675.7019	8.12	26.89971	38.8451	547.8237	0	65.3554	25.81	39.5934	76.2467	0	98.9845	990	38.94	805	1890	146	40.0807	433.1767	59.1889	97.3048	78.5132	25.5	798.0326	157896.8	14.755
p292	Admission	39.2353	0	55.9832	54.4573	0	547.6266	7.8568	117.5454	38.8451	372.3867	0	39.3657	45.23	32.202	77.3958	0	115.1834	1950	88.15	1604	3810	254	41.2798	914.3693	60.2335	106.9079	107.962	58.5	898.351	152943.5	15.5561
p292	D1	47.8424	0	56.9855	54.4573	0	521.8295	7.5929	122.4708	39.9703	555.8688	21.2198	50.8657	49.8	39.1219	78.1651	0	119.2004	2550	96.41	1848	4700	296	32.5606	1070.088	68.4625	107.3545	114.9991	44.8	891.4453	155131.1	16.9035
p292	D2	57.9177	0	50.9228	23.4758	0	417.9151	7.5929	78.4483	37.7334	22.2764	0	108.0616	25.29	28.4671	76.6291	0	90.7978	910	47.01	920	1590	140	86.4859	429.8648	193.5277	64.6139	74.3972	24.7	669.3977	119082.6	12.1275
p292	D3	61.1754	0	51.9417	41.0109	0	470.0262	10.4637	77.5491	38.1024	15.8682	0	66.5244	33.51	27.2273	76.6291	0	96.9436	900	75.99	1082	2960	135	78.1764	339.3224	225.7274	74.7727	82.1013	64.5	787.631	101357.3	14.2241
p292	D4	73.4367	0	56.4848	46.0999	0	1226.52	10.7217	94.5264	38.473	23.6854	0	88.5912	17.52	28.6758	77.0121	0	111.1538	570	50.61	470	2110	129	128.8178	282.1544	448.8226	91.1116	130.0218	62.8	953.5331	183480.5	14.755
p291	Admission	31.9	0	39.6	37.7	0	770.5	9.2	74.9	19.7	1101.1	80.3	33.6	0	51.9	36.1	8.2	107.7	0	9.25	0	790	0	47.3	708.3	29	59	52.4	6.3	414.6	59357.91	18.9
p291	Admission	29.7	0	59.9	77.1	0	318.9	13.4	125.6	22.1	142.7	81.5	9.9	0	31.1	41.1	13	154.4	0	0	0	0	0	45.9	223.6	50	91.7	60	5.8	780.2	151049.3	24.2
p291	D1	27.6	0	32.1	26	0	448.2	8.8	67.6	18.9	459.9	79.7	18.4	0	38.3	37.8	0	94	0	0	0	0	0	52	579.5	19.5	51.5	46.4	6.6	414.6	302176.2	17.6
p291	D2	22.8	0	46.1	53.7	0	448.2	10.1	94.1	20.3	284.7	78.6	9.9	0	34.6	36.1	8.2	120.8	0	0	0	0	0	60.6	431.3	22.7	73.7	47.2	5.8	572	181160.8	20.2
p291	D3	21.7	0	42.9	54.7	0	383.6	9.7	87.1	20.9	168.3	79.1	11.2	0	30.9	37.8	8.2	133.4	0	0	0	910	8.14	74.5	334.4	25.9	72.4	51.6	6.5	567.3	67001.34	20.6
p291	D4	28.7	0	69.4	130.3	20.7	415.9	15.3	157.5	23.9	105.7	82.1	20.1	0	34	48.9	32.4	202.9	0	5.5	0	760	12.15	205	433.5	70.1	105.9	79.2	6.5	934	38511.24	31.2
p291	D5	24.9	0	49.1	63.2	0	189.2	11.5	99.6	20.9	82.5	79.7	11.5	0	31.1	39.4	13	133.4	0	0	0	270	0	117.9	339.2	35.1	72.4	53.3	6.1	595.4	27818.53	21.9
p291	D6	29.9	0	36	68.8	2.2	124.1	11.5	85.5	19.7	75.5	80.3	6.4	0	30.6	37.8	10.6	130.3	0	0	0	0	0	75.6	296.7	29	68.6	37.4	5	491.5	15948.77	20.2
p290	Admission	44.6	0	67.2	69.8	0	415.9	12.5	132.5	22.1	120.6	81.8	19.1	17.05	29	42.7	22.3	165.9	630	30.11	359	1000	49.72	88.3	240.8	55.8	106.4	76	27.5	871	37270.03	25.2
p290	Admission	56.9	0	67.2	77.1	7.4	415.9	15.3	117.5	23.3	66.7	81.5	13.1	1.75	29.4	44.3	29.1	163	140	7.98	0	0	7.44	141.3	267.9	55.8	98.7	74.5	8.1	880	39914.27	24.9
p290	D1	41.8	0	36	34.6	0	156.6	9.2	67.1	19.2	57.9	79.1	7.4	0	28.7	38.6	5.6	104.3	0	0	0	0	0	144.9	140.9	5.9	57.5	46.4	5.5	341.2	27308.23	17.3
p290	D3	32	0	22.9	19	0	156.6	8.3	54.1	19.2	115	78	6	0	28.7	39.4	0	90.4	0	0	0	0	0	132.9	137.8	3.96	41.4	35.5	5.7	301.5	26971.75	16.9
p290	D4	44.4	0	64.8	68.8	7.4	1316.2	14.4	129.6	23.9	110.2	81.5	64.4	0.52	29	39.4	24.6	163	0	0	0	0	0	132.1	243.4	50	116.8	101.8	8.6	992.2	20606.95	25.2
p290	D5	35.9	0	39.6	38.8	0	383.6	9.7	73.3	20.9	80.3	79.7	8.7	0	29	39.4	10.6	111	0	0	0	0	0	181.9	201.8	12.9	63.2	48.1	6.6	477.2	20267	18.2
p290	D6	54.2	0	62.4	70.7	0	1412.4	14.8	133.7	23.3	49.6	81.5	95.5	0	29.4	41.1	20.1	151.4	0	0	0	0	0	145.6	324.8	44.1	109.9	103.2	10.2	1081.1	25355.35	24
p284	Admission	32.3	0	71.7	117.6	0	657.8	12.9	152.1	24.5	56.5	85.6	37.6	12.27	30.6	57.7	33.5	213.3	0	6.37	114	160	0	39.5	307.8	75.7	111.9	92.9	20.78	898.1	99349.1	30.2
p284	Admission	23.3	0	69.4	73.5	0	464.4	12.5	140.8	23.9	72.9	81.5	13.7	14.38	30.7	44.3	17.7	165.9	0	7.89	226	420	0	38.5	212.8	70.1	104.9	76	23.8	898.1	119187.2	26.5
p284	Admission	761.9	19.8	39.2	55.8	22.1	89.4	80.3	32.8	87.2	41.1	17.7	133.4	12.8	22.5	65.1	0	85.6	0	9.45	114	270	0	512.8	53.4	11.1	41.8342	280.5	15.27	11.5	118659	65.7
p284	D1	21.9	0	54.7	48.8	0	415.9	10.1	90.8	22.1	71.8	80.9	14	6.94	29.2	41.1	20.1	133.4	0	0.63	0	0	0	42.2	300.4	38.1	79.8	60	46.8	651.4	95930.62	20.9
p284	D2	27.3	0	57.4	60.4	0	834.8	11.5	111.3	22.7	70.1	79.1	10.2	9.08	29.2	41.1	11.8	145.5	0	1.63	0	160	0	62.5	381.4	45.6	90.1	55	8.3	771.1	44165.09	23.2
p284	D3	23.3	0	49.1	56.6	0	641.7	9.7	105.1	21.5	37.8	78.6	5.1	14.38	29.7	42.7	10.6	133.4	510	17.56	485	600	56.28	59.2	223.6	35.1	71.1	46.4	7.2	581.4	39884.51	21.5
p284	D4	27.9	0	57.4	77.1	0	657.8	12	124.4	23.3	39.1	79.7	6.9	14.9	29.4	45.8	15.4	170.1	680	33.23	713	1230	118	131.6	312.7	50	90.1	68.1	13.7	784.8	32006.07	25.3
p284	D5	35.9	0	62.4	78.9	0	641.7	12	134.9	22.7	32	78.9	30.1	12.27	29.4	46.6	15.4	161.6	0	8.67	103	180	2.95	87.6	358.1	58.7	97.7	88.3	44.6	889	17205.95	24.7
p284	D6	43.8	0	67.2	71.6	0	1956.5	12	142.5	22.1	32.6	80.3	97.7	14.9	29.4	45.8	8.2	163	0	2.22	0	130	0	58.5	444.6	52.9	98.7	88.3	34.5	871	14116.36	24.5
p284	D7	49.5	0	64.8	98.5	0	673.9	12.5	137.8	24.2	36.5	80.3	99	23.23	29.7	48.9	13	188.1	780	47.01	796	1550	122	57.5	526	55.8	99.2	71.3	28.4	771.1	22099.46	26.7
p283	Admission	38	0	78.1	96.8	22.7	995.4	16.3	135.5	29.2	131.8	83.8	17.4	0	32.3	48.9	42.2	196.2	0	0	0	0	0	42.2	293	75.7	119.2	110.5	10	1058.9	167575.6	28.5
p283	Admission	34.1	20.9	82.1	82.5	28.3	947.3	16.3	144.9	31.1	103.5	85	12.1	0	32.3	48.9	43.3	171.5	0	0	0	0	0	40.5	290.5	82.6	113.4	94.4	8.8	1023.4	153021.6	26.5
p283	D1	27	0	73.8	63.2	13.7	738.3	15.8	114.1	26.4	55.3	82.1	9.6	0	30.9	50.4	35.7	154.4	0	0	0	0	0	37.5	255.1	55.8	98.2	67.3	8.8	911.6	155631.2	24.5
p283	D2	29.7	0	54.7	34.6	2.2	512.8	11.5	81.2	22.1	40.9	80.3	9.9	0	29.4	42.7	24.6	117.6	0	0	0	0	0	52.2	300.4	25.9	69.9	63.3	9.7	539	82879.91	18.6
p283	D3	35.4	0	68.3	77.1	10.8	931.2	14.8	134.9	26.1	48.4	84.4	14	1.6	29.7	47.4	40.1	174.3	0	0.1	0	0	16.8	98.4	334.4	67.2	103.9	88.3	43.9	969.8	44063.14	25.8
p283	D4	24.3	0	52	56.6	0	480.5	11.5	97.4	21.5	57.3	80.9	10.7	0	29.4	39.4	22.3	130.3	0	0	0	0	0	311	270.4	44.1	83.3	79.2	10.6	771.1	49344.42	21.5
p283	D5	24.9	0	73.8	65.1	24.6	850.9	14.8	110.2	27.6	46.5	84.4	13.7	0	30.1	50.4	33.5	154.4	0	0	0	0	0	111.1	262.8	72.9	94.5	86.1	28.5	943	53986.73	24.2
p283	D6	26.2	0	61.2	42.9	0	609.5	12.5	80.1	22.7	22.5	81.5	16.2	0.6	29.9	44.3	24.6	124	0	0	0	0	5	53.5	199	38.1	68.6	68.1	25.7	697.6	26517.63	19.9
p283	D7	30.3	65.2	107.2	100.3	30	1316.2	22.2	119.8	32.1	51.7	90.2	15.7	0	32.5	65.9	71.6	201.5	0	0	0	0	4.3	40.9	239.5	81.2	120.6	89.9	55.5	1094.3	38574.75	27.3
p282	Admission	36.6	0	62.4	56.6	0	512.8	12	107.9	22.1	280	82.1	18.2	0	37.3	41.1	16.6	145.5	0	9.26	0	30	0	46.7	312.7	64.4	97.7	71.3	7.19	1014.5	217186.6	23.5
p282	Admission	36.1	0	64.8	69.8	0	545	13.4	131.4	23.3	271.3	82.1	20.1	5.41	33.3	41.1	21.2	165.9	670	90.84	741	3660	82.23	44.9	275.5	72.9	110.9	80.7	15.74	1067.8	230088.7	24.5
p282	D1	32	0	46.1	36.7	0	318.9	9.2	94.1	20.9	242.1	81.5	15.2	0	33	37.8	8.2	119.2	0	17.29	0	260	6.74	50.3	273	41.1	77.4	59.2	79.2	834.8	232498.2	20.2
p282	D2	32.5	0	59.9	58.5	0	673.9	11.5	109.6	22.1	107.8	80.9	25.9	0	30	41.1	20.1	139.5	0	34.34	274	860	12.16	47.9	406.5	58.7	94.5	71.3	13.297	983.3	197939.4	21.9
p282	D2	28.9	0	32.1	21.4	0	156.6	7.8	58.6	19.7	122.9	79.1	10.2	0	29	37.8	8.2	86.8	0	2.87	0	0	0	30.6	346.3	5.9	57.5	48.1	12.4	443.6	122723.9	16.9
p282	D3	34.5	0	59.9	66.1	0	415.9	12.5	117.5	22.7	40.9	80.3	18.2	0	29	41.1	17.7	154.4	0	13.83	0	50	0	51	388.3	52.9	101.8	71.3	31.2	871	71581.67	24.5
p282	D4	39.8	0	32.1	29.3	0	221.7	8.3	70.7	19.7	32	78.6	18.9	0	28.7	37.8	8.2	100.9	0	0	0	0	0	63.7	497.1	19.5	61.8	56.7	14.9	482	65448.55	17.9
p282	D5	40.6	0	36	26	0	318.9	8.8	62.1	19.2	40.9	78	27.8	0	28.7	36.1	8.2	97.5	0	0	0	0	0	59.5	413.3	35.1	61.8	53.3	38.65	520.1	56934.26	17.3
p282	D6	35.9	0	42.9	34.6	0	254.1	8.8	84.9	20.9	22.5	80.9	24.5	0	28.7	39.4	9.4	117.6	0	14.98	65.24	370	17.3	74.5	379	35.1	71.1	58.3	53.9	632.8	41799.32	19.2

p282	D7	41.1	0	64.8	90.6	0	480.5	12	127.9	23.9	41.6	80.9	30.1	0	29.4	46.6	29.1	171.5	0	1.13	0	0	0	66.3	372.1	70.1	98.7	74.5	19.5	947.5	40499.21	26.2
p281	Admission	66.2	0	67.2	67	0	866.9	13.4	132.5	23.3	201.2	80.9	170.5	0	36.5	42.7	20.1	151.4	70	0	0	0	0	46.2	843.7	67.2	99.8	159	22.3	1014.5	194053	23.2
p281	Admission	40.4	0	22.9	2.3	0	464.4	14.4	36.6	18.6	214.2	78.6	237.8	1.13	41.2	41.1	8.2	46.4	190	4.93	0	0	5.75	83	981.5	16.3	43.2	95.8	36	326.4	153105.2	14
p281	D1	49	0	42.9	34.6	0	448.2	29.2	69.2	20.9	202.7	80.3	90.7	1.13	37.8	41.1	34.6	107.7	210	6.12	0	0	9.08	321.3	800.4	61.5	61.8	103.2	31.6	706.9	232739.1	17.6
p281	D2	34.1	0	49.1	45.9	0	5100.4	23.6	59.6	25.1	213.2	82.9	95.5	0	74.5	44.3	63.3	90.4	0	0	0	0	0	2393.6	1685.2	1000.6	72.4	71.3	37	1743.1	226072.2	16.9
p281	D3	39.8	0	57.4	36.7	0	2531.8	10.6	67.6	23.9	51.7	81.5	41.9	0.52	36.3	47.4	24.6	104.3	0	0	0	0	0	1037.9	782.9	743.8	68.6	68.1	19.7	3001.7	227177.8	18.6
p281	D4	42.8	0	62.4	40.8	0	1059.6	9.7	69.7	22.7	32.6	83.5	49.3	0	35.6	47.4	25.7	111	0	0	0	0	0	664.2	518.5	311.4	69.9	55	16.7	2067.8	94553.42	18.6
p281	D5	40.8	0	62.4	44.9	0	545	10.1	91.9	22.7	43.4	81.5	23.9	0	31.1	47.4	26.9	130.3	0	0	0	0	0	357	348.7	135.1	82.1	55.8	13.1	1322.3	72633.97	20.9
p281	D6	28.1	0	59.9	54.7	0	512.8	10.4	89.2	22.7	35.9	80.9	22.3	0	29.9	48.9	24.6	127.1	0	0	0	0	0	214.2	249.9	92.3	89.5	53.3	15.9	1050	46709.92	20.9
p281	D7	24.7	0	54.7	30.3	0	448.2	9.7	63.6	20.6	39.7	81.5	24.1	0	30.1	53.4	22.3	111	0	0	0	0	0	160	240.8	44.1	68.6	41	10.6	651.4	31284.24	17.9
p280	Admission	40.9	68.7	67.2	71.6	2.2	963.3	12.9	134.3	23.3	71.8	78.3	55.2	0	34.8	41.1	11.8	151.4	0	1.95	0	0	0	72.1	587.8	61.5	104.9	200.4	8.9558	898.1	40767.72	23.9
p280	Admission	32.9	137.1	62.4	62.3	18.5	480.5	12	124.4	22.1	102.7	77.1	9	0	44.1	42.7	17.7	151.4	0	11.53	185	490	8.59	96.2	367.4	84	96.6	104.7	15.9	834.8	96022.27	22.9
p280	Admission	31.1	117.8	64.8	71.6	30	545	14.8	122.1	24.5	58.5	80.3	8.2	0	41.3	41.1	22.3	154.4	0	0	0	0	0	66.5	244.7	88.2	108.9	101.8	17.2	839.3	128744	23.2
p280	D1	34.5	49.5	64.8	75.3	13.7	577.3	12	117	22.7	27.3	78.6	5.9	0	33.8	41.1	18.9	151.4	0	34.9	429	860	18.97	68.4	276.8	89.5	110.4	88.3	9.9	925	89401.61	23.5
p280	D3	66	130.5	69.4	152.9	28.3	834.8	13.9	150.3	26.4	26.6	80.3	18.1	0	40.9	59.1	31.3	219.8	480	59.61	666	1590	57.67	72.8	402	84	121	186.2	10.6	951.9	52687.72	30.2
p280	D4	45.5	121.3	67.2	95	31.6	545	13.9	132.5	24.5	33.9	82.1	9.3	0	40.4	45.8	22.3	168.7	0	43.13	536	1200	22.28	65.4	265.4	78.5	107.9	88.3	10	825.7	38732.76	25.2
p280	D5	36	138.2	69.4	108.1	37.8	657.8	15.3	146.7	25.1	25.9	82.1	11.5	0	40.4	48.9	35.7	182.6	0	18.44	150	190	6.74	67.2	208.7	97.7	110.9	107.6	7.7	880	43728.29	26.5
p280	D6	57	80.2	64.8	134.5	30	609.5	15.3	152.1	25.1	9.6	82.1	15.7	0	35.5	53.4	29.1	209.4	0	1.13	0	0	0	55.1	204.6	82.6	115.8	97.3	12	857.4	51539.75	29.2
p280	D7	96	122.5	76	198.9	20.7	512.8	14.8	172.7	28.3	9.6	84.4	26.5	0	42	64.6	37.9	254.8	0	1.13	0	0	0	62	260.2	81.2	109.9	105.4	9.7	862	32924.68	35.6
p279	Admission	23	0	71.7	91.5	5.2	963.3	19.2	134.9	25.1	46.5	82.1	9	0	30.9	53.4	49.7	171.5	170	13.89	0	1830	22.9	22.8	249.9	70.1	120.6	94.4	6.6	943	261641.6	26.2
p279	D1	23.7	0	67.2	82.5	0	802.6	18.7	123.8	24.5	37.1	79.7	8.2	0	30.1	45.8	44.4	154.4	210	11.88	0	2100	24.25	23.2	223.6	55.8	113.9	91.4	7	916	282266	24.5
p279	D1	27.2	0	71.7	89.7	7.4	802.6	20.7	136.6	25.1	37.8	83.8	10.9	0	30.4	47.4	50.7	174.3	0	5.5	0	760	0	28.2	247.3	61.5	134.4	98.1	7.6	925	217338.4	26.2
p279	D1	31.4	0	59.9	60.4	0	706.1	15.5	97.4	22.7	40.9	82.6	9.6	0	29.7	42.7	44.4	133.4	0	4.34	0	610	0	31.6	310.2	41.1	98.7	91.4	8.2	656	147342	21.2
p279	D2	33.8	0	67.2	75.3	9.2	1043.6	19.2	117.5	24.5	28.6	80.3	7.9	0	29.9	45.8	37.9	155.8	160	6.72	0	1400	17.87	31.3	270.4	67.2	112.9	88.3	7.2	812.1	51305.53	23.9
p279	D3	39.8	0	49.1	34.6	0	866.9	16.8	70.7	21.5	19.6	82.1	5.9	0	29.9	42.7	15.4	104.3	0	3.77	0	670	3.84	33.6	251.2	29	89	66.5	8.1	395.2	25505.17	17.9
p279	D4	39.2	10	69.4	79.8	10.8	931.2	20.2	123.3	26.4	21	83.8	10.2	0	30.4	47.4	37.9	163	0	5.5	0	760	2.68	38.5	302.9	71.5	114.8	80.7	10.8	834.8	16635.73	25.5
p279	D5	41.2	20.9	76	80.7	10.8	834.8	20.2	127.3	24.8	23.2	85.6	11.2	0	30.6	48.9	42.2	163	0	0	0	380	0	42.9	279.3	82.6	124.8	86.8	15.9	898.1	13624.31	24.5
p278	Admission	40.1073	0	58.5157	103.7793	0	585.7561	8.6057	139.5646	6.1048	295.2881	66.0335	15.7443	0	7.6598	41.3617	0	156.8677	0	3.23	0	550	0	68.4495	257.8492	93.6553	79.5138	79.5887	11.335	885.5161	89322.65	18.0561
p278	Admission	42.1681	0	58.5157	61.0947	0	785.3439	8.6057	119.973	5.0906	171.837	61.758	54.6532	0	5.716	45.8862	0	131.6114	0	0	0	0	0	69.7612	292.5931	88.2733	88.1311	617.4524	11.637	983.3114	243355.8	11.7571
p278	D1	27.7196	0	46.6007	56.4045	0	498.3587	7.5284	102.8577	4.127	100.0515	60.7973	12.2443	0	4.5612	29.9416	0	110.176	0	6.1	0	380	0	57.3409	225.8086	61.1088	73.5436	49.7481	7.774	729.3359	194194.9	10.0332
p278	D2	53.8157	0	58.5157	136.3148	0	686.2214	8.9693	143.0425	5.7614	49.2342	62.7148	22.8287	0	5.481	42.8901	0	179.3972	0	8.61	0	700	0	67.794	516.3392	99.0231	95.8213	109.7746	7.05	803.5373	105532.5	19.4725
p278	D3	56.7507	0	58.5157	131.2578	0	614.6123	9.7028	140.1438	7.7118	86.6289	66.5039	22.8287	0	6.1915	51.6659	0	182.58	0	0	0	0	0	94.8502	530.6554	88.2733	89.6125	71.2321	9.686	832.2832	41262.81	22.4995
p278	D4	49.7995	0	58.5157	139.6958	0	600.2002	8.9693	130.3201	8.082	63.3029	65.5621	39.7511	0	7.1637	51.6659	0	182.58	0	0	0	0	0	149.8481	477.8614	115.0482	86.0551	92.9139	9.093	787.0841	21273.52	23.036
p278	D5	53.3003	0	66.4094	180.8181	0	671.957	10.8183	180.503	10.5936	34.7412	72.0824	40.7072	0	9.5746	68.9929	21.5954	236.5804	0	0	0	0	0	128.9369	366.8462	120.3662	84.8677	108.2836	6.192	852.7803	17002.26	32.2487
p278	D7	54.2457	0	73.9149	229.9081	0	869.3947	9.8875	233.6374	17.0017	12.4093	75.7378	39.7511	0	13.4894	85.3673	41.5654	278.894	0	0	0	0	0	85.5723	265.5062	115.0482	95.8213	162.7981	5.486	885.5161	25291.97	40.8734
p277	Admission	37.5746	0	73.9149	280.0011	113.621	1144.658	26.9802	246.3423	13.9132	44.4592	81.1345	34.8371	0	10.4953	77.6478	35.9869	294.7683	0	0	0	0	0	221.5663	225.8086	130.9699	155.5908	737.3327	5.206	729.3359	48319.92	52.9798
p277	Admission	40.1894	0	67.9389	201.7224	80.4274	757.1471	23.1715	172.2531	11.4011	69.9653	75.2835	28.0191	7.23	8.6707	76.4346	0	242.688	90	15.24	0	1300	2.68	233.9849	190.8648	115.0482	119.0143	563.0284	5.486	654.688	83713.72	42.7231
p277	D1	33.8651	0	50.1284	47.1328	81.1645	671.957	19.452	94.9286	3.5153	62.2721	63.1917	3.7473	0	4.114	31.6658	0	102.12	0	4.34	0	550	0	196.149	168.612	66.5802	104.6574	120.7266	4.376	461.4435	67595.86	10.8935
p277	D1	50.1393	0	57.7013	93.1266	89.8294	743.0126	23.8004	124.5647	5.0906	77.0745	65.0899	10.612	5.6	4.788	36.6378	0	140.4736	120	11.88	0	1100	9.17	237.7862	196.9393	109.7188	122.2258	192.0451	5.486	629.6965	72917.36	15.9421
p277	D2	42.1681	0	50.1284	57.9641	78.9455	671.957	21.0934	100.5884	3.8179	95.5954	63.6677	4.6475	8.68	3.4607	29.9416	0	109.0309	120	11.88	0	1630	16.93	225.0125	199.9351	61.1088	100.5386	130.7089	4.789	545.9533	23977.37	11.4113
p277	D3	59.186	0	44.7929	50.2064	105.6715	869.3947	23.1715	78.6296	3.8179	178.7278	63.6677	6.4856	0	3.676	29.9416	0	89.2546	0	5.5	0	1100	7.1	272.1378	217.3858	44.5468	109.0617	148.7334	4.513	367.4023	16626.56	9.0054
p272	Admission	32.2714	0	67.176	194.7309	0	585.7561	8.0644	173.4298	10.1958	147.9495	68.3768	21.9482	35.21	12.1104	65.1561	0	232.496	1590	81.79	971	4700	114	49.5482	202.9045	115.0482	93.4585	483.2819	9.984	1128.965	148501.6	33.5256
p272	D1	24.9505	0	53.5514	98.8496	0	349.2664	7.8851	117.6815	6.4533	93.6094	64.6168	14.2328	48.8	11.2973	38.2372	0	145.9691	1500	98.57	1202	6160	128	54.0887	171.8962	82.8759	83.3822	104.3106	7.629	1100.734	96930.43	16.9974
p272	D1	22.378	0	46.6007	53.2972	0	225.5398	6.8222	97.1902	4.2839	94.3546	59.8325	5.2549	27.28	8.1622	31.6658	0	103.2768	1100	54.58	877	3390	97.74	46.6376	137.0209	38.9662	60.6202	58.5091	6.05	774.7311	167765.2	10.3769
p272	D1	16.5094	0	25.4816	16.2309	0	0	4.1106	34.1625	2.6486	67.922	57.8902	1.5652	0.21	6.1915	20.6258	0	44.627	120	4.64	0	130	16.77	35.3881	0	0	36.1532	34.2922	6.05	341.52	80667.99	2.9815
p272	D2	20.8856	0	48.3785	57.1838	0	256.9665	6.4729	103.9935	4.127	8.669	60.7973	3.155	26.64	10.4953	29.9416	0	111.3193	720	64.07	625	2420	58.96	42.4457	72.2115	50.0949	60.6202	49.2626	4.376	745.8619	122240.1	11.066
p272	D4	59.3605	0	50.1284	82.5844	0	256.9665	7.8851	120.5464	4.7636	33.0955	61.758	9.4836	53.43	5.3643	38.2372	0	126.0261	1530	120	1124	5020	143	108.8342	322.9284	88.2733	76.8301	89.4534	5.909	762.3664	40264.71	13.6675
p272	D5	29.0426	0	44.7929	42.5568	0	177.5856	5.7827	73.6077	3.8179	26.4198	58.8635	3.4503	33.75	5.248	26.3711	0	86.8854	1190	66.76	1007	3830	108	82.9285	148.0095	33.3482	56.9894	57.5336	4.928	587.9127	26938.96	7.6436
p272	D6	34.0252	0	41.0779	32.8006	0	161.339	5.4418	70.2698	3.2193	12.4093	58.8635	4.1957	24.23	6.1915	26.3711	0	74.8795	790	48.42	667	2440	71.48	78.9691	166.9553	16.18	50.9106	48.7773	5.627	512.2491	23471.34	5.9573
p269	Admission	33.7852	0	60.1298	141.3891	0	1796.819	10.8183	140.7232	7.5285	592.5643	69.7726	151.2785	38.3	17.2018	55.8418	0	191.0275	2130	141	1473	7150	178	137.0157	974.3833	138.8964	84.5707	296.904	9.241	1056.284	443548.3	23.036
p269	Admission	21.1084	0	48.3785	73.7587	0	897.2437	8.9693	108.5443	5.4233	270.7203	63.1917	78.3837	33.42	8.1622	36.6378	0	121.5305	1660	124	1416	6120	150	131.2911	550.0643	82.8759	64.2398	80.0811	7.05	885.5161	486678.5	12.9712
p269	D1	18.9699	0	53.5514	51.7497	0	2707.244	10.0727	107.4055	5.0906	205.2222	60.7973	265.4115	0.52	8.4157	36.6378	0	110.176	150	9.26	106	0	6.61	146.1284	507.3001	82.8759	70.2496	85.0101	16.422	999.5541	481228.8	10.7212
p269	D1	14.2443	0	39.165	35.7776	0	1253.019	7.5284	70.2698	4.127	98.568	62.7148	43.7904	7	11.0287	29.9416	0	83.3124	990	29.06	739	1570	70.86	147.8187	942.9158	66.5802	52.1293	96.8735	9.093	828.1802	223282.7	7.3048
p269	D2	18.2408	0	32.1135	24.7463	0	585.7561	7.174	53.7093	3.2193	143.1443	64.1427	34.8371	39.28	12.6585	26.3711	0	62.5633	1970	113	1461	6350	165	229.1514	1242.243	93.6553	48.4687	88.9594	7.629	1007.67	234191.7	4.6236
p269	D3	29.9037	0	58.5157	158.4221	0	1360.545	9.335	168.7267	8.6456	56.8325	65.5621	57.4813	72.78	11.5671	52.3707	0	191.0275	2700	156	1819	10390	197	311.2956	1295.933	230.079	92.2759	159.2784	12.855	2070.026	205070	27.5311
p269	D4	36.6012	0	33.1597	27.651	0	671.957	4.4383	50.425	2.9303	38.5502	61.758	24.0686	37.98	6.9181	26.3711	0	65.0549	1810	93.33	1251	5780	137	211.9313	1286.565	77.4622	48.4687	70.2509	10.732	1859.113	199471.3	4.6236
p269	D5	33.3858	0	51.8522	51.7497	0	6348.171	7.5284	129.7438	4.7636	28.6631	64.6168	119.9661	2.36	5.716	33.3546	0	107.8839	0	2.47	0	0	0	276.3185	1213.891	77.4622	82.1929	321.5166	39.421	1464.853	98082.15	11.4113
p269	D6	21.7044	0	24.3046	17.6266	0	161.339	3.7867	36.3141	2.7885	34.1936	58.8635	16.9312	9.65	3.676	24.5148	0	45.9417	300	24.2	224	1520	38.95	151.5402	521.7284	0	41.7188	41.033	4.928	629.6965	57048.4	3.3072
p269	D7	50.0543	0	51.8522	85.0068	0	614.6123	7.5284	124.5647	5.0906	53.4397	64.6168	32.2326	6.69	4.788	47.3562	0	119.2732	620	23.51	461	640	48.43	233.294	619.1496	104.3773	85.7583	798.6078	6.192	1345.46	44283.61	12.4502
p267	Admission	65.8745	0	72.4411	219.2985	0	671.957	10.0727	172.8414	13.6989	149.3891	72.0824	39.5603	5.2	17.4945	68.9929	8.9637	258.8687	280	28.36	333	2170	30.42	118.8681	567.4519	136.2566	107.5946	407.057	7.919	1044.143	132992.6	37.3727
p267	Admission	37.0874	0	51.8522	88.2466	0	468.9181	8.2443	124.5647	6.1048	161.3526	63.6677	32.0477	4.01	6.1915	41.3617	0	141.5753	190	18.66	56.54	1560	11.17	143.4258	621.6452	104.3773	83.9766	133.208	7.484	836.3849	127751.2	16.9974
p267	D1	29.1207	0	46.6007	56.4045	0	318.7885	7.174	100.5884	4.7636	172.7883	62.7148	22.1239	8.88	6.9181	31.6658	0	100.9613	490	36.35	615	3320	45.63	150.5248	433.9728	77.4622	77.4269	71.2321	29.212	737.6016	187792.5	10.3769
p267	D1	32.2714	0	48.3785	61.0947	0	349.2664	8.9693	99.4548	4.7636	162.0687	63.6677	14.2328	12.19	5.716	33.3546	0	107.8839	560	37.39	558	4060	50.55	141.0627	402.1302	88.2733	80.7051	78.112	6.05	721.0647	115811.6	11.7571
p266	Admission	58.6628	0	75.3762	204.3501	0	785.3439	9.7028	176.3743	15.2169	144.587	74.8284	35.9624	72.11	20.1703	67.7232	0	244.7189	1950	129	1378	6640	181	92.1955	474.1227	141.5338	95.2309	684.6608	8.211	1096.697	304741.6	39.0286
p266	Admission	35.4706	0	37.2106	27.651	0	288.0339	5.4418	54.8063	3.8179	73.5279	63.1917	8.6852	34.72	6.4319	28.1781	0	69.9942	1260	67.1	1033	3850	123	92.8589	270.5425	16.18	46.633	75.1612	7.484	579.5353	338157.4	5.6225
p266	D1	39.8611	0	37.2106	40.2852	0	225.5398	5.7827	76.3954	4.127	73.5279	61.2782	4.6475	23.74	4.788	28.1781	0	84.506	770	45.68	687	2250	88.05	100.1683	265.5062	21.9682	49.6904	71.2321	4.651	545.9533	288460.1	7.3048
p266	D2	30.6111	0	56.8819	136.3148	0	468.9181	8.2443	154.6783	6.8069	129.3881	67.4421	18.6435	62.23	6.1915	50.2452	0	180.4591	2050	124	1264	7010	130	137.0157	282.9107	123.021	86.3518	88.9594	5.206	962.9861	148619.2	23.036
p266	D3	28.1855	0	44.7929	52.5229	0	379.4965	7.8851	105.13	5.0906	34.7412	63.6677	8.3676	53.1	4.3365	28.1781	0	110.176	1640	137	1117	5750	140	220.1886	148.0095	72.0307	69.9497	59.4851	3.966	869.1572	51182.87	10.3769
p266	D4	45.6657	0	53.5514	70.5726	0	7778.548	8.9693	138.4067	4.7636	16.6201	66.9734	278.233	3.59	4.9023	24.5148	0	112.4608	0	2.23	0	0	0	234.3303	384.694	93.6553	95.2309	656.803	0	1072.46	38500.96	11.7571
p266	D5	36.9252	0	48.3785	68.9844	0	379.4965	6.8222	102.2901	5.0906	18.0942	63.6677	15.5755	67.41	5.481	33.3546	0	121.5305	2120	135	1490	7890	165	338.0418	250.0613	104.3773	59.7136	76.1444	9.389	795.3132	38735.84	12.4502
p266	D6	49.5448	0	63.3034	159.2784	0	556.7675	8.6057	175.7851	8.2688	18.9723	69.3081	18.6435	63.73	7.6598	63.2052	0	195.2303	1750	121	1273	6740	143	338.3947	264.2387	154.6877	84.5707	122.7212	8.211	991.4347	32765.98	29.3407
p266	D7	62.8684	0	69.4536	222.8299	0	728.8534	8.9693	219.7947	15.6583	23.0153	79.3466	35.2116	62.07	13.4894	77.6478	16.2963	270.9092	1590	117	1264	6090	134	429.1711	300.9229	193.8552	86.9451	142.7175	14.859	1096.697	27620.63	41.2429
p264	Admission	143.3318	11.2086	73.9149	353.9542	0	671.957	9.7028	237.8655	19.538	93.1123	79.3466	57.8873	56.08	14.0489	81.2452	0	357.1621	2330	117	1619	8300	205	92.8589	402.1302	162.5548	91.0927	703.6063	26.071	991.4347	186989	68.6991
p264	Admission	89.9159	0	50.1284	57.9641	0	483.6592	7.8851	96.059	5.9324	98.8154	66.0335	12.2443	21.5	4.788	34.1866	0	110.176	1130	42.23	925	3190	103	121.5498	446.5841	141.5338	68.4495	69.27	36.446	787.0841	134333.3	12.1034
p264	D1	75.0215	0	48.3785	62.6656	0	379.4965	8.9693	102.8577	5.4233	49.7618	65.5621	19.6799	34.72	5.248	45.8862	0	117.0092	2520	96.61	1632	5850	191	78.31	377.3974	120.3662	77.4269	79.0964	30.211	877.3389	77986.08	12.4502
p264	D1	109.5372	0	47.4932	37.2747	0	409.5025	7.7065	87.0384	4.127	54.2245	63.6677	33.1594	12	4.5612	24.5148	0	82.1162	720	34.62	745	2110	87.44	84.911	652.9143	82.8759	64.8421	71.2321	24.185	712.7879	67669.8	8.664
p263	Admission	171.9745	0	55.2275	135.4708	0	193.6924	7.8851	155.8455	6.8069	23.0153	68.8429	12.5735	67.96	5.481	54.4636	0	176.206	2610	138	1555	15690	184	111.5064	451.3889	88.2733	77.4269	78.112	7.194	712.7879	46228.71	20.5383
p263	D2	88.612	0	63.3034	192.9867	0	349.2664	8.6057	187.5984	9.0268	37.4661	73.0008	20.0269	54.37	6.1915	63.858	0	206.7201	1540	105	802	9350	104	130.2819	357.2145	115.0482	82.1929	96.3783	13.622	836.3849	25885.42	28.7972
p260	D1	60.7652	0	99.2005	345.5463	198.711	1881.523	69.6939	310.9942	50.8072	142.1681	92.4054	56.8549	0	19.5527	103.7549	149.076	454.8043	0	7.91	0	60	14.44	43.633	602.0663	56.3713	244.9391	1410.08	6.946	1259.348	41373.47	93.1039
p260	D1	88.7232	0	80.0826	72.0936	184.7732	1855.301	63.0106	177.6533	24.9167	71.7635	83.8437	24.2059	0	0	0	89.9417	152.5174	480	14.44	0	220	30.5	39.9529	650.5239	56.3713	267.7688	915.438	7.296	1125.774	16905.14	19.0448
p260	D2	51.9362	0	68.0475	72.0936	171.9646	1398.479	57.4343	144.3459	24.1673	48.0375	76.0491	12.6118	4.92	0	0	51.3063	137.8942	0	12.78	0	210	29.21	43.8076	675.5233	48.0208	234.2976	218.5832	13.193	947.7764	8876.854	14.3556
p260	D5	71.2709	0	72.1149	49.2149	232.4777	2667.656	91.3708	135.2229	24.1673	89.4223	81.8919	13.8922	0	0	0	92.832	133.7124	0	1.09	0	0	0	62.7581	702.5204	44.5653	299.8582	280.8441	8.523	905.6256	12196.67	9.9664
p260	D6	48.6569	0	66.6781	76.2593	208.764	1521.642	67.1118	155.4349	21.9348	57.8447	79.2116	28.5893	0	0	0	67.4317	150.4296	0	0.63	0	50	0	54.0264	506.6487	41.0318	217.7001	235.48	5.899	888.6752	15343.78	16.4652
p258	Admission	39.943	0	74.7944	351.9981	28.5172	476.5144	16.2167	337.0363	45.1542	35.0149	79.4551	56.8549	11.27	0	100.1677	87.0419	404.318	680	213	1323	8200	92.54	50.4063	137.7819	54.7308	121.7053	984.6225	7.296	1031.182	141658.3	84.6129
p258	Admission	42.0608	0	55.4449	52.6778	16.3224	230.7196	14.9585	147.606	22.6763	27.4431	72.6497	24.767	20.71	0	0	4.8029	139.9844	1430	318	2774	13000	161	41.358	182.1615	46.3022	107.2005	60.8369	6.422	871.6713	118085.7	11.6443
p258	Admission	21.6023	0	51.0853	44.3689	3.8332	66.6488	12.4862	109.1975	19.7274	27.9126	72.6497	7.5516	0	0	0	0	123.2501	370	59.09	679	2170	59.15	42.4092	126.9546	31.7778	75.3739	37.1605	6.422	662.7905	143380.3	7.6473
p258	D1	38.9605	0	55.4449	47.1377	21.6158	192.4378	13.7147	120.9022	18.9976	27.9126	73.1349	5.0391	10.61	0	0	0	129.5288	620	191	1708	8260	103	44.8542	126.9546	43.6896	88.4616	71.3477	5.551	811.7123	161037.6	11.0899
p258	D2	25.1059	0	37.3674	2.2674	3.8332	0	10.4757	23.4115	15.3968	28.3826	72.6497	6.1972	0	0	0	0	72.8406	0	76.97	593	2530	4	22.6671	80.2839	3.8995	28.5519	0	6.771	197.7422	52388.66	0
p258	D3	61.743	0	45.1205	15.3501	35.9729	249.442	14.9585	46.8582	17.5472	20.4697	74.1058	4.9143	0	0	0	0	95.9891	170	101	1318	4790	20.82	37.484	134.2019	21.6668	71.8897	49.2092	6.422	363.9256	101758.7	0
p258	D4	83.7837	0	61.1268	67.9298	114.3466	793.256	82.8091	136.5257	23.4205	68.764	79.9422	3.0333	53.02	0	0	38.855	160.8651	1630	88.19	1437	7880	188	90.8363	215.7536	91.2405	231.6142	294.3954	4.508	807.4017	100923.7	14.8874
p258	D5	41.0704	0	48.1263	29.1556	33.3557	230.7196	17.0632	80.6203	19.7274	72.2642	74.1058	14.8727	13.84	0	0	2.0535	109.631	1160	26.58	227	1410	55.44	44.5055	211.0539	27.8618	86.8893	106.6435	7.646	344.0323	106734.8	2.0956
p258	D6	39.6619	0	46.6295	13.9711	72.441	230.7196	21.8176	40.3566	17.5472	69.2633	75.5631	0.7426	11.53	0	0	19.2486	93.8882	2850	29.88	1067	7300	115	131.0583	130.5932	34.6262	102.4541	82.0425	3.298	303.6107	58721.05	0
p258	D7	49.1648	0	49.6114	30.5376	91.7192	476.5144	28.9936	88.4106	18.2708	39.3247	77.0216	0	42.45	0	0	30.3168	119.0619	5790	89.83	2645	21090	340	228.3531	204.7393	59.6123	121.7053	122.7032	4.162	586.2086	85039.43	4.5432
p256	Admission	41.353	0	67.3637	76.2593	19.9323	823.5831	16.6392	167.8442	27.1798	605.6653	77.508	209.9546	12.94	43.4997	0	34.6142	175.4598	560	32.54	560	1020	59.6	30.3454	1584.68	54.7308	114.1185	355.8969	12.929	964.5502	334000.4	20.0615
p256	D1	25.6314	0	46.6295	18.109	0	192.4378	10.0796	52.0559	19.3621	405.2868	73.6203	135.2294	9.49	6.0881	0	12.2749	100.1893	340	20.04	279	670	36.95	38.8964	1347.48	14.9102	49.6128	112.3783	11.162	422.5164	417599.3	0
p256	D1	22.8909	0	43.5986	12.5924	0	192.4378	10.4757	36.4525	16.8269	505.9498	73.1349	93.5387	6.38	0	0	16.9603	85.4777	150	10.56	0	150	19.69	49.3687	1270.207	9.8778	41.7407	86.675	11.339	303.6107	391114.7	0
p256	D4	22.4386	0	54.0016	40.2173	0	374.7802	10.8739	122.2033	21.1962	207.7001	72.8923	11.3636	13.89	0	0	2.0535	133.7124	530	29.76	521	1240	51.32	60.3685	377.0264	35.5608	74.2235	60.8369	59.935	698.3009	70614.41	8.8204
p256	D5	20.3248	0	48.1263	26.3925	0	898.5773	10.0796	80.6203	18.2708	133.2634	73.1349	15.2033	9.18	0	0	7.3802	106.4851	370	21.43	300	830	43.55	49.7148	370.3693	21.6668	54.6619	44.0984	53.932	535.7342	86774.83	2.471
p256	D6	30.1602	0	65.3016	67.236	0	943.0515	13.3035	154.7823	24.9167	115.0654	75.0772	20.0156	22.14	0	0	32.4731	173.3759	730	43.96	667	1900	67.75	61.0518	439.3647	54.7308	119.0559	160.2593	40.881	993.7908	95508.39	18.0198
p256	D7	25.1059	0	38.9484	0	0	823.5831	7.7496	12.9251	15.3968	51.2112	71.6797	12.1399	1.44	0	0	0	66.5112	100	2.72	0	0	12.24	49.7148	337.9179	3.8995	0	54.0291	39.796	283.0435	158510.7	0
p253	Admission	78.5805	0	66.6781	64.4613	35.9729	1117.54	16.6392	144.3459	24.9167	74.7715	76.0491	59.6516	0	0	24.7351	12.2749	148.3413	0	2.19	0	0	0	51.4423	475.8296	62.804	123.0192	117.9866	24.682	964.5502	70410.67	13.5518
p253	Admission	68.121	0	68.0475	70.7055	33.3557	793.256	17.4886	146.9539	26.423	94.2593	74.5914	81.144	0	0	0	28.1438	145.2083	0	2.19	0	40	11.06	65.1429	542.9626	69.0544	120.8254	122.7032	21.82	1064.235	76143.89	14.0885
p253	Admission	88.5631	0	62.5262	54.0634	37.2479	701.0132	15.7958	139.1318	23.4205	103.4708	74.1058	43.6232	0	0	0	16.9603	135.8035	510	5.61	0	0	15.6	60.0267	597.3976	65.9503	113.6642	73.351	23.25	1031.182	75509.7	10.5308
p253	D1	117.328	0	63.9177	54.0634	38.5026	558.4497	17.9154	131.9666	24.1673	228.2929	73.1349	142.7852	0	0	0	28.1438	139.9844	0	6.37	0	0	5.95	59.6848	791.7483	53.0763	110.4572	60.8369	14	892.9178	67433.39	11.0899
p253	D2	86.6458	0	58.3033	42.9848	32.0098	152.7739	15.3764	103.3488	22.6763	141.1184	72.6497	18.2587	0	0	0	21.5077	127.4364	650	11.13	0	150	33.1	53.166	412.4218	39.2326	89.5009	29.3052	24.682	689.4527	49791.2	7.6473
p253	D5	63.0254	0	54.0016	41.6009	14.356	152.7739	12.4862	95.5536	21.9348	86.8837	72.6497	28.2009	0	0	0	12.2749	121.1562	0	3.47	0	0	7.9	103.282	250.8604	37.4095	73.0622	44.0984	11.692	563.3628	20574.21	5.1898
p253	D6	60.3899	0	61.8275	115.2261	9.9047	339.8324	14.5423	194.0272	27.1798	50.9666	75.5631	24.0196	0	0	44.0167	21.5077	225.3847	600	13.61	0	110	31.8	70.9176	247.8671	54.7308	110.9183	123.4818	13.282	977.0993	27024.62	28.6885
p253	D7	50.1102	0	73.4577	134.7699	16.3224	374.7802	14.5423	180.9255	31.7683	46.0913	76.2922	24.3926	0	0	60.327	30.3168	204.6024	460	13.61	0	60	20.41	59.8558	234.2631	54.7308	102.934	99.0571	15.232	854.6121	11809.35	30.8468
p250	Admission	187.4338	0	91.6756	334.0898	27.7938	1059.94	18.7733	295.684	59.8327	368.8629	87.264	91.9651	0	52.8464	129.4051	116.5915	460.9819	0	13.61	0	200	23.5	58.6585	2369.376	56.3713	141.1608	748.5445	32.942	1138.016	35729.74	95.7104
p250	Admission	90.8092	0	65.3016	120.8059	16.3224	574.5789	16.2167	176.3448	27.1798	235.9171	76.5353	14.8727	15.42	0	68.3998	38.855	217.0749	1400	54.51	481	3000	146	61.0518	571.5395	59.6123	124.326	203.2347	6.771	914.0811	52617.94	30.608
p250	Admission	118.5088	0	63.9177	62.3808	9.9047	409.1701	14.5423	123.5046	23.4205	132.4797	74.1058	27.0438	0	0	16.9797	7.3802	160.8651	1030	34.06	80.08	1140	91.87	54.5423	729.2488	46.3022	111.3784	143.0742	7.977	956.1693	53542.93	13.8206
p250	D1	86.9649	0	62.5262	83.901	7.2297	339.8324	14.1277	140.435	24.1673	59.326	73.6203	9.5554	9.49	0	30.6471	12.2749	169.207	1100	40.9	226	1650	112	64.462	617.1732	46.3022	109.9951	134.1768	10.281	939.3712	30447.25	17.504
p250	D2	67.1272	0	58.3033	52.6778	0	339.8324	13.7147	124.1553	24.1673	34.3	74.1058	5.0391	0	0	0	4.8029	144.1637	0	10.31	0	110	20.41	58.3162	530.9343	46.3022	105.7886	92.079	20.209	897.157	14171.87	13.8206
p250	D3	89.3642	0	58.3033	63.0743	0	509.561	14.1277	130.6644	24.9167	37.8844	73.6203	2.6012	0	0	0	0	152.5174	0	7.13	0	110	20.41	62.7581	551.339	46.3022	106.2603	92.079	12.045	880.18	7256.63	13.8206
p250	D5	76.5467	0	61.1268	68.6237	3.8332	409.1701	13.7147	131.3155	22.6763	142.6932	74.5914	6.7308	0	0	0	9.861	152.5174	480	13.61	0	190	34.4	75.6583	382.3281	49.7221	108.1363	118.7782	20.214	989.6222	27558.79	14.8874
p249	Admission	55.8	0	30.1	186.1	25	624.9	2	142.6	27.5	188.9	80.1	47.5	5.7	29	74.5	0	190.9	710	89.86	1902	3040	148	109.5	292.4	120.4	72	259.2	9.089	322.6	136915.6	26
p249	Admission	76.3	0	24.2	68.5	25.8	4800.7	0.2	93	22.7	484.2	77.3	127	0.59	38.6	57	0	72.8	1010	178	2179	3900	176	96.1	1428.5	0	65.3	31.1	15.159	354.8	281292.2	11.8
p249	Admission	30	0	24.2	44.3	16.7	360.6	0.2	60.7	17.1	617.7	73.6	37.2	0	33.4	52	0	60.5	530	69.9	1378	1750	97.49	76	736	0	59.3	31.1	6.602	220.5	332352.6	10.5
p249	D1	39.7	0	22.1	59.5	18.4	319	0	72	19.8	479.3	76	24.6	1.72	24.7	52.5	0	71	660	69.39	1569	2450	115	102.8	679.1	0	69.7	34.1	9.089	255.7	354551.7	11.3
p249	D2	29.4	0	24.2	68.5	24.2	74.9	0	73.1	22	168.5	69.9	6.8	0.4	21.7	55.9	0	78.1	600	131	1772	1420	154	127.7	551.6	0	64.5	25	5.651	244.1	173783.7	11.8
p249	D3	69	0	20	50.7	12.2	0	0	56.3	15.3	99.7	65.3	8.9	0	21.5	52.5	0	67.5	0	16.71	441	220	13.31	121.8	703.8	0	42.6	25	3.837	145	82232.81	11.5
p249	D4	38	0	22.1	45.9	14.9	74.9	0.2	59	16.5	56.7	62.9	1.1	0	21	52.5	0	62.2	310	45.3	962	590	57.3	115.6	445	0	46.5	29.6	5.651	158.2	48842.44	10.3
p249	D5	45.8	0	24.2	65.2	16.7	319	0.2	78.3	20.2	84.8	67	6.9	0	22.1	55.9	0	78.1	360	68.37	1013	960	59.83	119.8	481.6	0	53.3	38.6	4.614	220.5	41581.58	12.3
p249	D6	48.8	0	24.2	94.9	25.8	440.3	2	91.8	22.3	66.2	71.9	10.4	0	21.7	59.2	0	88.9	560	82.95	1476	1780	108	160.5	392.2	0	69.4	43.1	4.614	255.7	41523.63	13.3
p249	D7	67.8	0	20	53.9	27.8	1347.6	3.9	53	15.9	129.3	65.3	10.5	0	22.3	52.5	0	65.7	0	8.14	0	0	3	235.6	365.3	102.3	44.9	49.1	4.614	220.5	30216.68	11.3
p246	Admission	25.3	0	32	66.8	71.3	1655.1	11.6	88.3	20.2	150.1	76.4	59	35.88	25.9	57	0	79.9	1250	58.96	783	3630	110	81	312.6	52.5	77.2	119.2	8.834	272.8	296083.8	12.8
p246	Admission	20.3	0	26.2	28.7	72.3	1260.5	11.9	41.3	14.2	461.5	69.5	52.3	15.73	35.3	50.3	0	57	480	17.62	182	1310	38.28	68	420.5	0	66	65.6	6.723	322.6	235631	10.3
p246	Admission	14.6	0	24.2	36.5	77.3	728.7	11.6	48.7	16.5	617.7	69.9	16.7	20.34	27.2	50.3	0	57	550	24.53	272	1610	44.8	75.1	199.8	0	58.5	42.4	4.39	278.5	202620	10.5
p246	D1	25.4	0	47.9	320.8	105.2	1260.5	25.1	211.5	43.1	553.5	86.7	53.3	34.36	31.4	92.4	44	284.2	850	61.85	407	3260	62.02	103.7	233.6	234.9	99.2	369.8	4.167	447	117544.6	51.8
p246	D4	24.8	0	52.8	306.2	66.2	1019.7	15.3	212.1	49.8	271.4	86.3	42.4	76.8	26	92.8	15.7	282.2	1800	86.56	1229	7770	138	133.6	205.9	274.6	97.4	354.5	3.512	486.5	29187.92	46.9
p246	D5	35.8	0	49.6	260	83.9	1600.4	22.3	206.7	47.9	208.2	86.7	39.9	84.59	24.7	113	37.7	233.8	1750	88.19	1265	8530	141	151.4	260.6	257.6	97.7	197.5	4.614	476.7	29503.18	39.7
p246	D6	23.5	0	26.2	75	52	1201.6	9	87.1	19.5	67.4	67.8	8.8	85.07	21.5	55.9	0	82.6	2160	77.94	1571	10240	168	117.9	135.9	0	63.8	55.1	3.837	267.1	18943.05	11.8
p246	D7	43.3	0	32	90.8	70.9	1545.2	17.4	90	19.5	79.9	71.9	12	86.04	22.5	61.4	0	95.2	2220	81.23	1576	9000	158	156.8	277.6	80.9	70.5	70.8	3.728	267.1	18019.33	14.1
p245	Admission	202.5	0	28.2	162.5	41.5	3928.6	3.9	121.7	23.9	1729.5	99.7	76	11.23	30.9	75	52.9	140.1	990	59.04	1026	2050	160	375.8	1179.2	199.5	67.9	145.8	30.187	641.5	65620.41	23.3
p245	Admission	217.2	0	30.1	108.4	40.4	2682.7	4.9	95.4	23.9	1702.8	94.8	48.2	24.95	56.7	63.5	47.1	88.9	2010	104	1700	7190	286	458.5	1183.6	150.8	61.5	78.3	17.414	804.7	99074.83	16.8
p245	Admission	99.4	0	24.2	70.1	29	1035.2	2.5	68.6	20.2	426.7	74.4	54.7	6.62	24.3	55.9	0	67.5	660	36.37	429	1170	98.8	326.1	472.5	102.3	54	35.6	83.067	906.2	132724.4	12.1
p245	D1	32.6	0	20	36.5	31.3	516.2	3.9	44.4	13.6	117.4	65.3	2.4	1.95	23.8	49.1	0	51.7	310	13.74	79.85	310	34.07	212.6	147.1	0	45.7	32.6	4.841	939.4	127041.2	10.5
p245	D2	22	0	17.8	5.2	15.8	0	1.1	11.6	8.7	39.7	55.4	0.3	1.95	21.5	46.8	0	38	290	9.43	0	230	25.37	181.7	133.7	0	38	25	3.728	876.9	57607.2	8.9
p245	D6	23.8	0	17.8	18.1	17.6	459.6	2	20.5	9.8	19.3	56.7	2.2	1.46	21.5	48	0	43.1	320	8.91	79.85	270	39.85	298.7	98.5	0	38	31.8	5.185	505.9	15542.27	9.3
p240	Admission	17.1	0	24.2	52.3	29	297.7	1.3	64	20.2	32.2	69.5	3.3	12.56	23.5	52.5	0	64.9	340	24.69	212	3330	37.04	137.2	78.3	120.4	69.7	37.1	7.827	344.1	154872.1	10.8
p240	D2	31.3	0	24.2	54.7	25.4	319	1.5	66.9	19.5	80.3	70.7	4.6	13.2	22.7	51.4	0	65.7	380	26.79	292	4130	43.44	131.6	239.5	0	69.4	34.1	4.056	354.8	54125.46	11
p240	D3	49.3	0	24.2	61.1	25	340	1.1	69.7	22.7	79.9	70.7	4.6	20.59	22.9	55.9	0	74.6	980	41.06	788	8830	111	163.5	312.6	68	70.5	43.1	6.844	432	25718.61	12.1
p240	D4	44.4	0	24.2	93.3	28.2	571.3	1.5	88.3	23.9	76.7	68.6	5.3	33.08	23.5	63.5	0	104.3	1200	67.5	1197	9150	161	250	395.7	143.7	73.1	58.9	10.253	476.7	17035.02	14.1
p240	D5	53.7	0	30.1	122	29.8	711.7	3	109.9	27.2	78.3	74.8	11.4	30.05	24.1	71.9	0	124.4	840	46.25	384	7210	56.46	342.8	286	188.4	76.1	67.9	6.844	609.5	16297.17	17.1
p238	Admission	73.5781	0	37.4809	0	24.9742	376.62	21.0519	66.188	33.1893	44.9649	67.04	62.5263	4.41	0	0	0	66.9941	1.33	77.09	2069	5.79	148	78.3499	837.0964	44.5315	70.88	117.4995	3.7	129.9983	27976.83	0
p238	Admission	46.8903	0	37.4809	0	23.7346	339.2262	17.2743	49.8397	32.572	64.768	67.738	18.2757	23.68	0	0	0	61.1504	1.91	119	1982	11.11	191	84.4819	1146.219	14.6012	67.3989	151.4062	1.8	79.6512	25347.58	0
p238	Admission	61.4257	0	38.6485	0	17.1829	552.9557	18.2565	42.8409	32.7774	52.9009	67.04	23.7823	26.6	0	0	0	73.9385	2.15	133	2172	11.61	195	108.0454	778.8424	31.897	68.2717	110.8365	2.3	110.9946	44921.15	0
p238	Admission	72.2652	0	40.9243	0	15.0659	358.0353	17.2743	67.7563	33.1893	65.7552	68.4345	10.9118	28.52	0	0	0	66.9941	1.81	110	1924	8.77	166	108.3859	670.7863	44.5315	67.8355	85.2435	2.3	173.3571	51826.66	0
p238	D2	41.3291	0	35.0793	0	0	413.18	17.2743	51.5402	31.9585	53.3962	64.9384	1.0609	24.27	0	0	0	59.975	1.82	125	2093	10.92	175	79.0539	357.2721	14.6012	60.7943	46.9555	1.8	110.9946	38101.93	0
p238	D3	39.6575	0	37.4809	0	12.8496	413.18	18.2565	54.8911	32.3671	46.951	67.04	0.5413	4.8	0	0	0	65.8297	1.26	91.89	1978	5.86	130	68.5679	146.4392	0	59.0145	29.5801	4.4	129.9983	32899.35	0
p238	D4	52.9997	0	37.4809	0	29.757	484.2127	17.2743	64.6094	33.1893	38.0015	67.04	6.8019	5.6	0	0	0	59.975	1.3	87.86	1877	6.58	135	69.8247	376.8234	14.6012	65.209	27.8412	115.3	129.9983	13953.63	0
p237	Admission	22.2938	0	44.2066	0	0	4373.63	14.3821	36.3917	31.9585	65.7552	70.8624	4.9218	12.34	116.4289	0	0	69.3167	1110	63.34	1270	3270	166	28.0625	141.5166	44.5315	49.0649	3.532	6.3	129.9983	6056.522	0
p237	Admission	18.5287	0	39.7959	0	0	685.1338	14.5723	23.0975	32.7774	32.5151	70.5165	0.2453	4.63	0	0	0	62.3236	710	38.66	809	1600	109	23.9679	66.23925	0	42.5679	0	7.2	284.0553	38121.2	0
p237	Admission	14.6121	0	44.2066	0	0	131.8898	14.3821	10.115	31.1462	28.0147	69.1298	4.0348	0.98	0	0	0	57.6169	510	20.99	437	710	63.77	25.5834	0	0	44.905	0	3.4	455.362	71013.8	0

p237	D1	17.9991	0	45.2694	0	0	220.3111	15.529	53.2236	35.2732	57.8505	72.2429	4.1592	23.36	0	0	0	92.1512	1280	90.34	1613	5170	210	31.7741	61.0139	44.5315	59.0145	0	3.2	444.0354	44330.7	0
p237	D7	24.928	0	60.6997	120.1459	0	552.9557	17.4701	107.449	36.1179	135.3581	78.7365	13.5957	8.5	0	61.9827	0	180.9916	1140	20.99	1251	2610	162	49.5069	129.6452	135.1863	49.0649	37.8143	8.1	483.2257	3028.652	11.8582
p236	Admission	15.9317	0	39.7959	0	0	261.3104	15.1452	48.1209	35.063	43.9713	73.2751	6.2514	22.84	0	0	0	93.2765	490	41.41	476	4710	41.17	51.0359	131.377	73.5464	57.2262	0	83.7	421.0444	148925.6	0
p236	Admission	16.8444	0	37.4809	0	0	339.2262	15.1452	38.2715	32.1626	52.9009	73.9617	4.0348	29.57	0	0	0	78.531	610	63.06	453	5260	57.23	67.6679	109.6471	55.2379	56.7778	0	3.2	553.0347	1805.498	0
p236	Admission	21.6905	0	37.4809	0	7.9747	177.4219	15.1452	44.6232	32.7774	53.8915	72.9313	4.1592	24.12	0	0	0	76.2382	440	48.32	238	4440	29.3	94.01	154.4502	55.2379	56.7778	0	3.7	614.67	140086.3	0
p236	Admission	19.9137	0	37.4809	0	0	155.0547	15.1452	35.439	33.1893	61.805	69.1298	2.2795	25.73	0	0	0	78.531	520	47.63	338	5330	59.55	115.1829	138.1823	64.7905	52.7165	0	96.1	584.1528	90353.76	0
p236	D3	39.1702	0	40.9243	41.5433	0	261.3104	14.3821	85.9265	35.2732	56.8612	73.9617	10.6047	42.27	0	0	0	142.7158	1080	71.59	735	7420	79.05	82.9109	201.3644	81.7067	53.6231	0	2.3	624.7186	45575.9	0
p236	D3	29.1446	0	37.4809	0	0	131.8898	14.3821	48.1209	32.7774	37.005	71.208	1.7477	18.34	0	0	0	76.2382	380	26.79	186	3100	33.33	137.1764	209.5174	89.3986	44.4392	0	100	568.6726	24897.07	0
p233	Admission	32.3777	0	37.4809	20.8887	0	339.2262	17.2743	97.5336	35.6948	108.0056	78.0577	16.2331	13.73	0	0	0	110.0002	2040	47.04	498.84	1060	69.04	44.8531	495.5929	55.2379	64.3299	29.5801	9.3	147.9462	134346.5	0
p233	Admission	26.5372	0	37.4809	0	0	300.8549	16.3011	35.439	32.3671	90.3615	72.5873	14.0824	5.12	0	0	0	62.3236	1240	25.67	261.57	730	33.26	42.4834	469.8651	0	59.9054	67.21	3.4	90.5757	162710.7	0
p233	D1	23.3933	0	37.4809	0	0	177.4219	15.529	37.3357	32.3671	219.8219	70.5165	7.2213	1.77	0	0	0	59.975	1040	11.59	207.83	700	32.19	39.2719	1203.942	0	53.6231	0	4.8	68.0842	160041.7	0
p233	D2	23.5988	0	35.0793	0	0	2697.977	16.495	23.0975	31.9585	1301.859	78.0577	28.2506	3.04	0	0	0	59.975	700	16.16	127.84	520	18.24	67.6679	1649.866	0	50.8958	18.4583	4.7	147.9462	275774.4	0
p233	D3	20.4611	0	35.0793	0	0	586.6054	15.1452	0	28.7496	133.4076	68.4345	17.0778	0	0	0	0	48.0801	0	0	0	330	0	561.6842	501.0389	0	31.0347	0	3.7	181.4972	275269.8	0
p233	D4	25.6861	0	36.2917	0	0	220.3111	17.4701	18.4017	31.9585	82.0099	68.4345	16.0653	0	0	0	0	57.6169	0	0	0	270	0	405.52	609.2748	14.6012	44.4392	0	6.2	165.062	195983.6	0
p233	D5	21.4906	0	39.7959	0	4.0975	261.3104	17.079	41.0338	33.1893	22.9997	71.208	6.2514	0	0	0	0	57.6169	0	0	0	290	0	127.7122	362.4618	44.5315	48.1454	0	5.3	270.404	81972.53	0
p233	D6	21.3312	0	49.3843	0	26.7986	339.2262	21.4566	41.0338	35.2732	28.0147	71.8982	5.4453	0	0	0	0	66.9941	0	0	0	260	0	60.0192	316.6455	31.897	57.2262	24.2438	4.2	270.404	59716.39	0
p233	D7	17.5491	0	61.5868	0	23.7346	484.2127	22.6782	33.5059	36.5427	124.1365	77.3778	5.4453	0	0	0	0	76.2382	0	0	0	270	0	37.0247	149.6715	85.605	63.0077	29.5801	3.2	317.1257	74883.19	0
p23	Admission	86.3315	0	72.6625	85.9361	19.4913	639.9208	14.5058	110.5688	18.4915	394.0308	148.7301	28.8455	10.83	46.7552	48.7875	101.1966	169.3749	0	3.7	0	0	0	50.5896	598.0534	45.9052	346.5375	98.0388	16	844.0441	233745	29.3938
p23	D2	64.4991	0	75.9209	96.934	11.0443	511.9348	14.0062	126.2265	22.5734	237.9417	149.4459	17.2752	12.86	42.9861	57.3612	116.5967	169.3749	90	6.75	0	0	14.54	71.1198	653.0877	48.0034	376.03	100.6653	19	790.4095	49828.08	29.1057
p23	D3	61.0958	0	84.9535	147.2891	16.9455	675.8467	15.0075	157.5479	28.7152	161.2519	157.1837	28.2075	14.36	43.9429	110.4156	123.0495	269.2404	0	5.49	0	0	7.1	84.0823	724.0626	51.1236	615.4086	121.738	18	1086.851	38205.3	35.285
p23	D7	102.2361	0	86.3686	108.7492	24.1111	921.1185	14.5058	126.2265	24.2091	69.1145	163.3451	34.48	13.36	42.6691	158.0274	118.7564	237.2507	0	0	0	0	0	63.3946	315.7622	48.0034	378.759	152.5299	20	1118.498	35304.24	30.9892
p228	Admission	24.0116	0	37.4809	42.938	0	358.0353	15.1452	93.2191	34.8532	107.0267	79.0755	11.5316	34.71	0	0	0	108.8938	2300	98.4	2023	6330	314	49.5069	105.7917	31.897	57.6741	42.4678	35.5	348.9043	81161.9	0
p228	Admission	27.8309	0	39.7959	0	0	413.18	15.1452	49.8397	33.1893	99.1902	74.6473	9.5455	0	0	0	0	75.0893	270	11.03	180	70	22.45	52.933	209.5174	0	58.5682	54.1368	12.6	499.6552	103504.3	0
p228	Admission	27.3542	0	39.7959	0	0	261.3104	15.1452	51.5402	33.1893	69.7014	73.6186	3.1888	15.12	0	0	0	76.2382	1550	72.24	1466	3390	214	51.0359	250.5884	14.6012	58.5682	0	19.2	563.4779	96076.53	0
p228	D3	42.923	0	44.2066	131.1684	0	449.0156	17.079	100.3871	36.969	141.2065	80.7665	12.9527	25.96	0	40.2406	0	223.5993	1900	105	1833	4770	259	60.3874	480.036	152.111	59.0145	62.3616	14.6	499.6552	23715.16	0
p228	D4	26.8802	0	35.0793	0	0	220.3111	14.3821	39.1996	31.9585	52.9009	69.8238	0.12	10.82	0	0	0	73.9385	970	47.56	1083	1910	146	50.2727	378.8555	31.897	41.6275	18.4583	6.1	373.5393	16251.93	0
p228	D5	19.0635	0	37.4809	0	0	717.2688	14.7629	51.5402	33.1893	610.9194	76.0148	1.3461	6.08	0	0	0	80.8168	490	30.65	566	870	82.75	59.2815	308.9042	31.897	61.6811	3.532	3	568.6726	148857.3	0
p228	D6	18.681	0	37.4809	0	0	358.0353	15.1452	35.439	33.6028	200.4616	73.9617	0.5413	0.98	0	0	0	62.3236	270	8.39	43.99	120	29.24	57.8007	175.7888	14.6012	50.8958	0	4.3	664.3411	97454.44	0
p228	D7	25.264	0	36.2917	0	0	261.3104	14.3821	35.439	33.1893	57.3559	71.208	0.5413	7.96	0	0	0	69.3167	690	40.38	632	940	88.43	90.2124	230.5131	14.6012	52.7165	0	4.6	604.5608	64372.6	0
p224	Admission	110.1362	0	72.4889	71.0551	0	325.558	0	146.9118	0	28.0652	134.8053	0	14.8	0	43.2191	42.029	204.3918	350	23.29	322	2630	35.18	34.8243	492.7113	112.1829	92.126	112.9118	33.904	721.3661	54185.49	0
p224	Admission	104.1631	0	61.1642	62.215	0	234.2658	0	128.3577	0	55.0494	132.2836	0	8.76	0	38.8082	42.029	171.6789	190	12.15	142	1610	20.07	32.3733	563.2285	89.8281	100.9555	102.0194	30.703	602.4936	42980.21	0
p224	D1	66.8897	0	51.203	46.6499	0	131.6942	0	104.0312	0	35.6649	126.9512	0	12.88	0	32.6567	47.3036	97.4905	290	21.19	326	2250	31.12	53.0968	454.265	84.4531	76.9286	47.2493	12.44	705.5824	39538.13	0
p224	D2	82.1786	0	61.1642	59.7845	0	368.5994	0	133.4691	0	51.1023	134.8053	0	23.48	0	35.0043	47.3036	153.8776	660	53.13	614	5060	44.96	33.7687	347.0126	84.4531	79.9286	85.0068	15.344	853.6415	29757.11	0
p224	D3	85.4735	0	47.4587	49.9637	0	325.558	0	117.17	0	14.9573	126.9512	0	21.55	0	32.6567	42.029	115.2781	420	38.29	422	4540	32.96	43.9706	428.4892	99.5193	71.5356	50.6782	24.108	843.7232	24723.42	0
p224	D5	75.8444	0	51.203	39.1045	0	234.2658	0	104.0312	0	48.0493	126.9512	0	27.49	0	35.7755	39.399	120.7783	780	50.38	549	5190	68.1	37.7968	356.4259	84.4531	60.9394	40.1544	26.912	823.7683	27547.51	0
p223	Admission	108.6219	0	61.1642	74.2445	0	5492.939	0	146.9118	0	447.0145	137.2428	89.5826	34.35	0	40.2958	44.664	178.7732	770	68.18	645	8620	85.33	28.9112	848.6113	78.6082	80.6701	43.7448	20.731	646.6053	107493	0
p223	Admission	154.5353	0	72.4889	73.4483	0	834.8992	0	145.0598	0.8594	108.8623	139.6035	58.2573	49.82	0	50.2659	31.5415	212.1177	1130	82.46	840	7910	114	22.8082	1106.295	89.8281	84.3296	43.7448	14.758	941.2804	182458	0
p223	D1	77.5057	0	54.7094	63.8304	0	234.2658	0	136.7177	0	36.2917	144.1194	34.797	6.29	0	44.6567	36.7741	147.6545	110	7.43	0	950	10.31	27.6256	762.1089	72.1519	76.9286	0	10.451	1181.348	85356.99	0
p223	D1	117.1179	0	99.5126	71.0551	0	450.9328	0	175.5836	2.8951	78.0258	154.431	45.7566	12.88	0	50.2659	39.399	269.5958	290	23.29	135	3330	33.7	22.2431	985.317	108.1633	95.5672	24.6062	13.883	1208.047	69224.04	0
p223	D2	194.7384	0	54.7094	63.8304	0	234.2658	0	129.2877	0	22.251	134.8053	72.5962	22.36	0	59.6279	23.7419	174.537	810	42.79	629	7350	95.56	16.0647	780.2269	29.692	75.4071	0	63.031	1158.96	26509.61	0
p223	D3	256.3032	56.7382	67.0458	69.4556	125.9656	1150.76	0	118.5716	0	147.0179	125.5499	183.9735	4.8	0	31.862	119.8238	101.64	0	3.67	0	0	0	142.8061	3382.057	183.0561	149.3062	185.0245	67.893	1356.258	91985.6	0
p223	D4	217.003	75.0197	67.0458	64.6367	76.8964	1150.76	0	117.6373	0	154.6267	0	178.1844	5.69	0	31.0608	127.9932	88.7596	0	9.77	0	0	0	148.2752	3444.642	189.5814	135.0482	187.3564	61.65	1278.413	92611.5	0
p223	D5	89.335	0	86.9539	63.0232	0	257.8772	0	158.9396	0.5997	0	152.4653	5.3215	15.13	0	50.2659	42.029	247.6211	530	41.41	469	6090	64.21	34.9044	378.6181	89.8281	74.6407	10.5401	25.661	783.359	26414.63	0
p223	D6	184.9832	0	67.0458	70.2558	0	234.2658	0	121.8383	0	0	141.8938	14.9629	17.05	0	41.7657	31.5415	177.3677	670	44.87	415	5950	63.43	33.9875	603.3415	64.8565	67.5583	0	61.305	557.1899	16391.38	0
p222	Admission	39.5085	0	38.9919	27.9248	15.6833	184.9345	0	82.1136	0	51.7111	132.2836	48.9077	0	0	27.7852	36.7741	41.9835	0	1.94	0	0	0	31.0713	658.7752	56.3099	56.6146	26.6863	13.883	700.2939	136202.8	0
p222	Admission	45.9315	0	54.7094	72.6514	70.9299	450.9328	0	115.7674	0	84.782	152.4653	59.0307	0	0	40.2958	55.2483	127.8513	0	2.47	0	0	0	34.9578	457.6577	97.2087	81.4083	73.0972	55.479	833.7657	91806.6	0
p222	D1	66.4935	0	47.4587	41.6346	88.2255	280.9183	0	115.7674	0	120.3309	146.2853	46.2832	0	0	31.0608	60.5645	74.2188	0	1.45	0	0	0	43.6099	851.3068	84.4531	67.5583	73.0972	16.822	922.044	181907.9	0
p222	D1	92.7973	0	47.4587	67.8528	86.8478	209.9924	0	99.7812	0	114.8929	147.3472	54.1186	0	0	32.6567	48.6251	74.2188	0	4.89	0	0	4.83	42.9281	1229.401	84.4531	84.3296	66.9273	28.484	960.3902	150512.2	0
p222	D6	63.9126	0	47.4587	74.2445	56.5129	234.2658	0	131.1468	0	12.5768	132.2836	23.9236	0	0	29.4378	52.5959	84.1358	350	14.45	235	680	32.61	27.9522	296.2201	0	77.6838	28.7187	16.822	731.8223	131369.2	0
p221	Admission	27.8234	0	47.4587	74.2445	0	450.9328	0	116.235	0	148.1465	124.1181	14.0161	0	0	29.4378	42.029	97.4905	490	20.85	242	790	48.06	29.0994	718.2745	56.3099	79.9286	60.5848	16.822	803.6496	235156.9	0
p221	Admission	24.3333	0	51.203	69.4556	0	430.7571	0	128.3577	0	178.7281	132.2836	55.1556	0	0	32.6567	36.7741	84.1358	230	8.73	85.91	260	26.57	31.3647	937.439	45.6081	88.6228	50.6782	12.153	773.1466	188517.6	0
p221	D1	25.5387	0	54.7094	69.4556	0	529.3803	0	136.7177	0	243.3042	134.8053	42.0519	0	0	29.4378	39.399	79.3019	120	7.64	0	130	14.99	29.4725	816.0329	29.692	78.4356	63.7793	8.78	726.6007	193901.5	0
p221	D1	19.5567	0	54.7094	72.6514	0	713.4749	0	128.8227	0	172.3005	132.2836	20.7234	0	0	31.0608	44.664	84.1358	150	6.93	17.45	130	18.17	26.8262	769.095	56.3099	78.4356	36.4654	7.686	742.2271	116360.8	0
p221	D2	31.4819	0	61.1642	72.6514	0	6439.032	0	125.5657	0	30.6156	129.6689	22.1878	9.96	0	34.2276	39.399	113.4031	590	28.23	396	1900	54.62	31.1596	566.3517	56.3099	81.4083	116.9182	8.505	668.2622	29475.35	0
p221	D3	34.0374	0	38.9919	59.7845	0	5377.401	0	94.5648	0	32.5166	118.0445	0	14.08	0	27.7852	34.1549	63.0709	630	32.37	489	2660	57.08	44.2622	550.6691	89.8281	72.318	66.9273	7.958	591.286	25724.65	0
p221	D4	37.834	0	54.7094	53.2562	0	4616.647	0	86.9254	0	29.9797	129.6689	0	0	0	31.0608	39.399	74.2188	840	21.66	532	2110	66.96	48.3737	389.5178	64.8565	70.749	77.6255	8.78	504.5598	23455.38	0
p221	D5	41.9029	0	67.0458	41.6346	0	3628.766	0	78.2399	0	26.7827	141.8938	0	0	0	32.6567	79.2778	63.0709	580	13.66	389	1090	43.98	94.6997	512.3094	72.1519	68.3629	76.1249	6.079	522.3176	35802.56	0
p220	Admission	66.8897	0	54.7094	79.7973	0	4660.263	0	119.0386	0	171.741	137.2428	43.6437	0	188.1814	29.4378	51.2713	105.6671	560	19.21	387	480	66.46	0	431.9609	56.3099	95.5672	146.4753	30.385	385.8836	8624.868	0
p220	Admission	23.6732	0	47.4587	61.4059	0	450.9328	0	126.0313	0	54.7466	129.6689	20.4283	0	0	29.4378	44.664	84.1358	0	7.12	0	90	18.66	0	234.8499	29.692	78.4356	15.6411	23.181	1063.434	169442.9	0
p220	Admission	29.1201	0	47.4587	63.0232	0	450.9328	0	140.4272	0	37.5426	129.6689	25.3542	0	0	32.6567	47.3036	101.64	0	8.67	0	80	20.04	0	269.4393	0	76.9286	15.6411	18.614	1190.267	174874.6	0
p220	D1	119.9385	0	61.1642	74.2445	51.3051	567.4306	0	130.2174	0	55.3521	134.8053	30.1294	0	0	31.0608	63.228	113.4031	340	8.67	0	180	31.42	18.049	703.9315	78.6082	81.4083	70.0322	15.051	1813.245	146126.4	0
p220	D2	77.701	0	54.7094	66.2466	43.9256	567.4306	0	131.1468	0	35.0371	129.6689	23.3474	40.66	0	29.4378	47.3036	88.7596	1250	92.67	1057	4500	171	18.305	520.3899	56.3099	78.4356	18.0029	18.915	1017.007	93792.83	0
p220	D4	93.661	0	51.203	75.8347	43.9256	1112.383	0	133.9334	0	12.2337	124.1181	34.5249	46.25	0	29.4378	57.9045	101.64	920	84.37	1040	4420	172	22.4061	581.8714	64.8565	82.8751	43.7448	16.229	1100.124	37587.26	0
p220	D5	95.7694	0	61.1642	67.8528	72.4432	783.4816	0	151.5397	0	75.6658	134.8053	11.7513	52.83	0	34.2276	60.5645	127.8513	1320	90.79	1196	4700	201	24.4959	507.4375	78.6082	95.5672	47.2493	22.259	950.8508	33296.72	0
p220	D6	64.1115	0	61.1642	63.0232	54.8039	604.8065	0	153.3901	0	12.2337	126.9512	15.5873	24.52	0	34.2276	55.2483	117.1318	770	65.57	505	2020	117	25.2965	464.4138	56.3099	88.6228	40.1544	17.715	1145.465	17719.22	0
p220	D7	92.8933	0	64.1669	63.0232	84.0614	677.7792	0	143.2073	0	0.832	134.8053	26.772	37.09	0	34.2276	60.5645	156.9301	1430	83.61	1326	4360	226	28.4674	412.725	84.4531	90.0318	47.2493	18.614	1172.408	27680.64	0
p22	D7	72.1419	0	69.2501	88.3628	7.3017	675.8467	12.0288	118.7934	17.6765	87.9985	145.8443	29.4887	0	42.2479	43.7309	107.8613	148.172	0	0	0	330	0	40.3445	453.0113	40.5888	343.0862	92.7089	33	1005.654	149190.5	28.8184
p214	Admission	63.1164	0	54.7094	77.4218	93.6401	567.4306	0	126.0313	0	151.2477	134.8053	56.1909	4.76	0	34.2276	31.5415	122.573	780	48.42	798	1260	142	14.5598	595.707	29.692	90.0318	66.9273	30.067	678.9981	17186.31	0
p214	Admission	69.2621	0	54.7094	64.6367	100.2123	280.9183	0	116.235	0	148.9927	129.6689	59.8035	6.62	0	32.6567	26.3345	105.6671	660	44.4	795	1710	119	18.9519	597.2368	45.6081	82.8751	57.3398	20.427	762.888	29705.85	0
p214	Admission	30.4107	0	0	9.5456	90.9515	0	0	60.4507	0	179.5656	124.1181	48.9077	0	0	26.1	23.7419	0	210	12.65	37.17	0	14.65	32.4868	367.59	0	36.0735	4.4779	12.44	480.5166	12638.59	0
p214	D4	65.105	0	77.5824	82.1667	39.9993	510.0789	0	150.6143	0	11.1992	152.4653	27.617	2.96	0	54.3419	42.029	209.5593	480	32.36	494	720	77.07	69.7148	425.0063	108.1633	97.6041	120.8856	22.566	1398.96	4583.984	0
p206	Admission	74.4737	417.3823	186.0725	149.7803	193.6165	2060.982	22.4067	177.4334	19.5418	64.9728	188.6581	79.963	0	113.8664	60.9254	171.8429	168.7922	110	0.57	0	0	0	9.6955	686.5797	197.9151	143.1082	182.6845	13.593	545.6566	37979.26	2.3316
p206	Admission	106.9161	538.3172	233.0206	194.6172	205.1737	3025.113	28.5675	183.9099	16.804	93.8265	200.689	85.2826	0.75	148.2794	67.2857	205.0016	189.7965	530	24.95	387	560	87.48	10.4447	569.4684	206.8556	161.3889	203.4662	18.314	646.6053	100742.5	5.5607
p206	Admission	86.0538	379.4828	266.5772	178.2549	195.5614	2246.164	14.9585	171.4222	48.2013	61.9792	203.2199	57.4831	0	104.7665	100.0121	160.8392	197.8313	250	9.43	0	90	25.85	7.2699	509.0635	215.3982	176.2472	164.8574	10.17	673.6377	109814.3	9.8479
p206	D1	40.7596	253.9218	180.2506	127.0529	88.2255	1112.383	0	168.1859	29.6951	62.2791	176.8731	57.7412	2.2	49.0652	47.4882	103.5416	170.2392	560	34.94	494	920	81.81	6.1498	380.4434	123.3057	117.6296	82.0777	10.451	522.3176	1289.992	0.5797
p206	D4	90.2979	545.0514	389.6325	307.6807	365.9579	4496.185	92.5317	238.7808	105.3185	88.8748	276.9789	97.6676	29.29	166.1003	127.0053	516.8456	315.6445	870	86.32	795	3140	131	24.6801	723.983	335.7217	251.8702	375.187	7.146	1035.656	27423.12	35.6726
p206	D5	98.3514	587.3152	498.4042	397.8828	454.5529	6670.565	95.043	285.9435	153.9882	150.4023	317.7364	201.2426	18.51	148.5891	198.8224	647.7426	391.2371	1090	81.14	965	3370	144	67.957	871.4326	363.5467	329.9938	643.5439	6.343	1238.976	115213.6	41.6751
p206	D6	136.3863	748.9222	556.217	619.4408	726.7475	8714.527	181.4425	422.8104	331.2266	200.4435	403.9511	187.6958	14.01	253.7417	349.6166	1172.447	769.1988	650	51.86	536	2170	89.37	38.1462	1219.784	522.5487	445.1651	751.0047	7.146	2421.217	139483.9	103.2333
p206	D7	102.2608	604.4102	545.5699	444.3888	616.0279	7755.329	160.527	381.899	296.3906	168.942	368.1879	123.1891	37.07	186.379	258.701	878.6116	605.9533	1400	125	1231	5590	178	34.3134	1021.059	449.5329	353.7175	592.9771	7.146	1974.684	16837.4	76.5913
p204	Admission	68.4722	0	54.7094	79.0062	47.6856	4363.877	0	123.7028	0	208.4887	146.2853	231.8195	0	0	35.7755	98.1321	137.9884	370	12.11	278	290	43.7	23.0065	732.5158	133.3362	107.5085	166.0621	27.225	1072.643	184665.4	0
p204	Admission	76.5289	0	91.3124	86.8881	37.9633	2272.291	0	169.1106	4.8193	357.9506	156.3545	92.8683	0	0	60.2778	90.0361	317.7679	320	9.43	211	140	37.93	23.2421	802.3269	139.5531	110.0783	435.3379	34.549	1458.146	191507.7	0
p204	Admission	55.3999	0	34.0377	53.2562	26.7782	2272.291	0	118.5716	0	348.0967	141.8938	91.3521	0	0	31.0608	71.2388	101.64	270	8.91	110	110	24.4	19.2422	615.4823	133.3362	85.772	79.1176	25.661	902.6758	179266.1	0
p204	D1	83.9245	0	82.3889	79.0062	63.1156	8410.016	0	175.5836	2.8951	625.6681	170.482	105.4956	0	0	50.9525	92.7323	209.5593	330	12.92	242	100	26.34	34.9578	1061.432	141.057	108.7969	336.7841	71.741	1458.146	155386.1	0
p204	D4	67.0877	0	38.9919	59.7845	0	851.849	0	128.3577	0	93.2449	141.8938	82.2441	36.48	0	30.2529	92.7323	93.2033	1910	102	1667	6040	255	76.8662	1218.581	112.1829	87.203	112.9118	78.444	1002.949	89198.77	0
p204	D5	74.6697	0	38.9919	54.0763	0	1236.527	0	114.8317	0	76.5514	139.6035	76.6641	18.37	0	32.6567	92.7323	101.64	1000	65.07	1113	2980	148	63.4328	1551.335	119.7367	99.6213	126.1184	57.186	1483.31	82445.2	0
p204	D6	102.7366	0	47.4587	58.1591	0	1416.824	0	131.1468	0	74.1879	139.6035	95.3945	13.73	0	31.0608	98.1321	120.7783	910	54.44	907	2780	122	62.0686	1503.701	119.7367	99.6213	128.7117	69.989	1415.94	64981.72	0
p204	D7	95.7694	0	38.9919	61.4059	0	984.4258	0	104.9736	0	132.2963	139.6035	102.4655	20.5	0	34.2276	90.0361	124.3496	980	61.91	1100	3300	137	45.8993	987.8835	94.8278	95.5672	133.8543	45.705	1313.173	78009.84	0
p201	Admission	107.6745	0	163.0389	97.0461	144.0997	2976.533	24.3744	305.8263	52.0048	232.5493	179.1765	77.172	0	61.658	75.8782	738.9077	1842.465	0	1.45	0	0	7.92	25.1528	2080.541	229.7573	275.2608	874.073	62.34	778.2585	8575.94	41.4035
p201	Admission	113.1597	0	110.7913	66.2466	254.4227	5949.679	52.7283	163.563	0	265.8682	178.4139	86.5478	0	0	44.6567	515.401	156.9301	90	5.56	0	0	13.02	23.706	2252.067	254.4319	287.5917	463.3043	27.854	731.8223	42351.21	50.3156
p201	D4	98.0648	0	151.1134	74.2445	227.5616	6042.104	55.0896	193.1697	2.2846	2.8659	179.9339	14.3331	28.81	0	47.4882	544.3334	256.9786	850	56.88	429	3220	71.1	16.0054	820.1292	296.3737	274.8173	475.3053	8.505	823.7683	27690.5	53.5331
p201	D5	127.2479	40.5679	179.0674	86.8881	310.2453	8343.113	67.8914	254.7338	16.804	9.456	194.8137	25.0691	29.82	0	65.3988	733.0023	369.3184	700	53.15	496	3280	65.31	26.0028	1057.668	356.7674	352.0666	609.6305	8.231	1118.328	22387	76.8541
p201	D6	182.4264	46.298	197.2868	113.2796	257.9236	7108.7	63.8489	228.9647	49.088	12.2337	220.9045	38.5794	19.36	0	70.3931	665.3255	501.8936	360	39.01	160	1690	32.61	18.6565	1092.646	357.9049	254.1498	1163.11	11.298	1026.345	21764.42	53.2654
p199	Admission	40.1381	69.065	67.436	93.2368	103.8905	1207.449	20.4377	66.4796	68.9466	87.833	258.0832	53.3194	0	33.0546	71.4717	61.489	59.3101	0	1.01	0	0	0	15.7898	314.3751	153.0817	237.982	500.0519	14.602	847.4379	38445.21	12.1889
p199	Admission	62.8183	32.7694	55.4907	55.2832	56.344	612.5747	9.7655	57.2796	34.3243	69.5895	159.4274	39.089	0	18.0599	69.5006	40.9414	59.3101	0	1.94	0	0	3.29	12.9138	354.5483	115.8643	183.6246	474.7801	11.891	725.6814	35859.38	7.8723
p199	D1	39.4361	87.3188	63.4779	74.5491	76.816	859.3117	13.3739	66.4796	54.4136	58.0217	288.6564	37.3492	0	23.1312	67.4968	51.3828	67.306	0	1.01	0	0	0	15.928	307.5416	136.5477	224.5898	476.1104	23.183	937.6054	13178.49	12.0354
p199	D2	38.7383	79.7151	67.9997	86.9469	81.0126	1048.218	16.1578	82.5192	68.1277	75.4919	256.5412	53.3194	0	28.2096	73.4121	58.0886	80.5974	150	10.59	0	290	17.37	17.594	339.0856	132.5652	244.0319	436.1839	19.617	919.6436	8542.488	13.2718
p199	D3	48.3252	106.3835	73.6132	102.1369	109.0907	1481.287	28.051	101.5983	86.4217	60.4293	159.4274	64.3098	0	33.3849	84.4986	58.7662	68.637	0	0.63	0	0	0	21.25	336.8606	194.3225	306.951	542.6134	27.993	835.3451	9913.015	17.7359
p199	D4	41.7928	117.6039	78.0754	107.2697	111.669	1377.819	30.3108	108.4963	99.2006	69.5895	274.9595	59.7005	0	31.4165	93.1403	73.2683	109.7143	0	5.9	0	0	0	22.1019	367.6526	236.8951	268.0185	430.1905	29.205	1156.545	6619.27	18.0622
p198	D1	43.2896	0	55.4907	47.1109	33.5859	497.1064	8.4484	62.5215	21.242	325.2546	185.5719	65.2366	0	19.76	68.5029	35.8691	40.5757	0	0	0	280	0	32.2341	464.2729	100.769	167.0974	263.1183	42.103	601.8308	263749.4	6.5978
p198	D2	36.5574	87.3188	72.4938	103.417	83.7801	1173.705	23.3513	81.8447	72.2347	318.1921	315.7753	67.0949	1.79	28.8434	84.4986	62.1727	81.9244	0	0	0	0	0	32.2341	439.6532	150.6076	237.982	493.402	27.95	991.2887	146417.6	12.9609
p198	D3	33.9763	99.414	70.8116	93.2368	89.9263	1292.298	20.9201	86.5765	76.3725	215.8033	242.6015	52.867	1.79	29.1618	82.7103	69.7702	72.6275	0	0	0	0	0	33.7072	439.6532	196.4638	278.7105	504.0417	21.984	925.6347	107102.5	13.2718
p198	D4	50.0248	66.293	69.1257	100.859	97.9651	1292.298	22.3751	87.9329	84.7356	118.0055	209.6045	55.5883	1.79	28.3677	93.1403	59.4451	107.0734	0	0	0	0	0	41.7562	418.8413	183.4078	257.2649	522.6612	27.002	1050.601	72997.02	13.8969
p198	D5	38.5645	43.4791	75.2894	83.8241	81.0126	1377.819	19.4768	86.5765	62.4323	126.0947	211.192	51.5125	0	25.4056	78.1401	55.3907	59.3101	0	0	0	0	0	39.9546	460.1951	156.7501	252.4648	480.7662	23.858	979.3846	74214.66	11.7293
p188	Admission	39.7866	0	57.783	67.2364	26.3246	4703.918	10.2093	69.1305	21.242	408.5337	270.3721	33.0426	0	25.4056	67.4968	38.392	83.251	0	0	0	280	0	54.243	618.6107	121.581	160.6709	405.5332	21.984	651.6519	21321.59	6.4584
p188	Admission	28.8044	0	58.926	69.6629	54.8122	770.6351	9.3241	73.1242	31.3491	186.2852	239.4882	33.0426	0	20.3359	69.5006	44.8128	72.6275	0	0	0	0	0	28.7232	291.4262	112.9425	194.9273	342.6964	18.885	847.4379	174144.2	7.8723
p188	D1	31.7856	0	56.6379	57.6488	51.7135	636.0018	11.7794	62.5215	29.875	87.833	233.244	25.4621	0	18.0599	66.482	46.1153	69.9676	0	4.26	0	570	3.18	37.8617	300.6649	97.5874	176.0278	315.8321	11.891	853.4778	69224.04	8.4497
p188	D5	29.4347	88.5588	75.2894	103.417	90.6027	1550.754	22.3751	82.5192	78.8695	92.9781	201.6402	44.363	1.79	32.069	77.2078	60.8065	72.6275	110	3.67	0	840	6.33	41.1549	423.0263	148.1097	254.8665	473.4498	43.367	895.6401	27032.28	14.5264
p18	Admission	100.4623	147.9631	87.7614	180.6472	50.4218	2538.591	18.3138	176.1394	29.5356	1576.501	183.6179	205.7071	0	55.3826	86.0063	123.0495	21.6643	710	3.7	377.17	710	9.23	71.7291	2889.766	46.9562	424.9058	191.8604	25	965.1526	139081.1	41.7254
p18	D1	52.3084	121.3757	74.3096	69.2459	26.2483	1191.284	16.5239	126.2265	18.0839	1043.343	159.9397	29.1664	0	56.7997	0	105.6513	129.06	700	2.61	391.63	700	16.71	60.6704	696.0006	42.7282	414.0802	154.8215	23	893.3129	261447	27.3912
p18	D2	22.9072	58.6918	67.4784	77.524	47.3785	585.5249	19.6051	120.641	18.4915	150.6856	141.4449	20.9796	0	46.7552	0	98.9509	129.06	560	1.64	297.88	560	16.71	58.1784	327.8757	38.4309	309.6401	107.5567	24	844.0441	167024.3	27.1077
p18	D3	47.0217	73.1278	75.9209	81.1133	35.8305	799.7232	19.0873	116.0355	18.4915	628.7377	148.012	65.7177	0	55.1478	19.7867	107.8613	135.5412	700	0	501.26	700	34.32	74.1618	1631.841	43.7913	316.6612	174.3274	24	852.9947	105381.3	27.9601
p18	D4	40.0687	106.4475	74.3096	76.333	27.2828	1257.509	17.5441	133.7716	18.4915	650.7092	157.8755	49.165	0	62.3693	19.7867	112.2491	132.3154	500	0	99.04	500	0	71.2417	1422.631	44.8503	315.9605	271.874	22	924.7171	163034.4	26.5428
p18	D7	47.885	151.276	79.0452	71.008	37.5861	1107.815	20.6454	110.5688	19.3069	341.0436	169.7029	150.4079	0	62.8639	43.7309	118.7564	129.06	720	2.61	324.75	720	4.59	155.5387	2319.775	43.7913	365.7764	166.1475	26	1109.452	159670.2	27.3912
p177	Admission	105.4974	0	88.225	25.931	11.6117	7320.315	8.9297	92.953	187.4	2882.486	508.9685	598.6464	0	351.2764	71.0078	43.7357	70.918	210	0	0	380	0.28	41.3944	4706.172	150.5067	81.7788	315.8377	25	788.1413	9650.834	8.4991
p177	Admission	106.85	12.12	56.32	10.02	6.69	264.45	2.21	10.51	1.87	1126.05	6.79	110.07	N/A	40.22	4.1	2.85	2.93	220	N/A	0	490	15.56	245.67	1711.62	701.43	13.88	69.62	22.5	0	N/A	23.02
p177	D3	63.359	126.9408	117.6919	111.9367	53.4277	1532.72	31.5427	130.4452	457.0533	766.8081	613.4897	110.2263	20.98	42.0928	94.1153	98.6624	164.5543	0	75.49	0	270	4.59	84.9081	2047.646	243.9292	140.1429	234.071	25.5	1551.902	449954	17.9436
p177	D4	67.5534	93.8569	136.5444	91.7054	57.8923	3596.176	26.0622	112.5894	393.5943	724.4	655.2297	450.1256	6.37	316.2469	89.2843	94.3804	113.9672	2360	25.67	750.76	1130	74.94	157.4535	10169.78	374.3924	561.4411	1629.541	30	1732.383	610318.8	23.7188
p177	D7	100.5823	109.3012	120.3319	101.5234	79.7471	1783.462	41.752	141.163	419.8239	324.8462	667.3202	104.5774	23.82	29.7751	98.89	111.5548	97.6721	1900	54.02	280	2160	101	130.0251	1170.461	238.1466	146.0206	245.449	45	1427.774	220099.9	51.383
p175	Admission	186.8278	104.269	140.0848	98.7287	49.6316	12327.8	27.6268	125.9806	402.4781	4656.323	536.8991	937.9908	11.31	44.3968	89.2843	91.1742	100.9541	240	14.45	115	380	35.85	63.0015	5030.233	228.2854	116.8728	494.8338	31.9	974.7393	157654.4	17.9436
p175	D1	66.5942	120.6607	131.715	111.2456	40.9911	7000.567	31.5427	125.0877	472.9058	2471.734	709.0593	273.6628	15.5	45.9394	95.7129	96.5204	142.6744	390	30.71	227	860	51.34	90.0061	1700.309	266.1763	116.8728	324.3911	41.8	1012.585	398362.2	16.7183
p175	D2	100.6885	130.0294	144.705	127.7262	37.7309	2633.48	38.6066	137.59	495.9925	984.323	686.5074	124.8497	22.81	46.7127	87.6607	100.8064	148.9619	530	45.67	249	1610	50.52	175.1421	3366.932	300.6802	129.1717	335.0186	54.4	1037.688	296036.4	17.1262
p175	D3	138.3501	126.9408	149.2188	120.8852	34.394	2298.273	35.4647	142.9497	432.4938	761.5665	839.0589	110.2263	17.9	46.4548	97.3044	105.1002	133.1842	370	31.54	152	1090	39.5	233.9922	6523.151	287.2169	132.1906	385.1484	74.1	1087.602	231798.5	17.9436
p173	Admission	191.5586	237.5365	278.9151	96.0345	107.7807	4548.496	105.7552	204.6858	793.6024	129.183	1390.022	112.4817	52.52	71.8237	120.5318	214.4915	266.9588	2120	83.69	1223	6080	176	55.3908	1126.317	458.8429	167.9595	615.8525	26	1680.298	55907.56	28.3127
p173	Admission	62.4153	194.3013	166.3583	165.4953	98.8764	2674.215	61.489	194.8295	595.0816	105.8363	564.1977	71.425	23.81	44.3968	106.7333	158.2533	161.4495	680	50.24	454	2740	59.96	38.9181	521.7357	338.8614	151.3484	456.1372	28.2	1403.94	124331.7	27.0555
p173	D1	62.0388	164.9328	146.9746	141.3113	63.7104	2065.59	46.8709	150.0978	574.8616	124.2584	564.1977	74.8939	0	36.2617	87.6607	122.3481	161.4495	210	19.63	0	680	20.56	39.6266	493.7261	313.7499	136.184	363.3845	18.6	1332.049	169790.8	23.0959
p173	D2	69.6757	171.7934	160.0887	162.1543	72.1915	2267.878	51.9988	180.5035	595.0816	463.4153	733.7176	70.2662	0	35.7587	95.7129	141.8809	186.1124	140	20.44	0	790	17.37	44.9165	523.1996	323.318	140.6355	376.8861	36.5	1451.544	97375.32	24.5507
p171	Admission	123.6607	79.5223	180.4188	148.8132	0	1635.643	21.3996	115.5403	94.5232	102.4545	983.8643	90.0567	0	159.3422	53.477	89.4383	0	100	2.25	0	0	0	26.6232	580.4154	190.0635	66.0275	298.7286	30.797	889.1728	6080.178	0
p171	Admission	95.6736	357.0878	257.2704	299.1253	137.1751	6464.938	68.1681	194.0777	222.8248	844.8502	1102.53	150.5517	16.81	84.564	66.46	244.3926	237.0501	1010	54.16	823	2490	142	47.1134	1014.987	293.9916	221.6122	434.2976	29.288	1539.582	84308.05	28.9637
p171	D1	39.3366	388.9028	230.1622	318.2992	121.0244	2340.847	71.651	199.6863	242.2505	142.9865	1396.435	113.6739	11.51	61.5789	66.46	242.1878	232.2637	820	49.28	781	1550	119	29.6584	587.8571	295.0407	222.6057	379.7142	13.488	1453.846	32970.27	28.9637
p171	D2	73.2212	397.8932	235.7803	335.8074	143.2072	2719.127	70.495	197.8217	250.4445	170.3435	1102.53	100.2655	17.38	60.182	71.83	248.7932	241.7997	770	67.36	883	2250	130	32.6312	646.1043	302.2796	227.5019	402.6293	16.511	1518.295	14460.05	28.145
p171	D3	82.4827	466.7742	270.01	418.2148	175.8765	4433.427	90.6858	243.2044	300.841	178.9883	1297.071	150.5517	10.41	74.5131	75.3551	313.563	283.0834	700	45.55	614	1410	103	42.8102	901.5704	344.3095	272.2017	488.6923	12.948	1752.749	35726.1	35.8696
p171	D7	46.1917	248.843	235.7803	212.2966	0	3385.914	50.5649	185.5789	161.1899	191.3327	943.8239	132.4995	7.28	37.925	68.2615	239.9801	143.3747	660	39.81	468	1440	95.03	32.1397	408.8387	267.5202	190.269	386.3301	23.069	1231.76	29717.67	29.3685
p170	Admission	265.2662	203.5387	244.0141	253.6336	9.9516	1743.175	48.6502	192.1982	142.297	30.7953	1071.1	97.5161	32.12	18.108	59.127	167.4613	251.1937	1310	90.94	1002	10320	116	56.3999	498.8145	186.5843	231.3379	331.6269	40.678	1426.72	11936.71	3.068
p170	Admission	197.6367	379.755	279.8724	312.366	162.8861	3452.694	76.2297	218.0876	239.5024	257.527	1347.085	213.3488	0	58.3158	68.2615	287.9132	317.9392	0	0	0	0	0	58.1649	900.2896	287.6137	291.5862	434.2976	26.302	1464.651	37501.1	36.2331
p170	Admission	181.9452	296.9501	267.5002	256.2629	102.7559	5371.838	58.6364	188.4238	186.8554	527.7121	1242.074	397.1383	7.37	39.8522	60.0555	204.2138	278.6142	280	29.58	99.68	1990	19.73	101.5199	1212.224	233.8585	248.7001	381.3736	17.63	1355.389	40143.43	22.9288
p170	Admission	138.924	339.334	273.7409	339.26	141.7189	2616.514	70.495	213.5269	211.5098	434.0912	1267.567	243.686	10.02	46.5428	71.83	244.3926	383.7444	410	40.37	231	4100	34.44	178.48	721.7229	260.4529	292.3355	386.3301	21.043	1560.776	311869.4	24.2888
p170	D1	92.4132	360.9408	230.1622	346.1194	151.8884	3569.27	68.1681	218.0876	225.628	452.171	1146.833	297.2894	3.65	55.9767	60.0555	222.2054	367.7105	350	22.59	307	3290	50.3	230.0106	818.4831	258.0504	284.7566	384.6815	13.217	1623.816	54863.85	24.2888
p170	D2	74.5373	392.5172	249.3832	370.7884	153.2969	3502.7	73.9492	237.878	236.7457	148.5028	1084.614	201.5207	14.8	59.7158	68.2615	266.2836	363.6619	600	61.35	484	5260	51.07	423.3973	783.997	278.8727	299.7301	392.8899	15.402	1747.65	23524.84	25.6086
p170	D7	77.6174	425.7163	267.5002	408.6953	202.2264	3151.178	90.6858	241.4321	269.2928	124.0391	1138.026	178.844	9.39	70.8353	75.3551	322.0454	395.6098	390	38.64	178	3360	33.33	65.9998	623.0642	289.7559	286.288	394.5214	19.611	1888.76	37639.24	36.5948
p163	Admission	64.2933	283.2441	209.5704	253.6336	165.5462	10063.99	53.7101	157.3686	168.8597	1781.012	1089.104	625.5411	0	93.6309	66.46	167.4613	274.1177	0	2.81	0	0	0	63.6954	2310.344	235.2016	262.3927	412.2538	20.755	1453.846	80685.84	9.2731
p163	Admission	41.6096	183.9911	173.4127	178.9826	130.9161	5041.763	39.382	132.7881	119.1919	673.5386	1214.232	387.3364	16.94	55.9767	64.6463	139.0622	246.5138	1990	77.22	669	2080	159	56.3999	1193.041	190.0635	230.3854	306.1979	21.619	1316.47	83300.06	0
p163	Admission	34.1177	0	134.1365	105.7566	87.9952	1787.774	21.3996	115.5403	63.3083	437.5171	1220.678	316.9752	0	43.6852	59.127	89.4383	171.3273	700	30.63	217	410	56.99	53.4366	1019.952	124.7442	189.0818	217.3039	17.069	1220.322	82823.9	0
p163	D1	42.0242	387.0861	238.5499	303.9711	175.8765	2923.221	76.2297	197.8217	225.628	374.9272	1177.454	204.4936	0	67.6061	77.1024	246.5944	280.8522	0	15.28	0	0	14.44	207.5805	1979.83	298.1651	284.7566	376.3846	19.042	1560.776	76305.82	25.6086
p163	D2	49.3419	618.4114	321.2933	534.7772	319.71	4782.979	136.5976	332.8683	386.3729	311.2462	1578.356	273.8887	27.48	143.5283	115.1012	510.2645	533.1149	1210	92.24	544	2740	138	267.9515	1936.073	450.1253	385.3364	635.2688	12.41	2644.767	74183.37	65.2102
p163	D3	40.1617	460.3775	274.9757	390.4698	246.8923	3486.039	101.4583	227.1366	290.4221	212.0627	1384.157	191.0256	44.28	98.1424	89.0776	355.6706	399.536	950	114	461	3430	125	186.5623	1335.743	366.1164	329.0895	479.8307	19.611	2195.326	66645.96	41.4908
p163	D7	66.46	497.9088	239.9252	411.878	234.7081	4048.657	98.2533	246.7398	271.9568	477.4678	1009.425	181.9095	53.51	70.3746	87.3923	311.4374	434.2697	2020	148	863	5480	216	86.2365	1238.514	340.6983	332.4271	523.4297	13.488	1948.246	76246.33	41.4908
p160	Admission	130.045	273.7912	257.2704	253.6336	43.6693	2659.317	82.9749	173.102	167.3344	121.7888	1177.454	122.3408	0	42.7295	64.6463	355.6706	255.8405	0	3.04	812	0	0	210.8512	648.9619	346.1017	286.288	523.4297	86.239	1716.956	27589.11	17.4865
p160	Admission	107.4601	256.5457	249.3832	220.8854	337.5379	11671.72	71.651	136.9811	158.0907	186.5134	1077.865	132.4995	18.39	35.0195	75.3551	300.7775	251.1937	280	21.66	874	860	27.77	146.8851	738.1382	325.8677	302.6398	537.5969	44.473	1437.59	48265.65	15.83
p160	Admission	102.6828	388.9028	274.9757	341.5532	395.5289	4474.187	93.9452	198.7546	225.628	351.5032	1229.253	160.1354	29.82	61.5789	73.5978	376.4608	351.4144	790	58.13	880	2060	71.1	148.1948	1132.722	356.6753	320.9525	615.474	31.404	1701.545	69189.01	34.3969
p160	D1	74.5373	322.9174	232.9847	275.5728	327.4113	2659.317	75.0916	175.0382	200.0184	119.5337	1338.797	113.6739	21.08	37.925	68.2615	317.8082	330.6399	520	68.88	532	2090	44.39	121.5921	724.4677	306.3377	286.288	446.6538	12.142	1737.438	255829.9	21.997
p160	D4	84.7041	453.9188	321.2933	392.6315	410.9611	6591.796	116.1611	236.0962	266.622	125.1625	1229.253	138.3178	34.11	70.8353	77.1024	425.7434	417.034	640	61.85	518	2270	57.49	110.6649	754.4295	375.3384	334.4155	557.1569	15.402	2142.759	38664.78	35.1371
p156	Admission	50.0889	48.6558	88.4599	98.5803	45.2284	1416.16	18.0721	106.3164	16.0722	424.9835	511.4244	74.8853	0	112.4556	72.3559	71.3532	0	210	12.11	175	380	23.76	23.6062	481.0607	142.6635	92.2572	769.2545	8.253	1310.817	350201.8	12.9778
p156	D2	62.7871	65.4235	95.8867	106.0173	3.3246	1386.088	18.4554	101.3828	25.4999	424.2171	506.9424	54.6635	0	16.1405	69.3302	73.5742	0	110	11.35	0	230	10.66	26.7295	862.8942	138.7942	95.4437	383.4135	6.039	1246.526	124423	11.0017
p156	D3	74.0905	110.6267	97.1005	163.3396	22.5089	2236.907	27.737	103.0289	42.1878	283.2688	445.79	38.677	0	21.362	67.3272	73.5742	10.9021	0	5.56	0	0	3.29	25.4245	872.8635	173.5906	99.6722	377.7906	10.526	1153.689	71118.72	13.2245

p154	Admission	58.1589	427.4116	227.312	315.932	144.6831	2590.799	71.651	288.3333	225.628	409.0506	1355.355	402.3536	3.41	140.8841	82.2871	287.9132	351.4144	0	0	0	0	0	46.5033	875.8551	302.2796	280.1136	412.2538	12.35	3091.833	335223.9	35.1371
p154	D1	72.3451	343.3409	206.4959	258.8787	75.8402	1965.046	52.4586	247.6218	154.9726	276.5848	1138.026	359.7101	3.74	57.8485	66.46	217.7288	278.6142	60	1.85	0	70	5.38	78.1813	799.9722	258.0504	252.1953	359.5076	11.658	3632.844	261871.6	21.997
p154	D2	81.5959	387.0861	209.5704	269.2128	94.5807	2280.117	53.7101	260.7662	173.4108	256.4925	1355.355	278.0462	1.58	57.8485	70.0514	217.7288	269.5929	0	0	0	0	2.66	134.0157	778.6487	269.8316	260.7215	389.6169	9.512	4087.903	199901	21.0428
p154	D3	65.7009	388.9028	197.0435	299.1253	98.7454	2410.057	57.4161	262.5072	185.3762	144.0917	1138.026	229.3573	1.58	52.2188	71.83	213.2383	313.6634	0	0	0	0	0	108.2524	614.3384	278.8727	260.7215	392.8899	7.476	3632.844	136680.3	21.997
p154	D4	61.2723	450.6655	235.7803	382.8655	137.1751	2829.858	84.0863	271.1745	233.9801	178.9883	1205.617	226.4669	1.29	64.3657	74.4777	292.2104	355.5142	0	0	0	0	0	66.8605	590.823	331.5047	301.9149	437.4023	7.476	3174.085	78158.94	29.7705
p152	D1	53.5732	117.7235	162.4499	159.242	64.5475	8168.125	23.0643	153.361	101.7677	1107.895	1313.82	107.4828	45.94	14.4877	61.9019	122.0754	182.0114	910	65.28	1706	4170	158	33.2843	925.812	171.789	241.551	359.5076	5.753	1437.59	187557.7	0
p152	D2	37.0765	301.4039	203.3843	276.8362	143.2072	2061.834	56.1885	205.2524	182.4071	676.3759	1347.085	120.623	31.92	45.5919	64.6463	204.2138	300.7018	370	30.16	804	1010	66.05	35.8717	813.2078	243.0845	270.5917	369.6806	14.031	1838.749	186355.8	12.1548
p152	D3	35.0333	322.9174	209.5704	299.1253	140.2177	2676.419	61.0563	207.0987	202.9088	800.7205	1254.845	115.4232	38.77	43.6852	70.0514	210.9877	291.9428	610	51.25	1369	1560	94.15	163.8761	967.5188	260.4529	282.4444	359.5076	10.292	2181.026	250153	15.83
p152	D4	30.8816	278.5513	166.1693	279.3546	121.0244	4701.881	49.9291	178.8919	164.2709	680.1579	1286.563	120.623	33.52	35.0195	68.2615	190.5611	260.4553	800	39.42	1198	1970	85.65	385.2841	853.8254	243.0845	260.7215	328.0575	14.304	2743.716	125181	9.2731
p152	D5	35.0333	439.1471	230.1622	403.369	195.2957	3452.694	88.4985	244.9736	261.2592	312.2591	1483.352	158.5496	33.32	62.044	78.84	274.9651	422.8074	2030	39.42	0	1670	0	231.0638	1032.339	314.2952	309.8018	424.9183	27.491	2662.83	69445.13	28.145
p1514	Admission	54.94	3.73	7.1	0.39	0	26.54	0	0	0.25	34.91	0	13.52	0	65.28	0	0	1.1	0	7.64	0	0	0	321.51	558.92	1666.65	4.91	86.84	30.9	0	6504.2	12.79
p1514	Admission	46.23	6.78	32.73	5.72	0.47	65.82	0	0	1.26	14.96	0	10.77	8.77	7.85	0	0	3.45	130	38.65	24.94	3070	0	159.51	285.5	227.68	0	34.02	16.8	0	221330.4	8.44
p1511	Admission	56.72	21.91	69.56	34.41	8.14	170.09	4.3	19.57	4.92	82.53	30.96	12.99	0	17.68	3.91	10.41	18.54	1280	101	1012	0	144	348.47	145.62	3131.52	10.39	48.46	28.9	53.96	59136.6	18.94
p1511	Admission	60.29	16.09	68.56	35.76	4.45	117.78	2.95	13.45	4.16	91.64	26.57	12.17	5.72	12.43	2.55	7.79	13.76	1200	120	698	0	147	334.68	142.25	1411.76	6.04	41.33	23.1	60.54	79759.3	12.98
p1511	D1	57.76	18.83	79.97	40.6	7.89	177.33	4.22	26.75	5.17	48.36	21.59	9.95	10.09	19.62	6.71	10.17	21.62	1310	136	614	0	98.36	289.03	114.46	791.56	7.93	50.35	25.6	54.51	61482.9	12.98
p1511	D3	67.02	9.25	36.07	15.29	1.18	86.39	0	0	4.34	23.92	32.76	7.44	0	4.58	0	2.01	10.88	420	32.97	392	0	42.25	540.64	369.83	3308.26	4.35	49.09	13.3	85.46	17820.1	24.13
p1510	Admission	74.38	5.46	32.17	9.3	0	68.5	0	0	0.75	366.97	0	47.84	5.85	341.52	0	0	1.65	590	39.82	534	5910	99.92	305.35	1962.13	1262.16	0	52.42	41.4	0	18739.26	7.03
p1510	Admission	33.29	4.41	30.48	6.62	0.1	80.13	0.56	0	0.78	514.57	0	102.09	4.24	88.6	0	3.4	2.21	270	17.71	186	3690	57.14	160.67	898.99	823.88	0	47.4	19.6	55.61	350111.4	9.2
p1510	D1	41.58	3.29	24.14	6.02	0	39.04	0	0	0.6	767.48	0	47.76	0	16.7	0	4.52	1.93	160	10.22	36.25	1640	25.41	397.6	884.09	664.13	0	37.73	13.3	52.86	337163.8	9.85
p151	Admission	122.8507	287.8729	224.433	260.1816	192.9465	1787.774	61.0563	163.322	179.4234	163.827	1359.483	104.795	2.17	62.5088	60.9804	208.7334	217.6691	150	9.98	0	180	14.56	51.9439	862.9163	255.6231	246.9332	415.4376	54.148	1197.336	2667.357	14.6669
p151	Admission	67.546	248.843	190.5342	229.2993	173.3376	2599.374	48.6502	155.3688	158.0907	1436.576	1095.825	164.8667	6.72	43.6852	60.9804	172.1188	246.5138	280	20.35	36.75	720	29.47	50.1425	1123.013	240.4892	230.3854	294.9568	30.495	1333.195	141247.2	5.6471
p151	D1	44.7287	337.3165	203.3843	281.862	180.874	2262.736	62.2565	180.8097	185.3762	635.6413	1043.868	150.5517	9.85	48.4404	60.9804	204.2138	280.8522	380	29.45	154	770	39.27	55.8095	1043.459	266.3564	247.8183	362.9146	27.491	1410.364	65064.82	15.83
p151	D5	63.4287	362.8555	244.0141	331.1793	183.3347	2539.292	77.929	182.7216	233.9801	315.2957	1271.798	139.1431	6.04	54.1002	70.0514	239.9801	291.9428	340	15.57	304	520	38.36	84.5789	803.9501	287.6137	289.327	399.3959	10.554	1675.762	49077.38	32.5102
p1509	Admission	36.33	5.82	38.27	8.11	0	62.25	0	0	2.76	186.89	6.05	28.94	38.54	20.86	0	3.4	3.52	1860	137	1263	14910	169	226.72	686.12	688.65	1.94	36.82	19.3	44.03	151353.2	8.44
p1509	Admission	32.97	6.78	31.61	8.41	0.82	65.82	0.13	0	2.62	248.12	6.6	27.42	19.72	41.97	0	3.4	3.73	680	82.14	392	7980	40.14	240.09	485.59	427.86	0.51	39.99	15	28.93	136642	8.95
p1509	Admission	26.73	6.29	28.2	8.41	0.28	60.46	0.49	0	2.28	205.65	0	24.49	27.12	49.53	0	2.9	3.31	570	79.92	258	7300	22.13	230.73	428.26	438.47	0	37.73	15.6	56.71	124256.3	9.01
p1509	Admission	26.15	4.64	28.2	6.02	0	47.96	0	0	1.88	274.67	0	25.43	17.18	20.59	0	1.75	2.62	750	78.44	323	7060	34.67	244.42	497.4	509.88	0	38.64	15.6	44.59	136178.8	9.59
p1509	D1	18.19	3.73	27.04	6.02	0	37.25	0	0	1	103.27	0	14.37	0	6.01	0	3.65	2.9	530	43.94	395	5240	52.34	163.69	343.58	257.57	0.8	29.69	10.4	63.27	261461.4	7.22
p1509	D2	30	6.29	44.23	11.7	2.23	69.39	0.13	4.26	2.42	43.19	5.47	11.02	22.13	9.65	0	5.25	4.42	750	92.81	449	7930	47.3	245.95	311.21	183.02	1.74	35.66	7.1	103.13	118045.7	10.61
p1509	D2	23.92	3.07	5.66	1.42	0	64.03	0	0	0.36	30.04	0	77.87	0	2.57	0	1.88	0.26	0	0	0	0	0	517.12	747.64	1268.38	8.6	73.08	29.5	103.13	77621.3	14.52
p1509	D2	42.49	17.45	93.07	26.42	9.64	197.3	6.23	28.36	7.57	15.64	28.16	14.53	32.44	28.06	10.09	12.64	11.97	890	127	527	10270	56.53	220.48	170.86	177.09	10.88	60.66	7.1	69.25	69031.4	20.1
p1509	D3	48.02	13.27	79.48	15.89	6.81	139.38	3.98	13.93	4.23	11.57	21.31	12.34	26.8	16.42	4.69	8.51	8.2	710	93.17	386	7660	45.61	271.66	206.59	313.09	7.11	56.69	16.2	51.21	51290.1	17.79
p1508	Admission	37.2	7.02	56.33	6.17	2.91	108.8	2.79	11.55	2.28	31.48	11.96	14.94	0	27.12	2.55	6.35	4.62	350	21.36	606	2290	117	382.64	249.21	1589.92	4.91	25.67	15.6	31.18	31041	13.24
p1508	Admission	33.29	10.78	63.51	14.39	3.77	101.62	4.14	19.1	4.23	22.92	19.15	13.8	8.34	17.26	4.17	7.79	7.93	750	41.67	969	4870	188	347.52	264.79	1021.55	7.8	36.36	19.3	45.69	51323	14.91
p1508	Admission	40.52	17.73	96.42	27.32	8.48	186.4	6.56	30.66	7.46	27.48	25.44	15.99	24.86	29.94	6.71	13.7	13.89	1240	70.26	1245	9190	215	316.46	252.08	449.09	12.21	44.4	19.3	68.71	58880.2	19.46
p1508	Admission	34.53	9.76	67.05	15.29	4.96	115.99	4.14	22.82	5.68	31.92	19.74	14.05	12.5	24.01	3.37	9.1	8.13	820	59.03	1017	6090	171	389.66	288.14	174.12	7.11	35.2	17.4	73.05	46418	15.23
p1508	D1	26.35	4.53	19.36	2.16	2.4	153.82	2.4	11.55	2.69	71.87	0	189.91	0	15.85	1.7	8.03	0.54	220	11.31	421	370	117	795.56	923.19	2007.45	4.19	35.43	12.7	95.12	32857.7	18.17
p1504	Admission	46.6	4.88	25.89	6.92	1.18	63.14	0	0	2.21	56.2	5.47	8.11	11.89	32.74	0	0	3.8	430	56.78	296	0	29.63	368.9	174.69	580.01	0	44.4	16.5	0	29153	8.76
p1504	Admission	34.99	7.02	38.27	9.3	1.97	62.25	0.71	0	2.15	39.61	4.53	6.59	19.4	11.85	0.79	1.88	3.87	660	89.14	392	0	44.77	377.72	104.55	765.41	0.17	38.42	12.4	0	72486.4	8.82
p1504	Admission	41.32	9.76	53.22	11.4	3.68	87.29	0.94	4.26	2.55	33.86	9.93	8.19	16.55	12.43	1.55	4.89	5.24	390	65.41	299	0	18.02	328.7	133.91	748.51	2.34	49.51	14.7	0	75489.6	11.9
p1502	Admission	28.23	4.19	13.77	26.11	0	31.89	0.41	0	10.43	9.53	0	6.54	8.34	0	0	0	10.4	380	49.24	258	3690	41.4	195.79	154.93	187.48	0.17	29.69	9.5	0	45202.8	4.98
p1502	Admission	31.68	4.88	18.14	14.09	0.47	31.89	0.78	0	4.92	7.94	0	6.93	0	0	0	0	6.35	100	24.71	0	1820	0	301.1	216.49	112.16	1.63	32.84	10.1	0	32951.9	6.32
p1502	Admission	18.19	0.47	0	5.43	0	60.46	0	0	2.01	0.26	0	49.06	0	28.0336	0	0	2.48	0	15.53	0	0	0	266.81	198.09	920.875	0	38.19	21.8	0	33151	7.54
p1501	Admission	44.89	14.74	56.33	17.84	9.31	128.57	6.23	13.45	2.49	42.2	6.6	12.74	62.41	21.41	8.18	10.17	6.96	1180	140	894	13280	110	173.2	223.08	226.19	6.83	28.7	23.4	15.18	92285.45	13.18
p1501	D1	143.03	3.96	12.48	9.3	7.81	39.04	5.42	21.43	1.03	157.32	0	39.98	60.23	29.27	6.34	8.51	2.41	2150	177	1424	17330	240	543.85	2462.48	803.87	6.47	37.5	91.1	15.76	70119.39	13.88
p143	Admission	56.555	374.1861	224.433	323.0096	149.0397	3469.37	71.651	210.7783	232.5939	830.7702	1271.798	200.0301	5.71	164.5959	78.84	208.7334	341.086	180	14.23	0	140	38.36	32.3037	977.5592	320.1333	272.2017	467.8951	27.193	1613.364	230331.4	25.6086
p143	D1	48.4999	385.2631	235.7803	313.5567	134.0759	3201.615	68.1681	219.9049	217.1882	740.0637	1194.815	172.67	7.75	81.8314	71.83	204.2138	351.4144	320	18.97	192	370	55.15	44.9715	1032.339	300.2296	270.5917	391.2551	21.33	1518.295	180817.4	23.387
p143	D6	76.7361	445.7546	264.9718	403.369	208.9944	3369.199	100.3924	260.7662	255.8671	219.9738	1324.248	187.9999	3.41	73.5949	87.3923	302.9137	387.7143	200	9.98	144	160	69.59	82.5006	881.0152	323.9674	315.4224	534.7769	4.797	1853.796	104646.8	36.5948
p1415	Admission	55.25	6.29	38.27	7.51	2.91	71.18	2.09	5.25	1.39	37.45	0	20.42	8.27	77.09	3.1	1.36	4.56	840	75.79	627	8350	93.83	299.28	321.43	417.25	1.05	28.45	28.9	0	22816.23	6
p1415	Admission	46.11	8.25	42.08	8.11	3.6	66.71	2.09	2.75	1.32	23.03	0	13.97	13.96	8.91	2.55	4.64	4.83	1550	112	1188	14610	150	221.26	227.17	421.8	1.41	24.11	18.1	0	118615.8	6.58
p1415	D1	38.61	8.5	44.77	9.9	4.28	98.04	2.87	6.73	2.28	157.38	8.59	24.05	21.66	28.06	4.04	5.5	5.31	1440	128	1081	13230	143	189.67	336.67	206.81	3.17	35.89	15	0	106486.7	8.63
p1413	Admission	115.81	5.11	22.36	5.13	2.05	40.82	1.62	0	0.84	5.18	0	4.21	6.52	24.68	0	1.1	2	1440	122	1039	11680	131	559.92	311.29	1815.66	3.78	68.96	20.9	0	23427.9	7.67
p1413	Admission	71.39	9.76	39.37	9.9	4.11	72.97	2.01	0	1.42	7.82	0	6.33	16.55	12.14	1.4	2.65	5.8	740	111	608	8840	50.66	362.31	187.41	817.72	1.18	41.33	12.1	0	81130.3	6
p1413	Admission	70.67	3.96	19.97	4.53	0.28	33.68	0.13	0	0.66	16.42	0	6.63	0	14.15	0	0	1.65	270	30.71	0	3570	0	309.58	237.7	843.91	0	26.69	8.7	0	180172.9	4.27
p1413	Admission	79.08	5.93	27.04	6.32	2.4	56.89	1.78	0	1.16	53.08	0	15.99	0	24.41	0	3.9	3.04	270	43.19	0	4190	0	481.73	491.79	1074.22	0.51	49.09	7.3	0	99734.3	8.63
p1413	D1	74.21	8	30.48	7.51	2.4	69.39	2.4	0.12	1.42	36.24	0	5.9	8.05	15.57	0.62	4.15	3.45	610	77.7	340	7930	24.62	587.92	410.67	84.39	0.17	49.09	7.6	0	91040.6	7.41
p1413	D2	102.35	3.07	15.04	2.16	0.28	30.11	0.86	0	0.78	17.49	0	7.56	0	15.57	0	2.78	2.28	0	2.19	0	0	0	1208.92	1001.72	2528.39	8.47	71.96	19.3	0	69677.9	13.82
p1413	D3	87.47	4.19	18.76	4.68	0.28	54.21	0.56	0	1.29	2.58	0	4.87	0	5.54	0	0	2.07	420	46.97	138	4870	9.29	859.77	433.03	2229.67	1.29	46.76	25	0	41642.8	8.24
p1413	D4	124.43	1.5	0	1.57	0.37	51.53	0.56	0	1.03	1.52	0	44.56	0	13.87	0	1.88	1.31	90	0	0	0	0	1458.8	1061.05	3403.89	6.83	93.47	16.2	0	45842.5	19.2
p1413	D5	99.33	2.64	13.13	4.53	0	31.89	0.71	0	0.91	1.7	0	4.39	0	7.4	0	0	2.07	0	6.06	0	630	0	784.3	274.87	168.2	1.94	60.07	13	0	57967.1	7.28
p1412	Admission	106.11	7.14	29.34	7.21	0.74	55.1	0	0	1	58.44	0	19.63	0	26.85	0	1.88	4	0	8.69	0	930	0	158.64	527.14	607.5	8.6	18.07	56.2	53.96	7791.8	9.72
p1412	Admission	56.62	4.19	12.48	3.05	1.18	56.89	1.16	0	0.84	286.15	0	45.25	0	232.63	0	7.07	2.07	0	5.36	0	250	0	277.97	864.88	436.95	13.73	39.54	28.2	110.05	162381.5	11.13
p1412	D1	59.28	7.39	34.96	6.92	0.1	48.85	1.32	0	1.16	197.07	0	42.35	10.81	13.29	0	10.17	3.24	450	47.78	387	5430	21.22	336.58	591.82	310.08	0.8	17.78	22.4	129.65	165545.7	9.85
p1412	D3	60.88	6.17	15.04	12.59	0	39.04	0	0	1.03	33.7	0	15.79	0	8.61	0	12.53	3.04	0	0	0	0	0	1070.98	980.26	2180.77	8.07	23.58	9	215.62	43540.9	14.65
p1412	D4	48.95	7.39	31.61	16.19	2.57	91.76	1.78	3.26	3.25	14.4	7.88	11.52	4.85	14.44	0.79	9.93	6.07	130	17.27	0	2110	0	559.69	423.47	1798.39	5.67	25.67	9.8	177.82	49450.4	12.15
p1412	D5	54.94	9	47.96	15.74	2.4	112.39	1.62	2.24	4.23	18.28	15.2	11.47	11.82	12.14	0	7.67	6.48	330	33.01	258	3120	0	475.17	343.02	924.13	3.61	25.67	7.6	96.72	31652.4	15.03
p1412	D6	60.68	2.64	9.85	0.69	0	46.18	0	0	1.16	16.48	0	44.07	0	0	0	1.36	0.26	0	0	0	0	0	644.61	700.44	1158	1.29	16.9	11.2	189.25	14489.7	16.64
p1412	D7	76.46	22.48	112.92	28.37	12.61	241.05	6.72	32.95	10.81	22.36	35.88	21.81	4.85	23.87	9.85	18.23	15.26	0	14.12	0	1340	0	301.83	374.12	289.05	12.68	47.4	10.4	175.74	86232.7	26.38
p1411	Admission	38.33	8.75	36.07	9.45	1.09	93.56	1.32	0	1.03	16.2	0	8.4	11.62	17.26	0	4.03	4.62	150	29.02	0	3040	0	153.39	129.16	505.31	0	28.2	22.1	6.9	17602.8	5.49
p1411	Admission	37.2	8	36.07	7.81	0.28	59.57	1.24	0	1.1	55.98	0	15.26	10	5.54	0	4.15	4.56	130	27.03	0	2730	0	142.69	283.19	352.25	0.17	34.73	22.1	5.69	125508	7.47
p1411	D1	41.97	7.02	27.62	5.43	0	37.25	0.64	0	0.48	42.31	0	10.36	2.54	15.71	0	4.15	2.76	0	2.89	0	250	0	137.83	261.24	377.92	0	32.12	13.8	7.51	85201.1	7.54
p1411	D3	66.27	9.38	32.73	5.13	0	96.24	1.01	0	0.91	19.79	9.05	104.23	2.36	4.25	0	6.11	2.62	0	1.63	0	0	0	501.73	741.08	731.61	6.04	63.21	19.9	72.51	13082.5	18.24
p1411	D4	49.18	21.21	72.56	17.24	7.31	137.58	5.5	6.73	3.25	14.79	10.35	12.09	16.73	16.98	4.17	10.64	9.51	330	53.84	143	4160	0	312.85	200.82	473.38	6.69	46.12	15.6	42.92	20731.8	13.43
p1411	D5	51.23	8.5	11.83	3.05	0	121.38	0	0	0.6	14.12	7.63	114.02	2.08	2.57	0	4.77	0.26	0	2.45	0	0	0	396.67	559.46	840.83	3.61	64.37	30.5	119.07	12427.7	16.64
p1410	Admission	44.39	13.67	55.3	13.19	11.62	116.88	9.42	18.63	2.35	16.2	7.38	43.08	24.62	54.71	8.54	10.88	7.31	680	55.87	524	6530	48	321.51	227.94	1088.17	11.31	59.28	17.194	8.7	29576.9	10.04
p1410	Admission	36.91	5.58	37.18	8.41	9.39	67.61	7.94	21.2	1.95	18.22	6.05	39.82	13.04	104.45	11.03	11.7	4.14	280	33.68	205	3240	0	289.89	244.04	1041.68	13.67	63.79	10.137	9.3	140470.5	8.44
p1410	Admission	37.48	4.99	23.55	6.32	9.64	64.93	7.86	21.2	1.55	19.17	0	36.02	14.47	67.34	6.96	8.75	2.9	280	36.36	195	3320	0	253.41	258.39	1044.78	12.45	65.91	6.8	14.02	136706.4	8.18
p1410	D1	47.07	13.67	62.49	14.99	10.22	112.39	6.72	21.43	3.63	59.1	13.45	17.11	16.63	51.86	11.73	11.82	7.1	260	47.11	43.16	4220	0	248.99	220.48	256.07	14.3	71.59	10.7	51.21	98784.2	13.88
p1410	D5	69.04	12.61	64.01	15.59	12.44	119.58	9.18	28.59	4.81	16.54	14.51	19.63	11.62	28.73	14.14	14.86	8.27	180	18.55	0	2390	0	312.36	182.96	464.27	12.68	54.87	8.7	42.37	43284.2	14.59
p1409	Admission	31.33	3.47	12.98	3.45	0	45.9	0	0	1.19	27.18	2.16	4.21	2.92	10.36	0	0	2.86	0	8.69	0	0	0	420.9	147.67	1001.51	0	28.41	31.6	0	11646.8	0.04
p1409	Admission	22.91	5.87	17.4	5.76	0	35.85	0	0	1.04	28.74	1	6.66	10.91	0.33	0	0.16	2.86	410	41.05	95.87	0	0	352.98	139.36	730.99	1.51	29.74	32	0	29869.4	0.19
p1409	Admission	20.97	7.76	19.56	6.74	0	52.28	0.47	0	1.39	46.84	3.83	12.95	8	3.49	0	1.25	3.12	120	22.76	0	0	0	351.01	133.96	671.04	1.77	35.34	30.5	0	43316.2	1.37
p1409	D1	29.67	2.78	12.42	4.45	0	35.85	0	0	0.98	49.31	1.52	12.17	17.35	16.02	0	0.5	3.05	540	55.19	410	0	32.77	321.62	133.64	1097.65	0	34.1	56.5	0	86988.3	0
p1409	D3	48.61	5.7	19.56	5.6	0	39.51	0	0	1.45	4.06	3.7	11.88	4.65	0.02	0	1.66	3.39	270	21.13	95.87	0	0	366.71	308.94	226.33	6.93	33.85	52.9	0	46242.2	1.95
p1409	D5	35.77	2.78	11.85	2.78	0	26.66	0	0	1.1	7.33	3.7	19.91	0	1.22	0	0.84	3.12	0	1.63	0	0	0	533.51	191.03	749.07	0	34.6	35.4	0	20716.8	4.09
p1408	Admission	34.06	9.63	37.11	11.08	1.17	88.49	0.83	0.8	2.44	13.93	5.21	15.16	4.75	21.63	0	2.08	4.25	290	58.23	148	4620	0	170.45	181.25	189.99	0	42.28	25.2	0	7344.1	2.4
p1408	Admission	20.77	4.84	29.51	9.96	0	49.55	0	0	1.81	8.36	3.83	18.55	3.88	9.54	0	1.52	3.58	320	37.7	195	3980	0	161.56	189.61	195.3	0	39.94	13.9	0	33250.2	2.25
p1406	Admission	71.97	23.95	87.41	24.85	11.93	203.56	7.13	15.79	4.94	32.28	14.88	17.14	3.88	34.22	9.65	10.77	12.55	170	16.63	0	2950	0	436.76	166.99	402.64	9.83	38.51	15.7	74.43	16660.7	10.66
p1406	Admission	38.94	19.86	66.59	21.91	9.27	158.66	5.21	16.36	5.04	19.66	13.03	14.35	6.61	18.16	8.98	7.49	10.24	240	35.69	0	3470	0	377.92	112.22	342.86	6.93	30.78	16.1	60.27	36382.3	10.74
p1406	Admission	55.83	22.92	86.04	26.85	11.52	196.38	6.61	22.97	6.31	22.04	15.6	16.7	7.3	24.95	9.46	9.13	12.55	230	28.36	0	3750	0	427.54	127.21	1159.3	8.76	39.46	24.1	70.92	49065.9	14.8
p1406	D1	73.19	4.16	20.09	11.87	0	59.55	0	0.1	2	18.89	3.83	10.96	0	5.16	0	2.21	4.32	0	2.45	0	290	0	369.89	159.59	986.6	0	16.11	22.6	62.06	47581.6	2.1
p1406	D2	40.26	9.97	41.09	13.3	2.19	86.68	1.53	2.97	3.28	18.89	8.67	5.91	0	12.25	0.56	3.75	6.59	0	9.86	0	1390	0	422.32	155.92	412.89	0	18.35	53.7	61.46	19822	6.38
p1406	D3	77.35	30.44	89.69	27.77	12.34	204.46	7.65	21.83	6.96	20.67	16.5	14.88	7.11	25.22	10.61	11.71	14.88	280	32.35	49.49	3750	0	563.8	259.21	464.44	11.41	50.59	21.9	66.8	11356.8	15.4
p1406	D4	58.19	24.97	92.87	25.93	11.6	196.38	6.69	22.59	6.67	15.79	16.67	14.26	2.54	21.09	10.23	11.13	12.21	110	12.88	0	1860	0	650.69	269.02	875.59	11.94	48.18	20.8	67.39	16705.5	14.95
p1406	D5	81.62	16.79	66.12	19.73	9.11	139.79	5.04	14.48	4.35	15.39	12.84	14.92	4.65	17.09	4.07	8.63	9.56	210	19.51	0	2560	0	679.25	286.14	570.35	7.4	39.46	17.9	99.12	14840	13.62
p1405	Admission	33.59	0	1.45	39.68	52.58	17.4	15.12	0	0.49	44.74	0	85.14	0	10.9	0	2.63	29.79	0	0	0	0	0	953.12	171.76	1344.98	0	40.17	30.5	0	253311.4	9.63
p1405	Admission	32.79	0	0.71	16.6	12.26	13.66	3.64	0	0.65	35.59	0	70.08	0	7.09	0	3.05	10.72	0	0	0	0	0	762.92	174.01	1097.65	0	37.79	29.7	0	380129.6	9.48
p1405	Admission	38.2	1.38	23.28	67.61	31.45	50.46	11.3	0	1.16	24.82	6.72	63.85	0	3.77	0	4.73	20.51	0	2.03	0	0	0	746.98	168.06	759.94	0	52.11	31.6	7.18	380289.4	12.58
p1405	D1	53.05	3.98	38.11	31.76	8.43	72.23	4.86	0.8	1.57	20.67	6.15	51.48	0	5.99	0	5.29	12.96	0	4.33	0	0	0	906.27	197.73	464.44	0	48.18	29.7	0.42	298013	11.03
p1405	D3	68.7	5.53	38.61	58.95	14.23	70.42	12.52	0.8	2.47	19.72	8.77	49.83	0	2.93	0	4.17	23.48	100	12.27	0	0	0	739.89	231.1	700.95	1.38	44.58	9.4	0	66361.3	9.04
p1405	D4	68.02	0	3.48	26.85	20.94	15.53	3.29	0	1.81	61.43	2.6	56.64	0	1.36	0	3.75	13.65	0	0	0	0	0	848.2	232.32	610.43	0	31.3	9.4	0	101337.7	4.31
p1405	D5	45.63	10.66	46.01	77.11	32.86	114.62	13.3	9.61	4.18	115.05	13.22	39.88	2.17	19.23	1.05	8.2	21.48	110	18.55	0	0	0	368.79	141.45	251.22	3.62	40.88	7.7	22.72	47597.9	6.82
p1405	D6	67.56	14.4	85.13	191.02	97.91	167.64	52.01	17.49	6.17	125.78	17.46	92.03	0	17.09	1.15	10.7	73.72	0	15.37	0	0	0	640.4	253.29	293.95	19.32	81.83	6.4	49.4	47102	16.88
p1404	Admission	164.81	1.03	1.45	3.12	0	13.66	0	0	0.49	81.47	0	41.47	0	29.99	0	0.3	8.34	50	2.67	0	440	8.7	657.84	596.73	1391.2	0	40.41	90.6	56.67	37208.01	15.4
p1404	Admission	39.67	6.56	17.4	5.76	0	54.1	0	0	1.33	20.54	2.75	23.27	11.17	9.54	0	0.57	3.39	2880	191	1730	19010	254	269.36	120.79	1568.56	0	17.09	107.7	38.88	186974	5.35
p1404	D1	58.43	1.38	15.21	3.12	0	26.66	0	0	1.21	86.44	5.45	20.9	0	0.33	0	1.73	2.2	0	0	0	0	0	489	280.32	706.95	0	20.5	91	65.03	322816	8.74
p1403	Admission	34.84	8.61	35.61	14.25	2.82	70.42	4.51	3.33	1.93	50.73	6.38	60.58	21.49	10.9	4.55	2.35	5.72	460	68	568	5950	40.04	104.43	196.29	174.16	4.93	31.56	76.6	0	10077.6	1.73
p1403	Admission	31.66	10.66	39.11	24.07	3.44	84.88	6.26	4.07	2.31	64.48	7.05	19.67	13.25	6.81	3.1	3.12	6.73	170	37.03	0	2440	0	88.81	170.66	126.45	3.62	33.09	82.8	0	31799.4	2.25
p1403	Admission	26.92	5.7	23.28	18.95	1.82	61.36	8.52	2.6	1.57	72.67	5.56	59.54	7.8	15.48	0.96	2.21	6.59	130	22.43	0	1940	0	120.46	359.19	296.16	4.75	58.03	54.1	0	38508.2	3.13
p1403	D1	21.37	3.47	12.98	28.08	0.5	41.34	8	0.45	1.16	53.54	1.18	7.74	11.22	4.88	0.76	1.73	5.99	150	25.06	0	2780	0	211.65	168.83	191.05	0	36.08	30.1	0	43059.6	1.51
p1403	D2	39.82	13.55	44.54	30.84	5.19	99.31	10.17	7.2	3.11	39.64	7.49	14.1	18.17	8.73	4.84	4.87	9.16	240	45.76	0	3870	0	271.85	254.49	297.26	6.12	63.76	62.53	0	23616.1	8.08
p1403	D3	31.66	11.17	38.11	17.54	2.37	87.59	3.38	2.97	2.5	18.38	7.16	4.39	20.87	6.26	5.42	2.63	5.12	320	51.15	195	4590	0	156.52	103.71	825.48	1.64	29.47	118.2	0	26215	2.1
p1401	Admission	78.42	7.24	23.81	21.6	0	41.34	0.11	3.7	0.98	29.93	8.46	11.21	4.26	36.47	1.35	0.16	18.79	290	28.36	718	1610	57.82	531.11	354.97	924.76	7.4	111.4	55.7	4.01	14795.3	7.86
p1401	Admission	24.41	10.66	25.38	24.69	0.3	63.17	1.53	0.1	1.21	25.73	4.84	6.31	33.46	1.51	4.26	1.66	10.1	1650	138	1427	14600	160	165.05	131.12	149.1	0.2	44.12	20.4	0	45029.7	3.13
p1401	Admission	17.03	8.61	21.16	6.09	0.2	48.64	0.02	0	1.33	31.93	3.3	8.08	39.83	5.16	0.17	1.52	4.58	1670	145	1440	15230	163	118.62	139.2	164.72	0	46.84	18.2	0	97404.6	3.06
p1401	D2	49.4	6.22	20.63	29.62	0	54.1	0.83	0	1.39	35.53	4.09	9.45	11.32	2.37	2.13	2.63	8.88	780	60.59	949	5540	79.01	292.37	227.3	867.02	0	45.26	25.2	13.08	25293.6	3.87
p1401	D3	31.66	0	4.75	1.42	0	102.91	0	0	0.76	21.92	0	144.46	2.36	0	0	1.46	0.9	0	2.03	0	0	0	301.73	432.09	1523.03	0	40.88	37.3	57.88	20205.3	5.05
p1401	D4	52.79	0	0	8.03	0	68.61	0	0	1.3	14.06	5.09	120.99	2.73	0	0	1.52	6.05	0	4.84	0	60	0	686.38	556.08	929.7	0	56.36	66.5	55.47	11453.4	12.88
p1401	D6	38.49	14.06	44.54	17.7	4.93	113.72	1.8	7.76	4.01	13.12	10.11	12.46	33.04	9	3.87	5.01	8.21	1010	121	1098	11990	96.39	363.29	170.22	374.33	4.01	49.94	31.2	36.99	13998.1	6.23
p1399	Admission	40.84	6.56	11.85	8.68	0	32.18	0.11	1.16	1.45	0	7.38	2.64	4.95	6.95	0.37	1.73	3.12	540	29.24	413	4430	76.53	704.61	268.03	3909.1	0	31.56	64.9	38.25	24968.51	15.99
p1399	Admission	24.22	12.02	34.6	12.82	1.45	58.64	1.09	0	1.36	78.19	6.49	18.47	20.17	9.82	0	2.91	4.05	1110	74.35	907	11240	109	200.48	231.2	958.11	0	38.75	42.7	38.25	188338.4	8.15
p1399	Admission	22.53	6.39	32.07	9.96	0	55.91	0.29	0	1.04	87.29	5.09	20.86	11.27	35.94	0.96	2.21	3.39	520	47.69	376	5270	52.93	321.62	384.07	618.72	1.38	44.35	22.2	41.38	98052.75	9.85
p1399	D1	32.47	12.36	47.96	15.19	2.37	83.07	2.41	5.54	1.63	68.96	8.24	16.82	20.59	12.52	2.03	5.78	3.78	1120	72.18	860	10700	115	271.59	286.92	708.15	3.42	56.15	47	56.07	156273.2	13.47
p1399	D1	25.68	11.51	41.09	13.77	1.82	80.36	2.41	3.7	2.06	73.9	8.67	15.24	11.17	6.81	1.93	8.27	6.12	510	50.56	430	6060	61.92	318.35	231.68	446.04	2.36	33.85	13.9	54.87	95173.68	9.41
p1399	D2	27.62	8.27	20.09	9	0	54.1	0.38	0	1.48	27.42	5.21	4.84	5.49	1.22	0	3.96	2.92	340	27.16	252	3310	36.22	352.24	230.36	677.01	0	25.69	12.5	21.38	54468.66	6.45
p1399	D3	28.66	13.72	42.57	14.4	2.19	79.46	4.16	9.24	2.63	5.58	8.24	5.43	11.67	5.85	5.23	5.01	4.72	480	49.12	327	5320	43.97	317.59	197.7	541.05	6.12	34.1	17.9	26.7	34349.08	9.85
p1399	D4	36.08	8.95	32.07	9.64	0.3	67.7	1.09	0.45	1.93	9.58	3.83	7.14	10.19	4.61	0.17	3.05	3.32	390	35.56	247	4960	36.49	451.82	224.2	484.07	0	27.06	12.2	32.53	27902.7	5.35
p1398	Admission	85.58	10.32	30.03	10.92	8.94	55.91	10.87	38.97	2.56	31.46	3.57	18.95	23.78	24.82	9.37	7.13	3.39	1620	111	1429	0	214	431.92	285.55	1149.21	6.93	37.06	31.2	4.01	13462	8.89
p1398	Admission	58.19	5.87	26.94	7.71	6.48	57.73	7.48	26.76	1.81	19.28	4.35	13.61	21.7	35.81	6	4.66	2.59	960	78.78	1201	0	133	395.08	199.07	559.78	2.8	22.28	27.5	27.35	15287.3	6.97
p1398	Admission	122.56	2.26	22.22	5.1	8.43	32.18	9.48	34.19	1.48	15.92	2.46	15.49	15.91	19.49	8.69	5.64	2.46	480	50.47	760	0	105	386.27	251.93	849.89	4.57	19.29	24.8	0	26909.5	5.79
p1397	Admission	46.04	9.63	26.94	18.17	0.09	128.11	0	0	1.54	26.88	4.84	33.93	26.09	21.63	0	3.33	4.85	2120	141	1826	14170	185	856.48	229.64	3830.98	0	46.17	53.7	30.6	16332.1	8.52
p1397	Admission	39.38	16.11	36.61	13.14	2.19	108.32	1.36	0	1.75	21.98	5.21	18.79	70.24	7.64	0.07	5.22	6.52	6500	329	3498	34520	463	624.71	214.02	3308.88	0	40.65	24.8	34.45	49131.3	9.63
p1397	Admission	25.14	0	3.48	1.24	0	22.97	0	0	0.44	14.06	0.1	6.57	3.3	0	0	0.57	0.9	190	34.35	0	2500	0	358.51	144.38	2071.63	0	18.04	28.2	25.38	73123.9	1.66
p1397	Admission	38.64	7.24	23.81	7.71	0	56.82	0	0	1.36	7.7	4.84	7.22	12.94	2.08	0	1.52	3.65	650	79.12	766	7680	53.91	372.08	117.07	1760	0	32.07	18.6	46.95	102450.4	4.02
p1397	D1	49.4	12.7	44.05	14.72	2.91	86.68	1.88	3.33	2.72	19.28	10.91	8.17	14.88	7.91	0.37	4.94	6.05	400	70.03	308	5540	0	318.22	143.69	544.56	0	39.22	11.8	41.38	34793	6.97
p1397	D2	78.21	6.73	19.02	5.43	0	74.04	0	0	1.39	17.42	5.33	8.94	12.84	3.49	0	2.49	2.86	1860	110	1711	13710	215	443.13	340.13	1091.38	0	42.74	14.3	50.62	24913.5	7.26
p1397	D3	70.06	23.26	68.94	21.13	5.54	156.86	2.59	3.7	3.54	3.15	12.46	11.29	94.68	10.76	0	6.49	9.63	7100	366	3491	41900	554	427.3	193.22	1333.45	1.64	57.41	24.5	63.84	15153.1	11.99
p1397	D4	81.19	0.68	0	0	0	64.99	0	0	0.6	9.73	1.52	47.05	0	0	0	0.23	0.39	0	30.35	482	540	0	904.5	757.25	2421.52	0	72.02	41.6	100.25	12070.7	23.4
p1397	D5	80.77	25.82	67.53	23.15	6.23	192.79	3.64	3.52	4.15	6.35	17.2	15.32	50.11	13.33	0.27	6.99	12.83	3550	184	2532	22140	331	629.42	261.29	1679.72	4.75	71.64	49.8	72.1	12777.4	17.32
p1396	Admission	83.5	15.43	59.5	18.17	3.8	90.29	3.29	15.23	3.28	64.85	8.14	37.22	2.36	25.62	13.49	4.52	5.79	310	11.66	300	4040	24.14	733.02	410.39	1050.35	7.25	58.86	62.1	255.15	56811.2	22.51
p1396	Admission	56.46	7.93	42.08	11.55	0.5	75.85	1.53	8.68	2.82	53.54	5.33	30.86	3.49	13.87	10.71	1.66	3.52	460	23.74	614	5400	48	721.82	263.68	975.91	5.28	38.51	67.7	227.06	35504.5	14.06
p1396	Admission	60.02	12.87	47.47	14.4	2.19	90.29	3.11	9.06	2.69	63.8	6.26	39.19	6.81	15.48	13.1	3.05	3.92	700	45.09	718	7510	68.29	959.53	372.56	551.21	4.57	56.57	74.6	302.48	94435.4	21.47
p1396	D1	70.51	5.87	40.1	10.92	0	62.27	0.83	57.26	2.79	75.44	4.84	45.43	2.45	18.29	12.34	2.91	3.19	260	13.5	214	3440	13.56	1094.2	507.62	5155.06	3.42	50.16	15.4	342.21	91063.9	19.77
p1396	D1	88.86	2.78	29.51	4.78	0	70.42	0	41.85	1.75	60.17	4.35	148.21	0	10.36	10.23	1.39	0.02	0	0	0	1660	0	1560.43	1158.22	6896.92	7.86	68.91	91.8	507.43	27490.4	32.01
p1396	D2	73.85	7.93	46.01	10.6	0	55	0.47	5.54	2.37	34.9	7.6	33.24	2.17	8.73	8.79	2.21	2.72	250	11.66	249	3180	10.07	845.11	585.5	5681.53	5.45	47.29	60.9	359.44	24784.3	22.06
p1396	D3	60.74	3.98	26.42	6.41	0	45.9	0.29	17.11	2.12	33.21	6.26	23.27	2.26	9.27	8.41	1.04	1.41	170	11.05	143	2780	0	918.64	451.52	4593.01	4.57	48.18	28.6	368.54	7493.4	21.99
p1396	D4	50.19	8.78	41.09	12.35	0.69	70.42	1.53	5.91	2.12	23.65	5.21	21.02	3.11	10.9	7.54	1.94	3.52	190	8.11	95.87	2840	0	713.05	357.59	4547.07	5.1	43.67	33.9	343.23	9549.4	16.58
p1396	D5	48.34	5.7	31.05	7.71	0	44.99	0	4.43	2.59	20.29	5.33	17.95	3.11	10.36	6.87	1.25	1.68	200	9.27	143	2840	0	607.18	351.83	4819.14	3.52	44.12	31.6	292.24	15533.4	16.28
p1394	Admission	42.7	16.11	45.03	13.14	5.88	101.11	4.78	7.2	2.44	35.07	5.09	12.95	10.41	17.36	9.46	4.17	5.18	1110	49.13	1123	9810	131	211.37	135.84	473.67	3.01	52.11	20	18.67	20518.9	4.9
p1394	Admission	41.7	15.77	48.93	11.87	4.85	101.11	4.16	4.25	2.06	18.19	5.09	9.11	29.87	16.55	7.54	3.68	6.12	1280	99.62	1138	10730	135	195.91	98.82	172.06	3.91	41.58	13.6	13.08	4637.2	3.72
p1394	D1	133.52	11.68	22.75	11.24	0	55	0.65	2.24	1.04	4.95	1.85	8.34	0	3.21	0	3.19	3.45	130	16.63	153	1110	0	515.17	738.08	1380.91	5.45	112.23	116.1	12.36	7624	18.21
p1394	D5	30.34	6.56	23.28	7.06	1.08	41.34	2.24	5.35	1.45	17.22	1.85	5.96	22.12	10.09	4.26	3.4	2.46	1700	106	1451	11620	172	598.63	144.15	1239.07	3.01	45.49	14.3	0	3233.8	6.3
p1393	Admission	82.29	0	57.08	0	2.87	10.42	3.56	13.13	1.41	22.84	6.37	11.81	17.8	90.21	5.47	5.2	2.74	720	62.16	565	6210	79.56	324.75	378.21	1426.65	8.64	42.99	17.8	20.99	38304.6	6.66
p1393	Admission	43.98	0	30.18	0	0.49	0	2.77	6.52	1.06	13.35	0	10.17	24.52	5.21	0	3.49	1	680	70.25	446	6570	64.84	418.08	379.93	878.38	6.57	34.1	11.2	35.72	236781.2	4.81

p1393	D1	57	0	58.73	0	4.53	35.09	4.48	12.1	1.75	12.75	14.03	11.49	15.59	7.74	9.98	6.52	3.44	560	48.34	384	5100	55.98	383.37	295.78	885.39	8.27	47.86	6.4	72.34	204318.8	8.6
p1393	D1	92.44	0	69.69	0	6.04	26.32	4.09	15.54	1.49	11.53	18.12	11.39	9.96	5	8.04	8.76	4.83	530	40.53	446	4550	44.86	489.32	420.28	1830.53	8.92	54.58	9.7	99.28	128901.1	10.63
p1393	D2	65.71	0	5.09	0	0	0	1.41	2.24	0.71	3.9	0	2.85	0	0	0	2.78	0.91	0	16.87	0	1240	0	571.37	170	1357.22	6.68	25.9	6.1	27.97	74246	3.52
p1393	D3	87.15	0	86.65	0	2.31	10.42	2.77	9.32	1.41	6.13	11.62	7.44	2.27	0.95	4.93	4.35	3.26	240	25.91	153	2560	2.75	431.71	251.51	1041.26	6.26	35.57	8	17.92	42513.9	7.12
p1393	D4	138.14	0	5.09	0	0	6.86	1.13	4.39	0.89	6.13	0	42.85	0	1.14	3.51	2.41	1.78	0	0	0	0	0	419.2	519.31	2466.39	11.94	44.24	13.2	7.16	30803.1	10.24
p1393	D5	110.89	0	57.08	0	5.63	35.09	4.35	14.85	1.75	8.49	19.34	12.44	2.49	3.14	7.03	6.84	4.48	300	22.91	145	2450	17.18	404.38	353.75	1965.63	11.28	50.45	26.9	56.2	28550.1	10.32
p1387	Admission	57.13	33.96	318.47	127.48	47.84	282.8	21.6	306.53	18.6	126.86	62.89	87.45	38.81	48.7	124.49	36.71	10.69	9010	158	4897	61830	835	222.39	283.25	1678.99	23.37	70.14	22.9	251.2	20675	16.69
p1387	Admission	32.58	11.47	179.36	30.31	11.1	102.89	6.04	57.18	6.61	49.3	26.65	24.18	23.23	16.45	32.88	11.87	4.66	7140	88.7	3989	49900	625	187.61	273.37	824.13	13.61	43.62	16.3	64.33	52388.3	6.97
p1387	Admission	49.94	23.87	187.53	43.32	17.63	114.98	7.19	58.5	4.59	105.22	33.5	24.18	40.91	20.45	28.11	15.48	8.24	12520	193	5562	73940	1047	191.23	731.08	1250.41	13.3	50.74	10.9	116.22	75074.2	11.65
p1387	D1	49.94	23.87	187.53	43.32	17.63	114.98	7.19	58.5	4.59	105.22	33.5	24.18	33.32	20.45	28.11	15.48	8.24	10350	127	4992	66380	862	191.23	731.08	1250.41	13.3	50.74	10.9	116.22	67609.5	11.65
p1384	Admission	75.76	0	0	0	0	24.56	1.13	3.32	1.49	38.88	6.37	30.93	2.27	109.78	0.53	3.83	0.65	330	35.48	184	3550	13.02	1317.37	867.19	5141.55	6.68	122.08	90.9	125.42	68640.4	13.7
p1384	Admission	39.88	0	30.18	0	2.87	33.34	2.5	7.22	4.68	22.15	7.63	24.8	0	14.57	0.53	5.86	2.04	150	16.11	0	1900	0	597.17	371.39	3198.62	5.6	91.87	101.7	132.07	122759.1	11.49
p1384	Admission	34.17	0	22.74	0	0	24.56	1.41	5.81	4.85	25.88	0	26.28	0	9.24	0.03	6.19	1.34	340	28.14	137	3580	1.22	662.47	577.86	2605.55	5.82	97.15	76	192.84	145013.2	10.87
p1384	D2	45.66	0	68.18	0	2.03	24.56	2.23	8.62	11.69	48.8	2.94	30.16	3.36	8.06	1.29	10.64	2.04	300	25.91	54.72	3500	1.22	805.55	840.76	1583.68	7.69	108.98	50.7	401.01	273895.4	11.89
p1384	D3	47.23	0	19.96	0	0	13.97	1.41	8.62	4.99	23.81	0	24.29	0	5.21	0.53	10.02	1.52	260	22.91	0	3000	0	984.93	687.81	1802.84	6.68	78.18	40.4	529.65	189067.2	10.94
p1384	D5	47.79	15.68	72.56	20.1	9.97	144.79	4.7	19.57	5.61	15.86	19.89	16.67	8.86	21.69	8.9	10.64	10.05	320	31.09	63.13	3470	0	803.31	289.29	1771.07	8.99	63.98	40.8	124.37	124769.5	15.16
p1383	Admission	51.24	0	34.65	23.85	0	0	8.96	5.11	1.23	20.18	0	13.34	10.32	7.74	0	0.45	7.98	270	41.25	29.19	0	0	202.74	255.8	4798.5	11.2	50.45	25.9	9.41	15593.2	12.44
p1383	Admission	41.8	7.85	102.04	16.71	13.15	108.07	6.42	26.44	3.76	22.7	26.65	13.02	40.01	35.65	13.22	9.08	10.96	960	97.73	660	0	74.41	185.37	193.17	3177.78	10.26	65.89	23.6	117.06	56429.7	17.99
p1383	Admission	39.16	8.37	73.55	57.09	0	55.1	0.49	0	1.16	8.39	0	10.73	0	0	0	0.27	21.14	170	29.62	0	0	0	150.89	114.56	2866.46	0	36.36	64.3	3.23	118940.8	8.12
p1383	D1	38.33	6.82	89.31	13.16	6.73	76.91	6.16	17.6	4.28	37.52	15.49	12.76	31.46	15.78	9.02	11.56	8.16	730	78.23	520	0	53.39	250.63	238.66	2104.34	8.92	61.28	2	85.49	73512.8	15.44
p1381	Admission	72.52	3.21	120.85	0	0.34	121.89	1.68	26.78	7.89	734.57	14.03	183.01	0	32.1	23.77	7.49	2.91	0	0	0	0	0	436.5	2953.88	1501.51	3.39	115.47	27.2	54.38	39697.8	26.9
p1381	Admission	59.84	11.2	102.19	5.71	0	70.97	7.25	29.56	5.82	248.1	16.55	73.32	15.25	23.44	23.92	13.49	1.8	760	62.16	632	6180	48.57	488.71	868.82	620.65	7.84	38.85	14.4	63.48	299070.6	9.75
p1381	Admission	37.42	0.37	111.65	0	1.19	35.09	2.9	19.31	6.52	135.83	2.94	43.65	11.76	34.04	18.85	8.13	2.04	550	55.3	426	5850	34.43	443.07	712.64	509.07	3.11	56.96	11.4	59.83	343715.4	11.81
p1381	D1	29.81	6.1	99.61	3.2	0	4.71	3.12	18.96	3.81	81.32	0	27.46	13.52	12.05	13.94	8.21	0.8	540	50.44	419	5430	37.41	438.52	573.6	433.29	7.03	38.04	13.8	53.98	284229	7.41
p1377	Admission	42.43	0	149.57	0	3	63.01	2.23	4.39	2.01	6.96	15.49	10.59	0	14.46	7.54	9.08	2.22	0	0	0	0	0	470.56	217.07	3348.86	11.78	39.74	18.4	0	20568.6	12.44
p1377	Admission	30.01	1.66	138.25	1.65	8.92	104.61	4.61	14.85	2.45	7.93	23.72	9.26	13.87	5.94	13.67	13.69	4.66	250	31.83	0	0	0	312.87	149.33	2694.36	13.53	44.86	28.3	56.2	39852.5	13.38
p1377	Admission	44.6	3.72	116.3	1.33	9.19	111.53	4.87	18.28	2.32	9.21	23.72	9.26	17.63	7.53	14.11	13.09	4.83	210	30.36	0	0	0	289.24	196.72	1571.42	11.78	51.3	22.6	64.33	57659.4	13.54
p1377	D2	45.96	6.3	146.53	3.75	9.74	114.98	4.87	17.6	2.45	10.92	24.73	19.91	11.76	16.45	16.3	15.34	5.79	120	21.79	0	0	0	257.02	281.96	1439.19	13.06	60.29	10.3	75.87	182404.9	14.18
p1377	D3	44.67	5.79	149.57	2.26	10.69	101.16	4.35	17.94	2.45	9.05	24.23	10.33	15.08	8.28	16.95	13.99	4.83	250	34.75	0	0	0	251.26	239.88	1011.9	13.22	59.78	18.1	87.23	98737.4	15.6
p1377	D4	52.44	6.82	138.25	6.28	13.55	147.75	5.65	21.01	2.89	14.69	25.7	16.95	28.97	10.75	18.43	14.89	7.37	650	60.11	349	0	18.68	341.88	287.78	1709.15	13.22	59.78	45.5	74.99	36288.6	19.59
p1375	Admission	53	0	63.54	0	0	361.81	2.23	2.96	2.54	109.27	0	44.56	14.78	275.32	0	0	0.47	1280	85.16	916	10780	165	591.27	1318.61	3937.21	3.25	66.59	23.9	0	15219	15.6
p1375	Admission	37.42	0	30.18	0	0	214.75	1.41	2.24	1.23	93.4	0	70.22	8.75	29.25	0	0	0.39	580	43.39	406	5460	101	593.67	1391.2	4154.51	1.85	35.57	7.5	24.01	182672.7	12.83
p1375	Admission	37.42	0	85.31	0	0	29.83	1.96	5.11	1.41	126.53	0	96.1	4.07	88.17	0	1.67	0.82	300	23.73	282	3040	53.49	544.23	1326.29	3271.35	2.97	49.88	8.9	101.84	281442.4	17.03
p1375	Admission	33.64	0	81.2	0	0.06	0	2.77	8.62	1.93	283.57	0	87.44	39.99	36.68	0	5.53	2.04	1410	132	1041	14090	117	423.89	1128.5	2453.48	4.79	41.06	10.3	88.1	358586.2	14.89
p1375	D1	38.08	0	94.51	0	0	10.42	1.13	5.11	2.19	239.29	0	128.27	0	18.89	0	6.52	1.34	20	3.91	0	240	0	845.18	1896.18	4211.7	2.51	53.23	12.6	148.53	264244.4	19.67
p1375	D2	23.55	0	97.05	0	0	106.34	1.41	5.11	2.32	32.27	0	147.82	0	5	0	8.13	0.65	10	1.04	0	0	0	710.94	882.63	3108.06	1.85	47.86	21.6	144.43	178524.4	18.63
p1375	D3	35.56	0	82.58	0	0	10.42	1.13	2.96	3.32	59.61	0	38.52	0	4.37	0	4.35	0.3	30	1.94	0	340	0	773.76	1119.26	3011.6	1.85	38.39	7.2	102.69	102906.1	20.23
p1375	D4	30.94	0	97.05	0	0.34	36.84	2.5	7.92	2.54	30.61	0	26.49	26.62	5.42	0	5.03	1.87	940	80.11	663	9040	103	636.75	611.79	2623.73	2.82	39.4	5.4	82.88	80054.61	14.02
p1375	D5	22.8	0	78.41	0	0	97.7	1.13	5.81	1.41	23.4	0	54.25	0	3.55	0	4.35	0.65	0	0.79	0	0	0	604.39	742.68	2276.7	1.85	39.74	7.8	69.68	54621.38	17.35
p1375	D6	27.33	0	40.84	0	0	49.07	1.68	5.11	2.62	17.39	0	25.82	0	4.79	0	2.78	1	10	0.79	0	190	0	495.71	592.15	2081.37	2.19	32.58	6.7	50.71	44514.7	16.56
p1374	Admission	79.4	0	79.81	0	4.25	45.58	3.83	11.75	4.37	32.34	15.49	22.33	27.72	16.67	4.37	6.84	3.09	990	119	854	12360	118	533.49	368.5	2933.93	10.61	46.08	15.6	91.55	107094.3	16.71
p1374	Admission	65.84	0	83.95	0	1.19	13.97	2.5	8.62	2.75	23.47	0	23.16	40.57	6.68	0.53	5.2	1.69	1610	182	1102	15910	147	432.6	276.48	2962.23	7.69	34.84	14.7	61.63	134292.4	9.92
p1374	D1	86.36	0	112.82	0	0.34	31.59	2.5	5.11	2.71	132.62	0	49.76	15.39	40.14	1.98	8.13	1.61	770	75.79	606	8040	88.26	637.84	789.69	2748.41	15.19	62.03	26.2	119.58	268133	19.35
p1373	Admission	121.62	2.17	81.2	0	4.8	35.09	3.3	15.54	1.67	89.61	16.84	58.48	7.33	31.98	7.03	5.53	4.75	230	21.03	145	2300	36.22	339.79	870.85	1737.18	14.3	89.74	84.4	35.72	17395.96	14.97
p1373	Admission	30.38	1.4	75.56	0	3.97	35.09	4.09	14.17	1.58	18.04	14.03	13.23	0	2.63	5.2	6.52	3.61	0	0.79	0	0	0	149.49	233.57	540.73	8.07	39.74	24.2	51.63	81318.8	7.74
p1373	Admission	33.29	0	75.56	0	3.83	31.59	3.03	12.1	1.32	17.17	14.03	14.65	12.36	1.53	3.8	6.52	3.26	410	43.39	226	3910	41.19	144.17	201.42	727.73	9.38	43.62	21.6	32.84	88431.54	7.9
p1373	D1	35.13	0	132.94	0	2.45	21.04	2.77	8.62	1.67	18.18	10.73	12.55	8.65	1.73	2.61	5.2	2.48	260	24.41	69.54	2040	30.76	203.02	332	561.61	9.01	43.62	18.1	47.02	52327.97	7.28
p1373	D1	48.41	0	161.44	0	0	10.42	1.41	3.68	1.28	23.61	0	23.57	0	0	0	3.13	1.52	20	3.48	0	160	0	621.99	643.73	1005.1	8.64	45.77	19.1	0	34881.1	11.02
p1373	D2	45.36	3.21	138.25	3.75	7.82	73.43	5.39	20.33	2.62	25.05	23.21	21.2	0	2.94	7.54	8.45	5.01	10	1.94	0	0	0	542.47	576.97	1137.03	14.67	69.61	35.1	70.57	29507.76	19.27
p1373	D3	87.26	21.28	0	0	0	10.42	2.23	5.81	0.54	4.95	0	118.69	0	0	0	1.28	0	80	3.06	0	270	5.4	511.76	301.35	1487.02	11.28	58.26	18.8	34.76	36808.53	10.87
p1373	D4	45.36	17.67	208.48	13.42	19.79	154.63	9.96	38.55	3.93	16.74	43.49	13.92	25.1	9.78	20.73	16.37	10.96	710	73.26	576	7780	91.05	391.97	348.46	1612.21	18.24	84.18	43	136.2	21284.2	20.87
p1372	Admission	61.59	6.82	111.65	15.2	10.15	97.7	6.93	23.05	3.06	16.01	34.29	9.48	63.76	29.7	11.39	10.02	7.46	1190	159	951	0	97.13	368.45	214.55	3252.34	14.3	53.77	21.6	101.84	26232.1	14.18
p1372	Admission	38.58	0	44.71	0	0	0	1.41	5.11	0.8	14.39	0	11.28	39.71	0.03	0	1.67	1.61	1020	119	844	0	71.1	482.27	185.37	2501.34	9.1	30.2	10	4.83	47084.7	8.36
p1372	Admission	182.62	0	134.01	0	0	0	0.85	2.24	0.63	12.45	0	17.16	0	0	0	0	1.34	0	13.08	0	0	0	580.33	489.73	3789.17	7.59	27.68	12	22	30949	11.1
p1372	D1	72.16	1.14	105.69	6.28	3.7	36.84	3.03	17.94	1.84	7.93	18.12	7.98	28.65	3.96	6	5.7	5.36	460	66.22	238	0	8.47	460.16	224.23	2519.7	9.91	42.99	10.6	68.79	26049.3	11.81
p1371	Admission	64.5	1.14	97.05	2.87	5.9	106.34	4.09	12.79	1.75	230.5	15.49	22.74	33.32	33.58	1.98	11.87	5.18	1530	103	1215	0	123	304.09	490.26	1555.04	34.43	237.72	12	0	32404.5	9.3
p1371	Admission	34.35	0	97.05	0	2.31	33.34	3.3	12.1	1.49	46.29	7.63	12.97	6.78	7.32	2.61	9.24	3.61	280	37.65	113	0	0	216.65	191.41	899.39	17.41	184.51	8	0	54066.6	6.5
p1371	Admission	34.69	0	97.05	1.65	5.63	50.82	4.35	14.17	1.75	39.4	14.03	12.34	23.56	4.79	3.22	10.17	4.66	570	64.19	398	0	37.41	212.41	147.88	793.1	18.65	175.56	6.9	0	383847.2	7.97
p1371	Admission	49.14	0	66.65	0	0	0	1.68	4.39	0.89	13.95	0	16.58	0	0	0	7.17	1.52	0	14.6	0	0	0	500.77	329.08	1114.93	36.71	221.61	5.1	0	156235.6	8.75
p1371	D1	68.97	0	157.53	0	1.19	13.97	1.96	8.62	1.23	12.9	0	10.86	6.59	2.83	0	8.61	2.74	520	50.44	401	0	29.94	445.3	428.11	1235.29	34.22	237.01	4.6	0	66478.7	11.81
p1371	D3	53.21	0	61.96	0	0	56.05	1.41	4.75	0.89	11.07	0	51.16	0	2.13	0	5.53	1.61	0	0	0	0	0	293.09	206.72	1388.88	22.23	179.06	7.5	0	30623.5	8.52
p1371	D5	52.58	0.63	166.25	8.99	8.1	59.53	3.56	23.73	2.19	7.61	23.72	11.81	10.69	3.14	4.93	12.17	7.11	320	25.16	157	0	3.52	293.09	229.38	945.69	24.03	180.93	15	0	21593.7	11.49
p137	Admission	80.1159	87.2438	112.3422	99.4062	45.2284	2286.891	19.9919	94.7805	32.0496	431.8845	532.1695	223.1153	0	45.3132	69.8327	69.1354	32.1813	0	0	0	0	0	32.2609	1586.379	150.0928	107.0201	332.7244	22.803	827.1852	60768.58	10.0117
p137	D1	58.9254	48.6558	80.7567	85.384	0	1024.895	33.2116	88.147	19.9347	341.981	488.8773	439.9588	1.29	50.6709	67.8269	53.7069	0.9488	60	6.59	0	0	7.59	55.317	1367.277	199.9018	92.2572	255.3197	30.651	802.3564	191421.9	8.027
p137	D4	109.7018	41.5413	83.358	92.8029	0	3814.292	12.3625	47.3446	13.0622	473.8257	623.9114	207.293	3.1	22.8168	67.8269	48.242	0	200	12.24	56.21	150	20.3	93.7847	2308.779	163.9297	87.9873	180.0588	93.649	1144.336	131217.6	7.0317
p137	D6	68.1075	103.4622	126.7476	92.8029	53.2379	1506.353	25.0152	36.6982	47.3781	106.4331	247.7818	60.6879	0	13.6051	65.8325	38.4755	0	0	2.77	0	0	3.06	39.4793	784.9631	96.5441	109.108	93.9609	27	624.3177	61386.9	3.0189
p1369	Admission	82.06	0	57.08	0	5.49	135.69	2.23	2.96	2.41	272.16	53.69	70.66	3.98	174.73	19.27	16.96	76.3	120	2.25	30.52	260	0	279.79	1331.67	4319.08	5.38	70.29	27.6	7.16	9190.841	13.31
p1369	Admission	51.09	0	50.2	0	5.9	142.58	2.77	8.27	2.45	143.91	33.5	69.53	7.42	10.21	18.22	13.99	16.39	480	27.23	422	890	75.18	223.49	764.55	2578.22	4.18	42.03	14.7	33.8	46779.01	11.65
p1369	Admission	36.32	0	69.69	0	6.73	121.89	3.3	7.22	2.8	172.07	32.71	53.01	11.51	18.66	14.11	14.59	9.56	700	53.58	798	1510	106	551.45	472.38	1462.11	4.43	38.39	11.2	64.33	157113	11.65
p1369	D2	30.48	0	55.4	0	0	233.61	1.68	5.11	1.75	10.61	23.72	289.32	0	2.73	9.02	6.84	1.17	90	2.66	0	0	0	1069.8	612.29	2821.88	9.2	54.05	11.4	332.08	29451.55	14.97
p1369	D3	40.77	0	71.18	0	5.35	97.7	3.3	7.22	5.07	35.17	32.71	20.47	23.94	6.68	11.85	14.59	4.31	1360	92.93	1637	3480	223	356.42	482.38	737.5	5.15	37	3.8	51.63	0	11.02
p1369	D4	42.04	1.66	78.41	1.01	8.92	127.06	3.83	10.01	4.9	50.68	33.5	21.71	28.57	10.32	13.67	16.37	6.23	2310	119	2237	5690	310	346.51	381.79	993.74	4.79	42.35	5.9	36.68	42745.8	12.44
p1369	D6	36.75	0	72.65	0	5.08	219.9	1.96	4.39	4.11	17.89	31.07	304.63	3.98	6.47	14.77	11.87	7.28	90	1.5	0	160	0	201.52	468.32	1895.42	3.66	35.93	3.4	13.74	20852.87	11.02
p1368	Admission	35.52	15.28	86.33	8.75	3.37	211.94	3.44	0	1.92	284.21	11.71	106.25	4.2	89.77	0	11.76	0	910	69.58	801	7180	84.69	464.26	615.27	2137.71	8.39	40.67	41.9	0.24	89256.9	9.04
p1368	Admission	34.62	29.93	159.18	14.69	12.04	225.78	7.53	7.51	3.67	150.59	21.01	62.22	10.87	38.14	12.44	23.35	5.14	1270	93.23	1049	9290	123	353.66	255.52	1865.49	9.64	60.72	42.7	67.5	268477.4	15.42
p1368	D1	20.85	11.7	98.6	5.68	2.81	54.49	2.53	0	1.67	50.05	12.08	45.84	0	31.52	0	12.45	0	970	63.51	691	6430	71.47	297.05	255.87	1259.08	8.02	31.39	8.3	62.65	529451.2	9.46
p1368	D2	30.36	13.04	68.72	3.57	0.95	49.06	2.22	0	1.18	48.38	6.59	18.41	0	0	0	7.96	0	390	36.93	356	3280	29.94	518.87	495.26	1004.63	4.19	37.11	2	0	151783.5	8.13
p1368	D3	31.82	15.73	124.13	9.52	4.19	112.96	2.06	0	2.45	21.27	12.8	19.44	0	0	0	12.22	0	380	34.75	356	2290	28.07	480.96	357.45	915.25	8.02	40.67	2.3	0	71056.8	9.11
p1368	D4	28.06	12.82	165.63	2.42	0.5	65.27	1.9	0	2.52	22.24	6.08	44.55	0	0	0	7.23	0	0	0	0	0	0	865.95	693.9	2093.79	8.58	43.23	4.6	0	49495.3	15.91
p1368	D5	33.47	30.37	192	24.84	12.3	236.16	8.1	20.48	5.04	18.4	23.81	21.01	0	10.32	11.95	24.43	8.82	320	18.77	105	2950	15.67	499.76	249.75	1322.5	12.81	56.45	3.6	27.4	46322.5	15.14
p1368	D6	26.73	27.72	137.38	16.41	8.69	168.65	5.66	7.86	4.39	58.23	20.59	29.56	0	6.97	7.74	20.96	3.66	820	45.52	685	5320	67.79	325.22	230.73	1189.23	12.13	47.33	5.9	9.97	52499.3	11.91
p1366	Admission	38.13	14.39	59.17	3.38	0.65	920.25	0	0	17.93	199.25	6.84	125.7	0	672.64	0	16.54	0	210	31.83	261	1570	0.44	981.75	8339.07	6566.95	12.74	94.7	25.3	66.53	45604.7	21.53
p1366	Admission	28.58	9.9	31.46	2.99	3.5	25.06	5.08	0	2.24	142.6	7.09	65.05	23.07	36.21	1.06	16.54	0	1140	89.35	948	9120	93.48	361.36	885.08	2081.24	6.31	31.39	23.1	35.28	335977	7.01
p1366	Admission	23.92	15.73	59.17	8.37	7.12	58.09	6.67	2.78	1.81	125.44	9.34	56.35	29.59	396.01	6	28.06	0	1190	96.45	879	9180	92.38	273.19	867.29	1499.74	8.94	35.73	17.7	46.07	441994	7.01
p1366	D1	28.71	14.83	65.2	6.45	5	173.85	4.93	0	1.53	320.55	8.47	56.74	43.6	134.73	2.71	20.09	0	1850	155	1462	14840	170	302.7	796.6	987.62	7.44	33.36	4.9	83.49	537131.2	7.15
p1366	D1	19.19	10.8	37.65	3.76	1.68	49.06	3.74	0	1.67	59.51	9.54	30.17	11.58	0	2.71	24.43	0	710	65.21	520	5210	46.34	462.62	393.3	1423.84	5.99	31.39	17	86.87	431573.6	8.83
p1366	D2	27.27	9.9	34.61	11.82	1.1	107.7	8.38	0	2.24	53.68	9.75	155.21	0	1.51	0	38.29	0	0	0	0	0	0	6962.79	1822.78	4721.16	8.76	82.45	110	402.48	292906.8	25.83
p1366	D3	28.32	17.07	73.28	8.56	4.59	79.51	3.74	0	2.52	27.82	11.89	35.02	20.29	0	0	24.64	0	950	72.26	853	7490	73.31	2646.37	610.63	5752.09	10.64	56.45	189.9	127.3	229527.2	14.08
p1366	D4	36.84	21.51	87.38	9.13	5	142.61	4.93	0	2.67	27.08	14.7	30.07	18.97	0	3.43	24.64	0.23	1070	73.59	1054	8420	104	1446.37	685.48	2423.8	9.11	56.45	79.4	110.01	140914	14.15
p1366	D5	44.38	26.39	118.66	12.77	8.17	196.37	4.34	0	3.02	20.29	17.09	29.16	25.32	0	3.08	28.69	2.83	1110	75.91	973	7800	104	925.01	394.34	2392.52	10.64	57.89	15.9	68.47	47383	14.29
p1366	D6	41.93	37	161.61	14.5	12.04	336.68	7.82	4.28	3.63	14.51	18	25.12	9.6	0	11.45	27.42	5.25	420	43.03	121	3970	13.78	921.93	233.87	1913.71	11.28	66.84	9	84.94	44346.2	14.72
p1361	Admission	33.24	6.26	0	0	0	0	0	0	103.07	250.79	2.18	22.54	0	0	15.09	2.85	0	0	7.07	0	380	0	304.34	177.32	3205.69	0	39.8	33.7	67.99	100243.2	2.33
p1361	Admission	22.11	3.49	0	0	0	0	0	0	21.21	174.65	0	15.94	0	0	1.06	0.8	0	0	0	0	0	0	329.73	162.08	2033.1	0	41.53	33	47.05	171067.5	3.37
p1361	Admission	22.11	10.57	43.44	3.38	0.5	25.06	0.76	0	3.81	76.36	7.56	10.01	0	0	0.54	4.71	0	0	19.53	0	1690	0	333.21	145.56	1368.97	3.19	58.61	32.2	41.17	119671.3	4.56
p1361	D1	25.63	14.39	48.91	3.76	0.5	25.06	0.59	0	4.31	76.9	8.02	20.11	0	0	0	8.44	0	0	13.38	0	650	0	422.99	192.42	1423.84	4.58	70.43	30.4	35.77	105144.9	6.17
p1361	D1	31.34	17.07	73.28	7.41	3.64	75.96	3.74	0	3.95	95.33	11.33	11.16	3.84	0	1.94	9.4	0	210	43.58	0	3150	0	463.3	161.57	1579.77	5.31	73.61	41.5	54.37	61844.8	7.01
p1361	D3	35.63	7.63	7.37	0.69	0	9.53	0	0	3.02	268.57	3	116.94	0	0	0	8.68	0	0	0	0	0	0	500.57	491.93	1940.95	6.31	124.5	31.5	253.97	39351.8	10.93
p1361	D4	44.48	17.07	66.38	6.83	2.39	47.24	1.26	0	2.38	108.97	8.69	13.68	0.29	0	0	8.68	0	460	46.9	303	4620	16.06	388.2	238.84	1324.61	4.58	61.07	7.9	57.29	29260	6.03
p1360	Admission	43.9	10.8	98.6	5.68	0	21.26	0	0	1.89	291.97	4.97	58.23	0	327.38	0	11.06	0	0	13.5	0	0	0	1089.92	3770.54	4985.15	2.55	188.32	5.6	0	56093.6	24.77
p1360	Admission	29.86	12.37	75.52	11.82	3.91	17.42	1.58	0	2.95	81.16	10.75	43.08	0	102.53	0	11.52	0	0	16.79	0	720	0	426.3	379.72	3263.76	3.49	91.09	13.8	0	236885.4	16.12
p1360	Admission	34.39	14.39	66.38	11.05	1.68	32.55	1.26	0	2.45	56.12	8.02	41.87	0	83.43	0	15.19	0	0	5.49	0	0	0	362.78	608.78	2221.32	2.55	105.12	15.2	8.46	298058.4	17.46
p1360	D1	30.36	18.85	87.38	27.14	3.37	93.65	1.9	0	2.24	71.89	14.19	33.31	0	50.42	4.76	17.88	6.68	0	6.75	0	110	0	386.8	627.96	2742.49	6.63	137.93	9	17.48	245420.6	21.6
p1360	D1	35.18	15.06	61.61	6.83	1.68	43.6	2.37	0	1.89	60.96	10.16	24.27	0	15.57	11.19	11.52	0	0	8.04	0	160	0	425.75	709.08	1829.83	5.77	87.69	20.6	0	151213.1	14.58
p1360	D2	31.34	10.8	104.49	4.91	0	32.55	0	0	2.38	57.26	7.09	81.53	0	0	0	16.54	0	0	0	0	0	0	824.72	1107.94	2721.68	0	83.04	9.3	40.19	74417.8	25.55
p1360	D3	29.1	21.96	114.02	19.67	6.07	140.87	4.64	10.62	4.03	29.02	19.03	20.34	0.29	0.14	27.34	24.64	3.54	100	15.42	0	1740	0	383.71	250.75	1550.3	9.64	59.32	0	10.48	84671.5	11.49
p1360	D4	35.52	23.95	121.41	19.67	8.95	130.42	5.22	2.39	3.09	20.88	16.78	21.12	5.96	0	13.42	21.4	5.8	240	34.86	0	3440	0	321.13	292.72	1840.32	8.3	66.17	0	4.91	71831.9	12.47
p1360	D5	55.84	29.93	148.45	30.2	11.66	243.08	5.81	8.56	4.68	29.94	21.42	32.37	6.2	10.58	16.71	29.96	7.11	410	43.24	196	4590	14.43	320.69	415.68	2106.34	10.48	80.67	0	33.32	86380.9	17.46
p1358	Admission	46.09	12.6	156.73	17.95	10.25	104.19	12	74.62	7.33	92.82	8.91	31.87	7.2	212.84	49.47	21.83	0	250	7.19	95.42	450	34.4	587.39	908.04	1290.8	12.36	92.77	38.9	23.44	14727.96	14.44
p1358	Admission	96.23	22.62	231.05	30.96	16.59	170.39	17.68	103.69	13.25	179.23	17.7	99.18	12.25	233.97	67.04	31.01	0	530	17.12	224	1060	56.07	710.82	4892.27	925.9	15.75	94.15	27.5	63.62	53405.12	18.37
p1358	Admission	35.96	14.39	174.34	20.05	12.93	90.12	14.46	89.83	10.48	136.85	9.96	42.61	6.77	164.33	50.5	27.21	0	230	7.19	46.95	550	30.36	435.26	471.15	671.59	13.03	72.67	11.4	39.21	93645.94	12.75
p1358	D1	32.06	16.4	140.82	20.05	10.38	125.19	11.72	49.19	6.64	65.48	13.16	24.37	19.7	84.01	35.85	21.62	0	890	43.61	432	1730	86.86	357.66	211.43	555.52	10.14	60.02	11.7	0.77	84478.01	7.99
p1358	D4	35.96	19.29	126.82	18.71	10.25	132.16	10.34	40.25	5.47	21.37	17.7	16.9	7.84	50.14	28.54	24.21	0	470	20.5	176	760	43.95	381.74	175.79	356.43	13.25	60.72	0	10.98	125751	9.88
p1358	D6	32.54	19.29	137.38	19.48	12.3	135.65	8.94	34.81	5.91	28.83	18.44	16.18	15.58	53.23	29.72	23.13	0.23	870	35.44	345	1550	69.51	463.17	202.53	637.25	10.48	54.25	0	4.39	27867.93	9.67
p1356	Admission	43.22	27.27	117.74	18.71	8.43	100.68	5.52	1.18	2.38	19.5	12.98	8.67	25.49	5.44	5.39	11.99	6.46	410	72.3	162	5090	3.83	158.92	129.35	589.97	5.54	61.42	17	0	21378.1	4.56
p1356	Admission	37.06	10.35	61.61	6.45	1.1	25.06	0.59	0	1.32	9.29	8.69	5.27	7.37	0	0	4.19	0.23	160	37.22	0	2800	0	164.44	112.43	609.32	0	36.19	8.6	0	51445.7	0.39
p1356	Admission	48.12	8.99	43.44	5.3	0.18	9.53	0	0	1.04	8.48	6.34	2.98	7.14	0	0	2.57	0	330	49.55	183	4680	9.97	184.25	127.29	469.2	0	36.65	10.3	0	51471.1	1.43
p1354	Admission	52.07	9.05	55.05	10.12	2.42	63.49	1.5	2.25	1.03	17.9	16.36	105.08	8.31	9.23	0	6.02	5.43	250	59.05	0	3790	0	536.17	310.64	93	15.38	46.26	6	0	217432.6	5.4
p1354	Admission	55.57	6.99	52.18	8.75	3.47	76.28	1.14	2.25	1.52	13.4	17.48	83.34	5.25	6.56	1.05	5.67	4.96	150	51.85	0	2980	0	492.67	329.94	73.52	21.1	48.23	6.5	0	261685.8	4.92
p1354	Admission	109.08	2.53	32.52	5.15	0	25.34	0	0	0.04	29.83	12.6	175.3	0	2.29	0	5.67	0.9	0	16.11	0	540	0	700.29	594.3	344.89	4.16	37.59	12.9	0	224165.2	5.59
p1354	D1	67.76	5.45	35.84	4.29	0.52	39.17	0	0	0.46	22.08	13.06	97.08	2.19	4.18	0	5.67	4.01	150	35.2	0	2750	0	772.84	407.8	291.23	8.63	45.25	7.08	0	135472.1	5.4
p1354	D2	60.95	1.59	27.91	4.29	0	25.34	0	0	0.11	6.07	13.93	58.68	0	1.48	0	4.97	3.15	0	16.11	0	1420	0	665.56	318.65	254.43	0.29	41.02	9.66	0	120475.5	5.87
p1354	D2	36.23	2.81	8.7	3.26	0	48.42	0	0	0	11.71	0	1177.49	0	0.9	0	3.52	0.6	0	0	0	0	0	872.96	714.58	466.18	16.45	49.19	15.7	13.59	158061.7	8.22
p1354	D3	46.59	3.96	44.26	6.35	0.93	43.79	0	0.66	0.71	9.33	10.14	55.68	7.61	2.91	0	4.97	3.52	240	43.91	0	3790	0	930.69	304.14	154.43	7.57	48.23	8.3	0	103205.1	5.02
p1354	D4	53.25	3.96	32.52	4.63	0	25.34	0	0	1.09	7.09	9.03	107.76	0	1.68	0	7.04	3.52	100	26.37	0	1370	0	1666.51	466.87	434.85	4.67	50.13	15.4	49.49	122047.7	7.38
p1353	Admission	82.15	12.83	71.37	15.26	8.62	94.92	5.61	13.57	2.29	79.21	21.23	26.44	1.25	18.49	7.41	10.34	6.13	870	58.4	1476	6110	186	196.46	254.52	346.08	12.83	58.91	38.6	0	19174.4	6.53
p1353	Admission	52.24	14.52	81.6	14.23	8.62	126.48	5.14	12.51	2.52	32	20.59	19.45	3.84	9.46	9.29	12.88	7.04	1240	85.36	1448	8780	195	150.91	163.27	215.44	13.88	55.53	18.2	0	76329.6	7.85
p1353	Admission	59.4	11.17	66.08	11.83	6.91	118.29	4.82	11.8	1.94	54.07	19.59	25.49	0	19.2	9.29	9.69	4.96	1060	42.58	1554	5450	226	172.08	279.58	279.34	12	62.93	15.4	0	68008.8	8.79
p1353	D1	63.07	4.1	31.39	15.26	2.6	48.42	1.85	4.54	1.03	27.17	14.77	12.72	0	6.12	0	5.32	2.77	500	31.82	1151	3270	151	224.65	253.25	254.43	8.8	47.25	9.6	0	69489.8	3.96
p1353	D4	43.39	3.67	33.64	24.53	1.32	39.17	0	4.91	1.94	92.1	14.77	13.32	0	4.82	0	9.04	3.52	470	22.95	1023	2580	116	292.87	215.23	301.94	7.01	38.18	110.9	4.32	31065.2	5.21
p1353	D5	65.15	11.83	78.24	15.61	8.93	112.44	5.45	12.51	4.91	23.73	23.7	18.2	0	8.56	9.54	12.09	6.36	580	52.51	862	4890	105	306.84	263.2	341.3	13.62	57.24	15.4	12.64	20282.9	8.6
p1353	D6	83.03	11.83	64.29	14.58	6.11	85.6	4.18	12.86	6.51	19.9	22.49	19.61	0.53	7.44	3.44	9.04	6.36	680	56.44	1133	5030	112	317.52	246.96	422.82	11.72	51.98	7.8	21.81	12894.7	6.34
p1353	D7	77.76	28.59	139.24	48.64	21.11	271.2	27.2	33.16	14.04	215.15	38.91	42.88	0.53	115.84	14.84	24.59	14.17	730	55.79	1097	5150	125	3639.92	576.64	1816.52	26.03	119.58	9.6	93.02	113819.9	22.11
p1351	Admission	84.28	5.9	43.23	5.32	2.42	50.73	2.53	4.91	0.65	4.23	9.03	167.88	13.97	8.56	1.51	2.77	4.25	310	31.68	0	1410	38.03	737.43	310.12	361.63	8.63	52.88	15.9	14.54	18045.74	3.48
p1351	Admission	55.24	4.69	36.92	4.29	2.78	39.17	0.77	6.75	0.04	0	9.03	56.43	18.59	7	0	3.52	2	510	54.91	210	1950	66.6	771.12	200.68	471	7.39	45.25	14.2	0	77818.23	3.68
p1351	Admission	94.16	3.81	40.12	3.95	1.32	29.95	1.14	3.02	0.04	3.01	14.77	46.49	17.81	6.67	0	3.52	2.26	600	54.91	433	2380	76.03	1006.45	360.11	1225.31	8.29	45.25	18.8	10.68	169524.4	5.68
p1351	D1	103.67	5.14	20.46	3.95	0	23.05	1.14	1.86	0.19	4.04	7.82	36.1	0	7.33	0	3.15	0.6	110	14.56	0	0	18.69	1559.65	593.35	1132.3	8.63	51.06	21.5	102.66	170860.5	9.35
p1351	D1	34.46	2.95	30.24	3.26	1.51	20.75	0.39	0	0	0	6.46	4.74	4.99	4.5	0	0	0.28	320	26.95	59.76	770	43.9	672.23	69.68	133.47	6.01	29.81	14.5	0	86429.74	1.53
p1351	D2	71.7	3.38	14.97	1.89	0	13.89	0	0	0	0	9.03	5.06	10.11	6.34	0	1.98	0.6	530	40.71	195	1550	59.02	999.34	195.73	295.99	2.37	29.81	70.6	54.07	51198.05	4.16
p1350	Admission	63.67	4.55	27.91	3.95	0.52	40.32	0.59	1.47	0	0	7.82	5.16	21.56	6.34	2.72	4.61	1.73	1330	82.19	650	3790	131	220.75	228.82	263.92	5.14	45.25	15.1	0	4145.561	3.29
p1350	Admission	27.65	1.46	17.79	1.55	0	9.34	0.39	3.02	0	0	0	8.38	0	4.71	0	4.97	0.28	0	0	0	0	0	256.66	240.35	183.69	4.42	47.74	13.4	0	11550.93	4.82
p1350	Admission	63.07	1.2	20.46	3.61	0.3	16.18	0	0	0	0.42	2.77	8.56	0	3.96	0	3.89	1.18	0	4.87	0	0	3.82	180.73	242.42	191.91	2.71	39.9	11	0	15356.86	3.68
p1350	D1	67.19	2.53	17.79	2.58	1.51	20.75	0	1.47	0	14.49	7.17	16.12	4.41	4.71	0	4.61	0.9	200	25.49	0	620	32.04	449.9	252.93	266.29	4.16	48.71	9.4	0	28163.93	3.48
p1350	D1	37.74	6.67	56.93	6.01	3.98	62.33	2.02	5.28	0.71	6.41	11.17	10.2	30.69	9.23	0	6.02	4.13	1540	84.57	847	4170	140	205.83	98.21	323.39	9.62	67.51	16.2	0	66509.7	3.87
p1346	Admission	83.53	5.14	34.74	7.72	0	200.45	0	4.16	0	68.01	7.82	104.29	0	471.3	0	1.58	7.71	1080	32.04	478	950	71.34	340.23	570.36	473.42	4.67	39.9	22.1	0	28776.42	6.53
p1346	Admission	38.99	6.67	43.23	12.18	1.13	53.05	0.19	3.02	0.32	38.46	15.58	35.14	0	9.68	0	3.15	5.43	410	26.95	156	990	50.51	288.28	123.57	79.24	6.22	37.59	13.4	4.32	175622.8	3.29
p1346	Admission	33.54	7.3	51.21	10.12	0.3	41.48	0.39	2.25	0.59	33.7	13.06	20.69	4.41	7.89	0	5.32	4.13	520	34.58	313	1700	61.03	434.3	86.76	87.26	6.42	33.89	9.9	22.69	98246.29	3.29
p1346	D1	49.11	7.46	36.92	6.01	0	12.75	0	0.66	0	19.15	0	15.31	0	3.54	0	4.61	1.73	70	4.87	0	0	12.06	358.36	62.62	210.73	1.99	21.65	5.3	7.62	178599.3	2.12
p1346	D1	53.75	5.14	43.23	9.6	0	13.89	0.77	0.66	0.32	7.42	7.82	16.85	0	3.33	0	4.97	5.55	220	16.01	0	280	25.91	441.81	84.19	218.98	5.58	29.81	4.8	38.52	112733.1	5.02
p1346	D4	98.19	6.36	41.17	12.52	0.93	32.25	0.77	0.66	0	30.71	7.17	25.05	0	4.28	0	6.7	5.08	0	2.86	0	0	0	960.53	117.12	1034.58	5.58	31.22	17.4	0	16762.79	7.85
p1344	Admission	79.33	5.45	36.92	4.63	0.52	46.1	0	2.25	0.97	26.95	12.14	10.55	9.72	5.58	0	3.15	2.64	240	48.22	0	3670	0	119.4	137.16	114.92	6.01	38.76	13.7	0	58524	0.73
p1344	Admission	174.57	8.73	62.47	6.86	2.25	108.94	0.77	3.02	1.21	24.67	18.2	20.53	2.9	5.47	1.05	7.04	6.24	300	34.52	209	3240	10.9	228.53	201.74	443.28	14.89	52.88	10.4	148.38	49065.4	10.83
p1344	D1	95.32	5.9	45.27	4.98	1.88	55.37	0.96	0	1.09	20.14	13.06	8.38	29.67	7.33	0	2.57	2.77	610	91.69	444	7020	13.64	138.55	89.95	148.6	6.82	36.39	7.5	4.32	45600.5	0.32
p1344	D1	101.34	2.26	4.95	1.55	0	125.31	0	2.64	0	353.75	0	146.18	0	3.96	0	5.67	0	0	0	0	0	0	252.21	332.32	268.66	10.7	53.78	23.9	76.12	38730.5	10.09
p1344	D3	94.51	11.83	106.32	9.78	9.54	122.97	4.18	8.93	3.33	15.03	26	11.69	24.41	6.78	4.44	10.82	9.15	1060	111	1236	10250	78.48	186.25	123.23	335.33	15.86	63.71	14.8	88.85	27851.7	12.87

p1340	Admission	55.89	11.33	58.8	12.86	3.81	80.94	1.85	12.51	4.46	129.15	19.59	25.34	19.29	17.54	5.68	6.7	8.38	890	54.7	446	1900	81.61	83.18	314.52	145.1	10.85	61.34	26.6	0	20327.17	5.78
p1340	Admission	75.91	7.14	53.14	16.64	0.52	70.46	0.39	5.65	3.61	315.32	18.2	50.61	7.2	15.55	7.41	6.36	7.15	510	34.81	251	870	52.18	194.19	612.51	259.17	9.3	56.39	69.8	0	93619	4.06
p1340	Admission	52.24	8.89	66.08	13.55	5.14	97.26	2.53	8.21	2.41	193.43	20.26	35.75	9.74	20.03	5.38	8.38	8.05	780	41.11	390	1400	71.03	240.01	310.16	216.62	11.72	66.01	26.6	21.81	195238	4.82
p1340	D1	35.35	8.57	59.72	13.55	2.78	80.94	1.5	8.21	3.56	192.33	15.97	35.75	15.99	19.79	2.34	10.66	9.04	830	49.8	435	1550	77.85	219.96	370.38	260.36	11.14	76.71	9.4	38.52	239447.8	7.1
p1339	Admission	108.44	22.58	96.21	14.58	10.15	120.63	7.94	13.92	2.99	17.64	23.1	14.48	16.9	39	10.56	11.77	12.81	640	44.2	989	4900	93.9	509.52	280.47	471	17.93	77.72	25.1	12.64	12083.36	9.35
p1339	Admission	70.03	13.34	73.11	11.49	8.01	90.26	5.14	15.33	2.11	33.49	19.93	17.4	6.8	9.57	11.3	9.36	7.83	180	27.3	290	2600	26.1	354.89	191.82	336.52	14.89	85.78	16.2	0	32407.73	6.81
p1339	Admission	41.23	5.75	39.06	4.63	2.78	41.48	2.86	8.21	0.59	54.43	10.67	16.28	13	11.96	1.51	5.67	2.77	410	39.5	736	4200	60.3	325.03	149.81	408.4	8.11	57.24	106.8	0	40892.32	4.06
p1339	D1	59.71	6.99	51.21	7.89	4.81	50.73	2.19	6.75	1.58	47.13	13.5	16.28	5.7	12.42	2.72	7.38	3.02	240	25.5	527	2400	33.7	345.92	166.97	217.8	9.3	57.24	8.1	4.32	41817.69	3.29
p1339	D2	71.43	9.21	52.18	8.41	5.14	71.63	4.18	10.37	1.94	8.39	21.23	12.38	13.2	6.56	6.85	8.21	4.61	410	36.5	482	2900	53.8	263.82	146.66	188.38	12	56.39	14.5	3.13	57954.8	4.44
p1339	D2	88.1	8.09	47.28	7.38	4.81	71.63	2.53	12.51	1.34	10.24	14.36	11.43	8	6.34	6.56	8.38	4.96	370	28.2	510	3200	45.9	282.06	216.28	189.56	10.55	62.14	6.3	27	49632.29	5.96
p1339	D3	94.16	8.57	67.86	8.75	6.43	78.61	4.5	16.03	1.7	18.4	17.48	14.48	7.6	9.91	6.85	10.34	7.38	340	34.3	560	3700	51.4	1282.13	269.04	175.48	13.62	90.07	6.8	38.52	36028.98	8.22
p1339	D4	80.1	5.45	31.39	3.95	4.48	23.05	2.36	17.43	0.84	9.17	9.03	15.63	0	8.11	4.44	10.17	2	0	7.1	290	100	0	1645.81	513.93	1211.66	18.91	111.74	8.6	40.12	22178.87	12.87
p1339	D5	74.43	13.51	72.24	11.49	7.69	89.09	6.7	13.22	2.41	8.86	22.49	13.48	6.4	9.68	6.85	8.71	7.26	240	28.2	365	2700	33.7	621.11	190.15	798.31	15.26	75.69	12.05	5.46	22780.56	7.19
p1336	Admission	76.84	2.39	20.46	3.09	0	16.18	0	0	1.7	27.4	7.82	18.98	0	18.37	0	6.7	6.93	0	7.39	0	0	0	991.8	488.02	449.3	7.2	74.66	13.7	20.04	13745.7	8.41
p1336	Admission	122.09	16.41	81.6	12.86	8.78	108.94	3.36	7.48	2.99	32	24.86	24.08	17.99	7.67	6.27	10.82	8.82	1350	93.58	1331	10580	162	394.65	360.71	253.25	15.26	59.73	8.8	0	22734.7	7.76
p1336	Admission	124.68	2.26	20.46	1.55	0	13.89	0	0	1.21	44.62	6.46	20.3	0	2.19	0	4.97	5.66	0	6.75	0	0	0	807.42	674.98	323.39	5.14	55.53	10.466	0	31561.9	6.34
p1334	Admission	80.74	5.45	62.47	11.49	41.61	86.76	41.84	130.07	1.94	91.62	19.25	43.21	30.7	104.62	50.75	30.91	6.47	190	11.5	52.2	2700	18.8	407.44	424.89	695.57	37.22	59.73	24.2	4.32	30933.91	7.29
p1334	Admission	52.75	8.09	70.5	10.8	38.51	78.61	40.5	118.71	2.99	95.08	18.56	40.82	44.8	61.98	47.12	30.77	6.13	490	43.4	448	5400	67.2	292.16	236.11	307.89	35.15	56.81	23.9	35.3	134747.7	7.1
p1334	Admission	57.34	3.38	36.92	11.15	46.73	36.86	48.47	115.55	2.05	101.74	13.06	45.71	30	62.63	57.82	32.32	3.76	200	20.1	75.4	2500	26.1	395.29	263.83	951.93	38.23	60.54	24.2	42.49	185611	7.19
p1334	D1	68.62	4.25	36.92	7.38	49.77	27.64	44.23	163.25	2.41	116.93	10.14	69.08	38.5	87.33	62.53	42.07	3.52	290	14.7	224	2900	45.9	601.16	371.28	934.68	44.39	61.34	19.4	90.24	148609.1	13.61
p1334	D1	70.31	8.57	67.86	9.78	35.64	73.95	33.76	96.16	3.33	94.46	19.25	38.81	31.1	51.66	44.77	31.48	6.13	430	32.6	413	4000	47.1	352.4	280.47	481.86	32.7	68.98	38.3	53.31	80500.85	8.79
p1334	D2	78.15	13.51	91.43	13.55	34.2	133.51	32.13	99.99	5.91	76.06	28.42	39.35	51.8	47.32	46.45	31.19	9.37	790	56.3	866	6500	99.6	500.88	279.22	433.65	35.15	79.38	23.9	76.12	70705.47	13.98
p1334	D3	84.03	19.03	124.96	21.1	37.99	169.87	38.49	103.49	7.98	60.4	36.76	37.73	37.7	52.43	47.12	36.26	15.82	430	30.8	436	4400	62.6	703.89	252.07	648.02	37.22	76.37	26.6	51.02	77594.94	17.64
p1334	D4	111.84	10.67	97	14.58	36.69	139.36	35.66	120.61	7.65	87.41	27.36	61.28	46.5	66.16	44.6	36.81	11.21	530	32.6	516	5500	85.9	1179.47	397.4	761.57	37.15	76.71	17.9	141.91	59983.86	20.2
p1334	D5	110.57	6.06	47.28	12.52	36.95	53.05	30.22	106.99	4.12	100.2	13.06	41.89	31.5	65.5	38.58	32.61	4.72	150	12	251	2100	28.9	1259.92	311.08	1337.22	36.78	49.19	18.8	93.02	99589.94	12.32
p1334	D6	114.45	15.21	122.04	17.67	39.55	209.87	29.4	106.67	7.65	89.4	31.21	48.69	41.8	47.32	44.09	33.31	15.21	530	21.9	527	4400	76.7	673.24	279.76	742	35.9	87.02	16.8	265.53	126058	22.38
p1330	Admission	75.11	10.18	58.8	221.34	0.06	132.33	0.39	0.66	1.82	130.44	18.56	84.91	0	8.11	0	6.19	111.03	0	8.7	0	260	0	453.94	266.67	922.37	91.86	98.79	30.3	0	56695.3	19.11
p1330	Admission	47.32	12.67	70.5	138.04	4.48	70.46	4.82	9.66	2.52	39.78	20.91	38.54	18.94	9.57	3.78	9.04	61.64	990	82.83	806	9270	76.34	288.28	152.46	548.39	73.28	97.09	23.6	0	222313	9.35
p1330	Admission	60.95	3.96	30.24	60.56	0	31.1	0	0	0.71	55.99	7.82	73.51	0	1.48	0	5.32	84.34	0	4.88	0	0	0	431.79	322.85	800.76	36.49	80.04	26.3	0	222801	16.91
p1330	D1	78.15	8.25	73.11	399.55	2.6	66.98	1.14	8.21	2.29	56.81	23.1	69.02	0	11.05	2.34	10.66	90.47	240	29.1	170	4260	5.12	607.63	352.59	745.66	132.35	115.83	16.5	0	153977.5	16.27
p1330	D2	87	7.14	47.28	386.98	0	23.05	1.5	2.25	1.09	55.81	23.1	66.47	0	14.27	0	13.97	107.3	0	0	0	0	0	1171.45	573.61	1116.2	126.96	158.5	17.1	0	52268.3	38.05
p1330	D2	68.9	13.51	71.37	91.77	4.98	76.28	4.5	8.93	3.1	40.28	26	35.55	0	10.25	0.5	13.5	29.1	170	24.32	0	2010	0	984.13	495.78	378.4	53.75	104.86	17.4	0	50718	18.28
p1330	D3	41.83	3.67	4.95	11.83	0	23.05	0	0	0.32	64.81	2.77	86.07	0	4.28	0	10.98	55.91	0	0	0	0	0	1153.7	550.85	2213.97	18.04	74.66	25.1	0	35639.2	21.29
p1330	D4	55.89	22.94	66.97	179.9	6.11	115.95	5.77	19.17	3.22	134.57	26.82	65.81	1.96	12.42	11.06	12.4	57.89	320	39.9	209	3560	6.97	529.57	363.18	1231.52	63.6	134.8	38.9	0	57504.9	15.45
p1330	D5	62.47	19.73	100.92	107.27	8.47	153.44	9.15	19.17	6.07	177.94	33.37	71.66	0	15.55	14.38	20.78	40.6	210	26.71	0	2100	0	1152.99	429.69	1498.12	48.17	99.35	11.2	42.49	56755.5	20.74
p1328	Admission	66.87	22.75	205.21	48.77	24.46	142.3	33.97	202.29	10.13	141	26.56	75.85	28	107.68	124.79	26.87	7.83	1300	58.73	1426	11410	190	345.45	188.89	267	19.34	72.72	34.68	40.22	19096.4	4.83
p1328	Admission	51	19.03	183.99	37.11	17	147.41	20.89	141.6	9.34	104.84	26.56	55	27.28	95	95.14	17.3	8.32	940	46.9	1136	9150	112	190.3	85.85	220.62	16.27	66.07	30.71	33.88	23520.6	2.75
p1328	D1	37.54	20.68	181.96	40.6	19.15	124.55	26.93	151.86	9.58	105.22	22.55	58.18	28.12	78.91	101.94	21.25	7.59	1400	60.68	1503	12150	163	85.41	39.53	44.78	16.52	61.87	7.99	7.77	113645.7	1.11
p1328	D2	35.16	14.08	183.99	42.65	17.46	99.52	20.53	152.59	8.86	114.7	20.78	64.29	26.8	86.95	112.95	25.51	5.85	1400	47.23	1671	11750	227	91.89	37.59	67.06	16.4	58.97	44.86	9.32	143602.9	1.35
p1326	Admission	48.04	6.65	41.1	4.99	1.42	129.6	0	0	0.16	70.87	20.78	24.59	0	44.23	0	1.12	2.65	0	2.04	0	0	0	519.18	257.06	2797.55	0.55	52.87	21.7	0	52880.2	0.87
p1326	Admission	33.69	11.19	57.11	7.92	2.88	62.88	0	0	0.37	26.3	17.39	17.86	6.43	4.58	2.2	2.28	5.1	2230	125	1544	13470	228	424.84	136.86	1662.54	1.72	42.72	20.14	0	178551	1.11
p1326	D3	46.54	8.72	46.66	7.25	0.65	56.9	0	0	0.57	12.47	19.86	9.19	3.14	3.35	0	1.12	4.08	1900	95.46	1517	10430	225	372.75	166.16	261.22	1	46.27	7.72	0	62623.3	0.63
p1326	D4	63.23	13.67	58.11	7.92	2.52	66.49	0	0	0.51	23.07	18.41	12.94	7.61	5.1	0.5	4.53	4.84	2710	158	2242	15860	304	448.91	208.9	1108.38	1.56	49.64	7.17	0	41090.8	2.52
p1326	D5	43.92	14.49	74.33	12.53	4.1	90.87	1.02	5.78	1.04	5.86	29.12	12.52	4.9	3.35	0.5	4.53	7.09	1390	97.35	1257	11100	134	545.87	127.53	681.8	2.33	53.66	14.94	0	26020.8	3.45
p1326	D6	37.19	0.56	0	0	0	49.78	0	0	0	0	7.19	58.05	0	0	0	0	0	0	10.69	0	0	0	674.95	174.74	2559.41	0	69.11	16.75	0	20228.2	0
p1325	Admission	56.73	14.9	70.64	13.83	5.8	170.54	1.84	7.74	2.45	71.19	32.18	56.73	21.91	198.08	8.45	9.41	8.45	1070	110	656	8510	52.77	169.66	401.04	452.23	6.74	71.09	22	22.53	262549.6	7.33
p1325	Admission	62.84	16.97	90.2	12.97	8.28	130.87	4.28	10.13	3.08	21.25	28.77	40.72	26.09	66.79	9.23	9.59	10.75	1070	120	926	9080	65.79	109.15	230.55	168.05	7.18	62.23	21.39	46.31	392430.8	6.42
p1325	Admission	58.32	18.62	86.76	15.98	6.63	120.77	5.63	10.6	3.96	21.25	30.17	26.1	29.44	41.28	11.96	10.3	11.94	960	121	606	9970	60.84	101.49	213.85	253.5	8.03	56.73	17.6	27.21	364734.6	5.05
p1325	D1	68.95	9.75	60.1	11.66	1.42	62.88	0	4.27	2.45	32.96	26.56	17.86	53.61	14.93	6.56	5.63	4.84	1200	161	1573	15240	99.45	107.35	251.03	107.02	5.03	45.83	14	6.14	139092	1.11
p1325	D2	57.53	19.86	104.33	17.26	9.26	124.55	6.29	22.32	4.95	26.3	36.55	14.07	37.97	15.26	16.8	10.66	10.63	1100	141	772	11260	47.28	184.98	163.15	170.01	8.69	55.97	13.75	9.32	42276.8	5.4
p1325	D3	80.34	2.48	3.28	0	0	149.96	0	0	0	75.5	0.62	152.95	0	0	0	6.18	0	0	2.84	0	0	0	176.31	459.65	484.41	2.33	51.27	120.73	94.93	27438.9	5.74
p1325	D4	46.76	16.97	93.59	16.84	9.1	119.51	5.41	17.91	5.08	81.37	38.31	12.73	27.52	12.59	13.85	12.42	12.18	1150	139	839	10670	67.46	134.96	137.88	15.82	9.01	58.23	10.8	12.23	24575.8	6.65
p1325	D5	34.67	1.21	3.28	0	0	104.49	0	0	0	119.98	0	93.38	0	2.46	0	7.08	0	0	8.04	0	0	0	143.36	277.75	299.7	0	47.55	19.5	83.08	53280.2	6.42
p1325	D6	48.15	25.65	117.05	26.96	15.75	205.72	11.46	37.62	6.67	65.72	47.31	21.14	28.6	19.75	18.34	17.99	15.14	1780	159	1248	13570	115	183.37	160.63	142.56	12.25	68.78	16.45	67.18	25285.9	11.84
p1325	D7	53.79	4.99	59.11	5.45	0	46.25	0	0	0.37	40.75	21.24	4.77	11.85	1.55	0	4.9	4.08	2140	169	1552	16020	166	235.35	224.68	274.71	1.72	33.34	20.76	0	14140.5	0.39
p1324	Admission	71.57	11.61	69.7	10.79	4.95	149.96	3.34	5.78	1.62	143.24	24.22	36	7.6	548.81	4.82	9.24	7.59	420	30.8	224	4600	72.8	274.06	593.82	890.35	9.32	52.07	26.8	23.72	12652.9	8.01
p1324	Admission	67.63	7.89	30.25	4.07	0.65	50.96	0	0	1.3	266.95	15.77	89.23	2.9	19.25	0	8.52	2.51	190	16.9	0	1800	19.5	415.9	779.2	395.26	4.4	36.99	11.4	0	258357	4.37
p1324	D1	39.87	4.16	30.25	2.67	0	30.02	0	0	2.96	262.77	18.9	56.47	8	18.76	0	13.12	0.87	300	21	83	3600	53.2	392.58	582.35	339.94	4.51	28.81	9.68	26.06	320167.4	6.42
p1324	D2	44.14	12.02	61.08	8.14	1.04	81.05	0.72	0.41	4.33	122.87	22.97	28.31	26.6	18.26	6.56	18.85	5.85	890	62.8	917	7300	89.6	280.5	423.73	320.8	8.2	40.87	7.44	54.13	135673.7	7.78
p1324	D3	38.71	4.78	8.39	0	0	97.04	0	0	2.96	64.85	26.56	395.04	0	8.87	3.57	17.99	0	0	0	0	0	0	441.8	1015.11	1123.11	5.62	36.99	6.9	234.64	74817.72	18.96
p1324	D4	54.19	26.06	110.75	20.24	12.62	193.94	9.04	20.56	4.58	45.94	37.14	33.42	11.7	15.76	15	19.02	13.61	310	35.2	0	3600	48.4	295.15	265.42	446.54	12.73	52.87	6.9	67.18	32443.72	12.06
p1324	D5	58.22	31.46	149.92	26.96	15.91	281.59	13.03	31.76	7.28	56.95	44.29	38.38	12.2	23.55	15.68	24.83	20.59	270	23.7	224	3600	40.8	384.38	275.15	709.84	14.65	66.75	5.82	105.62	31913.01	18.52
p1324	D6	55.52	31.46	159.18	28.21	16.22	285.64	13.42	34.29	6.12	29.41	52.95	37.94	12.2	22.89	18.12	26.53	19.67	260	24.6	83	3600	43.4	451.33	174.43	604.98	14.9	65.38	6.35	117.64	27935.67	22.05
p1321	Admission	66.01	9.13	48.82	12.97	1.79	42.73	0	1.6	0.23	7.62	12.75	8.95	13.09	16.76	1.82	2.85	4.84	1090	71.02	1112	4550	98.77	306.32	467.22	380.03	3.37	40.87	9.68	0	5401.934	2.52
p1321	Admission	38.94	21.51	100.24	19.18	11.83	109.48	8	16.11	3.34	42	32.83	16.92	8.47	11.58	11.72	14.52	11.7	410	42.36	401	2020	32.79	396.66	215.94	201.2	8.85	55.97	6.62	0	11995.05	5.74
p1321	Admission	31.44	15.52	74.33	14.26	5.29	84.72	3.81	15.66	2.9	32.15	33.47	13.56	11.42	12.25	10.24	9.95	8.08	680	50.72	698	3110	43.95	319.01	167.03	79.12	7.53	50.05	4	0	31728.43	4.94
p1321	D1	37.78	13.46	74.33	12.1	4.95	77.39	2.36	13.38	3.02	58.42	25.02	13.35	18.06	9.89	6.84	6.36	7.34	990	68.62	829	3780	74.07	303.33	275.98	99.07	7.01	44.52	4.25	0	44866.67	3.21
p1321	D2	52.24	13.67	74.33	18.75	6.3	79.83	5.19	18.8	3.59	18.42	33.47	11.23	25.43	8.19	9.74	10.83	10.51	1040	74.61	946	4430	83.12	418.57	212.09	132.72	7.53	50.05	6.35	7.77	21960.54	5.05
p1321	D2	50.37	20.48	105.14	20.66	12.78	124.55	6.51	28.36	4.95	15.79	40.56	13.87	13.09	13.93	17.46	13.99	12.42	590	47.33	487	2470	45.53	409.39	189.95	109	9.09	58.6	7.17	33.88	14644.23	8.91
p1320	Admission	62.16	4.16	19.07	1.21	0	30.02	0.02	1.6	0	183.27	0.62	64.29	0	270.35	0	1.9	1.43	170	13.12	41.35	0	34.62	126.91	1374.34	1480.42	6.29	90.19	12.27	9.32	9897.539	13.74
p1320	Admission	44.8	9.96	50.94	6.58	0.65	143.58	0.02	0	0.37	82.28	17.9	47.94	9.58	29.15	0	6.72	5.1	790	43.93	460	1720	85.33	134.64	654.66	368.59	5.53	50.46	12.5	22.53	190370.6	8.12
p1320	Admission	41.8	2.48	19.07	3.61	0	39.24	0	0	0.09	143.07	12.07	51.64	8.89	21.41	0	3.04	1.15	480	37.11	187	1330	54.99	127.56	717.52	286.25	1.72	36.99	10.8	24.9	230042.6	3.91
p1320	D1	42.81	9.13	55.08	6.35	1.42	50.96	0	1.6	0.23	136.15	17.9	44.37	8.2	10.9	0	7.44	3.82	460	33.49	90.57	1700	45.56	157.97	466.61	101.06	2.87	36.99	14	108.05	202028.8	3.45
p1320	D4	31.44	2.9	6.07	1.21	0	34.61	0	0	1.69	81.17	10.64	127.35	0	3.35	0	6.9	0.58	0	0	0	0	0	464.68	938.67	1527.83	1.88	43.62	9.39	286.8	28236.82	15.63
p1320	D5	42.02	2.48	15.87	0.7	0	21	0	0	1.69	58.98	3.34	52.86	0	3.88	0	4.16	1.43	0	4.24	0	0	6	266.43	814.24	971.83	0.55	26.34	5.03	190.49	18477.4	6.42
p1320	D6	30.16	3.32	26.29	2.67	0	38.08	0	0	1.94	23.96	3.34	29.35	0	2.28	0	1.51	1.43	0	8.88	0	0	11.08	225.23	395.01	438.96	1	26.34	3.74	143.87	18411.56	2.75
p1319	Admission	53.68	4.99	31.52	10.79	76.13	48.6	16.28	35.12	0	602.86	19.86	85.1	5.18	1303.5	19.2	15.56	17.36	0	1.99	0	0	0	601.45	4537.33	791.88	15.66	122.19	20	32.79	18734.1	16.08
p1319	Admission	63.81	2.48	15.87	3.14	6.3	880.25	2.36	4.78	0	1398.19	0.62	145.5	3.29	62.21	6.56	7.8	3.3	0	4.63	0	0	0	587.23	4102.68	1427.47	8.85	87.62	20.76	69.89	256312.2	14.52
p1319	D1	32.07	5.41	37.62	9.03	6.63	41.56	5.85	10.6	0.64	163.7	17.9	45.78	11.77	55.18	11.96	15.04	4.33	960	63.71	940	9020	126	596.78	507.31	463.59	7.7	46.27	14.04	114.47	98132.8	7.78
p1318	Admission	63.42	15.32	63.04	11.88	10.87	99.52	11.46	32.6	0.64	22.77	21.24	21.14	40.19	20.41	12.2	10.66	7.59	1830	97.37	1036	5760	179	155.04	315.07	299.7	7.7	47.12	17	6.14	19835.77	3.45
p1318	Admission	48.47	12.64	57.11	9.47	8.93	139.75	9.45	24.93	0.44	80.15	21.68	46.97	35.84	80.88	11.72	7.8	5.98	1460	92	881	3720	154	162.29	545.46	197.31	6.83	56.35	12.5	34.95	56149.53	4.83
p1318	Admission	57.93	3.74	22.07	4.07	3.93	30.02	5.63	18.8	0	166.83	12.07	38.46	28.24	50.77	5.71	6	0.87	1000	74.34	596	3660	113	173.27	595.93	332.29	3.13	33.88	14	16.29	164827.1	0
p1318	D1	40.55	10.57	46.66	8.14	5.46	53.33	7.15	20.56	0.3	40.25	15.2	20.43	32.13	11.41	9.23	7.44	3.82	950	77.08	610	3830	121	142.13	246.25	164.14	5.13	37.98	10.25	111.27	193460	1.7
p1318	D2	53.38	16.55	76.15	13.4	8.45	109.48	10.26	26.65	1.3	14.07	29.47	16.15	17.22	12.59	13.85	10.66	8.57	600	53.5	317	2050	61.83	828.06	170.01	776.99	7.18	52.07	8.55	87.36	48444.72	4.83
p1315	Admission	60.5	5.2	44.47	6.8	0	46.25	0	0	0.51	11.55	25.02	15.76	43.2	8.7	0	6	2.25	1480	80.3	1273	11700	160	1130.97	276.55	2641.85	0.55	50.87	50.76	55.09	13695.88	9.59
p1315	Admission	56.52	3.32	19.07	2.67	0	117	0	0	0	284.94	16.86	100.59	9.3	40.3	0	6.72	1.15	480	29.9	459	5000	106	2478.58	1165.97	1629.81	2.18	98.47	42	42.27	55311.19	11.84
p1315	Admission	51	2.06	30.25	2.67	0	34.61	0	0	0.23	238.83	18.9	52.05	2.4	14.6	0	4.53	0.87	190	19.2	0	3700	34.3	1060.26	424.96	1471.29	0	46.7	32	34.95	127514.3	6.88
p1315	D1	38.71	6.03	37.62	4.76	0	43.9	0	0	0.23	124.48	18.9	25.27	9.5	12.59	0.99	6.9	2.25	350	35.6	20	3600	47.1	810.78	254.35	456.02	1.72	37.98	13	102.36	142806.8	4.14
p1315	D6	52.86	27.72	127.8	26.96	17.3	187.41	13.8	37.62	6.06	28.43	52.04	27.09	29.8	20.58	16.36	21.42	14.9	640	52.2	560	6800	87	697.89	135.51	529.72	11.69	70.76	8.27	140.09	18147.77	10.83
p1314	Admission	66.97	6.65	45.57	7.25	0	50.96	0	0	0.37	64.19	16.86	23.06	26.85	101.91	0	3.42	3.82	2470	80.56	1247	5360	250	313.11	644.23	942.23	1	79.62	16.15	0	6770.638	6.2
p1314	Admission	32.82	12.22	63.04	21.08	0.85	73.74	1.84	10.6	2.33	35.35	29.47	18.33	35.93	6.48	4.82	7.8	8.08	1170	58.36	430	3140	90.23	171.89	188.53	210.92	3.13	47.12	8.27	0	54405.44	5.74
p1314	Admission	31.95	10.37	71.57	15.98	2.16	72.53	1.3	9.66	1.88	37.7	29.47	17.3	29.48	4.92	2.2	10.3	9.78	1120	54.4	503	3330	114	152.39	159.68	173.92	3.13	48.81	7.17	0	74007.75	7.33
p1314	D1	43.59	15.94	65.92	15.12	4.95	99.52	3.34	10.13	2.9	31.34	34.1	16.54	32.31	11.91	4.82	10.12	8.57	1050	67.12	422	2810	83.12	193.98	229	54.94	4.72	52.87	11.4	7.77	46707.81	5.05
p1314	D2	28.06	6.44	47.75	12.1	0	64.08	0	5.28	1.69	19.69	24.22	7.74	17.23	4.4	0	6.72	5.1	600	35.44	139	1520	51.8	170.21	126.14	24.2	2.04	36.99	4.77	0	17536.19	2.52
p1314	D3	38.36	16.14	91.05	17.69	7.63	124.55	3.81	15.21	4.21	17.12	35.65	16.15	37.93	11.24	11.23	11.72	8.93	1120	75.8	503	3630	90.6	180.13	156.43	150.42	7.27	54.82	6.9	29.48	19941.83	7.78
p1314	D4	39.41	22.34	127.05	21.08	14.04	186.11	7.15	24.93	8.25	18.74	51.58	20.52	31.5	14.09	15	20.22	13.37	1070	71.92	390	2460	93.2	215.39	132.51	128.78	10.83	65.38	6.62	52.2	11334.34	10.94
p1314	D5	32.7	4.16	27.63	2.43	0	72.53	0	0	7.4	7.41	18.9	82.99	0	0	0	5.27	0.43	220	0	25.21	420	22.16	360.4	432.46	614.36	2.87	61.51	11.98	0	9787.261	8.24
p1314	D6	43.7	32.5	140.48	29.46	18.23	241.41	12.05	42.58	11.21	28.01	57.33	28.39	40.73	22.73	18.55	22.11	19.44	1310	86.78	645	4250	123	272.72	183.4	218.68	13.47	79.62	6.35	94.93	10326.7	17.41
p1313	Admission	47.83	5.41	35.23	7.25	17.46	39.24	21.97	61.13	0.01	60.1	20.78	43.52	17.38	304.99	20.27	16.6	13.84	1490	52.44	1540	3940	235	945.64	970.14	2367.31	18.13	159.57	56.3	81.35	13584.8	28.21
p1313	Admission	31.44	7.06	44.47	7.7	8.93	54.52	11.06	28.36	0.09	40.5	15.77	30.86	33.83	23.22	13.62	8.34	9.3	2040	87.47	1730	5820	298	468.73	236.91	734.11	7.18	36.99	39.05	52.2	152452.9	3.21
p1313	Admission	30.16	13.05	69.7	10.35	9.58	82.27	9.85	23.19	0.64	38.98	19.86	22.19	44.19	19.59	12.2	9.59	10.51	1170	106	1013	5300	163	321.52	133.6	179.77	6.65	40.87	27.45	0	192595.8	2.98
p1313	D1	33.94	13.25	75.24	12.53	10.23	79.83	9.85	23.63	1.04	37.57	22.97	19.16	78.89	22.23	11.23	13.12	11.94	4230	179	2970	12800	474	371.17	144.94	359.05	7.36	44.52	12.27	17.58	160217.7	4.71
p1313	D3	34.91	9.13	59.11	9.69	8.93	62.88	11.26	26.22	3.08	28.57	16.86	16.44	40.91	16.26	13.15	10.3	9.42	5140	110	3698	16160	543	384.57	215.49	561.74	7.7	38.96	6.35	13.62	23916.23	6.2
p1313	D4	41.91	17.38	90.2	18.12	11.83	139.75	13.61	28.36	5.69	23.66	31.52	19.61	72.71	17.26	19.42	15.56	13.61	4410	101	3039	14120	457	367.99	190.43	418.08	9.09	55.21	7.17	54.13	21284.58	10.72
p1313	D6	53.68	28.14	149.2	26.96	20.98	228.13	23.4	57.15	6.79	54.67	45.56	32.96	42.7	30.47	28.21	28.05	20.82	2710	73.1	2350	10350	377	441.62	220.16	1165.43	12.59	73.36	11.1	176.23	18865.83	21.83
p1312	Admission	54.5	7.89	30.25	5.9	3.23	65.28	6.51	8.7	2.96	334.82	19.86	139.62	5.71	28.17	5.42	13.47	3.04	0	2.98	0	0	0	998.55	1604.76	1253.58	11.41	77.15	24.2	161.71	11134.2	15.96
p1312	Admission	52.45	2.69	12.37	4.53	9.58	492.48	8.62	18.8	0	183.03	0.62	79.65	0.79	1150.02	8.71	11.19	2.25	0	0	0	0	0	913.75	1157.93	2469.7	12.66	101.66	28.7	0	73781.6	14.52
p1312	Admission	37.78	6.24	24.91	5.45	1.98	48.6	6.51	4.78	5.57	1067.16	21.68	179.57	2.47	81.53	3.57	13.82	2.78	0	8.27	0	530	0	901.04	1761.88	1034.64	7.87	53.66	10.82	47.3	72241.6	7.44
p1312	Admission	33.2	5.82	24.91	3.61	3.93	34.61	11.85	19.68	10.19	1084.31	15.2	178.71	1.92	162.7	6.84	19.54	2.51	70	8.9	0	160	0	891.42	990.2	938.52	6.47	46.27	7.17	152.85	90146.7	9.37
p1312	D1	28.06	8.1	43.36	14.26	4.61	77.39	13.8	17.91	17.24	482.35	25.8	91.31	10.72	70.72	8.19	20.39	4.72	650	66.44	752	6300	90.78	1479.47	740.48	1143.36	8.53	43.62	9.68	254.71	97290.3	7.9
p1312	D3	29.38	7.27	41.1	11.66	4.27	50.96	5.63	18.35	5.08	80.55	26.56	58.05	0.79	49.13	4.21	19.71	4.97	90	20.97	92.14	580	2.74	1292.77	464.25	1314.06	6.83	43.17	5.29	605.92	73373.3	10.94
p1312	D4	33.2	14.9	86.76	17.69	11.51	114.49	10.86	29.64	4.95	37.7	41.65	45.99	5.98	27.18	11.23	18.16	11.47	550	47.93	649	4570	79.73	1148.78	320.12	355.23	9.16	47.12	6.62	260	65442.8	8.01
p1312	D5	32.45	12.43	81.52	15.98	9.42	102.01	7.79	30.91	4.7	43.49	38.88	35.1	8.89	21.9	12.68	16.26	9.3	760	56.18	981	7320	124	1121.59	270.98	758.35	9.78	45.4	10.53	128.59	43443.5	5.17
p1312	D6	31.69	16.14	96.94	22.77	10.55	125.81	11.46	35.96	3.96	66.48	41.38	37.94	5.98	28.17	14.08	20.39	12.18	380	41.37	300	3170	31.57	1008.28	251.41	313.13	9.94	47.12	4	164.64	28319.1	10.27
p1310	Admission	160.21	0	0	2.57	0	0	0	0	0.41	41.91	0	13.28	15.49	47.7	0	0	0	1580	89.73	1568	4110	231	161.4	902.18	609.75	0	41.44	17.1	0	9950.506	4.19
p1310	Admission	113.98	10.77	47.48	17.64	5.63	46.38	2.31	4.43	6.28	212.58	4.55	42.34	74.7	11.47	0.61	7.64	9.54	2310	148	1984	8050	274	201.71	925.33	367.27	0	50.62	7	0	59350.62	8.1
p1310	Admission	82.58	16.19	45.63	17.64	7.02	43.63	2.93	3.27	4.41	111.02	8.54	25.9	76.19	18	2.3	10.1	11.96	3930	191	2544	10560	445	267.28	444.63	391.48	0	43.79	6.2	17.71	65192.38	7.59
p1310	Admission	70.28	10.43	43.76	15.87	5.48	35.43	1.48	2.78	3.62	32.59	5.59	18.15	16.6	13.15	1.16	6.36	9.62	1090	63.01	1061	3090	156	178.52	331.13	426.61	0	39.04	9.1	3.5	62406.18	7.42
p1310	D1	40.94	3.33	23.87	8.64	0	7.08	0	0.06	1.48	16.99	0.29	10.66	26.08	4.27	0	1.27	4.36	1250	124	1159	4060	164	172.2	225.02	187.84	0	34.33	5	0	66205.38	2.61
p1310	D2	56.26	2.57	13.22	5.66	0	7.08	0	0	1.38	13.92	1.81	7.2	36.76	2.64	0	0	2.98	1410	111	1418	5320	203	224.02	258.55	287.93	0	34.08	3.5	0	45380.25	1.83
p1310	D3	89.49	12.48	46.25	14.08	2.89	46.38	1.65	1.67	3.42	11.98	6.38	10.43	61.93	8.38	0	5.2	8.53	2900	162	2088	8610	337	225.77	286.79	342.81	0	43.32	2.44	0	31492.74	7.25
p1310	D4	48.86	4.81	23.15	8.46	0	31.81	0	0.46	1.64	9.09	2.92	6.54	21.02	4.59	0	0.25	4.88	730	57.27	804	3090	109	215.69	198.2	437.71	0	43.32	1.7	0	23703.07	3.18
p1310	D5	89.25	2.95	25.29	9.74	0	14.89	0	0	0.74	7.23	3.41	8.24	20.14	10.54	0	2.89	3.85	1310	75.43	1299	3460	166	330.57	299.82	643.23	0	39.04	5	0	19795.8	3.92
p1309	Admission	46.56	12.14	52.94	10.84	5.91	276.64	4.38	12.12	4.12	71.3	16.07	12.87	10.72	112.08	2.89	5.03	8.11	290	34.8	74.18	3390	6.63	251.71	240.24	437.71	0	38.79	60	3.5	40052.5	10.1
p1309	Admission	46.01	20.54	56.51	20.62	7.82	78.07	6.38	8.56	3.42	33.15	16.74	12.78	14.76	15.73	1.99	8.27	13.38	450	48.62	239	4450	16.77	244.24	143.8	509.78	0	35.59	26	25.16	130185.3	8.85
p1309	Admission	50.66	20.21	62.37	19.05	10.14	65.85	7.27	12.12	4.12	36.35	17.67	12.78	9.68	17.39	1.34	8.89	11.8	260	40.33	104	3280	2.74	268.52	153.81	442.45	0	39.28	19	16.45	79920	10.43
p1309	D1	50.24	23.37	86.44	26.01	16.57	131.93	9.84	23.4	7.93	20.71	21.71	14.87	2.47	26.37	8.35	13.5	15.78	0	6.1	0	580	0	406.15	152.6	140.17	0	45.41	13	42.74	52381.3	13.27
p1309	D1	35.32	11.8	48.7	15.15	6.19	55.61	2.93	9.26	3.32	12.92	13.26	6.16	5.18	10.85	3.18	4.34	9.29	120	19.6	0	1510	0	312.93	117.88	218.65	0	28.85	6.5	0	36458.4	5.17
p1309	D2	52.6	18.54	64.1	19.05	8.87	100.93	5.99	12.12	5.01	18.76	18.45	11.81	3.56	17.24	7.13	7.48	12.46	100	14.83	0	1160	0	226.92	141.06	270.95	0	37.08	11	27.61	40206.3	11.74
p1309	D3	77.56	28.69	80.97	21.15	13.91	108.62	9.24	10.32	4.81	44.35	20.11	15.86	7.57	20.25	2.6	11	16.36	180	22.34	0	2160	0	276.89	238.27	71.25	0	44.26	4.5	39.74	56353.5	12.87
p1309	D4	46.01	31.5	91.84	25.67	16.46	145.68	11.36	20.39	6.96	82.63	26.51	17.21	9.94	27.7	7.87	17.34	18	230	26.49	18.52	2570	2.36	242.54	149.69	279.47	0	47.47	2	43.34	35698.7	13.27
p1309	D5	54.25	34.49	97.18	24.97	15.66	163.49	11.6	20.39	8.27	27.94	24.91	15.23	11.51	23.54	8.35	16.77	17.59	280	32.72	182	3390	9.05	241.83	138.1	53.53	2.57	50.62	7.3	53.46	37168.4	15.83
p1309	D6	72.48	4.44	19.48	8.64	0	26.42	1.82	2.78	1.06	22.24	11.31	11.58	2.47	7.6	0	1.71	4.62	80	8.27	0	900	0	298.14	265.98	354.26	0	25	16.8	0	24091.9	4.28
p1308	Admission	57.13	8.01	19.48	10.84	1.67	68.66	1.65	2.46	1.79	109.42	8.2	18.83	19.03	148.3	0	2.32	6.08	560	65.42	392	5470	43.47	197.48	465.79	357.52	0	39.52	18	0	10811.8	2.61
p1308	Admission	53.26	11.8	39.98	13.37	5.34	46.38	1.98	5.61	2.71	62.79	10.68	23	37.91	13.3	0.99	3.99	7.09	910	106	686	9850	71.41	146.95	271.69	150	0	41.44	62	0	53205.4	5.35
p1308	Admission	160.43	3.89	4.02	2.17	0	12.27	0	1.67	0	142.69	0	48.99	10.46	24.44	0	0	1.57	480	58.24	284	4910	25.55	137.86	980.41	292.99	0	33.05	28	0	65402.8	1.43
p1308	D1	28.78	12.82	49.92	16.58	6.33	65.85	3.52	11.76	3.92	16.58	12.96	9.04	22.51	13.45	5.35	8.58	9.54	400	58.92	274	4960	19.14	137.69	128.16	161.57	0	34.08	3.5	20.83	87649.5	4.64
p1308	D3	51.85	9.57	33.49	14.26	4.16	50.07	1.3	4.76	4.12	19.71	10.68	11.49	15.54	11.47	0	3.99	6.59	520	52.75	483	6130	51.73	283.43	185.87	239.84	0	41.44	6.5	0	20500.2	9.6
p1308	D4	39.36	23.04	92.91	28.94	16.23	125.1	10.19	24.92	11.29	16.3	26.4	9.55	12.81	27.56	10.83	13.35	14.7	180	34.1	0	2400	0	293.7	126.7	209.69	0	59.7	10	32.49	13775.5	11.82
p1308	D5	42.49	15.86	74.88	25.67	11.63	99.01	9.24	18.9	11.1	10.29	21.59	9.6	13.07	18.45	5.87	9.35	11.63	160	32.03	0	2570	0	243.95	137.23	341.17	0	51.51	8.3	30.06	16353.1	12.14
p1308	D6	43.53	30.01	104.56	29.97	21.02	134.87	12.29	26.25	11.96	12.63	28.07	12.46	24.18	29.19	12.77	17.48	16.6	360	63.03	92.14	4740	7.68	244.1	127.21	372.13	1.94	60.96	9.1	52.86	12585.4	18.2
p1308	D7	38.96	22.88	74.32	24.63	13.08	108.62	9.48	21.33	6.72	10.58	22.19	10.34	25.98	17.85	8.35	13.64	13.29	320	65.08	110	4480	6.12	248.9	111.6	386.66	0	49.73	18	15.82	10468	12.22
p1307	Admission	116.71	1.37	22.43	10.11	0	18.41	0	3.43	1.48	37.45	0	16.67	0	41.56	6	10.71	2.98	0	0	0	800	27.5	474.52	497.38	1290.72	10.24	93.62	29	0	7779.344	9.85
p1307	Admission	61.3	5.71	23.87	12.29	0	19.29	0	2.46	1.06	40.35	2.11	17.3	9.3	11.77	1.99	5.37	4.36	630	29.1	493	5300	97.5	221.4	236.17	393.09	0.4	37.57	25	0	348960.7	3.09
p1307	D1	59.93	3.71	22.43	20.1	0	19.29	0	1.2	0.63	31.85	2.39	19.9	0	10.54	7.74	6.52	5.73	170	12	0	2800	36.3	419.82	298.67	246.82	0	68.35	26	0	240831.2	7.93
p1306	Admission	49.55	6.25	29.45	7.16	6.33	113.45	6.25	9.97	1.17	47.48	6.38	11.58	20.3	114.11	4.56	5.7	2.11	360	35.6	83	3700	39.6	104.17	199.89	103.08	0	36.58	11	0	23311.9	0.31
p1306	Admission	69.79	17.87	44.39	14.62	12.6	107.66	12.4	14.66	2.2	39.51	11.31	18.02	27.3	36.27	5.61	13.93	6.42	660	52.6	716	6700	101	131.01	175.38	155.81	0	63.04	18	33.7	83275.74	5.61
p1306	D1	26.25	1.98	14.84	5.28	5.34	63.05	6.64	11.76	0.63	19.71	3.65	7.96	10.5	22.05	4.29	7.95	1.93	120	17.9	0	2200	14	106.33	90.34	63.86	0	22.7	4.5	0	158546.1	0
p1306	D2	57.76	1.58	13.22	2.96	2.21	36.34	2.31	5.78	0	12.77	0	7.58	23	10.23	2.89	2.89	0.02	400	44.2	257	3300	49.6	121.81	123.81	89.87	0	34.08	40	16.45	131676	0
p1306	D3	76.38	8.7	27.39	8.64	6.33	52.83	5.99	8.2	1.79	10.73	8.71	8.34	14.8	14.52	5.09	4.69	3.5	150	22.8	0	2100	17.2	214.07	125.49	71.25	0	43.32	5	12.02	64456.45	1
p1306	D5	80.28	1.78	8.02	2.37	13.44	100.93	12.51	22.83	0	17.47	10.2	15.77	7.6	29.19	7.13	13.79	1.13	0	0	0	0	0	308.42	228.51	332.95	0	75.3	3.3	52.86	31796.14	5.87
p1305	Admission	94.47	6.78	23.87	6.41	0	405.67	0	0	1.79	171.46	7.12	37.58	10.72	530.47	0	0	3.41	330	46.55	303	6190	28.15	358.34	910.75	519.09	0	71.16	22	0	17824.9	11.17
p1305	Admission	97.66	1.78	1.59	0.93	0	22.84	0	0	0	65.87	7.85	24.67	0	16.64	0	0	1.4	0	2.98	0	0	0	311.87	535.9	1215.28	0	65.1	20	114.28	287116.2	15.27
p1305	Admission	54.9	3.89	9.82	4.52	0	3.68	0	0	1.43	49.76	5.99	19.94	4.51	19.35	0	0	3.15	210	20.97	68.28	2290	0	258.41	295.01	568.44	0	23.28	21	59.95	342670.4	6.22

p1305	D1	74.04	7.31	29.45	9.38	0	21.06	0.51	3.43	2.2	38.97	10.84	17.17	3.43	19.65	0	6.68	5.56	180	23.72	0	1910	0	311.87	331.35	688.65	0	37.57	34	166.15	257040.4	10.92
p1305	D1	70.62	7.31	30.81	10.11	0	24.63	0	1.36	1.69	35.5	11.61	12.5	24.57	13.3	0	5.37	5.14	850	85.8	616	8600	92.56	308.42	275.82	721.81	0	32.02	28	101.71	192535	7.42
p1305	D2	71.52	7.66	23.87	6.04	0	12.27	0.92	0.61	1.59	119.53	8.54	27.04	3.29	79.14	0	8.58	4.54	360	32.03	404	3420	17.78	425.7	557.91	762.38	3.42	51.95	158	150.46	244852.4	14.16
p1305	D3	91.76	30.01	78.21	29.45	12.6	116.36	7.27	12.3	7.16	21.31	25.94	14.14	17.48	25.33	0	13.35	16.03	810	65.08	495	4620	53.41	292.09	325.75	407.5	3.42	56.73	9.1	110.29	25952.7	18.2
p1305	D5	76.51	30.51	100.35	36.44	16.91	159.52	9.36	22.83	10.05	23.42	33.73	15.32	7.97	32.44	4.02	15.93	19.07	280	45.17	98.2	3450	0	278.53	228.75	415.48	7.58	55.87	7.3	108.57	23318.7	20.85
p1305	D6	95.41	23.54	77.66	24.28	10.39	163.49	5.6	12.66	7.93	19.3	21.95	14.05	4.1	24.73	0	15.93	13.79	510	41.03	354	4010	42.65	292.63	268.42	169.17	1.25	59.07	7.3	160.55	29662.3	17.02
p1304	Admission	67.03	4.44	14.04	5.28	0	7.08	0	0.9	0.3	8.63	0	10.11	19.81	4.59	0	0	1.84	450	66.44	98.2	4310	5.87	1062.93	145.81	976.96	0	8.98	14	0	41916.8	0
p1304	Admission	68.74	0.94	9.82	3.74	0	7.08	0	0	0.19	9.84	0.77	12.87	17.35	4.91	0	0	0.4	350	65.76	166	5470	14.49	729.29	137.18	742.86	0	9.38	14	0	72482.7	0
p1304	D1	78.73	1.37	7.07	3.55	0	3.68	0	0	0.07	39.93	0	14.82	14.37	3.46	0	0	0.49	270	51.37	0	4230	0	751.91	195.16	523.73	0	7.73	7	0	95801.2	0
p1303	Admission	147.59	4.63	4.02	3.35	0.64	28.21	0.92	2.14	1.9	24.2	0	28.96	74.8	26.81	0	1.27	1.75	5220	227	3014	29100	446	303.09	327.99	957.67	8.84	71.16	7.3	0	38855.6	4.64
p1303	Admission	85.08	7.31	19.48	7.53	3.54	32.71	0.92	1.82	2.81	51.85	4.98	20.96	45.99	16.03	0	1.27	2.63	1110	127	1038	15100	120	208.59	215.19	431.37	2.11	58.22	5.2	0	55854.3	3.55
p1303	Admission	93.42	11.12	32.82	11.02	4.76	43.63	3.81	4.09	4.21	60.88	8.2	42.98	59.59	21.45	1.99	3.99	6.42	1110	132	943	12540	72.28	203.66	232.79	341.17	0	53.26	7	0	58182.6	4.82
p1303	D2	103.76	1.37	9.82	0.04	0	21.06	1.65	3.43	0.74	71.47	1.5	17.21	30.6	17.7	0	6.68	0.86	1400	109	1190	9670	120	319.01	286.45	439.29	0	56.94	8.3	0	43531.5	4.82
p1303	D3	152.38	2.57	0	0	1.08	63.98	2.62	4.43	7.25	155.94	4.77	47.89	11.77	32.73	2.3	14.79	0.31	110	14.16	50.92	110	0	337.49	847.93	840.15	3.63	98.7	7	0	33998.3	16.94
p1303	D4	91.88	35.65	93.98	28.25	19.04	171.46	12.97	19.27	8.22	48.42	21.22	31.49	83.98	30.37	8.83	18.59	17.43	1760	264	1701	16480	164	346.2	339.29	444.03	9.45	74.91	11	39.14	33400.6	16.54
p1303	D5	88.52	26.2	77.66	28.77	16.12	139.77	12.29	17.97	10.82	54.21	19.48	26.03	75.83	28.59	12.09	17.76	14.29	1820	199	1323	16230	106	482.87	346.98	641.71	9.35	69.56	9.4	10.73	22205.6	14.48
p1303	D6	103.09	55.12	152.07	50.56	27.28	266.23	20.69	36.68	14.62	72.02	29.5	37.23	53.81	49.31	14.33	30.84	23.49	1280	133	963	9910	113	640.53	346.3	923.52	17.48	88.45	9.9	66.41	15170.7	27.7
p1302	Admission	56.34	4.44	0	4.52	2.03	57.47	3.81	11.4	0	127.45	0	18.78	6.8	866.38	2.45	4.69	3.15	0	0	0	200	0	265.07	611.91	1107.3	14.93	159.36	41	0	14756.95	13.19
p1302	Admission	47.98	5.89	18.73	12.29	2.56	175.45	1.65	19.08	2	190.34	2.92	49.67	19.7	32.14	0.16	3.99	4.19	330	21.9	224	3900	60.3	996.83	920.8	783.36	2.11	88.83	34	0	227089.6	12.79
p1302	Admission	38.15	3.33	14.84	7.53	0	63.98	0	6.64	1.95	218.23	3.41	42.85	22.8	128.21	0	4.17	2.11	470	34.3	459	5900	81.6	548.27	426.28	828.21	0	58.85	39	0	331966.3	6.65
p1302	D1	35.64	3.52	14.84	2.57	0	21.06	0.92	4.76	0.74	257.81	4.33	56.28	10.5	75.94	0	5.37	1.84	180	13.8	36.4	3300	44.6	312.13	570.5	458.21	0.86	48.38	26	35.52	322548.1	10.02
p1302	D1	38.35	0	0	0	0	10.53	0	0.32	0.3	206.05	0	48.61	0	14.82	0	4.69	1.04	0	0	0	100	0	373.21	790.57	1011.06	5.21	60.12	14	65.82	215431.7	20.15
p1302	D2	28.66	11.46	31.48	17.81	1.28	41.8	2.93	19.64	2.56	86.56	12.67	33.48	0	19.65	0	10.71	7.69	0	0	0	300	0	457.74	561.44	850.59	3.83	61.17	8.9	75.16	122816.5	22.17
p1302	D3	31.41	23.54	61.2	41.01	7.69	110.55	9.72	33.56	5.01	33.09	16.61	25.64	9.7	16.64	5.09	15.36	13.29	320	23.7	184	4000	60.3	326.12	454.63	439.29	1.94	67.34	6.5	112	72009.58	17.88
p1302	D4	36.7	18.04	53.54	33.89	3.22	44.55	4.65	52.13	2.81	45.3	14.83	23.53	2.2	17.85	0	9.65	19.24	0	10.2	0	1500	20.3	449.33	414.08	551.53	2.71	63.87	6.5	97.12	65635.1	21.24
p1302	D5	30.47	16.87	65.25	39.66	7.15	61.19	6.25	61.42	2.91	61.89	15.11	24.06	10.9	22.05	10.71	11	15.12	370	22.8	231	3700	50.8	369.75	317.26	337.89	5.21	68.75	7	52.86	55755.41	14.72
p1302	D6	33.01	19.04	53.54	34.24	6.74	59.32	3.52	39.81	4.41	63.35	17.28	20.96	2.4	19.05	1.99	9.5	14.62	110	4.9	0	1500	18	427.19	241.7	1169.43	4.34	65.3	5	46.33	37890.29	16.23
p1301	Admission	47	6.6	32.82	10.47	0	39.07	0	5.78	2.91	59.8	11.61	9.74	15.15	79.14	2.6	0	6.59	500	51.37	312	5420	29.28	164.49	214.53	608.23	0	48.38	15	0	22614.6	5.87
p1301	Admission	46.01	12.48	46.25	21.15	4.76	59.32	1.3	8.73	4.26	37.09	15.25	14.78	7.83	14.21	4.83	4.34	8.7	140	33.41	0	1590	0	148.81	117.77	373.75	0	43.09	7.5	0	57485.2	5.26
p1301	Admission	48.86	12.31	43.14	13.37	1.48	31.81	0.72	5.78	3.22	46.6	11.61	10.11	36.25	6.65	0	1.91	7.18	590	97.6	426	8160	45.1	84.78	174.85	195.18	0	37.08	6.2	0	72969.6	6.74
p1301	D1	38.55	13.16	49.92	13.37	2.56	51.91	1.11	9.26	4.51	146.86	13.55	15.95	29.84	29.19	4.02	6.36	8.61	620	76.65	470	6450	53.41	107.11	187.25	126.11	0	38.55	4.5	0	86450.9	4.82
p1301	D2	38.05	12.14	47.48	16.93	7.15	61.19	3.81	7.86	4.61	52.43	14.41	10.89	19.03	16.34	6.88	7.64	8.78	410	61.66	426	5470	5.38	172.36	107.24	287.93	0	42.38	7.3	0	49145.4	4.73
p1301	D3	44.55	14	66.4	22.2	7.42	75.24	4.1	5.78	5.99	30.47	16.48	11.08	21.87	18.15	3.47	6.84	9.71	540	79.02	433	5530	37.03	250.3	128.36	820.74	0	44.49	13	25.16	30301	9.6
p1301	D4	62.21	20.21	69.81	25.32	12.72	87.55	6.64	17.6	6.96	28.13	21.46	22.51	26.5	21.75	6.38	11	11.46	650	56.87	382	6250	24.82	240.12	192.22	1049.58	0	53.7	18	43.94	34613.3	14.16
p1300	Admission	160.43	4.26	52.34	13.37	17.14	165.48	13.53	37.07	5.3	23.03	0	15.91	16.83	48.44	25.98	17.62	4.02	510	76.65	306	5790	17.78	133.14	242.99	232.82	2.86	33.05	31	0	12748.6	3.73
p1300	Admission	87.67	2.95	69.81	20.8	15.89	183.47	16.08	36.09	5.1	120.45	4.33	38.52	14.37	43.9	24.13	20.38	5.22	230	34.8	0	3200	0	217.01	315.37	216.87	2.86	44.95	27	15.19	26635.2	5.87
p1297	Admission	47.89	9.05	39.98	8.64	5.63	63.98	6.38	9.26	2.31	28	9.22	7.77	75.34	21.45	4.29	8.89	6.42	1980	127	1696	6040	257	219.21	155.81	245.08	1.25	42.62	17	0	13322.39	5.87
p1297	Admission	48.07	2.95	12.4	4.71	0	22.84	0.92	4.43	0.3	13.06	3.65	6.82	17.93	415.72	0	0.25	2.72	1000	56.66	1103	1890	153	193.68	223.73	383.44	4.84	69.76	11	0	23285.54	4.19
p1297	Admission	30.71	0	0	0.04	0	15.76	1.3	3.11	0	76.62	0.77	14.42	8.11	9.31	0	2.51	0.95	420	21.78	254	730	61.04	197.03	232.43	204.28	3.56	46.56	8	0	127251	3.73
p1297	D1	67.53	3.89	13.22	4.13	0	29.11	4.1	3.43	0.19	45.83	3.41	17.75	70.66	14.82	0	7.64	2.63	1760	126	1902	5830	244	407.91	274.8	216.87	0	60.75	5.3	0	99693.54	4.28
p1297	D2	88.28	3.71	7.07	23.07	0	26.42	6.77	4.93	0.9	21.71	2.11	30.36	47.99	19.65	0	12.19	3.33	1540	110	1440	4610	226	1729.04	318.64	1318.83	0	57.37	8.6	47.52	40900.18	5.7
p1297	D5	70.55	45.94	94.51	34.24	16.46	151.6	11.48	14.84	6.33	12.34	23.27	17.12	32.85	26.52	6.38	19.01	22.27	770	52.14	695	2540	120	1088.37	164.65	423.44	5.68	88.27	11	103.43	14183.78	16.7
p1296	Admission	80.93	9.12	26.5	3.87	3.12	64.74	1.77	5.47	2.12	206.8	9.87	18.06	12.96	524.8	3.2	5.55	1.94	950	54.86	892	2210	132	374.91	2045.57	1683.47	9.21	80.29	33.3	12.31	4411.222	11.67
p1296	Admission	246.09	14.6	32.57	4.7	12.04	851.03	2.69	5.87	3.52	8863.3	3.91	782.05	46.03	155.63	3.44	5.74	4.19	1310	92.45	1236	3900	188	902.23	741.3769	1124.13	11.37	202.25	84.3	101.78	219644.8	29.4
p1296	Admission	332.61	14.16	39.35	6.77	7.3	589.75	3.06	4.46	3.66	8216.07	8.36	582.75	41.15	175.75	3.67	8.86	3.34	1180	93.13	1180	3900	161	1250.91	621.52	1015.52	10.83	88.27	177.6	207.05	474308.2	22.56
p1296	D1	50.37	6.09	45.87	2.22	0.95	138.02	2.14	0.71	1.89	834.86	1.89	118.83	8.91	310.65	1.31	7.67	1.94	650	36.75	748	970	79.99	725.64	1643.8	872.77	9.42	49.94	282.2	472.06	606516.5	15.96
p1296	D2	25.32	9.68	36.48	4.7	4.58	40.39	1.95	2.67	3.19	69.64	6.25	30	35.75	53.39	2.72	8.26	2.72	1480	99.59	1364	3620	194	1029.82	1307.3	639.49	15.33	48.8	124.3	134.55	495600.9	14.57
p1296	D3	34.84	14.9	45.87	6.77	6.06	64.74	3.06	7.49	3.37	28.7	6.52	27.59	34.03	24.98	3.91	5.17	3.42	1050	83.55	963	3440	141	1162.26	1288.51	931.17	10.13	52.19	50.881	92.08	337393.9	15.33
p1296	D4	64.61	14.16	38.4	5.94	6.06	55.61	2.69	4.66	3.16	42.95	6.79	33.95	49.93	46.6	3.32	5.83	3.65	1480	102	1361	5090	185	1157.06	1696.44	1397.77	12.95	76	98.5	82.4	216968.8	18.49
p1296	D5	51.46	16.68	30.57	4.9	6.31	70.83	2.69	7.09	4.55	40.09	11.78	33.31	36.48	78.95	3.56	5.93	5.66	1190	82.17	1116	3740	156	1477.23	1120.75	1600.22	10.13	65.51	35.8	166.18	103930.8	20.27
p1296	D6	45.72	14.9	40.29	4.7	4.34	55.61	2.14	5.07	4.4	35.42	10.83	18.77	19.35	52.8	4.52	5.36	4.04	680	54.15	654	1830	84.2	683.99	659.56	513.01	7.09	53.97	23.8	116.33	68750.59	15.4
p1296	D7	35.65	15.49	51.31	6.77	5.44	73.88	3.81	6.48	3.37	19.23	12.25	13.59	20.8	23.53	3.91	3.9	3.88	680	57	757	1970	82.4	522.43	517.08	599.07	6.2	52.64	11.3	74.53	54320.95	17.65
p1294	Admission	49.51	25.12	113.47	18.67	8	89.96	11.02	0	1.89	43.98	30.49	15.12	0	50.67	9.17	13.08	5.76	130	59.62	231	1600	25.12	168.77	141.52	278.03	10.31	53.05	23.4	25.09	8243.785	6.63
p1294	Admission	21.31	11.39	53.39	3.36	0	38.98	1.51	0	1.03	20.69	19.01	9.62	0	0	0	7.58	0	180	47.4	292	1280	27.14	156.11	58.09	555.6	5.74	29.47	11.3	0	44438.65	3.1
p1294	D1	49.67	24.46	109.79	14.49	10.22	108.33	14.56	4.76	2.13	323.58	32.75	20.72	0	0	17.97	17.66	4.99	150	39.86	124	1010	24.12	163.25	116.96	596.34	11.88	54.69	8.3	22.46	47516.42	7.29
p1294	D1	53.29	27.44	119.47	14.49	7.75	106.66	11.02	7.14	1.89	173.42	31.35	15.6	0	0	11.35	15.87	5.85	130	57.27	408	2020	19.15	181.9	147.4	309.14	10.31	53.87	6.3	20.28	21454.02	6.96
p1294	D2	43.39	26.61	111.02	16.59	8.12	129.9	11.66	5.96	2.48	30.6	32.19	11.02	4.77	0	11.35	15.08	4.04	560	92.92	746	2740	92.51	204.98	149.75	762.58	12.57	52.5	7.4	37.95	10864.38	8.74
p1294	D3	40.79	16.27	88.96	8.79	2.65	86.6	5.64	0	2.33	33.56	31.63	12.35	0	0	6.2	8.61	1.29	0	34.21	194	490	8.91	387.53	199.69	1458.69	11.32	57.63	9.5	30.83	2471.132	10.26
p1293	Admission	44.31	11.72	53.39	13.31	0	62.97	4.58	0	0.95	18.14	14.38	12.8	0	0	0	3.57	0	1210	0	747.22	1330	58.06	649.53	207.19	4995.96	7.99	36.69	62.9	6.98	54266.34	9.58
p1293	Admission	68.39	6.05	6.42	0	0	3.32	3.45	0	0.95	61.24	10.38	46.18	0	40.66	0	2.7	0	950	0	506.2	650	38.88	1662.42	795.98	7920.5	10.09	63.07	54.7	0.45	219808.9	13.18
p1293	Admission	104.98	16.27	79.03	5.62	1.27	54.45	7.38	0	1.4	108.58	17.53	54.49	0	33.56	0	12.27	0.42	970	5.68	777.06	770	39.61	1459.48	1106.34	8935.08	13.18	60.25	41	32.6	156893.7	18.26
p1293	D1	136.66	12.69	73.07	4.87	0	32.04	8.1	0	1.29	64.89	16.77	56.92	0	12.54	0	9.43	0.25	1080	7.94	653.99	1190	36.7	1394.28	657.95	4753.4	9.71	45.07	81.9	29.06	145278.3	12.49
p1293	D1	40.79	6.05	0	0	0	0	0	0	0.74	34.24	16	19.16	0	0	0	4	0	740	0	0	390	15.04	687.21	237.14	2689.55	7.66	28.74	38	1.23	87147.03	7.35
p1293	D2	62.7	16.92	79.03	12.37	0	62.97	9.02	0	1.49	15.72	21.12	19.04	0	0	0	9.23	0.77	870	0	961.67	900	39.97	445.94	278.72	2879.33	9.26	46.83	30.4	18.97	41453.9	11.8
p1293	D3	101.57	16.27	98.35	9.27	1.75	38.98	7.14	0	2.03	10.47	21.12	27.83	0	0	0	8.61	0.6	1350	7.94	801.25	1160	68.41	479	442.66	6626.59	9.1	44.48	39.9	18.54	28330.61	10.77
p1293	D4	61.07	23.48	104.78	16.35	5.95	114.98	9.02	2.93	1.89	11.92	27.84	39.6	0	0	9.17	12.07	3.01	1540	7.18	963.76	2020	95.31	447.34	375.7	6144.29	13.18	66.33	34.4	31.72	29522.16	12.14
p1292	Admission	40.28	24.63	143.19	13.78	6.6	139.81	14.15	5.96	2.28	44.87	34.94	20.11	0	27.9	7.73	15.08	5.33	0	0	0	0	0	293.46	203.22	738.13	10.31	66.58	42.9	32.16	79355.09	10.83
p1292	Admission	31.59	16.76	93.04	6.85	2.94	73.13	3.15	0	1.58	27.57	28.14	18.07	0	5.78	0	5.91	0.25	0	0	0	0	2.4	254.81	154.04	620.78	7.5	66.09	38.8	9.51	195259	8.35
p1292	Admission	37.86	28.76	118.28	16.82	7.24	166.12	14.15	8.9	2.1	37.47	35.47	19.77	0	1.35	9.17	19.62	5.07	0	0	0	0	0	257.82	177.76	410.46	9.94	83.96	36.9	35.27	200481	10.54
p1292	D1	25.58	25.45	109.79	5.37	14.93	159.56	10.14	17.19	1.89	32.86	32.19	16.26	0	0	10.28	30.66	2.67	0	0	0	0	0	293.34	188.84	638.71	7.66	87.29	33.3	46.92	179352.2	14.57
p1292	D2	49.35	6.86	23.69	0	0	28.54	0	26.35	0.95	52.61	8.87	141.82	0	0	0	6.12	0	0	0	0	0	0	749.23	409.09	4411.59	7.5	118.76	31.9	4.48	94653.63	19.39
p1292	D3	31.22	13.02	42.04	2.85	0	42.44	2.85	0	1.47	26.75	21.8	15	3.31	0	0	6.54	0	540	20.38	1305	1820	113	447.45	194.5	1476.83	6.11	58.42	23.8	6.14	63907.36	9.13
p1292	D4	36.64	20.85	108.55	12.61	5.16	136.51	10.36	4.15	2.38	24.83	29.91	17.38	3.89	0	3.06	11.47	2.84	410	24.26	927	1200	91.34	541.49	202.83	1027.04	9.41	70.01	21.4	17.24	63218.75	13.93
p1292	D5	43.98	11.56	51.59	0	0	35.52	0	0	1.56	29.09	23.14	12.67	0	0	0	4.22	0	300	5.75	738	360	53.46	978.2	358.47	1561.01	6.47	61.54	30.4	7.82	54898.09	12.43
p1292	D6	44.47	32.58	153.81	23.25	12.37	189.03	15.35	9.48	3.42	21.64	41.14	21.48	7.62	3.27	10.82	20.21	8.33	410	20.38	867	1010	67.28	548.03	263.52	558.85	13.64	81.71	25.9	39.29	45877.34	15.5
p129	Admission	27.0593	831.8114	397.8924	187.6169	318.6723	559.9269	122.5488	122.4748	82.2781	219.84	250.7821	16.1128	9.32	64.8275	62.9667	204.4188	261.4738	450	40.13	125	980	45.72	12.0628	301.0261	514.9802	230.2103	2467.07	21.569	1528.511	46796.68	82.5772
p129	D1	35.8345	946.889	470.5802	238.84	411.8238	2918.885	168.9915	180.3963	120.6798	329.0384	320.3248	20.1356	28.55	105.1638	80.5732	287.6942	339.6756	870	84.02	628	3280	111	17.4693	418.3047	668.2564	285.2126	3165.645	21	1993.378	20626.6	107.5665
p129	D3	55.397	1326.579	556.331	330.1106	465.8455	5251.237	242.6635	234.1369	153.9127	341.7558	406.558	27.7758	8.45	158.2301	88.5513	362.799	451.4843	300	32.29	0	760	34.31	37.2368	533.5545	830.1622	346.1582	3647.327	21	2323.128	7290.013	136.7327
p1288	Admission	39.85	14.32	45.98	0.72	0	171.04	1.51	0	0.9	372.04	9.89	152.34	0	368.1	0	3.36	0	480	0	0	360	21.72	227.07	2511.6	2338.58	8.07	105.05	59.2	2.03	7565.348	5.6
p1288	Admission	62.7	13.67	26.32	0	0	149.7	4.85	0	1.38	307.69	6.64	295.78	0	222.13	0	3.14	0	600	0	0	410	0	475.08	6737.82	2436.55	27.07	583.85	19.6	0	47180.72	2.28
p1288	D1	42.14	16.76	45.98	0	0	73.13	10.14	0	1.47	694	10.38	245.1	0	176.09	0	10.25	0	460	0	0	330	0	693.41	2409.17	2111.63	4.27	135.56	24.2	35.72	295675.6	5.54
p1288	D2	31.04	18.23	45.98	0	0	78.19	8.79	0	1.98	150.4	13.54	73.88	0	39.48	0	11.06	0	480	0	0	350	0	866.79	1507.82	1346.74	5.74	63.07	47	41.08	180530	8.07
p1288	D3	27.05	17.58	71.54	1.8	0	106.66	8.56	0	2.06	55.78	18.28	44.82	0	32.96	0	10.66	0	750	0	233.47	440	13.83	595.71	879.8	2098.23	6.99	54.41	41.7	91.28	24223.4	10.43
p1288	D4	29.35	18.39	95.71	5.62	0	133.2	11.23	0.35	3.23	41.12	22.47	44.55	0	25.81	0	11.47	0.94	720	0	0	460	13.42	714.94	565.1	2309.92	7.99	54.41	33.3	95.91	34527.98	12.83
p1288	D5	31.59	11.07	0	0	0	71.44	0.71	0	0.99	44.7	11.78	71.96	0	124.2	0	6.12	0	460	0	0	360	0	407.35	504.46	1946.11	4.27	65.34	15.6	173.25	0	11.57
p1288	D6	63.44	9.13	28.82	0	0	123.28	7.62	0	3.04	154.41	20.43	117.37	0	47.73	0	15.08	0	450	0	0	360	0	889.6	1321.77	2234.17	13.38	90.57	74.8	149.91	29887.8	28.91
p1288	D7	64.47	18.72	95.71	6.85	0	169.4	10.36	1.02	2.03	63.85	26.63	54.1	0	47.14	0	15.48	0.42	530	0	0	350	0	550.62	588.44	1038.49	10.46	54.14	22.9	99.17	2128.75	14.1
p1287	Admission	22.35	9.13	31.22	0	0	33.78	0	0	0.86	322.15	6.64	44.37	0	16.19	0	1.81	0	510	0	0	330	0	258.57	405.58	1584.14	1.07	29.84	26.5	0	176608.9	0
p1287	Admission	22.66	14.32	63.62	3.87	0	66.36	6.65	0	1.2	405.55	18.28	77.13	4.27	77.51	0	3.36	0	4240	35.37	1827.73	8020	270.07	256.19	600.75	1534.58	5.08	49.7	16.8	0	354758.6	2.79
p1287	Admission	16.38	10.1	44.04	0	0	28.54	0.71	0	0.99	164.7	11.32	35.28	0	4.53	0	4	0	630	0	0	380	0	224.83	295.22	716.93	2.68	46.83	15	0	318701.5	2.69
p1284	Admission	43.64	15.46	63.62	0.72	1.75	149.7	13.33	0	0.99	91.15	31.06	23.69	0	473.99	0	4	0	0	0.85	0	0	0	253.3	525.45	9291.88	6.56	92.3	24.8	2.84	9072.565	11.34
p1284	Admission	13.31	7.18	0	0	0	49.32	2.21	0	3.8	34.76	3.95	15.36	5.55	4.53	0	0	0	810	52.15	862	1710	132	140.08	223.88	596.34	0	18.17	12.5	0	80600.09	0
p1284	Admission	14.52	8.8	28.82	0	0	56.16	5.38	0	1.38	33.56	7.23	10.88	2.45	0	0	1.81	0	440	20.99	289	770	64.72	124.17	125.28	323.86	2.29	20.83	12.8	0	174375.4	0.26
p1284	D1	18.62	10.75	33.52	0	0	68.06	9.92	0	0.99	95.85	13.54	27.72	0	15.89	0	3.57	0	160	6.85	0	0	8.77	113.53	250.3	250.16	3.73	25.73	11.7	6.14	187941.6	1.28
p1284	D1	17.74	10.1	37.9	0	0	99.99	1.51	0	0.99	139.76	6.64	344.69	0	24.32	0	8	0	0	0.85	0	0	0	173.78	641.53	534.41	1.07	45.07	9.3	18.54	146343.9	4.15
p1284	D2	18.4	12.37	42.04	0	0	59.57	4.58	0	1.11	34.5	15.2	23.37	0	1.03	0	4	0	170	3.74	30.52	0	6.8	219.14	203.43	325.5	4.17	26.88	5.7	1.23	96372.22	1.38
p1284	D3	14.76	10.1	10.94	0	0	56.16	1.51	0	0.9	10.36	9.89	26.91	0	0	0	3.57	0	100	3.08	0	0	0	365.31	224.26	320.59	1.07	20.83	5.2	0	55702.95	1.78
p1284	D4	22.24	13.02	69.99	0.14	0	119.96	9.25	0	1.56	14.9	26.32	22.13	0	0	0	5.28	0	150	3.08	0	0	0	236.57	192.02	372.9	6.99	40.83	4.7	19.84	34691.19	5
p1284	D6	25.09	15.29	94.38	1.8	0	141.46	8.56	0	1.49	13.96	29.33	22.4	0	2.63	0	8.82	0	90	1.5	0	0	0	262.07	201.8	888.18	7.66	43.27	6	45.57	18425.58	3.41
p1283	Admission	30.48	20.52	73.07	30.03	0	84.92	2.85	0	1.33	112.25	6.64	31.95	0	38.89	0	0	4.22	0	0	0	0	0	302.18	790.24	782.15	4.88	44.18	36	0	6849.205	2.38
p1283	Admission	28.4	12.69	42.04	0	0	57.86	2.21	0	2.08	71.98	6.64	28.83	0	41.25	0	2.26	0	0	0	0	0	2.67	310.93	415.99	720.19	0.68	30.55	17.8	0	67957.68	2.53
p1283	Admission	21.2	17.9	42.04	0	0	61.27	4.58	0	1.93	54.16	14.38	25.93	0	17.4	0	4.86	0	80	1.43	17.05	0	10.09	315.4	338.1	335.31	2.29	31.61	16.2	0	144760.9	3.83
p1283	D1	19.5	11.39	0	0	0	32.04	0	0	1.51	55.16	5.37	21.27	0	25.81	0	4	0	0	0	0	0	0	396.81	341.28	482.24	0.14	33	14	0	97821.4	5.22
p1283	D1	40.28	20.85	84.78	0	0	103.33	12.5	7.73	3.8	115.63	15.2	33.75	0	97.18	0.71	14.28	0	0	0	0	0	0	560.87	692.98	544.19	5.74	46.83	14	7.82	57878.71	9.19
p1283	D2	20.78	14.64	35.75	0	0	76.51	4.03	0	2.33	59.2	6.64	247.99	0	7.02	0	7.79	0	0	0	0	0	0	412.16	723.7	483.87	3.28	33	30.2	0	54791.55	5.22
p1283	D3	35.93	15.95	37.9	0	0	62.97	5.9	0	2.63	119.76	6.02	41.27	0	21.02	0	8.61	0	0	0	0	0	0	729.65	498.94	570.26	3.17	28.74	20	0	112510.7	5.22
p1283	D5	61.07	15.78	28.82	0	0	62.97	3.74	0	2.65	15.52	6.64	28.73	0	10.71	0	6.12	0	0	0	0	0	0	592.93	357.78	625.67	3.95	25.34	4.9	4.48	46620.82	4.41
p1281	Admission	41.3	15.62	103.51	4.87	2.05	42.44	1.51	0	1.61	21.83	25.7	10.19	2.24	19.21	4.18	9.84	2.67	860	9.13	379.62	720	33.02	126.55	153.02	194.29	5.74	38.62	27.2	18.1	44069.45	4.15
p1281	Admission	32.51	13.99	106.04	6.11	2.5	42.44	4.58	0.35	1.79	30.86	26.93	11.42	0	1.35	4.18	9.02	2.15	730	6.61	0	760	8.02	139.44	180.65	148.12	6.9	47.41	18.7	10.36	45949.33	4.36
p1281	Admission	55.31	12.69	90.33	1.54	2.05	26.79	0	0	1.49	38.72	19.37	13.38	0	0	0	6.54	1.63	630	0	0	390	0	282.48	385.35	276.39	6.73	58.16	16.5	14.64	73262.5	7.18
p1281	D1	46.93	8.16	53.39	0	0	0	0	0	2.06	17.15	15.2	6.32	0	0	0	2.92	0.07	700	0	0	430	6.28	593.96	321.06	368	5.46	46.83	11.9	4.48	65913.88	4.9
p1281	D1	37.16	10.1	86.18	5.12	1.75	25.03	2.21	0	1.91	9.56	21.8	5.19	0	0	0.71	6.33	2.5	530	0	0	300	0	413.69	197.32	348.39	5.93	44.77	16.5	13.78	32277.49	4.9
p1281	D2	49.83	27.6	191.2	20.51	11.06	159.56	11.23	14.65	3.04	21.64	38.87	12.48	0	8.87	16.33	26.43	10.73	860	4.88	271.66	460	18.61	547.92	225.89	586.56	11.04	62.56	15.3	45.57	17299.5	10.77
p1281	D3	46.44	19.54	149.61	13.55	5.16	91.63	6.65	5.96	2.18	10.59	32.19	7.85	0	0	9.73	13.88	4.39	590	0	0	360	0	545.23	196.45	710.42	8.48	51.67	14	22.46	15511.17	8.68
p1281	D4	113.62	6.54	42.04	0	0	0	0	0	1.24	5.68	19.72	12.08	0	0	0	0	1.03	440	0	0	280	0	754.51	556.16	1701.67	9.86	69.77	12.8	24.22	9004.839	20.52
p1279	Admission	44.06	10.1	61.98	3.62	3.51	38.98	2.21	0	1.33	105.28	17.15	16.08	36	10.71	0	8.82	2.75	1710	92.89	555	4510	197	132.57	428.53	228.82	8.32	62.82	57.7	14.64	32251.98	5.65
p1279	Admission	45.79	10.75	90.33	0.72	3.51	42.44	9.47	0	1.16	246.57	17.53	36.99	12.21	199.89	0	12.48	3.36	1000	43.37	268	1860	120	225.49	781.76	281.3	8.48	49.42	22.9	53.69	96075.46	6.52
p1279	Admission	49.59	14.97	88.96	10.23	3.79	66.36	3.45	0	1.61	257.29	22.47	46.18	41.76	88.52	0	13.08	4.56	2180	112	796	4880	261	227.72	927.97	228.82	8.87	49.7	15	51.88	130279.9	7.51
p1279	D1	27.82	9.77	66.85	1.54	1.1	25.03	0	0	1.29	350.74	22.47	30.53	47.55	44.79	0	10.25	1.29	1960	111	615	5880	229	559.94	507.84	126.61	6.11	31.61	10.5	84.8	143112.8	3.31
p1279	D2	17.29	11.07	100.94	9.03	5.42	49.32	3.45	0	1.79	16.94	25.07	9.91	68.46	6.4	0.71	13.08	2.5	1610	135	667	5840	229	220.73	71.7	103.37	8.07	28.74	6.3	76.51	146867.9	3.1
p1279	D3	36.29	14.64	121.83	9.75	5.29	62.97	6.65	2.3	1.79	62.28	25.07	24.21	53.85	7.02	3.44	17.46	4.04	2450	141	908	6890	277	333.31	262.03	207.45	7.58	41.44	8.8	57.77	107880.1	4.73
p1279	D4	37.25	17.9	125.32	12.61	5.42	64.67	8.1	3.54	2.03	48.54	30.49	26.91	22.45	15.58	0	18.64	4.39	930	52.55	273	2440	119	461.46	334.39	392.5	11.74	46.83	11.1	72.84	70409.52	7.07
p1279	D5	32.97	3.46	0	0	0	44.16	0	5.36	2.13	150.7	12.68	187.07	0.77	43.32	0	12.48	2.15	0	2.27	0	0	0	721.03	1586.42	1384.58	9.86	48.56	11.1	275.99	36285.2	7.57
p1279	D7	27.05	17.25	141.02	13.31	6.73	83.24	6.65	7.14	3.69	46.79	32.75	22.83	19.58	17.7	4.18	17.46	5.93	620	51.72	180	1920	80	320.2	340.08	307.5	9.86	40.51	6.3	108.95	23726.17	7.57
p1278	Admission	37.71	18.29	50.48	6.9	2.86	58.7	0.91	0	1.89	293.03	6.74	48.64	0	20.28	1.87	3.34	3.09	650	7.13	357.38	560	76.98	480.09	548.56	1207.38	2.92	32.3	24.7	30.15	20537.23	8.38
p1278	Admission	38.05	6.89	30.91	1.37	0	4.56	0	0	1.08	648.55	4.43	62.34	0	12.9	0	0.73	0.63	640	26.12	904.04	1870	69.65	499.02	561.72	889.82	1.51	23.34	12.8	90.94	87828.28	9.75
p1278	Admission	38.31	3.71	10.2	0	0	0	0	0	0.83	394.83	3.85	43.61	0	21.13	0	0	0.22	870	9.21	448.9	0	32.09	724.84	640.28	1524.39	1.98	22.18	21.6	163.73	57375.06	12.31
p1278	D4	22.94	17.81	65.92	9.68	4.17	61.54	1.3	1.49	2.69	46.06	7.62	9.77	9.63	3.64	4.27	5.86	2.7	560	37.82	680.26	2470	91.28	328.92	110.35	586.65	2.43	29.96	38.8	41.63	26354.95	6.68
p1278	D5	26.26	0	0	0	0	29.63	0	0	0.7	25.78	2.56	33.54	0	0	0	0	0	50	0	0	0	0	675.53	276.49	1546.71	0	15.28	32	97.8	18777.51	9.54
p1278	D6	27.66	20.73	70.68	15.06	6.14	114.29	3.85	5.4	3.09	39.39	8.98	9.2	0	12.9	4.01	7.8	6.63	210	33.97	0	730	42.95	644.08	199.07	1228.42	4.49	33.32	26.1	130.03	33390.15	10.92
p1278	D7	27.46	0	0	0	0	11.16	0	0	0.59	9.3	3.77	10.46	0	0	0	0	0	40	0	0	0	0	730.81	208.32	2310.52	0	17.58	19.9	153.42	15700.28	12.1
p1277	Admission	93.19	114.26	334.57	87.5	25.8	276.71	15.39	44.2	14.76	47.41	36.08	19.47	0	53.94	8.73	26.43	37.98	0	0	0	0	0	238.81	195.47	297.83	11.2	119.8	46	186.48	8170.301	40.99
p1277	Admission	64.45	90.37	268.01	53.05	24.92	271.46	11.59	47.58	13.74	35.29	29.93	14	0	41.65	8.99	24.58	21.71	170	13.84	0	0	26.8	176.49	88.94	247.16	10.47	94.51	27.5	124.61	15755.04	29.41
p1277	Admission	56.83	16.36	89.81	18.01	0	0	0	0	2.37	21.77	10.09	7.51	0	0	0	0	4.72	300	24.03	103	450	43.07	145.07	105.6	329.51	0.83	56.22	24.7	8.43	17430.46	10.64
p1276	Admission	58.29	20.97	49.76	7.88	4.39	52.98	1.88	0	2.43	77.72	4.67	20.86	54.46	10.26	2.67	3.49	3.94	3220	204	2221	22260	339	189.6	266.51	1190.14	2.13	38.76	23.7	0	39673.9	5.15
p1276	Admission	47.99	31.1	90.46	20.29	10.51	154.87	6.81	15.63	4.68	103.69	10.36	25.44	45.99	57.31	8.21	10.51	7.92	1350	127	1038	10630	167	229.2	222.54	655.2	4.58	52.17	3.1	23.36	51526.6	8.99
p1276	Admission	56.03	33.6	82	16.7	8.32	111.55	3.94	3.45	3.36	53.55	6.59	18.24	93.85	13.57	3.74	9.01	7.11	6880	390	3144	30080	503	218.4	212.87	895.78	4.13	49.33	5.6	6.37	55022.2	10.64
p1276	D1	45.61	19.51	56.89	9.84	4.17	78.42	2.47	1.98	2.69	44.07	5.92	15.68	61.91	6.35	3.48	6.31	3.47	1780	198	1586	12780	140	227.47	150.82	698.36	2.63	36.32	8.8	0	34442.2	6.95
p1274	Admission	47.67	8.75	29.32	3.64	1.11	32.6	0.43	0	1.49	15.48	3.6	4.99	12.03	0	0	0	1.06	380	51.37	414	3810	30.8	350.33	215.92	1270.36	1.29	22.76	26.8	9.45	37125	4.29
p1274	Admission	59.3	6.89	20.21	1.69	0	11.16	0	0	1.2	7.19	3.26	2.48	2.75	0.16	0	0	0	110	22	110	1160	0	377.24	153.62	722.87	0.71	20.69	20.9	18.95	30934.2	2.61
p1274	D1	78.32	16.36	53.35	10.33	3.73	75.63	1.69	4.43	1.98	19.91	5.38	6.31	4.1	7	3.87	4.52	3.71	190	26.14	185	1860	15.78	392.16	264.09	1174.78	3.02	35.33	44.9	35.91	37327	8.58
p1273	Admission	61.22	0.68	0	0	0	0.97	0	0	0.75	65.4	1.21	14.04	1.92	171.11	0	0	0	220	25.1	452	1320	18.46	296.2	563.94	761.47	0	64.03	78.3	0	12602.4	3.9
p1273	Admission	32.43	8.75	19.35	3.64	1.54	41.42	1.69	0	1.26	30.79	2.56	10	7.83	13.9	1.05	2.46	0.63	460	51.72	492	4510	45.1	166.14	216.66	367.47	0	35.33	41.6	0	59317.6	1.98
p1273	Admission	21.42	1.95	6.09	0	1.54	14.34	1.69	0.5	0.86	55.73	1.71	8.33	7.83	5.07	1.05	2.46	0	560	53.43	764	5270	71.41	248.28	253.81	360.81	0	33.83	32	0	79350.9	3.77
p1273	D1	34.89	3.71	11.17	0	0.45	14.34	1.01	0	0.86	22.21	2.83	6.61	1.64	7.32	0.49	1.59	0	150	16.87	89.12	1510	5.87	281.18	311.35	235.47	0	46.2	18.8	0	81094.9	3.38
p1273	D1	53.8	1.95	0	0	0.67	208.19	0.72	4.18	1.03	42.62	2	160.93	0	6.35	0.49	4.82	0	0	1.54	0	0	0	469.58	902.99	809.89	1.4	57.06	35.6	8.43	49773.9	11.61
p1273	D2	41.86	13.24	43.16	5.92	2.64	72.82	1.3	0.5	2.17	11.05	4.91	6.39	6.77	3.8	6.12	7.5	1.8	740	62	891	6620	85.9	303.15	324.71	396.16	0.83	48.44	16.5	43.52	26286.5	7.36
p1273	D3	48.38	13	44.64	5.92	4.06	86.78	2.28	5.4	2.24	7.39	4.59	9.96	6.51	5.71	5.07	6.61	3.01	560	44.48	888	4400	81.05	311.92	306.94	523.45	1.18	62.01	24.3	61.38	20592.4	10.23
p1273	D4	26.26	7.35	26.9	2.34	0	26.63	0	0	1.43	0.2	2.56	4.3	16.45	0	0.77	0.73	0.49	760	67.81	746	6910	67.07	232.31	188.88	437.65	0.17	37.79	33.1	5.85	10253.3	3.38
p1273	D5	49.23	4.15	12.12	0	1.98	45.78	0.62	1.49	2.37	19.74	4.51	11.15	1.22	7.64	2.14	4.23	0.77	0	2.23	0	0	0	415.25	524.84	797.82	3.02	76.12	21.2	15.98	20684.4	13.71
p1273	D6	31.41	1.09	0	0	0	142.76	0	0	1.14	3.23	2.91	77.15	1.36	0	0	0.44	0	0	1.32	0	0	0	294.93	302.71	773.61	0.17	56.64	25.7	52.95	23283.7	9.13
p1272	Admission	36.92	2.82	9.21	0.23	0	29.63	0.53	0	0.97	81.71	2.56	22.7	6.71	10.26	0	2.17	0.42	370	32.04	55.22	400	45.15	188.49	540.49	686.06	2.23	20.69	25	0	36451.71	2.61
p1272	Admission	36.84	15.39	44.64	7.88	3.73	61.54	2.57	0.5	1.86	40.52	4.43	14.32	28.64	22.66	0	6.31	3.32	820	52.45	340	1620	79.15	135.57	291.51	356.36	2.82	29.16	26.8	0	83787.2	3.64
p1272	Admission	32.8	19.26	53.35	9.51	6.36	86.78	3.65	2.47	2.11	28.51	4.99	11.76	20.57	10.26	0.49	7.8	4.56	740	46.42	448	2200	83.02	134.1	250.94	242.49	3.4	31.27	16.5	0	97190.32	5.08

p1272	D1	28.05	2.82	12.12	0.39	0.01	4.56	0.53	0	1.08	16.12	2.19	5.17	8.89	1.63	0	2.75	0	240	26.95	0	590	29.43	176.49	234.08	136.27	1.4	16.93	11.1	0	66018.07	1.24
p1272	D3	37.1	13.95	41.67	6.58	4.17	71.42	3.06	0	1.86	40.2	4.1	7.28	17.61	8.78	0	5.56	2.86	560	46.42	250	2150	59.42	147.51	154.51	288.7	2.23	27.55	17.5	0	19405.23	3.06
p1271	Admission	36.4	14.91	60.39	10.49	3.95	72.82	1.69	1.49	2.17	41.82	6.67	11.88	64.82	16.24	0.49	5.71	8.73	1440	206	2284	6380	211	371.64	185.84	2135.61	1.93	55.37	23.7	0	45785.35	4.82
p1271	Admission	30.94	19.26	74.71	14.41	7.01	82.61	3.65	6.38	2.46	34.63	7.11	9.01	67.13	13.4	1.87	8.1	9.95	1400	232	2037	7160	233	318.91	152.72	1272.27	3.4	51.52	18.8	0	145684.8	5.28
p1271	Admission	38.66	17.32	74.04	14.08	4.83	72.82	3.06	3.45	2.27	39.22	6.59	11.45	0	15.9	1.18	7.8	11.35	400	13.4	1237	850	78.09	390.71	188.53	821.94	2.43	57.48	16.5	0	141852.6	7.49
p1271	D1	24	12.52	64.55	8.7	2.53	29.63	0.43	2.47	2.05	21.51	7.04	5.61	0	7	0	5.41	7.43	0	0	0	0	0	599.39	114.03	692.21	1.4	37.79	11.8	0	26048.46	5.35
p1271	D2	21.42	0	0	0	0	55.84	0	0	0.7	32.64	1.62	57.22	0	0	0	0.73	12.18	0	0	0	0	0	230.24	231.07	875.9	2.33	43.7	19.5	0	120969.1	3.45
p1271	D3	42.85	25.64	94.95	16.7	9.2	122.46	4.44	7.84	3.26	17.31	10.29	8.22	0.5	10.59	4.01	11.42	9.22	0	19.92	758	0	14.76	426.09	160.29	710.63	4.49	55.37	38.4	0	40779.71	7.9
p1271	D4	37.45	26.63	102.55	19.97	9.85	138.72	5.72	16.11	3.36	34.96	11.5	10.08	0	10.92	4.4	10.97	11.93	0	2.66	194	0	0	366.61	183.97	735.09	5.84	59.35	19.9	0	27670.38	9.75
p1271	D5	29.61	0	0	0	0	160.24	0	0	0.62	21.33	3.6	118.18	0	0	0	0.44	15.52	0	0	0	0	0	827.83	109.95	1587.55	1.51	35.08	24	0	21418.44	3.51
p1268	Admission	52.45	37.87	73.37	23.57	9.96	119.74	9.79	8.09	2.76	66.77	11.3	11.84	3.6	42.52	8.47	14.76	12.35	160	7.58	24.16	1750	10.07	252.3	334.79	863.95	9.38	63.23	59.8	63.24	12419.3	12.17
p1268	Admission	41.53	39.89	108.81	19.97	12.04	136.01	7.01	8.82	3.49	59.44	8.06	14.24	6.83	22.66	8.73	13.24	9.22	460	23.63	323	4890	38.6	208.3	285.61	1062.71	5.8	55.8	106.7	43.05	23981.9	11.82
p1268	Admission	55.36	25.64	75.38	11.8	6.79	84	3.26	4.91	2.43	73.24	7.84	20.38	0	13.9	5.99	10.36	7.84	80	3.07	0	680	7.14	249.96	445.47	964.94	6.39	63.23	91.2	74.37	31853.3	15.97
p1268	D3	24.83	24.16	65.92	13.92	7.23	95.08	3.75	5.16	2.82	40.2	7.19	13.69	3.6	11.58	6.12	10.51	5.75	140	11.53	0	1750	9.31	217.88	295.25	320.5	5.32	48.44	12.5	111.47	30174.5	10.37
p1266	Admission	114.98	3.71	10.2	2.34	0	0	0	0	1.26	0	2	4.7	0.25	4.27	0	0.17	1.95	480	25.14	347	1170	47.89	239.83	232.53	671.68	5.63	41.37	69.6	0	10751.09	3.51
p1266	Admission	127.04	5.97	8.2	1.37	0	274.08	0.34	0	0.97	120.58	5.14	25.27	0	241.1	0	3.49	1.65	230	10.85	150	240	30.36	139.11	539.95	671.68	2.38	29.96	42.1	0	114710.9	8.72
p1266	Admission	119.1	6.89	15.82	3.16	0.23	19.02	0	0	0.86	46.46	4.99	22.42	0	19.44	0	2.75	2.55	330	16.44	160	470	33.59	113.74	551.82	400.56	2.68	33.58	32.1	0	233997.4	9.4
p1266	D1	84.51	8.28	23.6	9.84	0	0	0.43	0	1.38	29.11	4.26	14.86	2.47	21.3	0	4.52	4.1	530	29.04	251	1100	53.74	182.9	429.78	230.77	3.4	29.96	22.5	0	166902.7	5.61
p1265	Admission	55.07	80.64	253.81	213.59	21.87	634.8	28.66	119.85	48.82	115.88	13.8	90.37	13.05	203.17	167.53	91.94	33.93	53330	25.78	7348.13	62770	1127.68	189.6	117.3	968.87	19.88	161.77	20.9	48.25	12452.29	24.15
p1265	Admission	62	78.81	274.8	178.55	24.05	566.62	33.92	119.37	41.76	126.03	14.05	82.33	17.18	191.96	152.19	86.35	30.96	23280	51.6	6361.76	66800	1072.54	163.61	152.93	1000.27	20.02	163.69	11.8	66.96	17767.77	20.54
p1265	D1	56.47	35.61	189.19	113.78	13.9	400.85	19.2	88.57	27.92	200.18	9.6	93.09	5.7	184.13	110.08	49.17	18.73	4540	16.79	2311.3	9390	281.67	141.79	239.98	673.74	12.7	113.41	8.6	8.43	29064.26	12.73
p1265	D1	52.75	64.74	259.09	132.18	21.65	516.94	27.45	103.01	32.21	128.25	13.6	69.42	12.7	148.15	116.77	67.45	28.96	5530	36.44	2170.51	15540	265.59	298.89	108.81	819.94	17.09	125.12	3	64.17	89683.18	18.82
p1265	D2	54.62	78.81	260.67	192.53	25.8	645.31	33.31	108.3	47.02	114.68	13.28	69.76	8.87	163.69	149.65	97.37	35.69	4530	23.68	3158.11	24040	349.59	294.3	88.1	601.27	18.98	172.44	6.8	73.45	26986.62	26.19
p1264	Admission	46.88	4.15	11.17	0.07	0	727.05	0	0	1.49	319.47	3.52	77.72	0	456.2	0	0.59	0.36	840	0	291.78	430	17.03	1560.64	1928.33	5467.72	7.83	65.63	27.8	0	46337.63	29.7
p1264	Admission	29.51	3.26	17.6	0	0	154.87	0	0	0.97	74.74	4.1	33.42	0	63.87	0	0	0.36	1590	0	767.21	740	43.21	840.97	759.57	4015.05	4.22	34.33	34.2	0	249648.6	19.1
p1264	Admission	22.51	7.82	26.08	2.34	0	57.27	0	0	1.2	32.97	4.18	17.65	0	12.24	0	0.44	1.21	5050	19.65	1708.98	3310	189.47	699.1	441.59	3103.23	6.01	35.33	35.5	0	287737.7	16.11
p1264	Admission	32.06	4.38	26.08	2.99	0	12.76	0	0	1.03	25.69	3.52	12.92	0	12.74	0	0.73	0.15	2070	8.27	1263.42	2960	145.73	744.25	354.68	1897.49	2.63	26.73	17.1	0	54522.14	8.72
p1264	D1	31.97	0.68	12.12	0	0	0.97	0	0	0.86	20.09	3.26	6.69	0	3.8	0	0.3	0	1410	12.73	834.2	2190	88.67	675.02	375.96	1859.31	1.77	21.29	12.5	0	121245.4	6.82
p1264	D2	24.62	15.15	46.11	8.21	3.08	58.7	0.72	0.5	2.21	20.18	7.04	6.98	15.3	3.64	1.05	2.17	2.7	1520	55.85	1051.04	2300	119.5	510.18	198.57	1300.76	3.3	29.16	31.5	0	46140.05	7.83
p1264	D3	38.83	1.95	6.09	0	0	41.42	0	0	0.86	10.86	2.91	13.93	0	0	0	0	0.09	510	0	0	390	0	719.12	523.75	2752.55	3.49	35.83	15.5	0	36508.13	17.82
p1264	D4	25.04	1.95	1.26	0	0	75.63	0	0	1.4	18.3	2.91	32.84	0	0.44	0	0.59	0	490	0	0	350	0	669.11	517.25	2365.56	3.99	23.92	16.8	0	25319.59	16.11
p1264	D5	29.13	13.47	46.85	10.16	2.64	95.08	1.11	0.99	2.95	18.66	7.19	7.51	6.03	5.71	0.91	4.52	3.32	2830	31.38	1550.36	4790	186.08	603.45	265.85	1272.27	3.58	26.73	31.1	13.99	19967.83	8.86
p1264	D6	36.4	13.95	54.77	8.53	3.29	100.59	1.3	1.98	4.47	17.94	6.52	7.54	4.29	5.71	1.32	5.41	3.32	2840	22.53	1690.45	5400	207.9	752.86	324.76	1382.06	4.4	29.69	19	0	19595.43	9.27
p1263	Admission	51.54	13	35.6	7.07	1.54	72.82	1.2	0	1.61	89.33	4.02	13.38	21.49	27.1	2.94	3.93	2.25	1100	78.98	372	2220	142	258.3	485.58	592.92	3.95	53.24	36.9	0	31830.54	3.51
p1263	Admission	48.14	20.24	56.89	12.61	5.05	89.55	2.47	0.99	1.98	27.92	5.22	10.65	20.53	8.62	5.07	5.71	5.12	670	72.41	190	2040	97.93	255.97	232.04	444.15	4.18	53.89	28.8	0	96463.19	5.48
p1263	Admission	49.39	16.36	47.58	7.55	2.86	55.84	1.88	0	1.8	34.13	4.1	10.23	32.76	5.55	3.21	5.71	3.47	840	99.41	268	3250	114	240	272.71	428.96	3.49	54.53	33.8	0	110037.9	4.75
p1263	Admission	81.25	6.43	19.35	2.02	0	7.91	0	0	1.32	56.51	3.52	16.23	9.62	22.83	0.49	3.63	1.65	670	42.53	103	1250	69.67	426.09	650.19	565.68	3.95	84.66	34.5	15.49	119343.4	6.68
p1263	D1	71.79	13.47	42.42	12.45	2.42	64.37	0.91	0	2.24	45.82	5.06	12.76	38.75	10.59	8.47	8.1	3.32	1380	123	537	4300	176	368.09	559.63	448.48	4.58	67.62	20.6	11.48	79285.13	5.81
p1263	D2	52.6	6.43	20.21	3.16	0	11.16	0	0	2.11	91.23	3.18	17.93	35.25	10.26	0.21	6.01	0.77	1270	112	551	4230	177	551.9	609.95	340.73	3.49	51.95	17.1	3.21	57475.41	3.64
p1263	D3	45.05	23.67	70	15.88	4.83	108.82	3.65	2.22	4.36	242.13	6.89	33.83	43.27	19.44	4.27	13.24	5.67	1550	127	626	5360	232	825.7	511.02	1334.87	5.15	65.63	7.7	40.68	70354.73	9.95
p1263	D4	49.54	1.09	0	0	0	104.71	0	0	3.43	67.68	2.38	268.29	1.36	0.16	0	5.41	0.36	150	15.76	0	70	5.77	636.82	960.68	1464.66	4.67	49.99	47	87.27	75580.64	10.51
p1263	D5	29.8	21.21	60.39	11.47	3.29	70.01	1.49	1.98	3.84	26.04	6.22	9.69	27.32	4.43	6.77	6.75	5.12	760	83.9	265	2470	115	457.91	228.16	2338.94	4.84	45.07	44.5	30.15	49674.48	8.45
p1263	D6	31.5	21.46	56.19	14.08	4.61	84	3.06	0.99	3.77	21.6	7.33	9.09	37.25	2.55	5.59	8.55	4.25	1050	101	386	3360	139	287.92	257.67	320.5	4.76	44.38	11.5	38.77	63409.34	6.95
p1261	Admission	37.79	16.6	64.55	11.8	5.26	65.78	1.69	4.43	2.79	30.96	7.11	5.14	69.59	52	4.27	5.56	4.64	2570	151	1924	7490	293	183.27	131.13	454.96	3.4	30.22	30.1	6.37	37065.36	5.75
p1261	Admission	28.84	18.53	55.48	10.82	3.95	55.84	1.98	3.45	3.29	15.39	6.59	4.41	54.04	4.12	3.21	3.93	3.32	2170	144	1455	5810	234	123.22	84.11	254.14	3.35	32.56	11.8	0	72568.51	4.03
p1261	Admission	33.53	3.71	15.82	0	0	0	0	0	0.92	10.28	2.56	3.76	0	6.35	0	0	0.22	390	24.85	279	610	49.71	209.91	134.46	540.39	1.4	20.08	19	0	84022.25	9.47
p1261	D1	78.06	16.11	65.92	10.65	3.84	52.98	0.53	4.43	3.02	25.35	8.06	8.78	15.06	6.67	2.14	4.23	3.63	840	53.79	766	1930	109	343.54	279.97	356.36	3.58	38.27	10.3	7.41	65890.04	10.23
p1260	Admission	56.03	26.63	92.39	14.74	9.85	986.58	4.24	4.43	2.46	1015.64	10.77	551.93	3.77	292.2	2.94	21.8	9.06	350	18.48	176	470	43.95	811.16	2978.09	1528.11	20.91	107.41	42.8	1793.79	422930.8	87.5
p1260	Admission	139.89	17.32	43.16	4.95	6.79	6763.24	1.69	0	1.08	1924.38	6.52	146.92	0	691.46	0.35	18.43	7.27	0	7.19	0	70	13.73	1000.44	3900.54	986.55	39.37	196.56	44.9	1262.07	393637.6	107.54
p1260	Admission	274.01	39.64	91.1	17.68	10.29	4167.48	33.51	1.73	2.99	273.63	9.12	873.16	10.16	302.47	3.21	18.28	27.48	730	31.62	406	930	75.58	1278.59	4731.51	1652.27	33.54	205.69	59.8	1480.84	437440.4	124.21
p1260	D1	100.4	40.65	132	26.02	9.85	383.9	5.03	6.62	3.56	1343.31	12.37	482.54	2.91	106.73	6.64	24.58	20.17	390	17.8	160	470	43.95	1305.04	3422.67	3375.02	16.56	85.39	99.9	1103.55	455350	72.6
p1260	D4	53.2	2.82	1.26	0	0	47.23	0	0	0.59	8.9	2.19	54.42	0	16.58	0	8.71	0.36	0	0	0	0	0	741.46	604.31	536.16	3.54	18.53	74	443.01	181450	17.82
p1260	D5	136.37	13	43.9	7.72	1.11	133.31	0.15	0	2.14	31.13	6.59	83.63	1.59	20.96	0	10.97	2.86	530	22.83	297	810	60.7	664.22	909.05	500.04	4.4	27.55	45.6	296.46	163685.2	22.12
p1260	D6	88.95	27.62	101.29	18.01	5.48	168.27	2.86	3.45	3.63	31.63	13.21	59.39	5.92	23.68	0.35	11.72	7.92	910	29.69	361	1400	72.93	651.58	533.57	563.58	4.49	37.3	82.2	253.35	167434.2	24.3
p1259	Admission	29.61	3.35	79.77	25.94	0	11.66	1.14	6.99	1.41	32.38	5.56	8.42	0	12.19	1.17	5.31	5.43	0	5.68	0	0	9.39	316.53	159.42	694	0	24.71	34.9	0	16275.4	0.82
p1259	Admission	16.43	3.22	19.85	10	0	11.66	0.2	8.1	1.39	10.22	1.89	3.89	0.25	1.79	0	4.66	2.84	230	17.8	35.78	260	28.73	120.24	98.98	385.72	0	15.75	21.6	0	69617.58	0
p1259	Admission	21.94	7.58	33.43	8.86	1.37	25.45	1.14	10.61	1.81	19.45	4.93	6.49	7.62	3.74	0	5.98	4.09	610	27.75	150	920	54.91	147.15	161.76	330.55	0	24.95	20.5	0	66145.57	1.83
p1259	D1	28.88	12.33	48.66	14.27	3.44	50.11	2.94	13.42	2.58	9.66	8.43	5.38	17.23	7.67	4.23	6.8	6.4	830	49.8	464	1740	86.12	162.69	112.26	133.11	0	30.21	20.5	0	27941.39	1.83
p1259	D2	29.79	1.77	33.43	13.13	0	15.6	0	4.24	1.28	12.39	2.1	5.22	0	3.18	2.95	5.18	3.02	0	4.9	0	0	0	245.74	134.18	229.23	0	20	18.4	0	21465.14	0.41
p1258	Admission	38.01	5.71	21.43	3.6	0	0	0	0	1.64	20.49	2.1	10.04	0	2.48	0	4.02	2.84	190	0	0	0	0	218.13	237.72	906.7	0	23.46	15.5	0	10735.16	1.61
p1258	Admission	32.26	7	21.43	4.31	0	0	0	0	1.93	15.42	4.45	12.51	0	0	0	4.4	3.09	4700	12.07	1555.84	3260	247.24	249.45	277.8	259.11	0	24.71	11.9	0	17323.8	1.93
p1258	Admission	28.14	4.03	14.93	2.89	0	0	0	0	1.96	10.22	2.5	7.92	5.87	0	0	3.41	1.74	1140	29.89	847.26	2940	103.76	329.93	269.07	964.93	0	23.72	8.3	0	17298.14	1.77
p1258	D1	43.05	3.62	13.22	2.03	0	0	0	0	1.87	4.43	2.5	4.06	0	0	0	3.65	1.86	1010	0	214.97	0	41.68	276.26	178.16	497.65	0	22.44	5.8	0	12051.92	0.07
p1258	D2	22.46	1.9	5.82	0.04	0	1.79	0	0	2.83	358.7	0	39.02	0	16.46	0	4.66	1.03	280	0	0	0	6.87	395.85	533.52	1004.65	0	31.07	18	0	54287.91	5.11
p1258	D3	19.9	1.64	0	0	0	0	0	0	1.19	125.72	0	15.31	7.23	0.41	0	4.53	0.8	590	23.26	0	0	33.43	284.18	345.28	979.9	0	15.75	25.6	0	84996.4	5.17
p1258	D5	35.9	1.77	0	0	0	13.63	0	0	1.81	24.54	1.46	50.59	0	0	0	5.18	1.98	50	0	0	0	0	364.17	484.04	1002.18	0	28	24.9	0	21915.9	10.52
p1258	D6	24.19	10.23	37.7	6.02	0.22	15.6	0.29	2.62	2.4	10.31	6.16	9.75	0	0.82	0	5.44	5.24	1520	9.81	770.34	470	94.24	280.23	219.59	431.15	0	27.54	13	0	16801.44	4.03
p1256	Admission	55.34	26.84	107.46	31.93	15.33	146.46	8.3	43.01	7.41	27.4	24.05	16.21	21.35	24.34	15.43	13.88	13.28	870	57.14	377	2540	74.44	591.27	136.72	1938.51	14.76	49.45	55.5	17.2	41532.13	10.4
p1256	Admission	45.87	7.87	39.1	9.14	3.04	45.17	2.59	14.55	3.26	12.03	8.71	6.02	28.68	9.93	5.59	6.66	3.34	810	61.39	347	2550	70.65	475.61	87.07	1217.87	0	27.08	58.3	0	61118.1	0.61
p1256	Admission	43.35	8.45	40.49	8.43	4.62	60.98	4.54	15.68	3.14	16.34	6.75	8.12	29.08	12.76	5.88	7.22	4.09	740	54.7	283	2130	59.55	554.1	111.77	2536.19	3.17	28.9	75.3	0	68896.9	2.81
p1256	D1	52.78	21.68	94.7	21.1	10.92	130.49	7.17	34.3	6.3	24.15	19.84	12.51	83.46	23.62	10.45	10.06	8.51	2000	142	1056	6720	194	498.14	100.29	1384.95	11.01	42.09	21.6	2.54	60782.88	6.31
p1256	D1	41.07	9.63	45.97	9.43	5.96	58.01	4.89	17.09	3.45	15.5	11.54	8.27	43.92	12.48	7.46	7.5	4.92	1410	90.62	650	3660	120	612.24	102.55	1896.37	5.3	29.34	22.3	0	45520.75	4.26
p1255	Admission	45.35	14.16	51.33	12.13	4.81	55.05	3.3	13.14	2.89	18.15	11.29	8.17	15.13	13.05	4.54	7.22	5.88	320	33.88	297	3780	29.6	338.6	125.76	705.34	0	32.95	24.9	0	16230.8	3.03
p1255	Admission	53.73	16.02	55.27	13.7	6.43	68.89	5.25	18.8	3.58	19.69	8.57	14.45	21.41	13.05	6.61	7.93	8.37	570	52.31	574	6030	51.74	328.46	208.21	790.97	4.12	41.56	20.2	0	24801.9	4.71
p1255	Admission	64.28	8.45	34.86	7.44	2.22	29.39	5.95	3.16	2.31	18.72	5.86	22.55	14.07	12.19	1.17	6.25	5.95	280	33.88	146	3130	22.63	431.68	237.4	612.47	0	34.56	28.1	0	24328.1	2.04
p1255	D1	53.19	13.09	51.33	14.27	5.96	57.02	3.83	14.83	3.05	12.21	9.11	6.44	11.68	12.76	8.43	7.93	5.88	200	26.71	76.93	2370	17.6	339.45	135.66	1699.91	0	32.33	88.6	0	27429.8	2.7
p1255	D1	52.64	13.86	53.96	10.57	5.58	59	3.83	18.23	3.23	17.33	9.63	8.52	17.12	10.49	4.84	7.01	5.24	390	40.92	386	4310	35.82	342.24	121.54	1627.02	0	34.76	67	0	20305.4	2.48
p1254	Admission	47.82	3.49	18.24	4.02	0	25.45	1.68	7.55	1.87	26.28	3.95	6.91	3.6	8.23	0	5.71	2.84	140	8.24	0	1330	6.8	276.39	89.57	2308.73	0	23.21	50.4	0	7476.4	1.83
p1254	Admission	36.31	2.16	11.47	3.31	0	11.66	0.39	3.7	1.53	21.36	1.68	5.65	4.9	1.65	0	4.15	1.98	160	14.53	0	1950	12.87	179.44	75.49	722.22	0	22.18	24.9	0	27932.3	1.4
p1253	Admission	46.16	15.86	54.62	23.95	3.04	50.11	3.83	1.55	3.08	12.03	10.15	17.77	3.77	15.61	0	6.66	9.3	380	21.17	160	500	44.74	359.5	304.14	727.81	1.71	58.26	24.1	19.82	17359.7	7.24
p1253	Admission	31.83	12.64	41.88	13.41	2.33	29.39	0.48	1.02	2.28	11.86	7.75	11	0	3.46	0	5.84	6.53	0	5.68	0	100	9.83	325.64	184.16	865.32	3.82	43.16	14.8	0.68	30546.97	4.83
p1253	Admission	20.12	12.94	53.31	11.7	3.04	33.33	0.39	5.89	3.23	5.3	10.54	4.17	2.91	4.02	0.97	5.98	6.27	270	13.67	29.35	650	26.28	217.72	85.39	699.68	0	25.68	10.1	4.27	27728.4	3.03
p1253	D1	26.25	6.42	27.56	10.57	0	0	0	0	1.93	2.93	5.56	2.54	2.91	0.69	0	4.27	4.6	260	16.44	44.68	450	29.55	290.9	114.1	487.91	0	23.72	10.1	0	22276.94	1.29
p1253	D1	18.55	3.35	10.58	18.25	0	0	0	0	1.58	10.22	7.32	1.17	0	0	0	3.53	6.53	0	0	0	50	0	401.37	115.26	656.63	0	18.86	8	0	21515.36	1.5
p1251	Admission	54.87	1.52	2.56	1.04	0	0	0	0	1.3	21.67	0.71	4.78	3.6	0	0	5.05	1.15	170	15.56	56.67	1580	11.64	449.24	181.94	1647.39	0	14.38	40.7	0	50911.5	0.46
p1251	D1	47.82	3.9	13.22	2.89	0	0	0	0	1.56	54.43	1.68	7.37	8.79	2.07	0	5.44	1.38	290	38.56	242	4360	25.24	306.84	205.89	774.69	0	22.7	27	0	85948.5	0.36
p1251	D2	42.6	0.78	0	0	0	0	0	0	1.33	44.05	0.71	4.51	0	0	0	4.92	0.57	100	7.8	0	1210	8.2	403.95	188.02	788.26	0	18.56	15.5	0	48259.2	1.61
p1251	D3	40.45	2.29	0	0.04	0	0	0	0	2.71	59.27	1.89	9.95	6.69	0	0	5.98	0.91	360	32.14	317	4110	35.32	530.37	194.32	906.7	0	26.39	34.6	0	43127	3.25
p1251	D4	30.42	5.29	24.53	5.16	0	13.63	0.39	2.62	4.03	18.32	4.61	5.17	16.79	0.96	0	5.84	2.35	630	62.06	565	5840	46.03	241.61	78.37	520.11	0	27.54	24.1	0	36210.6	1.29
p1251	D5	67.41	1.02	0	0	0	23.48	0	0	3.52	18.8	0	55.7	0	0	0	5.18	0.8	0	1.11	0	0	0	520.56	316.37	1487.84	0	52.2	24.1	56.42	33729.1	10.87
p1250	Admission	46.45	3.62	31.98	4.88	0	0	0	4.24	2.02	14.31	1.46	29.49	9.91	4.86	5.44	3.65	1.8	430	36.79	416	3870	39.11	348.54	246.44	1278.99	0	14.02	25.2	0	17850.1	0
p1250	Admission	49.66	5.85	36.29	7.01	0.08	13.63	0.39	8.66	2.16	31.44	2.3	27.09	10.75	8.66	10.84	4.66	2.1	450	44.49	337	3800	26.92	316.4	236.36	774.69	0	19.44	37.1	0	23836.2	1.34
p1250	Admission	62.72	3.49	29.05	5.73	0	0.8	0.2	5.89	2.46	21.04	2.5	21.6	0	6.26	6.75	4.66	2.59	50	5.27	0	360	0	420.88	271.18	1002.18	0	22.7	21.3	0	22989.3	2.37
p1250	D1	45.28	3.62	31.98	5.3	0	0	0	6.99	2.83	15.16	1.22	9.17	6.13	7.1	9.11	4.27	2.1	280	26.43	222	2460	23.1	424.37	178.25	713.8	0	17.96	20.2	0	34982.5	1.08
p1248	Admission	37.44	2.95	13.22	6.44	3.14	44.18	4.89	21.08	1.44	69.38	2.5	17.68	1.15	57.06	5.74	8.66	3.21	190	6.44	87.88	240	27.1	641.18	494.21	1785.41	0	55.81	39.2	4.27	27288.26	12.24
p1248	Admission	30.42	7.87	45.97	15.55	10.29	108.58	8.65	37.2	3.39	74.59	12.04	17.24	10.16	40.68	10.18	11.82	7.32	750	22.5	307	1230	67.98	632.39	337.93	1529.9	7.52	49.77	30.6	20.56	48245.12	14.04
p1248	Admission	22.98	5.29	41.88	16.97	5.96	53.07	4.72	21.08	3.01	53.21	13.95	12.04	8.47	17.89	6.89	8.15	6.93	470	22.5	198	840	44.74	444.17	200.19	870.53	0	35.16	20.2	8.35	42541.84	6.2
p1248	D1	35.48	17.26	85.2	20.24	10.38	146.46	6.47	36.04	4.95	39.49	17.14	13.16	13.92	25.77	12.02	11.67	9.84	710	34.17	262	1790	61.86	381.32	211.88	444.76	9.5	46.78	23.8	45.83	40671.49	10.99
p1248	D1	29.24	11.28	94.7	44.21	7.46	64.93	7.35	24.51	2.95	35.44	15.57	13.16	21.35	17.89	6.18	8.95	17.34	1140	46.09	501	2820	96.55	440.47	212.17	708.16	4.26	44.04	17.7	29	22068.36	10.05
p1248	D2	43.2	17.89	76.72	22.24	8.56	120.52	8.39	34.88	5.15	51.39	16.25	12	33.12	34.44	10.84	14.2	11.05	1190	71.02	542	3740	114	497.24	259.5	844.34	8.51	48.79	27.4	35.49	13912.91	10.28
p1246	Admission	40.14	6.71	33.43	8.86	17.75	212.7	16.14	54.11	2.25	33.89	7.04	21.95	37.41	55.89	18.48	16.58	5.11	590	24.48	576	1130	92.53	321.95	231.85	1220.16	12.26	62.58	156.5	0	15961.87	2.92
p1246	Admission	40.29	8.16	35.58	8.29	15.33	160.47	15.88	44.17	2.05	25.9	6.6	15.81	43.25	39.52	14.06	13	4.85	810	41.6	768	2610	124	363.69	228.82	1122.7	12.26	45.42	91.9	0	27142.53	1.5
p1246	Admission	56.27	2.95	3.71	1.32	16.2	196.59	15.11	40.1	1.09	28.37	0	38.06	16.16	45.93	12.02	15.58	1.92	0	0	0	0	0	572.37	597.9	2138.71	13.36	72.64	34.2	0	22477	3.03
p1246	D2	45.87	3.9	13.22	9.71	15.85	165.48	16.14	44.17	1.7	17.83	1.22	14.59	17.04	42.57	17.03	14.85	4.28	0	7.33	47.94	0	30.33	412.37	224.07	532.78	9.82	33.56	51.8	0	11610.91	0.46
p1245	Admission	103.1	139.28	531.71	313.92	45.27	837.55	75.39	457.26	74.81	230.94	39.22	111.17	67.29	288.96	216.36	104.96	75.6	20510	116	6023	104000	818	526.43	723.62	2500	43.07	205.3	38.2	202.47	33812	57.3
p1245	Admission	90.47	119.64	407.89	275.44	47.6	706.54	63.46	328.07	53.58	191.41	33.73	131.39	58.52	213.13	157.26	79.44	59.88	16350	141	5150	91110	728	571.59	483.53	1769.63	37.68	184.06	35.6	162.55	108694.5	42.86
p1245	Admission	108.01	137	514.88	371.65	47.84	1395.41	99.14	390.19	62.6	463.17	34.9	480.84	58.21	523.85	163.84	91.66	92.01	14040	109	5449	83810	665	1420.84	1121.83	9844.02	39.85	237.46	38.9	297.52	383712.4	82.96
p1245	Admission	100.12	86.98	351.46	198.02	36.28	649.12	53.75	269.95	44.15	1183.05	25.25	1038.46	38.3	371.41	121.19	53.83	55.24	5560	116	5293	40440	392	1208.55	2643.31	9323.89	30.55	147.95	80.3	200.03	514044	56.13
p1245	D2	52.5	55.51	271.38	141.49	21.34	323.31	31.87	208.24	27.8	115.03	15.91	69.38	20.02	114.18	106.04	38.08	30.33	2340	49.89	2214	20750	186	353.36	174.37	1972.74	18.73	89.57	144.6	135.57	391918	16.75
p1245	D3	95.09	60.75	344.17	192.74	27.72	451.98	36.69	287.75	38.33	144.72	23.65	114.22	17.63	164.42	107.29	46.22	46.64	4320	39.74	3238	36290	326	602.1	698.05	2621.56	27.44	126.62	47.9	213.96	287865	33.6
p1245	D5	71.72	58.47	294.14	138.15	31.36	409.57	32.97	216.15	29.12	106.2	23.25	61.64	45.43	116.75	83.15	43.98	29.9	5780	120	3354	39890	412	493.94	385.5	1677.77	28.2	99.96	51.5	114.13	195310.4	22.75
p1245	D6	86.29	76.7	292.26	142.51	33.09	514.43	37.36	232.32	33.25	119.51	31.54	70.24	35.01	146.44	90.41	49.63	37.84	4820	103	3153	33930	367	633.4	476.35	1213.3	28.13	125.04	28.5	140.41	116820.1	28.71
p1245	D7	51.75	26.84	100.54	25.66	9.02	109.57	6.12	25.66	6.72	18.48	18.88	17.51	12.87	25.63	9.52	10.29	11.59	350	37.68	297	3990	36.83	2643.29	200.06	7896.07	17.72	64.48	55.1	32.98	34991.8	15.48
p1244	Admission	51.54	10.83	55.92	12.27	22.36	255.15	24.58	97.87	3.2	48.91	9.24	24.92	36.07	64.13	33.36	23.75	4.15	760	63.9	1017	6690	74.09	321.95	208.16	733.38	17.87	34.16	18	0	12428.4	4.09
p1244	Admission	37.28	8.75	44.62	6.58	17.41	208.67	19.38	80.04	2.52	34.38	7.32	20.22	30.84	51.19	24.94	18.68	3.4	700	51.1	851	5590	55.92	196.48	179.45	771.96	14.34	23.72	11.9	0	16285.4	2.26
p1244	Admission	37.44	9.93	48.66	8.86	19.98	192.57	18.62	78.27	2.89	37.83	7.89	19.88	33.29	45.93	26.65	19.02	3.77	770	55.34	916	5840	58.54	220.61	123.59	668.22	12.26	29.78	13.7	0	23053.1	2.48
p1244	Admission	42.67	14.16	68.05	13.7	20.92	212.7	16.99	91.32	3.8	37.41	12.4	22.8	31.97	47.68	27.79	19.37	5.88	620	51.1	797	5640	55.66	327.12	165.76	591.43	16.34	34.76	9.1	0	35047.1	5.39
p1244	D2	29.43	4.45	29.05	3.46	20.49	218.75	22.45	90.13	1.93	59.21	4.28	20.48	20.71	56.18	29.81	22.15	1.74	220	11.53	280	1290	13.49	289.51	112.3	576.22	13.92	21.65	9.1	0	30238.7	2.64
p1244	D3	43.95	5.57	21.43	3.74	17.06	202.63	18.7	86.87	1.81	44.79	2.5	22.93	19.59	58.83	25.28	18.51	1.5	210	7.8	323	1050	14.97	555.34	283.46	1395.8	15.1	30.64	9.1	0	19514.3	7.53
p1244	D5	45.72	19.78	88.79	18.53	17.41	232.89	19.21	86.87	5.92	29.11	17.36	22.25	41.83	43.3	19.56	19.88	7.71	990	81.54	1264	7950	105	423.21	202.19	1430.32	17.25	46.1	17.3	10.71	11733.1	9.22
p1244	D6	74.41	5.85	43.25	5.16	22.86	313.1	19.81	118.79	2.71	31.73	4.61	166.47	23.89	69.15	32.7	23.66	1.5	180	8.92	317	540	9.69	765.73	434.18	2354.9	19.52	37.68	25.6	1.64	12346.1	14.46
p1241	Admission	152.76	1.27	11.47	0.89	0	0	0	0	1.58	165.41	0	29.86	0	245.49	0	5.31	1.26	460	2.4	0	320	10.55	1535.04	1375.62	3041.52	7.61	115.57	111.7	42.28	15586.08	11.59
p1241	Admission	88.02	2.95	14.93	1.75	0	285.6	0	0	2.58	1089.17	1.22	101.99	0	681.8	0	6.73	1.26	560	2.4	0	380	0	1720.75	8419.94	2560.2	22.19	214.83	45	36.57	105866.8	19.68
p1241	Admission	69.1	4.17	14.93	1.04	0.22	111.56	1.04	1.29	2.46	1068.11	2.1	262.27	0	79.54	0	9.02	2.47	480	1.9	0	380	0	3063.31	6366.44	3591.38	16.17	136.95	36	46.54	166201.9	25.84
p1241	Admission	48.6	5.01	19.85	2.6	0	23.48	0.58	0	1.87	113.83	4.28	66.89	18.67	15.89	0	6.73	2.1	550	4.78	0	380	0	1293.85	687	1468.76	12.92	45.93	36	63.78	342840.8	13.98
p1241	D5	72.31	2.29	13.22	0.32	0	7.71	0	0	1.47	22.14	2.5	19.44	0	7.25	0	6.25	1.62	720	4.78	0	390	4.5	813.29	605.88	1428.17	0	41.2	6.9	16.45	161076.8	8.75
p1241	D6	102.34	8.16	53.31	7.72	1.37	35.31	1.32	5.89	2.83	37.27	11.67	29.82	0	13.9	0	9.1	5.5	3540	38.91	1364.03	2020	179.57	699.15	601.7	862.7	1.08	46.44	3.3	65.53	177186.6	10.87
p1240	Admission	37.04	12.03	38.4	7.15	1.91	29.39	2.05	3.7	2.28	14.14	8.3	13.9	15.13	5.7	0	6.12	5.24	560	43.3	574	5440	62.75	583.9	106.37	1661.6	0	33.56	39.6	15.31	36497.2	2.92
p1240	Admission	37.77	5.29	19.85	3.46	0	0	0	0	1.41	8.91	2.88	15.22	8.71	0.41	0	4.4	2.96	350	31.56	256	3970	32.82	526.77	122.02	382.14	0	27.54	38.2	28.63	68662.6	2.42
p1240	Admission	33.55	7.58	31.98	5.16	0	7.71	0.2	0	1.87	9.85	4.61	7.37	5.71	0.14	0	5.05	3.59	210	20.06	104	2550	17.6	400.9	101.7	2515.53	0	25.68	35.3	5.1	75879.8	1.08
p1240	D1	88.93	9.93	29.05	8.01	0.7	13.63	0	0	1.87	19.37	3.95	16.53	0	1.79	0	5.38	4.85	70	4.5	0	480	6.8	626.38	279.51	1771.6	0	34.36	26.3	22.78	47957	3.25
p1240	D5	32.09	2.95	9.67	2.03	0	0	0	0	1.61	14.22	2.88	5.17	0	0.55	0	4.02	1.62	70	3.76	0	860	8.56	511.97	106.81	862.7	0	23.21	17.7	16.07	17459	0.98
p1240	D6	33.38	9.34	30.52	7.01	0.93	28.41	0.2	0	1.99	8.53	4.93	8.47	4.12	2.9	0	5.51	4.09	140	10.09	43.41	1580	14.12	478.34	129.24	1594.22	4.4	29.99	28.5	26.45	23672.2	2.59
p1238	Admission	57.08	6.32	33.87	6.87	8.37	68.45	5.76	7.87	2.47	33.27	8.45	51.45	0	11.22	5.46	7.72	3.89	1310	0	843.44	2010	97.95	311.71	735.04	340.35	7.02	25.11	83.2	3.57	8990.341	9.54
p1238	Admission	50.68	3.3	22.06	1.96	4.73	40.7	4.58	3.2	1.2	46.51	6.76	22	0	8.44	3.41	7.37	3.04	2250	16.07	1213.28	3780	155.83	337.83	549.96	353.48	4.48	25.99	96.9	0.84	17826.12	6.86
p1238	Admission	61.61	3.05	22.78	1.96	3.74	55.73	4.51	3.2	1.88	43.66	8.86	18.74	0	11.84	3.57	6.68	3.15	850	0	160.84	510	12.61	398.31	276.71	319.1	6.08	24.52	13.2	0	27606.31	6.06
p1238	D1	37.43	3.05	29.8	5.08	4.73	43.18	5.1	6.55	1.88	28.13	7.63	29.99	0	8.44	5.15	6.94	3.25	3400	33.11	1414.62	4270	202.39	278.13	266.5	218.53	5.83	24.81	9.4	23.38	97852.63	6.41
p1237	Admission	95.81	5.3	27.04	8.65	3.32	14.13	5.23	6.12	1.13	99.32	0	13.12	12.08	17.36	0	4.43	3.99	580	33	442	5090	54.35	641.34	599.14	1066.75	0	37.09	35.5	0	60537	13.63
p1237	Admission	161.4	9.39	55.57	14.45	10.33	41.94	6.77	10.8	3.11	46.85	3.87	18.08	17.29	9.99	2.93	6.42	5.93	870	49.89	840	8410	109	479.7	825.91	1035.96	0	41.05	49	17.37	38237.2	18.24
p1237	D1	62.44	7.85	50.62	10.88	7.53	23.6	6.97	9.21	2.76	119.62	0	23.33	14.32	11.53	1.12	5.64	4.76	600	39.74	448	5190	49.14	510.25	392.64	584.02	0	37.36	22.6	0	25768.4	13.71
p1237	D2	35.77	1.08	18.37	0.16	0	0	3.99	1.66	2.54	80.86	0	10.68	0	3.13	0	4.6	2.73	80	6.08	0	860	5.96	694.82	386.91	994.63	0	25.4	23.3	0	15930.4	9.37
p1237	D3	46.43	6.32	56.19	10.44	7.53	30.86	6.97	11.96	5.39	72.1	5.39	20.82	13.59	12.14	2.28	5.64	4.92	400	43.9	399	4310	55.4	783.47	527.03	675.6	0	39.08	40.2	11.23	168589.5	17.36
p1237	D4	30.02	5.18	55.57	9.1	5.57	26.01	5.37	11.03	5.12	130.89	1.35	17.49	16.12	13.07	0.95	5.12	4.21	630	45.09	580	6130	56.97	796.94	483.25	1040.08	0	30.4	20.8	3.57	66769.3	12.97
p1237	D5	129.39	3.05	65.26	5.98	3.74	9.52	5.23	9.21	7.94	92.62	4.9	20.98	5.65	5.33	0.61	4.6	3.15	240	14.53	229	1970	32.32	766.01	944.9	1172.12	0	38.68	75.2	9.36	37394.8	18.16
p1236	Admission	144.53	11.2	51.87	15.12	11.17	53.21	6.43	15.27	2.54	7.15	12.59	6.71	31.97	6.58	3.57	5.46	5.03	1680	128	1519	14660	143	357.76	208.86	417.53	0	30.82	32.6	63.84	12876.2	14.04
p1236	Admission	144.08	9	42.39	10.88	8.09	38.23	6.3	11.96	2.11	27.75	3.87	5.11	20.89	5.17	2.28	5.29	4.54	920	79.98	924	8460	75.41	394.3	224.9	637.04	0	26.85	30.4	13.09	17277.1	10.73
p1234	Admission	63.17	1.32	37.85	2.85	0	0	4.31	2.42	0	17.23	0	3.14	5.58	3.29	0	1.68	2.53	270	12.26	222	2990	24.29	1648.17	225.98	887.54	0	33.58	34	0	15074.4	11.14
p1234	Admission	24.53	3.3	44.32	5.08	2.19	0	4.97	4.01	0	13.39	0	4.11	0	2.5	0	2.02	3.04	170	6.92	30.46	1690	15.4	602.58	126.06	611.81	0	26.85	49.7	0.84	84857.2	6.77
p1234	Admission	24.24	3.3	43.04	6.42	2.19	0.81	4.9	4.01	0.1	10.93	0	4.23	9.01	1.86	1.12	2.54	3.3	380	17.65	416	3700	38.35	435.71	88.32	292.03	0	27.72	72.2	9.36	99325.9	6.15
p1234	Admission	33.58	2.8	58.02	2.85	2.19	0	4.97	3.41	0	12.71	0	5.99	6.69	2.5	0	2.54	3.04	320	16.6	290	3440	26.92	545.03	112.32	261.55	0	31.66	25.4	0	96743	8.6
p1234	D1	43.53	1.56	58.02	29.13	0	0	4.44	1.66	0.47	20.62	0	2.74	6.34	0.89	0	2.02	2.53	530	17.12	499	4700	58.8	858.86	166.43	590.99	0	28.57	18.4	0	34254.2	8
p1229	Admission	60.02	5.56	58.63	7.32	6.55	1109.55	5.23	5.69	1.28	80.2	3.87	13.67	7.93	94.84	3.89	6.59	3.89	4520	28.4	1899.41	5990	315.74	805.93	578.42	657.52	12.63	38.42	63.5	0	24901.37	15.75
p1229	Admission	28.96	1.69	44.32	0.61	0.76	29.64	4.64	2.04	0	24.14	0	12.25	0	9.99	1.62	3.91	2.83	3320	21.08	1527.65	3880	194.73	544.69	241.73	286.55	5.31	25.99	21.2	0	83905.83	7.82
p1229	Admission	46.33	0.59	46.86	0	0	7.26	4.31	2.42	0	74.64	0	20.9	0	15.83	0.43	4	2.33	1990	14.75	1387.51	1510	160.24	581.65	477.55	840.52	3.7	40.92	17.7	0	130266.2	11.89

p1229	D1	58.61	2.06	68.23	0.16	1.62	26.01	5.23	2.04	2.9	699.26	0	92.35	20.33	58.38	1.45	7.11	3.57	9010	53.96	2385.78	8710	427.91	692.32	870.31	581.69	11.72	38.95	19.4	0	131372.7	17.44
p1229	D3	48.21	1.81	68.83	0.16	1.05	38.23	5.1	2.42	2.18	100.26	1.35	39	0	10.91	3.25	11.59	3.15	150	0	0	0	0	2097.59	980.85	639.32	9.68	40.79	17.3	0	66209.85	23.07
p1229	D4	48.42	1.56	35.2	0	0.33	33.3	4.77	1.48	2.03	37.83	0	26.58	0	7.51	1.62	8.16	2.53	650	0	0	0	11.9	2877.22	713.11	1015.34	4.18	41.05	50.5	0	41327.43	18.08
p1229	D6	58.99	5.05	49.37	4.19	3.6	51.95	5.37	5.69	1.59	34.24	4.9	18.08	5.22	9.99	2.6	7.37	3.78	440	19.65	480.03	1710	80.79	1876.63	362.34	593.31	8.1	34.13	20.8	0	30205.06	20.47
p1228	Admission	85.98	2.43	32.52	4.41	1.9	240.79	4.9	2.81	0.89	54.65	7.63	27.01	15.4	48.07	0	4.95	2.94	810	46.3	736	7700	111	2276.84	679.39	3890	34.42	207.3	25.4	2.23	37390.23	14.77
p1228	Admission	55.25	1.81	43.04	2.85	0.76	11.81	4.84	2.42	0.1	17.23	0	16.58	25.4	25.6	0	3.74	2.94	790	62	560	7700	91.2	1092.5	288.36	2404.21	16.92	127.64	14.9	4.89	175221.3	9.46
p1228	Admission	42.88	5.3	56.8	8.21	4.16	38.23	5.63	6.99	1.88	11.87	7.2	10.6	0	17.67	1.95	5.12	4.48	170	9.3	0	2700	39.6	715.45	193.23	1281.58	14.93	104.07	14.2	20.99	230024.5	9.71
p1228	D1	62.53	2.06	22.78	1.51	1.05	0.81	4.38	2.04	0	6.62	0	7.7	12.4	1.86	0	4.08	2.83	410	47.2	224	4400	56.8	759.36	255.02	1504.94	5.83	99.96	11.8	0	208867.2	7.12
p1228	D1	93.74	1.32	45.59	0	0	0	4.44	0.29	0	8	0	6.79	8.5	0.89	0	4.08	2.53	460	30.8	315	5800	80.5	1059.77	420.26	1863.31	12.87	145.86	102.3	0	139952.8	11.06
p1228	D2	108.89	2.06	46.86	1.51	0	2.87	4.18	1.48	0.97	15.95	0	7.74	3.8	6.27	0	4.6	2.53	320	21	113	4400	52	1389.81	645.23	786.39	18.12	144.04	68.8	6.18	49492.4	10.39
p1228	D3	97.11	1.81	37.85	1.06	0	16.47	5.1	0.02	2.32	31.12	0	15.99	0	37.91	0	4.69	2.83	0	7.1	0	1800	15.6	2255.71	683.97	4560.42	27.77	200.97	15.6	20.99	44521.68	18.24
p1226	Admission	40.88	0.95	16.86	0.16	2.47	26.01	4.05	0.77	0.64	15.3	7.2	4.71	0	34.42	2.93	4.6	2.83	140	0	0	0	0	1148.02	302.14	1692.38	0	26.57	15.6	0	9506.404	10.05
p1226	Admission	30.8	4.3	36.53	5.08	7.39	48.18	4.84	5.26	2.03	15.23	16.28	12.1	0	9.06	6.39	5.98	3.67	450	4.88	484.32	240	43.38	775.99	157.38	664.31	8.71	29.98	13.5	0	30764.54	12.14
p1226	D1	37.9	2.06	27.04	2.85	3.03	35.76	4.71	2.04	1.59	21.56	5.86	16.74	7.23	7.2	4.04	4.77	3.35	1240	21.8	1003.22	3230	198.64	1638.53	246.86	3328.02	6.56	25.55	27.5	0	24566.65	11.56
p1226	D2	38.13	1.81	22.78	1.06	3.46	45.68	4.58	1.66	1.74	9.33	10.03	8.7	9.02	5.17	3.57	5.81	3.15	540	26.18	651.23	2300	72.75	1343.55	202.94	503.56	7.68	21.82	33.3	0	13727.84	10.56
p1226	D4	35.29	2.3	29.8	1.73	2.47	33.3	4.44	2.61	1.13	3.8	7.63	4.87	10.91	2.5	2.93	3.31	2.63	1250	39.63	948.54	1940	180.93	1027.46	183.82	467.14	5.83	20.9	19.8	0	9414.522	10.39
p1225	Admission	63.99	3.05	23.5	0.16	0	67.17	4.18	2.81	0	318.25	0	101.99	0	983.51	0	2.88	2.94	730	5.45	820.45	80	52.52	652.51	2677.82	3671.37	5.57	77.05	42.7	57.04	5468.7	15.5
p1225	Admission	59.27	4.17	24.93	2.4	1.33	123.36	4.97	4.01	0.81	431.2	0	95.96	13.23	32.75	0.25	5.29	3.89	2940	44.57	1614.83	1810	140.82	475.27	2154.63	1243.98	0	38.82	28.6	17.37	101689.2	12.56
p1225	Admission	30.28	4.3	41.75	4.19	1.62	9.52	4.97	4.01	1.88	235.58	0	66.55	15.72	170	0	4.95	3.67	790	54.36	1091.3	3260	118.82	365.89	1199.98	684.6	10.05	54.33	29.7	59.31	386117.8	16.15
p1225	D1	23.35	2.55	34.54	1.06	0	0.81	4.44	2.04	6.34	111.21	0	49.68	0	133.81	0	6.5	3.15	3180	17.7	1463.11	3010	212.89	822.9	1070.23	1281.58	10.95	57.26	22.6	139.52	317999.3	19.44
p1225	D4	29.89	2.06	49.37	1.51	0	2.87	4.44	0.94	1.28	44.29	0	16.66	0	5.02	0	5.98	3.57	270	2.09	114.17	0	14.01	1178.27	757.8	775.47	9.87	46.33	12.8	46.76	119902.2	14.36
p1225	D5	26.51	1.32	49.37	0	0	5.04	4.44	0.61	3.18	50.11	0	16.55	0	17.67	0	6.68	3.67	340	2.76	0	0	19.53	1140.56	680.07	967.57	10.95	50.23	79.7	39.83	116070.1	16.63
p1225	D6	28.16	1.81	55.57	0.16	0	7.26	4.44	1.66	2.61	65.48	0	17.72	0	16.13	0	5.46	3.78	1410	2.76	893.93	490	120.23	1152.27	581.23	740.3	7.89	38.16	12.8	44.45	54105.85	13.05
p1224	Admission	109.76	2.06	24.93	2.63	0.62	9.52	4.58	3.2	0	52.44	0	24.51	21.39	35.63	0	3.14	3.04	590	82.63	1577	1820	126	291.07	641.87	702.53	6.9	43.38	54.6	3.57	65598.01	12.89
p1224	Admission	166.76	4.8	29.8	5.98	3.32	23.6	5.83	6.55	0.3	37.53	0	30.38	66.36	23.16	0.25	4.69	3.78	870	217	1760	2990	163	394.65	497.73	363.9	5.44	37.89	36.9	34.6	286693	12.72
p1224	Admission	151.96	6.57	29.8	6.42	5.29	26.01	5.37	7.42	0.47	61.21	0	27.99	54.01	6.89	2.28	5.29	4.1	580	197	1441	1820	120	250.52	584.57	430.05	4.48	38.16	28.3	19.18	275482.2	13.71
p1224	D1	98.54	8.62	37.85	10.44	6.41	35.76	6.56	9.66	1.13	75.47	0	26.86	32.91	12.3	2.93	7.02	5.2	0	127	239	0	6.93	527.33	579.72	498.74	11.81	44.54	47.5	20.99	200432.6	16.79
p1224	D2	117.87	2.55	28.43	1.96	0.19	11.81	4.84	2.04	1.59	100.35	0	43.71	4.71	36.85	0	7.2	3.78	0	25.18	0	0	0	796.61	1228.45	898.16	7.89	43.12	15.2	2.23	190467.9	14.36
p1224	D3	90.01	1.69	35.2	0	0	301.33	4.71	1.66	3.25	81.53	0	624.69	0	15.22	0	7.37	2.94	0	0	0	0	0	735.58	1194.64	827.6	6.79	47.47	34.7	70.61	242748.4	15.18
p1224	D4	104.14	1.56	29.12	0	0	0	4.44	0.29	1.74	55.8	0	32.15	0	5.64	0	6.85	2.63	0	0	0	0	0	3058.45	1085.48	1592.52	8.1	27.72	38.7	77.33	264249.2	11.89
p1224	D5	105.83	1.56	43.04	0.16	0	2.87	4.71	0.94	3.74	45.49	0	27.37	0	7.51	0	6.5	2.83	0	2.66	0	0	0	2768.2	1435.18	1609.56	8.1	39.08	19.1	140.6	163511.1	11.56
p1224	D7	88.2	8.74	66.45	9.55	7.95	45.68	5.96	13.37	3.74	24.21	8.45	15.17	75.28	12.76	3.25	6.16	4.98	4490	142	820	5960	221	724.77	522.17	1558.36	10.51	39.34	25.8	81.25	54088.83	16.79
p1222	Admission	88.99	1.56	32.52	1.06	0.04	334.13	4.31	2.42	1.59	123.31	0	27.76	0	318.93	0.06	3.4	2.83	560	4.31	0	0	40.82	566.21	1224.78	1740.98	0	50.73	21.5	29.31	7239.671	11.98
p1222	Admission	35.53	4.8	40.46	5.98	3.88	89.12	5.37	7.87	1.44	30.07	0	11.7	17.06	26.21	1.95	4.6	3.89	1870	27.53	1517.01	5110	224.75	235.01	267.42	1437.66	6.08	37.09	21.2	66.1	87295.03	10.73
p1222	Admission	42.21	5.05	39.16	6.87	6.13	28.43	5.1	6.12	1.44	29.19	0	11.62	11.33	17.67	1.29	4.26	4.21	1600	29.14	1242.17	4890	100.12	348.17	314.44	1038.02	3.19	48.22	18.7	44.45	137484	12.81
p1221	Admission	54.47	2.55	33.87	54.89	1.9	39.46	5.37	3.2	0.47	150.59	0	27.72	18.37	1223.95	8.24	5.29	5.99	1210	64.52	1319	2870	192	207.16	977.54	891.79	13.58	41.96	33.3	40.41	11313.72	13.05
p1221	Admission	115.62	2.3	58.02	0	0.48	754.62	4.71	4.01	0.2	241.68	0	109.01	10.81	66.57	1.29	5.64	2.73	540	33.78	615	1200	94.47	255.27	1912.62	412.49	9.2	28	35.1	54.2	215580.4	11.89
p1221	Admission	89.46	2.8	40.46	1.06	0.19	38.23	4.84	1.66	1.74	333.15	0	65.13	38.06	119.56	2.28	5.81	2.94	1310	79.08	1322	4430	189	359.17	1127.38	258.74	8.1	31.66	24.3	101.77	277002.2	10.39
p1221	D1	89.39	1.81	51.25	0	0.76	35.76	4.97	3.2	2.18	190.44	1.35	40.1	6.75	33.81	2.28	7.9	3.25	470	28.98	523	820	73.95	660.68	939.87	1135.87	6.79	19.03	20.1	237.63	194879	11.56
p1221	D1	26.79	1.08	25.63	0	1.62	182.75	4.71	2.42	2.32	126.23	4.9	396.87	0	12.45	3.09	10.53	2.83	0	0	0	0	0	399.71	1187.83	917.19	10.05	17.43	28.6	229.14	138756.2	11.23
p1221	D5	50.78	3.3	36.53	2.85	2.47	91.73	5.23	3.2	3.04	20.48	1.35	34.22	59.15	10.3	1.95	7.37	3.78	3000	162	2302	9050	351	831.88	399.57	657.52	9.68	21.82	12.8	64.41	30911.47	14.45
p1221	D6	123.79	2.55	51.87	0.16	2.33	115.39	4.71	2.42	3.46	22.37	1.35	43.51	17.27	8.6	3.25	8.07	3.46	1510	95.83	1628	4210	216	603.42	515.1	1019.47	7.57	23.18	112.4	170.87	27977.52	15.66
p1220	Admission	87.09	3.3	5.11	9.99	0.19	2.87	5.7	5.26	1.13	55.91	0	42.02	14.95	20.11	0	4.43	5.37	1020	47.04	1590	3310	195	7459.4	417.23	1048.74	25.49	47.72	84.7	61.01	72575.1	49.88
p1220	Admission	100.8	4.05	5.11	17.56	0	16.47	6.43	3.61	0.64	77.74	0	75.43	7.19	20.42	0	5.12	7.26	330	21.83	610	260	66.05	7926.51	504.84	2264.1	35.74	46.45	62.4	36.35	108535.6	44.92
p1220	Admission	128.92	6.7	0.61	22.01	0.19	0.81	8.45	26.17	0.81	88.61	0	49.99	1.14	21.94	0	4.95	10.57	0	8.09	359	310	45.6	573.704	276.87	1311.369	19.85	45.18	17	46.18	83604.44	28.93
p1220	D1	82.03	8.1	10.48	23.35	1.9	16.47	6.7	44.1	1.13	151.16	0	77.82	30.93	19.19	0	5.64	7.26	510	65.42	1041	1390	97.49	5360.47	425.34	8354.12	32.11	38.42	22.2	76.22	182135.8	42.39
p1220	D2	65.35	7.34	16.86	17.12	2.89	14.13	7.64	7.87	1.59	141.25	0	63.86	74.81	31.68	1.29	7.29	7.5	660	214	1314	3090	150	5074.62	383.2	3289.06	20.9	44.8	33.6	127	151409.9	50.87
p1220	D4	60.4	3.05	0.61	11.55	0	0	5.9	2.23	0.64	44	0	32.15	14.54	10.6	0	4.77	7.26	530	43.15	1116	1150	128	5991.53	239.86	1088.23	15.37	32.49	18.4	13.7	104344.1	45.83
p1220	D5	115.9	9.26	17.62	35.13	5.57	2.87	8.25	16.23	1.44	58.14	0	34.22	75.96	14.6	0.61	6.85	15.99	1600	170	2316	5020	244	5711	250.34	6404.33	25	44.54	10.8	9.98	75954.35	43.77
p1220	D7	94.59	4.55	6.99	31.35	0.76	0	7.5	5.26	0.81	63.03	0	25.33	11.7	9.06	0.61	4.43	12.77	370	46.07	564	680	72.14	6274.05	227.13	8901.48	23.03	39.61	11.1	0	57652.55	41.55
p1218	Admission	84.21	1.08	15.32	3.3	0.33	5.04	4.71	1.3	0.3	41.87	0	14.58	6.41	9.99	1.62	3.22	3.57	440	36.21	337	3610	47.07	1877.36	516.69	1050.35	0	23.33	28.3	0	24738.1	10.89
p1218	Admission	74.79	1.56	22.06	2.85	0.76	9.52	4.97	1.48	9	68.81	0	29.68	16.62	6.89	2.6	4.69	4.21	1130	101	1060	7900	99.98	1925.2	671.28	975.91	0	31.66	34.4	1.54	71598.2	16.31
p1218	Admission	68.65	3.67	37.85	5.53	2.75	18.83	5.37	4.43	8.01	70.8	0	18.39	16.45	9.99	2.11	5.98	3.78	730	73.77	724	6690	72.77	1625.33	493.63	551.21	0	38.02	18.4	35.18	81356.5	14.04
p1217	Admission	87.65	3.24	44.79	1.22	2.27	25.05	0.34	1.56	1.28	55.53	7.72	9.54	15.05	56.95	0	3.19	0	630	35.59	619	1390	93.5	334.42	509.71	1105.44	2.01	38.13	21.8	0	8922.377	6.65
p1217	Admission	43.51	3.42	18.17	0.91	2.42	25.05	0.7	0	1.68	50.18	7.72	42.13	37.19	81.59	0	2.82	0	940	61.5	915	3800	143	246.94	450.86	752	0	50.17	12.8	9.21	43793.74	6.01
p1217	Admission	51.13	3.96	57.49	2.18	3.63	19.35	1.05	0.59	1.18	74.95	4.7	143.28	40.87	54.31	0	3	0	990	71.79	1075	4350	159	198.53	654.8	845.35	2.01	50.57	10.3	30.25	116031.2	8.2
p1217	Admission	56.88	3.59	90.87	1.22	1.18	10.36	0.61	0.59	4.16	100.92	3.5	36.66	6.98	58.4	0	4.49	0	370	22.98	275	980	59.03	218.51	648.04	424.75	3.46	45.64	8.1	16.44	310110.4	8.07
p1217	D3	68.72	3.77	54.2	2.34	1.96	13.87	1.05	0.59	11.8	25.24	9.48	12.65	40.66	7.22	1.23	3.93	0	1150	80.9	1145	4910	178	346	671.42	517.85	7.22	42.2	11	14.53	88803.7	10.14
p1217	D4	75.04	3.24	89.44	0.6	1.49	15.67	0.52	0	10.31	10.12	4.7	12.6	9.23	8.08	0	4.21	0	650	30.18	670	1740	92.53	665.99	632.51	900.95	8.09	45.22	12	9.9	46201.3	10.53
p1217	D5	106.1	3.59	89.44	1.54	2.42	28.94	0.7	2.28	9.22	29.24	9.48	63.08	0.77	13.93	0	4.68	0	360	15.17	384	460	48.43	861.34	1147.37	1457.58	14	74.31	12.4	22.59	30249.66	17.1
p1217	D6	75.49	3.84	74.75	3.31	3.63	44.1	1.05	3.01	4.69	12.3	14.82	14.5	14.38	10.64	0	4.49	0	700	27.18	854	1530	102	897.16	572.22	1507	10.98	34.05	12.4	22.59	32352.05	13.39
p1215	Admission	24.46	4.45	89.44	5.45	3.03	21.23	1.23	6.86	3.64	22.93	0	13.84	60	30.06	17.8	2.64	0	3440	131	2241	12730	378	2271.55	134.47	2201.83	1	38.59	23.2	48.78	18255.84	4.46
p1215	Admission	22.73	5.09	77.75	5.12	4.24	15.67	1.76	6.38	3.33	19.56	0	12.7	41.09	19.73	15.12	2.54	0.27	2860	116	1835	9910	322	1285.71	92.42	1736.79	0.56	34.79	16.4	62.86	35332.65	4.21
p1215	Admission	23.36	5.09	70.94	3.48	3.03	11.22	1.76	4.46	2.34	17.88	0	12.17	78.52	13.81	9.51	2.09	0	2960	190	2410	14010	380	1111.71	93.88	1180.78	1.54	34.79	13.5	46.58	38964.27	3.44
p1215	Admission	25.9	4.77	77.75	3.8	3.94	17.5	1.58	4.94	2.91	14.36	0	10.58	41.52	15.26	14.34	2.64	0	2200	94.61	1750	8230	289	1054.38	109.82	906.67	2.58	36	6.7	53.69	42368	4.14
p1214	Admission	35.05	3.59	104.8	0.91	2.73	34.9	0.34	2.53	2.19	304.57	15.52	29.96	0	170.74	8.67	7.38	0	710	0	492.81	0	47.57	256.15	1231.02	1526.34	5.32	94.24	34.4	0	3378.022	8.4
p1214	Admission	39.58	4.2	65.51	1.22	3.33	268.6	0.34	3.49	3.27	380.62	17.2	92.54	0	49.14	8.88	10.75	0	520	5.37	711.34	290	45.9	303.54	2164.1	1125.73	5.12	46.07	18.5	0	55903.13	10.27
p1214	Admission	23.11	4.27	138.75	0.29	1.8	74.18	0.34	3.73	2.96	200.72	11.11	61.62	0	62.97	2.81	8.96	0	1960	8.33	914.07	1270	132.86	240.66	1171.93	577.74	2.97	32.55	20.7	0	77508.09	6.59
p1214	Admission	11.17	3.71	133.07	0.6	3.63	28.94	0.34	1.07	1.79	39.65	16.2	26.18	1.72	314.27	4.49	8.96	0	2410	14.77	1238.15	2550	130.08	149.21	398.01	366	4.05	36.24	20.3	9.21	209482.9	6.07
p1214	D2	48.74	3.84	183.17	0.29	2.73	64.3	0.7	0.1	2.19	323.83	15.86	182.84	0	53.1	3.3	15.64	0	1730	5.37	1019.43	1170	136.68	1369.36	2648.95	702.65	15.75	78.54	17.8	0	146320.5	23.84
p1214	D3	17.97	4.39	140	2.83	5.43	36.92	1.76	2.53	1.89	46.63	16.2	43.09	14.43	17.43	4.26	10.75	0	1090	47.61	1481.2	7090	215.78	1505.61	1089.56	716.53	13.87	45.22	13.1	0	49631.01	12.61
p1214	D4	23.85	4.08	159.62	1.22	3.03	35.91	1.58	0.59	2.19	26.22	14.82	39.03	1.72	17.55	4.72	10.95	0	3490	10.2	1513.45	4500	230.8	1993.46	1191.79	1423.91	5.12	42.2	11	9.21	27175.53	13.84
p1214	D5	18.8	3.71	119.52	1.22	2.27	30.91	0.52	0	1.79	26.3	13.39	28.36	1.35	18.88	1.23	8.56	0	1520	6.52	1091.3	2480	134.25	906.77	942.59	1439.87	4.16	38.59	13.5	8.52	27877.29	11.05
p1214	D6	23.73	3.9	173.86	1.86	3.03	34.9	0.7	0.1	2.91	35.12	13.39	25.17	0.59	20.45	4.26	10.05	0.12	1430	7.71	936.34	1690	99.75	704.78	964.01	1034.89	5.42	37.66	12.8	12.58	13819.06	11.57
p1213	Admission	27.88	3.59	100.68	3.15	5.87	0	1.58	14.54	8.78	146.49	9.48	2.57	6.2	2.35	0	0.54	4.57	450	24.74	486	4110	64.32	857.68	305.94	714.55	7.84	40.64	25.7	24.38	25540.4	6.33
p1213	Admission	13.82	3.36	96.51	0	0	0	0.16	0	0.22	11.23	0	0	0	0.52	0	0.46	0	110	5.67	0	1050	7.49	165.88	119.13	484.34	1.7	19.53	15.3	0	30522.8	4.59
p1213	Admission	20.41	6.71	120.83	9.62	8.95	25.05	3.17	9.99	1.48	22.14	5.78	4.24	15.38	5.36	3.54	2.09	2.84	760	66.97	638	6510	59.33	102.55	122.21	241.02	7.04	39.51	11	22.59	26434.5	8.46
p1213	D1	26.49	4.33	99.3	3.15	2.42	2.36	1.58	2.04	0.6	20.21	0	5.38	0	4.11	0	1.3	0	230	11.04	63.38	1750	23.34	222.89	163.47	458.95	3.1	30.22	12.4	0	31082.3	6.65
p1213	D2	26.37	3.48	136.23	0.6	0	0.4	0.7	0	0.79	54.71	0	4.73	0	6.1	0	2.18	0	150	8.7	24.16	1410	14.12	695.67	239.59	1521.07	5.22	35.76	14.9	11.26	14824.4	8.91
p1213	D3	42.85	3.71	116.22	1.86	1.18	3.09	1.23	0.35	1.63	22.62	0	5.76	4.64	3.99	0	2.27	0	350	21.97	303	2950	37.84	527.04	303.68	1379.4	6.3	34.79	13.8	8.52	34890.3	8.46
p1213	D4	38.09	3.71	144.98	0.91	0	0	0.52	0	1.43	14.09	0	3.96	0	1.83	0	1.39	0	120	6.5	0	860	11.64	938.4	372.93	1594.63	5.52	37.66	25.7	15.81	10724.8	10.92
p1213	D5	38.09	3.36	85.84	0.6	0	0	0.52	0	0.94	6.61	0	2.2	4.64	1.18	0	0.7	0	300	17.65	327	2830	41.66	833.52	325.84	1510.52	0	28.88	16	11.92	12135.8	8.2
p1211	Admission	18.11	4.83	70.17	5.45	3.63	5.38	1.58	3.01	0.7	108.34	3.5	7.07	17.12	33.18	0	0.87	0	700	78.74	803	6080	54.87	206.08	321.43	933.29	3.1	44.37	36.2	17.7	13159.3	6.2
p1211	Admission	19.88	5.22	57.49	5.12	5.13	7	2.29	4.94	0.99	173.28	6.78	9.19	15.13	10.03	0	1.39	0.41	690	71.29	710	6280	54.61	198.23	261.75	763.76	7.13	42.2	33	7.09	36247.6	6.65
p1211	Admission	19.21	3.59	65.51	0.91	0	0	0.34	1.56	0.18	228.87	0	20.55	0	3.36	0	0.87	0	120	8.24	63.38	1050	10.85	212.54	382.43	1229.99	13.8	83.77	30.1	9.21	48665.6	9.75
p1211	D1	19.35	3.84	74.75	2.02	0	0	2.11	0.83	0.5	229.92	0	16.41	10.22	11.01	0	0.96	0	380	37.97	249	4550	27.41	297.85	389.16	1186.27	2.71	33.31	35.5	5.6	49905.7	7.56
p1211	D2	13.5	3.36	68.63	0.6	0	0	0.52	0	1.68	282.59	0	11.06	7.4	4.36	0	1.91	0	370	31.27	256	3250	29.85	340.72	289.86	1036.75	4.49	28.05	29.7	0	22261.2	8.01
p1211	D3	15.81	3.48	83.66	0.6	0	0	0.34	0	2.5	234.12	0	6.75	4.64	0	0	0.96	0	170	19.25	66.76	2010	15.84	754.78	396.38	1184.44	7.84	30.22	41	0	16794.9	7.62
p1211	D4	20.94	3.77	82.19	2.5	0	0	0.7	0.1	3.64	218.51	0	6.65	0	1.83	0	1.39	0	120	12.51	0	1520	7.14	1002.71	416.27	1471.73	4.7	36.96	38.8	4.83	15711.9	9.88
p1211	D5	25.9	10.43	97.91	14.53	16.97	53.54	5.27	13.1	8.51	133.38	13.75	11.45	14.64	10.28	5.63	6.99	6.22	710	61.45	812	8000	76.99	493.99	384.38	1423.91	17.29	68.26	40.289	67.14	12474.1	15.8
p1211	D6	19.48	4.45	55.85	4.46	1.49	1	1.58	1.07	13.35	117.9	0	4.9	23.35	2.6	0	1.3	0	1430	104	1367	12470	124	705.26	297.15	1538.64	9.58	43.07	34.8	12.58	12985.8	8.65
p1210	Admission	30.68	8.3	67.07	9.12	11.84	41	4.22	13.1	1.79	32.64	9.48	5.76	7.89	23.82	5.18	3.56	3.97	200	38.12	0	3400	0	222.89	162.93	732.33	3.1	37.19	25.3	38.8	18626.9	9.37
p1210	Admission	19.07	3.84	83.66	0.91	0	0	0.7	0	0.13	75.26	0	2.69	0	0.52	0	0.87	0	90	20.56	0	1640	0	181.88	166.08	475.9	1.7	16.67	14.6	0	59472.4	5.24
p1210	D1	30.24	12.35	99.3	8.95	10.4	21.23	3.78	14.06	1.89	12.11	8.62	9.34	16.49	8.81	4.26	3.28	2.84	790	75.43	702	7810	121	203.91	168.15	2121.8	5.32	45.22	19.4	46.03	32823.2	12.74
p1210	D2	29.13	4.27	85.84	1.22	0	0	0.7	3.01	0.89	5.64	0	4.95	0	0.92	0	1.04	0	200	24.57	0	1930	0	210.25	148.18	1171.64	5.72	23.71	42.1	0.2	16418	7.81
p1210	D3	24.46	4.08	69.4	1.22	0	0	1.05	0.1	0.89	5.51	0	0.91	0	0.79	0	0.96	0	90	24.57	0	1550	0	291.62	169.9	1136.78	5.52	19.87	14.9	0	15270.5	7.62
p1210	D4	26.37	4.33	81.46	1.54	1.18	0.4	1.14	1.56	0.84	89.45	0	5.81	4.39	3.74	0	1.04	0	170	31.87	0	3530	0	415.2	148.54	1313.12	4.49	29.69	39.3	0.2	13291.8	8.98
p1209	Admission	57.88	6.58	67.07	8.11	10.69	100.18	3.52	10.71	1.33	80.65	8.62	34.33	31.8	35.58	4.72	4.21	2.41	1210	54.7	976	9000	141	171.33	895.18	1596.38	3.1	35.52	19.7	31.98	81604.58	11.05
p1209	Admission	41.61	4.45	43.9	3.15	2.73	1	1.32	2.53	0.6	55.47	0	37.89	5.5	15.38	0	2.73	0	140	16	0	2100	23.2	138.71	694.29	1334.67	0.56	18.84	12.2	0	273157.5	6.14
p1209	Admission	30.68	4.52	33.78	2.83	3.78	0	1.32	3.01	0.79	47.87	0	25.96	12.2	8.08	0	3.84	0	350	24.6	67.7	3900	58	157.64	518.91	999.33	0.56	21.52	11.9	13.89	347080.6	7.3
p1209	D1	30.46	3.71	72.47	1.54	0.7	0	0.7	1.07	0.32	65.75	0	29.79	9.3	20.69	0	2.82	0	260	23.7	36.4	3300	38.3	177.94	560.83	891.4	1	20.87	12.5	11.26	247365.7	8.01
p1209	D2	17.12	3.59	88	0	0.21	36.92	0.52	0.1	0.94	98.36	0	485.77	0	10.52	0	5.25	0	0	0	0	0	0	170.87	921.5	734.3	4.49	18.13	36.5	18.94	111196.9	9.37
p1209	D3	22.48	3.36	99.99	0.29	0	0	0.34	0	1.33	73.35	0	14.26	0	5.86	0	6.02	0	0	0	0	300	0	412.51	661.01	798.89	4.91	18.13	8.5	4.04	55330.08	11.18
p1209	D4	23.97	4.08	114.23	1.22	1.18	3.83	1.05	0.83	0.79	49.09	0	12.51	7.2	5.36	0	3.65	0	270	22.8	127	3100	42.1	365.98	505.16	835.7	5.02	20.87	11.9	0	47919.3	12.48
p1209	D5	23.73	3.96	95.11	1.54	0.21	1.66	0.96	1.56	0.99	35.66	0	12.41	6.8	6.35	0	4.49	0	260	19.2	44.4	3200	44.6	584.59	552.14	1164.31	3.58	23.41	9.2	0	36690.99	11.83
p1209	D6	35.15	5.42	89.44	7.44	6.61	10.36	3.34	8.78	1.89	23.09	9.48	14.87	25.2	9.55	2.81	5.25	2.19	930	51.4	660	6800	89.1	429.32	446.9	1302.33	5.72	34.79	9.2	26.16	32002.92	12.48
p1208	Admission	64.55	4.96	49.13	2.5	10.97	1959.51	1.41	3.49	6.08	399.08	34.29	94.31	16.79	162.44	15.89	18.54	0	2740	28.1	1527.65	2100	134.25	460.43	1710.65	3026.63	19.21	95.56	25.6	36.54	39163.43	16.77
p1208	Admission	71.41	4.83	95.81	1.22	7.78	641.7	0.88	3.49	4.85	658.81	24.8	162.12	13.11	49.98	8.25	12.77	0	1410	27.33	919.06	520	72.37	386.02	2759.97	2500	20.59	118.68	15.6	46.03	143074.1	16.06
p1208	Admission	57.05	4.2	45.67	1.22	5.43	104.8	0.88	3.49	3.22	875.28	18.82	157.72	11.56	126.98	5.63	10.25	0	3240	35.67	1343.99	2350	154.84	378.62	2119.41	1608.59	10.9	61.93	13.9	94.5	188074.6	10.92
p1208	D1	33.38	4.02	63.93	0.75	3.03	55.68	0.7	1.07	4.8	224.12	16.2	68.56	11.05	51.9	6.96	12.77	0	1760	27.33	1147.33	1780	128.33	538.4	817.8	1665.94	12.6	34.3	11.2	141.45	184711.9	9.62
p1208	D2	25.9	3.71	58.31	0.29	6.31	171.9	0.7	1.56	3.64	60.99	22.49	772.98	6.69	22.61	7.82	15.02	0	50	0	0	0	0	613.93	1395.43	2016.08	14.95	54.49	44.2	211.49	105258.2	12.02
p1208	D3	36.18	4.2	49.13	1.22	3.48	57.82	1.23	0.83	3.22	73.03	17.53	59.99	20.56	20.93	5.63	13.08	0	3200	51.58	1232.1	2310	131.47	1044.26	1207.71	1524.59	12.17	58.64	10.8	164.99	93597.77	14.04
p1208	D4	31.34	4.08	42.12	1.86	6.9	93.3	0.88	0	2.91	65.2	23.36	37.8	12.59	16.83	10.34	17.81	0	720	14.04	563.95	440	58.2	1275.08	923.62	1345.43	11.43	65.48	13.2	198.71	67901.27	14.69
p1208	D5	54.94	4.71	54.2	1.22	10.4	133.04	1.5	1.32	12.63	28.36	25.36	48.41	14.15	19.48	11.96	20.63	0	610	33.41	578.69	420	43.38	2871.66	1157.54	2079.94	24.84	94.68	32.2	243.73	35066.22	27.38
p1208	D6	54.17	4.96	65.51	2.83	9.24	104.8	1.58	1.07	12.19	31.52	27.57	50.56	34.19	16.71	14.73	20.84	1.17	6980	67.18	2093.01	5780	270.36	2281.18	966.08	1469.96	18.54	83.92	38.6	253.99	36282.76	24.89
p1205	Admission	201.93	141.6	92.29	3.15	3.94	48.27	3.69	3.49	1.38	23.71	7.72	7.99	8.36	8.57	0	3.37	0	540	62.86	672	6050	58.26	446.9	322.36	540.69	0	56.39	34	0	14346.1	7.04
p1205	Admission	127.81	118.42	76.25	3.8	3.03	37.94	3.17	3.01	1.18	13.25	4.7	4.4	4.39	6.97	0	2.82	0	310	43.66	473	4090	31.63	326.95	144.73	413.98	0	42.42	15.9	0	19843.3	6.46
p1205	D1	156.68	84.93	88	6.11	6.76	59.97	4.05	6.38	1.63	10.52	10.31	16.82	3.91	9.06	0	2.64	1.29	280	34.51	0	3400	0	221.2	116.28	632.62	0	50.57	10.2	17.7	41949.9	7.23
p1205	D2	174.1	140.19	89.44	2.18	3.03	38.96	3.17	1.56	1.48	10.12	8.62	3.73	0	8.81	0	3.74	0	120	16.55	0	1420	0	603.55	194.11	761.81	0	57.9	21.4	0	16356.1	9.04
p1205	D3	167.6	147.14	83.66	2.34	3.03	59.97	2.11	0.59	1.58	0	6.78	2.98	0	4.74	0	2.82	0	0	11.9	0	1040	0	511.25	122.25	952.23	0	50.77	29.4	0	10779.1	8.33
p1203	Admission	100.14	9.65	87.28	14.53	17.82	100.18	5.62	16.46	4.06	12.97	20.68	8.24	82.97	24.78	10.75	9.65	5.06	2280	189	2046	9840	302	165.09	236.73	712.57	24.89	55.06	56	55.86	5046.663	10.53
p1203	Admission	55.19	4.96	30.87	4.13	5.72	29.92	1.76	3.97	1.58	13.44	8.62	5.38	107	7.09	4.26	3.56	0	2430	200	2241	9950	325	122.48	163.9	668.78	9.66	33.81	30.8	8.52	8893.206	5.75
p1203	Admission	64.71	6.71	59.12	5.78	8.95	42.03	3.17	7.34	2.4	24.63	15.52	7.17	91.42	16.95	6.96	5.16	1.97	2150	175	1796	8030	315	141.07	203.36	509.51	13.8	43.51	25.6	29.67	18500.5	6.85
p1203	D1	83.82	8	105.48	11.81	14.98	62.13	4.4	10.23	2.91	21.91	14.82	7.58	155	13.68	7.39	8.16	4.27	7040	316	3503	17430	598	166.19	233.78	454.7	14	51.17	11.9	52.06	36792.38	10.14
p1203	D2	198.06	12.51	133.07	23.46	24.85	121.17	7.19	19.33	4.8	9.91	21.89	10.97	136	17.68	14.73	11.86	8.35	6440	314	3642	16190	623	208.68	464.41	833.77	20.35	68.26	12.9	80.37	13941.14	16.51
p1203	D3	88.71	5.62	82.19	5.78	6.9	53.54	2.29	4.94	2.96	5.89	11.89	4.51	74.49	9.18	6.08	7.38	1.64	2360	154	2154	7890	319	231.55	232	1112.83	11.58	41.31	21.8	21.38	7252.561	8.59
p1203	D4	107.21	3.96	76.25	1.54	6.17	45.13	0.96	2.53	2.19	5.13	13.39	17.92	6.75	10.28	4.02	5.83	0	0	10.96	422	0	18.54	238.05	401.74	1824.32	40.57	74.31	36.5	8.52	5782.594	12.74
p1202	Admission	37.39	5.49	55.85	5.45	5.72	219.56	2.64	6.62	2.5	178.54	16.87	31.42	7.9	82.32	6.96	4.12	1.05	5230	50.29	1891.49	6340	277.48	794.05	900.03	2342.54	10.98	64.07	80.5	56.4	14260.09	18.73
p1202	Admission	31.77	4.77	73.23	3.8	4.98	50.37	2.11	3.97	3.27	380.49	14.11	65.85	4.1	24.42	4.26	5.25	1.05	6220	26.73	2091.49	7330	345.88	472.12	701.97	1817.48	11.58	43.29	72.1	46.58	164095.1	15.86
p1202	D1	24.46	4.64	63.93	2.5	3.33	36.92	2.29	1.07	2.6	252.1	7.72	53.07	7.52	32.22	1.78	6.21	0.55	530	25.93	1817.84	2470	162.27	472.84	660.84	1962.06	11.06	41.76	66.7	71.92	176197.7	16.25
p1202	D3	26.84	3.48	88	0.29	1.96	41	0.43	0	1.09	39.58	9.9	33.01	2.19	8.57	1.51	10.15	0	1040	9.98	831.26	790	134.95	737.2	464.7	829.91	8.93	33.81	35.4	90.33	157682.1	16.51
p1202	D4	32.52	3.59	52.52	0	0.86	36.92	0.52	1.07	1.18	33.4	7.72	25.35	1.24	6.35	0	8.07	0	760	13.56	753.04	610	69.65	615.12	296.76	503.23	5.52	32.3	37.5	74.57	95276.37	13.78
p1202	D5	101.47	4.08	28.88	4.46	12.56	337.58	4.4	0	1.38	100.22	8.62	104.19	1.43	452.36	1.78	14.71	4.67	1040	13.56	755.94	1490	166.96	2964.06	3974.33	808.6	107.44	513.68	29.8	37.67	120947.1	60.23
p1202	D6	32.2	4.2	52.52	11.48	4.53	224.66	4.22	2.04	1.28	162.36	19.76	946.16	0	100.82	2.3	14.2	4.87	80	3.25	0	0	0	3972.39	918.81	4295.93	19.63	71.65	45.6	144.52	413967	46.4
p1201	Admission	32.02	11.91	57.78	15.22	2.94	86.86	0	5.58	4.51	50.88	12.14	6.93	0	7.17	0.08	4.08	5.96	880	3.77	0	0	0	606.39	281.2	1498.84	2.78	34.11	38.7	27.4	7393.072	10.1
p1201	Admission	22.46	9.33	44.02	16.51	1.3	19.46	9.73	2.84	2.75	85.36	10.22	16.49	0	11.85	0	4.54	5.96	2380	13.56	805.63	430	102.38	600.67	352.29	1233.92	6.17	37.45	36.5	41.72	235979	10.97
p1201	D1	22.21	13.38	51.43	26.07	3.21	34.71	6.22	4.1	2.75	78.34	10.93	18.26	2.77	4.03	3.09	6.44	10.96	3440	21.19	1413.54	4580	142.8	679	280.78	960.26	6.39	42.84	40.6	86.71	291833.6	9.88
p1201	D1	29.25	10.44	72.94	30.82	0	0	5.38	6.22	2.42	77.16	14.3	23.69	0	11.14	0	8.29	12.96	0	1.88	0	0	0	1153.88	433.42	954.36	7.16	40.07	52.3	94.18	163459.8	21.43
p1201	D2	30.76	14.11	0	28.29	0	211.33	14.5	0	2.09	72.15	3.65	107.32	8.66	32.69	9.19	7.86	11.63	0	1.5	0	0	0	1977.38	1183.11	2397.91	4.5	57.45	55.7	453.52	80848.72	33.3
p1201	D3	34.68	13.56	50.62	48.1	0	0	3.29	3.68	3.71	85.1	11.63	24.61	77.26	31.33	0	7.32	21.84	0	5.45	0	0	0	4602.09	618.44	1228.27	5.58	50.7	53	356.57	93141.95	37.97
p1201	D4	48.14	21.37	127.25	55.6	5.01	31.99	0.66	18.09	5.44	84.2	21.97	26.37	33.59	31.67	0.51	15.8	26.37	370	1.5	0	0	0	2144.03	453.33	2026.21	7.84	58.62	54.5	937.64	172557.5	31.79
p1201	D6	40.69	11.18	60.12	25.27	0.65	0	0	1.16	1.88	113.99	5.94	27.11	19.16	25.15	0	6.55	16.39	0	3	0	0	0	1174.55	483.46	1471.5	1.46	37.67	46.6	1069.11	59450.98	29.85
p1200	Admission	163.87	0	0	0	0	0	0	0	0.64	52.07	0	11.44	0	4.59	0	0	0	0	0	0	0	0	107.62	683.65	609.16	0	29.98	51.1	0	3051.602	3.43
p1200	Admission	47.18	7.84	14.8	2.19	0.09	6.19	0	0	1.45	53.74	4.14	7.13	37.19	0	0	0	0.34	1430	106	1434	6130	192	114.45	185.82	78.67	0	21.43	69.9	0	14100.63	0.81
p1200	Admission	34.68	6.71	22.2	2.53	0	0	0	0	1.17	49.34	4.14	6.64	41.52	0	0.71	0	0.83	1180	99.81	1196	4350	170	113.07	233.9	202.05	0	19.61	36.1	0	15804.03	2.63
p1199	Admission	36.47	13.75	38.03	7.14	2.13	34.71	0.37	0.32	1.59	19.9	7.58	8.1	19.7	32.01	0	2.9	3.72	1360	73.31	1393	4460	213	142.75	240.66	401.84	0	29.51	51.5	10.38	23608.8	4.96
p1199	Admission	21.96	5.57	4.7	3.53	0.09	0	0	0	1.45	96.8	1.04	24.53	43.25	1.93	0	0.56	0.83	1400	114	1455	5690	239	80.06	281.74	291.31	0	21.18	26.7	32.3	264768.8	2.19
p1199	Admission	19.8	8.02	19.13	4.68	0.65	0	0	0	1.8	70.03	3.65	14.81	57.44	0.73	0	1.13	1.98	1440	123	1263	7830	192	81.14	252.45	256.63	0	22.45	42.5	20.08	241326.4	3.8
p1199	D1	19.8	4.02	10.11	6.98	0	0	0	0	2.02	77.29	0.65	14.95	0.29	8.08	0	0	3.22	480	30.18	541	890	77.89	143.31	232.12	335.91	0	19.61	33.5	51.17	183747.4	3.72
p1199	D2	19.54	8.96	21.19	6.81	0.84	6.19	0	0	1.52	102.91	5.29	12.22	48.85	5.89	24.04	1.53	2.31	1550	125	1532	6420	211	117.8	196.36	252.94	0.19	20.14	20	38.85	39610.7	3.94
p1199	D3	18.08	2.03	0	21.46	0	0	0	0	1.24	42.73	0	9.82	0	0	0	0	5.13	0	6.72	81.87	0	27.69	182.43	154.67	260.32	0	15.54	17.7	45	32006.83	2.78
p1199	D4	15.55	8.58	30.86	16.83	1.58	18.04	0	0	2.67	69.63	4.61	11.37	0	6.81	0	0.56	4.71	0	0	0	0	0	334.23	179.7	148.84	2.6	24.21	12.8	7.17	23723.27	5.47
p1199	D5	23.07	10.81	34.49	6.49	1.58	45.46	0	0.95	1.88	27.62	9.85	13.04	0	0	0	0	2.97	0	2.42	0	0	0	297.61	213.14	330.61	0	28.33	30.1	110.81	27809.14	10.61
p1199	D6	12.81	2.03	3.11	1.86	0.09	124.3	0	0	0.9	18.93	8.74	57.3	0	1.53	0	0	0.34	0	0	0	0	0	274.53	226.38	636.13	0	34.11	20	147.94	40879.45	15.23

p1198	Admission	82.58	1.61	4.7	2.53	1.76	56.01	5.98	7.91	0.84	88.06	0	28.89	2.65	121.29	3.26	3.26	1.9	220	2.87	0	70	2.45	513.11	616.47	1967.73	12.77	173.5	69.6	0.08	9734.225	19.12
p1198	Admission	29.04	2.03	7.54	5.18	2.13	94.43	3.03	2.21	0.77	56.5	2.03	19.72	2.09	16.1	1.1	2.42	0.83	0	0.74	0	0	0	397.1	393.57	459.29	4.66	26.66	53.4	0	103686.7	3.87
p1198	Admission	30.55	7.84	29	4.19	2.85	71.56	5.38	2.84	0.97	51.86	3.14	17.33	2.65	11.5	4.12	3.62	1.65	110	0.74	0	50	0	286.41	318.16	945.51	4.82	30.91	85.4	0	154563.5	4.23
p1198	Admission	39.27	6.33	29	5.5	1.76	46.79	5.25	3.68	1.04	189.54	4.38	17.58	10.9	35.59	2.2	7.32	2.48	880	34.15	91.89	1250	85.79	306.07	349.81	593.2	4.58	46.61	26	0	143521.6	6.12
p1198	D2	25.44	4.8	25.17	4.68	1.94	34.71	4.28	2.84	1.52	97.24	0.13	13.87	2.65	9.35	0.51	4.66	1.32	150	3.51	0	0	0	488.38	328.76	637.71	5.8	33.66	21.8	0	103358	7.2
p1198	D3	27.27	0	0	0	1.39	106.95	3.03	10.02	1.31	39.92	1.39	79.45	2.94	9.71	11.55	7.43	0.83	0	1.7	0	0	0	551.34	480.34	966.15	9.84	51.91	23.7	4.78	53952.13	13.5
p1198	D4	22.95	3.24	26.14	6.49	1.03	44.13	1.61	0.32	1.24	218.84	3.14	29.41	1.83	28.59	0	6.44	0.99	120	1.19	0	0	0	448.15	358.29	678.56	5.43	47.43	17.7	0	108263.6	10.32
p1198	D5	35.58	0	0	0	0.65	103.2	3.29	3.26	1.59	101.16	5.29	174.31	2.36	11.85	0.08	9.68	0.66	150	7.79	0	0	0	736.71	613.89	2601.78	13.68	67	20.7	42.13	78682.47	20.42
p1198	D6	22.95	4.8	29	11.04	0.84	56.01	1.75	1.58	1.38	48.85	3.65	11.98	7.96	5.7	0	5.22	3.14	590	26.59	0	620	60.6	651.42	211.49	680.12	1.79	39.85	18.8	35.57	59342.4	9.67
p1198	D7	25.67	0	0	0	0	96.94	0	0	0.77	34.11	6.15	117.66	2.09	13.99	0	5.45	0	0	2.27	0	0	0	407.93	311.55	1014.5	11.01	59.59	20	64.36	77540.36	14.65
p1196	Admission	56.72	7.65	23.21	3.86	0.18	15.16	0	0	1.11	11.5	2.6	21.05	0	0	0	1.4	0.91	0	0	0	0	0	321.09	157.02	512.25	0.19	40.93	61.3	0	10199.66	3.36
p1196	Admission	29.9	11.91	36.27	5.83	1.58	23.69	0	0	1.59	3.98	4.14	3.55	0	0.31	0	1.66	1.49	0	0	0	0	0	182.68	72.69	430.73	2.69	37.67	45.5	0	27980.31	1.61
p1196	Admission	34.88	4.8	21.19	3.36	0	0	0	0	1.11	14.78	2.03	5.62	43.9	0	0	0.85	0.01	1410	99.6	885	11100	141	192.55	170.73	285.89	0	41.35	28.2	0	62444.76	2.34
p1195	Admission	49.17	18.11	67.74	12.01	4.29	86.86	1.48	2.42	3.04	34.41	12.65	12.84	1.09	74.26	0	6	5.46	190	29.88	0	2510	0	208.16	644.31	833.57	3.67	52.31	72.9	11.59	14865.9	10.61
p1195	Admission	26.36	11.18	32.69	5.18	1.21	20.88	0	0.32	1.95	27.15	4.61	8.7	2.77	1.53	0	2.66	1.98	330	42.36	440	5050	32	175.61	537.89	881.63	0	29.98	59.7	0	50267.7	5.17
p1194	Admission	38.32	12.65	39.77	7.79	3.39	86.86	0.66	3.68	2.02	95.09	10.93	11.81	35.5	66.08	0.71	5	2.72	1410	93.13	1274	4360	175	137.12	167.67	146.81	5.73	31.38	26.1	0	32786.06	3.36
p1194	Admission	19.28	3.24	14.8	0.84	0	0	0	0	0.8	61.56	3.14	10.36	0	10.78	0	0	0	0	0.96	0	0	0	161.3	99.99	132.47	0	9.88	21.9	6.77	143724.8	0.88
p1194	Admission	17.39	6.33	21.19	2.69	0.65	13.7	0	1.16	1.59	72.15	5.94	10.9	28.87	4.78	0	3.73	0.99	880	57.71	707	2810	112	122.3	107.87	83.17	0.38	15.82	23.8	18.86	170381	2.19
p1194	D1	31.5	7.46	20.17	3.19	1.12	13.7	0	0	1.45	38.39	5.51	6.55	31.32	0.31	0	3.26	0.99	1060	71.43	982	3230	134	132.55	108.94	40.68	3.24	19.35	22.3	3.99	117483.8	2.56
p1194	D1	23.55	3.63	4.7	0	0	0	0	0	0.84	58.7	0.65	8.8	29.85	0.31	0	2.17	0.01	900	54.86	783	2350	109	169.36	153.36	78.67	0.55	15.26	19.6	0	63410.13	2.49
p1194	D2	25.9	3.24	12.51	0.15	0	0	0	0	1.31	23.18	4.14	5	29.6	2.31	0	2.66	0	970	61.25	892	3160	128	210.84	134.65	74.13	0	19.61	20.4	0	25311.71	3.5
p1194	D3	47.88	0.74	0	0	0	56.01	0	5.37	0.77	15.12	0	27.33	0	0	0	1.92	0	0	1.72	0	0	0	340.66	274.46	305.7	5.13	28.1	19.6	18.46	17871.97	10.46
p1194	D4	28.27	0	0	0	0	101.95	0	0.74	0.77	61.01	2.03	67.71	0	3.46	0	3.38	0.01	0	2.89	0	0	0	511.45	316.23	490.85	7.43	26.42	22.7	10.38	9013.483	10.32
p1194	D5	17.11	0	0	0.84	0.09	104.45	0	3.05	0.64	61.42	1.04	84.62	0	3.08	0	5	0	0	4.17	0	0	0	632.64	242.07	467.63	5.05	22.71	21.5	23.33	32463.1	8.87
p1194	D6	18.21	0.27	0	4.19	0	197.02	0	0.74	1	41.08	3.65	58.38	0	2.7	0	3.85	0	0	0	0	0	0	1149.33	183.49	835.08	2.6	24.21	22.7	80.9	21701.42	10.17
p1191	Admission	37.16	2.84	0	12.49	0	3	0	0	0.64	129.52	12.31	54.44	0	178.55	0	0.56	3.63	0	2.09	0	0	0	454.35	730.44	3473.21	9.34	77.64	45.8	0	13907.61	6.26
p1191	Admission	48.92	2.44	0	27.81	0	16.6	0	0	0.7	68.16	9.85	73.29	0	27.9	0	2.17	2.72	0	1.15	0	0	0	289.22	493.53	1428.95	2.6	33.21	28	9.98	290484.3	2.49
p1191	D1	22.95	4.02	0	16.51	0	0	0	0	0.64	99.64	8.74	36.65	0	19.43	0	1.66	4.3	0	0.36	0	0	0	292.92	314.87	881.63	2.4	25.2	20.7	22.51	330479.7	1.9
p1191	D2	24.97	3.24	4.7	8.44	4.11	6.19	0.05	0	0.61	78.93	7.58	29.04	0	15.75	0	3.73	3.8	750	1.88	0	0	0	300.06	335.12	469.3	4.01	30.45	21.5	22.51	146633.5	3.14
p1191	D3	33.87	3.63	0	45.29	10.94	0	0	0	0.9	92.1	9.12	61.66	0	7.35	0	4.77	12.13	0	0.36	0	0	0	362.96	297.03	435.79	7.97	31.84	19.2	3.99	65748.16	2.05
p1191	D4	28.27	0	0	0.5	0	86.86	0	1.37	0.7	41.94	6.36	277.85	0	11.14	0	5.11	1.65	0	0.2	0	0	0	497.26	425.89	615.52	2.2	38.55	21.9	43.77	44510.54	4.52
p1191	D5	27.27	0.74	0	2.03	0.09	61.23	0	0	0.64	54.22	8.36	162.65	0	7.54	0	2.78	1.16	0	0.58	0	0	0	384.73	437.79	720.54	4.01	29.28	18.8	22.11	46047.7	3.87
p1191	D6	31.71	4.8	1.24	33.81	3.12	0	0.8	0	1.04	34.41	4.14	16.7	0	1.53	0	2.17	4.55	220	3.25	0	0	0	419.58	211.57	344.7	2.4	27.14	10	0	51849.89	3.07
p1186	Admission	69.89	5.95	23.21	3.53	2.85	45.46	3.29	5.37	1.38	33.07	2.03	6.16	29.85	10.07	1.66	3.85	0.83	1120	69.33	1023	2160	154	192.8	195.01	884.62	4.82	52.71	75.1	0	16822.33	3.65
p1186	Admission	26.59	13.75	53.04	7.14	5.19	85.59	4.77	5.16	1.95	29.76	8.74	7.26	77.59	7.54	4.29	6.99	4.3	1930	131	1600	6380	257	149.4	129.81	554.6	5.73	35.91	48.1	0	81173.24	3.36
p1186	Admission	21.84	10.62	44.86	4.52	3.57	56.01	2.27	4.53	1.88	19.25	7.58	5.91	5.99	7.54	3.09	4.54	2.64	0	1.2	0	0	0	113.22	77.28	264	3.67	31.38	25.7	0	200139.9	1.61
p1186	D1	81.83	1.61	0	0	1.03	50.75	1.35	0.95	0.64	53.74	0.65	37.18	28.37	8.81	0.08	3.62	0.66	1040	89.03	1217	2320	167	164.76	392.97	580.38	3.67	33.66	23	0	194465	3.94
p1186	D2	54.94	1.19	0	0	1.76	91.91	0.37	15.11	0.7	121.85	2.88	73.62	5.21	75.43	0	9.36	2.15	0	1.72	0	0	0	309.06	719.91	1208.47	10.52	61.89	29.5	51.99	90527.71	10.17
p1186	D3	30.97	2.84	12.51	0.5	1.58	38.77	0.8	1.16	1.11	51.23	0.65	14.7	45.06	4.96	0	6.11	0.99	1640	102	1576	3850	214	282.46	214.34	504.04	2.6	29.28	23	14.41	93434.4	5.39
p1186	D4	41.15	1.61	10.11	0.5	0.75	18.04	0.94	0.74	0.97	12.9	2.03	8.73	12.04	0.09	0	5.22	0.34	580	23.35	514	710	67.7	337.4	177.98	330.61	2.78	25.93	20	3.59	65751.63	3.94
p1186	D5	148.7	7.84	21.19	3.19	3.03	48.11	1.75	2.42	1.11	11.24	7.18	16.49	30.34	1.93	0	5.67	1.65	1430	80.1	1355	2280	165	386.01	517.47	1088.47	6.67	51.3	26.9	8.37	33062.89	10.97
p1179	Admission	45.24	14.11	67.74	9.26	3.57	58.62	2.01	5.37	1.95	39.99	10.57	15.3	15.58	15.4	0.08	6.33	3.63	860	67.59	711	7110	76.34	152.95	347.78	484.24	6.46	52.91	44.7	12.8	68784.6	9.81
p1179	Admission	35.88	14.48	53.04	9.58	3.93	58.62	1.75	3.26	2.02	30.97	10.22	13.08	20.85	19.77	2.38	5.89	3.97	440	59.06	440	5530	39.22	124.38	194.47	637.71	5.13	61.32	37.1	8.37	228012.2	7.78
p1179	Admission	48.74	32.2	113.7	21.62	10.5	156.09	8.11	12.99	3.75	34.11	24.34	15.16	0	25.15	8.39	12.61	12.29	0	0	0	0	0	154.17	204.26	675.43	8.89	79.43	37.1	33.12	239031.8	13.28
p1179	D1	41.24	11.55	48.18	7.63	3.3	45.46	1.21	5.16	1.84	54.57	7.18	14.6	12.84	6.62	0.91	5	2.97	230	33.19	0	3310	0	195.8	269.58	273.15	4.89	56.47	24.6	23.33	181610.2	7.86
p1179	D2	56.56	12.65	44.02	6.65	2.13	45.46	1.21	1.16	1.49	33.22	3.14	8.96	0	0	0	7.21	2.97	0	2.1	0	0	0	293.14	291.85	746.76	2.2	37.23	20.7	22.51	99212.3	9.3
p1179	D3	89.66	10.81	44.86	7.3	1.49	42.79	1.08	1.16	1.66	30.82	4.14	17.19	0	0	0	6.99	1.98	0	1.15	0	0	0	395.82	541.03	809.37	4.26	51.5	22.3	28.62	79128.8	10.17
p1179	D4	51.04	9.33	44.86	7.14	1.94	29.24	0.05	2.21	1.66	11.15	4.14	7.65	0	0	0	6.55	2.48	120	14.55	0	1480	0	400.71	234.72	648.75	2.2	44.11	23.4	7.97	58181.3	8.65
p1178	Admission	79.01	5.57	12.51	3.86	2.4	1357.98	2.4	5.79	0.84	275.65	4.14	178.67	0	170.9	2.56	6.33	3.47	40	1.15	0	0	0	4114.3	4022.8	3779.56	35.69	1054.97	31.4	20.89	50514.98	28.63
p1178	Admission	60.05	4.22	22.2	0.84	0.84	29.24	1.48	0	1.35	202.89	2.03	60.74	3.34	51.66	0	5	1.49	990	9.98	1037.73	1580	107.01	1261.94	457.76	1117.24	5.88	75.11	23.8	27.4	271924.5	6.48
p1178	Admission	53.79	7.27	34.49	2.86	1.94	84.32	1.75	2	2.31	168.98	7.97	67.47	0	77.76	3.95	6.99	2.48	170	1.88	0	0	0	1587.71	415.44	2317.06	7.77	135.48	29.1	42.95	180989.4	10.53
p1178	D1	42.9	5.57	14.8	1.86	0.84	23.69	0.94	0	1.31	127.46	2.6	34.75	4.48	9.89	11.08	5.45	0.99	2170	15.05	1266.13	1870	156.87	606.6	360.32	1511.12	4.97	60.93	23.4	48.7	158242.5	7.28
p1178	D5	58.64	5.18	23.21	0.15	0	37.42	0	0	2.09	16.29	2.6	12.12	0	1.14	0	2.42	0.58	890	0.58	563.95	0	47.57	774.48	250.27	445.89	2	35.68	12.3	44.59	19396.97	5.17
p1178	D6	78.31	2.84	0	0	0.09	58.62	0	14.48	2.02	14.1	10.22	19.22	0	1.14	0	2.66	0.99	50	0.58	0	0	0	568.3	366.35	2203.82	6.74	59.78	16.1	108.31	14555.25	17.54
p1177	Admission	55.59	4.22	13.67	1.86	0	85.59	0	0	1.11	93.56	4.61	8.3	0	42.21	0	0.56	1.07	0	4.17	0	0	0	225.09	329.06	827.53	7.57	44.95	32.9	0	15611.21	4.45
p1177	Admission	35.18	7.84	30.86	4.52	1.03	20.88	0	2.42	1.59	31.34	9.12	7.13	0	0.31	0	2.9	2.15	0	1.72	0	0	0	151.18	108.55	374.32	4.42	28.81	25.7	0	53871.1	1.98
p1177	Admission	22.95	2.44	0	0	0	0	0	0	0.64	28.23	0	4.47	35.01	0	0	0	0.01	1450	89.72	1458	3900	215	183.45	128.2	328.85	0	24.95	19.2	0	67977.78	0.51
p1177	D1	24.15	5.95	36.27	5.5	0.28	3	0.66	0	1.24	66.02	6.57	8.8	0	45.59	0	3.38	1.82	0	1.2	0	0	0	227.12	102.77	271.33	4.58	26.17	17.7	0	39268.44	1.47
p1177	D3	14.81	0.27	0	0	0	0	0	0	1.17	16.63	0	3.49	0	0	0	0	0.5	370	17.32	128	430	36.03	412.49	137.59	645.6	0	21.18	22.3	0	11043.55	1.9
p1177	D4	22.34	10.07	44.86	7.47	3.03	49.43	0.37	5.37	2.45	21.35	11.45	6.84	106	7.17	0.31	6.33	4.3	2550	194	1871	8940	310	202.25	96.27	460.96	4.89	31.15	22.7	0	3972.912	4.81
p1174	Admission	63.2	126.54	74.42	2.14	0	0	0	3	0	12.2	0	8.27	18.45	2.85	0	0	0	500	64.76	510	7170	50.21	247.87	349.55	829.11	9.97	32.24	38.7	0	14283.7	9.48
p1174	Admission	112.91	218.55	93.51	5.04	4.35	6.45	0.19	8.57	0	13.2	0	12.35	82.9	4.21	0	0	0	3370	227	1602	21080	281	213.11	492.71	956.54	11.38	44.84	45.1	0	15343	13.14
p1174	Admission	51.64	96.11	45.94	0.97	0	0	0	0	0	19.24	0	7.83	3.09	0	0	0	0	140	29.88	0	2060	0	326.87	314.59	1701.74	10.4	21.41	42.1	0	22825	9.72
p1174	D1	60.39	154.65	94.89	3.5	0.84	7.95	0	6.41	0	7.45	0	2.84	36.72	0	0	0	0	610	90.92	558	7550	60.7	228.41	143.56	366.36	7.75	31.55	33.3	0	37385.4	7.32
p1174	D1	56.66	46.33	53.85	0.27	0	0	0	0	0	6.26	0	3.91	9.29	0	0	0	0	380	37.79	0	4120	23.63	269.37	351.74	1245.36	7.51	14.4	40.4	0	33703.9	7.18
p1174	D2	85.74	120.67	74.42	2.92	0	0	0	1.11	0	5.57	0	6.44	52.25	0	0	0	0	2080	157	1198	15850	189	390.87	351.33	1422.63	10.52	29.41	45.1	0	23738	11.95
p1170	Admission	59.74	138.02	77.52	6.77	0	0	0	9.62	0	72.59	0	13.39	0	3.19	0	0	1.76	610	8.62	0	0	25.17	289.38	191.99	1202.62	0	32.92	53.7	11.08	21577.44	11.67
p1170	Admission	32.19	28.71	41.69	9.07	0	0	0	0	0	23.28	0	13.22	0	0	0	0	1.02	0	1.15	0	0	0	227.31	151.02	1043.35	0	17.62	49.9	0	36616.35	11
p1170	Admission	36.42	28.71	53.85	10.98	0	0	0	0	0	42.64	0	16.68	0	0	0	0	3.1	0	0.84	0	0	0	214.79	206.04	1193.87	1.55	24.8	44.4	0	38092.09	11.86
p1170	D1	68.36	208.79	138.72	6.58	4.79	16.94	2.51	14.21	0	30.02	0	14.14	13.23	4.12	2.25	0	2.07	2090	45.39	817.06	5210	137.11	337.87	253.69	917.59	10.4	44.84	38.7	18.32	31676.24	14.32
p1170	D3	25.55	0	45.94	9.65	0	0	0	0	0	22.17	0	14.34	0	0.87	0	0	2.67	0	1.5	0	0	0	305.83	183.03	738.82	5.78	20.5	27.6	0	41691.65	10.57
p1170	D4	54.62	18.39	41.69	3.3	0	0	0	0	0	21.43	0	29.84	0	0.68	0	0	0	20	10.68	0	0	0	310.67	253.92	662.79	5.78	11.31	59.9	0	17223.51	10.15
p1170	D5	23.73	46.33	45.94	8.31	0	0	0	0	0	17.3	0	18.73	0	0	0	0	0.57	0	1.15	0	0	0	307.24	220.78	790.37	6.1	22.29	45.5	39.28	32756.38	13.99
p1167	Admission	37.56	83.01	71.23	0	0	28.91	0	0	0	44.89	0	20.09	0.67	12.42	0	0	0	550	5.45	798.49	2100	121.65	167.09	435.34	1842.01	0	31.55	56.8	23.9	31228.47	5.54
p1167	Admission	30.28	191.34	105.5	2.72	3.42	4.96	1.61	8.57	0	52.16	0	21.82	2.96	36.15	0	0	0	180	8.62	224.29	860	26.18	131.3	336.65	1357.51	0	48.38	63.7	24.98	66423.65	7.95
p1167	Admission	19.63	69.16	71.23	0.47	0	0	0	0	0	61.18	0	16.06	0	5.7	0	0	0	290	1.5	0	0	26.64	165.11	273.44	1066.19	0	30.13	40	0	29824.43	6.84
p1166	Admission	49.73	61.88	75.98	0.57	11.06	129.11	0	3.59	0	29.26	56.51	10.04	8.67	14.61	16.68	18.71	0	730	30.55	1089	2700	263	325.37	124.98	1461.34	10.52	33.26	39.7	0	44654	8
p1166	Admission	32.61	138.02	136.51	3.11	10.34	100.7	0.19	11.43	0	35.1	44.35	7.77	45.81	8.06	18.54	12.49	2.15	1640	121	1343	11600	279	224.23	101.68	1146.86	9.44	46.76	141.1	8.44	100960.4	9.24
p1166	Admission	29.74	108.62	133.15	2.14	6.49	69.3	0	9.09	0	43.35	31.19	9.04	33.08	10.21	14.13	4.82	0	1410	98.61	1226	9650	255	205.88	151.21	1014.02	9.73	45.12	40	0	195115	8.14
p1166	D1	46.55	18.39	80.56	0	4.9	85.75	0	0	0	165.94	44.8	15.28	4.72	10.21	11.39	23.19	0	480	14.88	972	1040	219	1193.75	338.11	1873.54	12.63	33.59	29.9	17.17	141272.5	11.19
p1165	Admission	60.31	244.65	97.6	3.89	0	31.9	0	11.17	0	107.18	0	23.39	45.08	300.34	6.62	0	0	8460	138	2580.73	20780	464.25	116.97	305.79	172.74	5.97	49.17	28.2	6.34	2571.749	7.8
p1165	Admission	50.26	235.27	89.31	3.79	0	13.94	2.34	12.19	0	66.18	0	31.29	10.4	124.88	5.33	0	0	5400	56.28	1914.51	8870	247.85	426.8	529.59	573.15	16.52	69.56	27.2	37.31	21803.88	12.33
p1165	Admission	58.44	201.38	74.42	3.69	0	3.46	0	6.41	0	121.59	0	44.28	26.7	37.3	5.99	0	0	2320	90.32	1895	7680	227.23	563.16	771.93	965.21	16.05	76.16	26.2	24.45	31246	18.08
p1165	D1	50.95	173.33	77.52	15.18	0	0	0	4.17	0	95.58	0	34.91	39.63	83.69	3.4	2.29	0	3270	126	1847.43	8760	243.41	680.14	629.32	3893.46	10.04	50.48	27.6	26.06	26692.78	11.62
p1165	D1	41.4	186.25	64.6	5.14	0	14.69	0	2.08	0	120.19	0	34.96	10.4	49.62	8.94	8.77	0	2580	51.36	1637.85	7810	175.74	876.25	794.75	3472.11	10.98	54.52	37.3	31.28	23177.17	17.94
p1165	D2	17.5	175.94	71.23	1.55	0	0	0	3	0	27.77	0	13.31	11.68	20.11	0	0	0	2550	51.01	1479.97	6470	168.42	389.52	199.49	546.54	4.94	23.14	33.6	0	39766.48	6.31
p1165	D3	59.99	249.3	94.89	42.09	0	22.92	0	8.04	0	205.59	0	34.61	72.61	16.63	0	10.51	0	8700	198	2764.49	20130	487.89	938.53	1030.12	1864.53	12.67	46.22	81.2	22.26	44844.6	11.43
p1165	D4	29.96	249.3	97.6	45	1.46	25.92	0	6.96	0	31.86	2.59	31.37	23.21	23.18	5.33	15.71	0	7620	97.07	2750.49	14010	419.11	1516.88	370	6279.4	7.43	41.7	24.6	57.06	35982.31	14.32
p1165	D5	21.16	220.96	57.56	6.01	3.54	219.55	0	0	0	39.88	14.47	319.53	0	15.47	0.76	16.4	0	370	36.07	1274.33	3460	25.17	1275.03	778.86	4760.19	8.21	50.99	32.9	81.78	59816.48	11.95
p1165	D6	35.97	285.46	115.54	6.97	2.43	22.92	0	10.14	0	14.65	8.57	25.16	69.79	9.58	6.62	11.56	0	7170	165	2423.2	13640	339.98	1698.39	189.39	1284.88	8.33	52.02	32.2	60.65	65407.29	15.02
p1163	Admission	67.74	79.62	77.52	2.04	0	50.6	0	1.77	0	49.29	18.98	20.39	0.5	167.16	0	9.65	0	1410	15.87	776.5	170	62.11	163.11	847.5	590.2	19.09	60.03	54.7	0	16748.57	11.67
p1163	Admission	18.97	61.88	49.99	0.37	0	15.44	0	0	0	74.29	0	22.78	3.45	6.93	0	4.57	0	760	8.78	0	0	23.34	147.49	499.82	158.06	5.23	20.5	31.2	0	334769	4.57
p1163	D1	38.53	132.32	94.89	2.53	0.67	30.41	0	4.17	0	40.91	0	17.12	0	11.16	0	3.67	0	0	2.94	0	0	0	157.56	536.8	93.08	11.08	41.11	25.6	0	246988.2	8.48
p1163	D2	25.67	54.3	66.3	0.27	0	18.43	0	0	0	228.89	0	71.12	0	62.05	0	4.57	0	0	2.94	0	0	0	260.36	580.36	243.1	10.4	20.96	20.6	0	108071.7	5.44
p1163	D6	21.41	18.39	83.54	0.17	0	25.17	0	0	0	106.87	2.59	42.11	0	39.43	0	16.3	0.76	0	1.9	0	0	0	751.31	545.08	567.82	13.48	27.55	16.7	95.38	153656.4	9.57
p1162	Admission	106	105.54	111.84	1.94	0	0	0	1.77	0	106.62	0	18.67	32.21	14.45	0	0	17.44	1470	106	1013	11630	154	296.52	408.61	1777.94	0	31.55	49.9	0	11332	9.05
p1162	Admission	58.6	92.89	77.52	1.94	0	0	0	0	0	87.51	0	19	15.89	7.25	0	0	8.02	470	55.24	0	4650	25.41	314.78	291.92	470.06	0	29.41	40.7	0	25065.3	6.84
p1162	D1	65.01	260.78	155.77	7.73	8.27	22.92	4.51	17.41	0	66.9	0	11.35	45.24	5.21	6.62	0	7.04	1740	127	1061	13230	156	171.03	277.17	508.27	1.91	57.67	27.6	85.53	137464.3	13.28
p1160	Admission	75.55	465.16	161.89	36.92	4.46	32.65	3.63	25.28	0	150.26	21.35	27.93	6.87	143.69	17.26	10.93	9.68	1280	14.56	779	6560	146	107.11	758.1	1320.06	17.93	168.81	49.6	0	1724	12.85
p1160	Admission	49.03	332.81	140.92	16.7	5.87	78.27	1.99	22.46	0	62.52	21.35	42.94	7.86	38.51	16.82	11.98	7.04	1200	16.01	769	6310	124	121.8	594.81	747.41	10.42	99.02	35.6	46.47	133904.7	9.76
p1160	Admission	52.54	239.98	145.25	16.7	5.87	43.87	3.63	21.98	0	50.8	21.35	37.96	18.79	33.49	14.44	9.65	7.41	1520	33.49	946	8590	145	112.18	512.06	635	11.73	83.94	36.3	52.51	210694.6	8
p1160	Admission	38.73	260.78	115.54	10.03	5.23	30.41	0.19	9.09	0	39.68	0	36.02	23.77	23.72	13.35	4.7	4.54	960	33.86	557	4670	102	128.22	378.62	495.53	7.51	52.27	25.9	0	173128	7.32
p1160	D2	36.12	216.12	113.08	13.56	0	22.92	0	7.5	0	30.26	7.01	19.22	5.76	11	9.67	6.97	6.48	550	5.51	240	3190	56.6	281.17	283.47	883.02	8.79	51.25	22.6	0	63113.31	8.57
p1160	D3	39.02	253.91	134.27	16.7	1.16	30.41	0.19	13.2	0	35.21	8.57	21.15	0	11.08	11.73	10.4	9.45	710	1.93	369	3810	76.81	338.37	343.49	1237.68	9.64	80.5	25.9	2.4	27850.54	8.33
p1158	Admission	29.2	225.77	20.98	6.39	0	24.42	0	3.88	0	69.97	0	32.06	7.56	2067.04	0	0	1.36	1460	52.13	434	1810	136	118.74	773.02	247.3	8.46	49.96	32.2	0	24855.64	7.37
p1158	Admission	30.71	151.92	13.75	5.62	0	22.18	0.5	0	0	226.34	0	93.6	1.15	21.42	0	0	1.11	190	2.87	0	190	1.76	140.32	896.11	175.89	6.88	49.43	31.9	0	140145.9	6.7
p1158	Admission	38.68	140.83	20.98	3.21	0	17.69	0	0	0	346.47	0	97.52	5.27	262.67	0	0	0	690	21.57	78.47	1090	69.67	132.41	617.08	144.43	5.65	30.13	27.6	28.17	153412.9	6.22
p1158	D1	30.07	285.46	64.6	7.93	4.35	15.44	3.32	11.68	0	397.25	0	76.43	80.75	24.41	2.25	0	1.6	2660	163	973	8190	297	154.24	343.42	89.94	9.62	48.91	31.6	99.02	155208.3	8.33
p1158	D2	26.81	175.94	13.75	3.89	0	0	0	0	0	512.99	0	45.67	4.92	13.05	0	3	0	630	23.66	36.16	1060	69.67	304.82	455.37	193.73	7.54	23.14	26.2	88.84	133048.7	7.18
p1158	D3	27.03	140.83	0	4.56	0	0	0	0	0	499.41	0	49.62	6.78	15.7	0	5.92	0.47	740	41.7	190	1450	88.99	333.81	624.93	224.18	7.75	28.67	29.2	78.84	112891.1	9.38
p1158	D4	25.61	138.02	2.23	3.89	0	0	0	0	0	175.1	0	28.6	26.83	16.09	0	0.29	0.37	2330	112	759	4080	252	319.09	385.76	305.19	6.35	26.4	21.6	105.03	82462.82	8.67
p1158	D5	27.65	105.54	2.23	3.3	0	4.96	0	3	0	65.38	0	21.04	6.78	6.85	0	1.85	0	1010	50.05	406	1480	103	320.79	273.03	389.6	6.53	31.89	22.2	66.83	71602.21	9.19
p1158	D6	35.92	211.25	26.99	6.97	0	10.95	0	1.45	0	102.47	0	41.67	9.2	22.49	0	6.74	4.21	1160	61.68	522	2080	127	353.33	356.8	390.66	8.79	42.28	23.9	107.81	63683.28	10.81
p1157	Admission	72.93	37.87	0	1.94	6.79	105.19	0	0	0	111.47	51.61	19.85	27.27	236.36	17.11	24.65	0	410	37.86	1237	1150	81.02	243.58	752.37	305.19	10.48	60.72	35.3	0	2699.922	11.62
p1157	Admission	45.55	61.88	13.75	1.16	6.18	117.9	0	0	0.65	310.85	47.43	72.28	46.16	306.98	17.54	18.71	0	650	77.06	1519	2310	115	198.9	1157.82	196.88	11.22	40.52	27.2	0	29134.19	10.15
p1157	Admission	59.82	37.87	0	1.16	5.44	91.73	0	0	0	820.83	48.71	80.5	17.86	284.18	17.97	23.01	0	0	24.89	301	590	33.34	303.21	2140.46	305.19	16.39	73.73	25.9	0	64217.11	15.26
p1157	D1	35.52	132.32	64.6	1.94	3.42	79.77	0	3	1.02	260.06	37.42	39.4	19.66	272.62	16.09	6.27	0	360	25.98	903	640	45.6	199.71	342.49	179.04	10.77	36.53	26.9	22.26	121785.3	7.28
p1157	D1	26.47	37.87	20.98	0	1.89	88.74	0	0	2.92	243.84	40.63	30.25	17.25	305.06	15.5	15.22	0	0	13.04	281	0	10.44	364.67	326.41	150.72	10.81	22.29	44.8	24.45	122737.5	9.81
p1157	D2	32.09	86.34	37.17	1.55	4.57	90.24	0	0	3	134.05	41.58	34.76	28.42	155.38	16.68	20.88	0	0	34.63	654	200	45.6	293.05	417.22	224.68	10.14	21.41	24.2	44.1	87410.41	8.71
p1157	D3	37.95	126.54	37.17	3.5	7.39	108.18	0	3.59	3	85.77	47.22	24.66	41.28	115.2	18.4	19.75	0	370	63.26	1397	2110	81.6	353.33	491.5	551.86	9.86	29.77	28.6	18.9	50679.54	10.53
p1157	D4	53.52	96.11	13.75	26.07	5.87	141.07	0	0	3.92	115.12	52.82	53.57	24.38	155.62	22.33	52.29	0	310	31.03	450	130	2.4	1344.28	551.65	2272.49	11.45	27.17	27.9	33.32	38057.5	15.68
p1157	D5	81.55	54.3	20.98	35.6	6.49	152.28	0	0	4.07	64.28	50.59	47.68	29.85	192.93	20.19	37.74	0	510	34.99	1126	1730	85.65	2096.18	664.53	4638.9	11.38	36.53	30.2	59.76	46248.4	21.45
p1157	D6	138.21	0	0	4.46	7.89	130.61	0	0	6.11	87.88	64.66	48.18	17.86	217.92	23.11	39.75	0	0	13.04	0	0	0	1257	1381.92	6083.09	14.99	47.58	45.5	73.74	39876.35	27.6
p1156	Admission	39.4	65.55	77.52	0.57	0	0.46	0	1.11	0	34.22	0	5.66	32.26	26.4	0	0	0	1450	74.05	391	3400	152	126.24	265.04	432.98	6.16	36.53	32.6	0	31834.75	3.01
p1156	Admission	51.21	289.87	161.89	7.54	9.97	40.88	5.61	18.63	0	66.14	17.28	16.68	94.98	18.57	9.49	2.15	0.15	1900	163	843	7070	243	71.18	302.05	115.08	9.75	68.71	31.6	39.76	47120.53	11.9
p1156	Admission	37.56	143.63	94.89	2.53	0	0	0	6.96	0	63.25	0	14.82	12.65	16.09	0	0	0	990	50.88	175	930	72.26	51.18	242.7	264.13	8.06	56.96	25.2	38.79	66835.68	9
p1156	D1	45.55	126.54	72.83	2.14	0	0	0	4.74	0	69.4	0	14.28	34.25	13.05	0	0	0	1860	82.26	490	3470	181	54.18	297.66	193.73	7.8	47.31	23.6	32.81	77064.12	9.1
p1156	D1	48.94	83.01	97.6	1.16	0	0	0	0.41	0	77.79	0	9.68	26.34	10.84	0	0	0	880	65.81	283	2130	100	168.57	203.16	145.48	7.35	30.84	21.6	0	41595.39	7.18
p1156	D2	42.34	96.11	102.91	2.24	0	0	0	2.08	0	52.94	0	12.2	44.78	11.48	0	0	0	1440	90.44	460	3700	173	233.21	190.83	160.16	9.4	34.26	24.6	0	24872.49	7.13
p1155	Admission	74.95	96.11	77.52	1.36	0	108.18	0	0	0	26.12	12.35	6.76	0	153.94	0	0	0	210	7.36	574	0	112	154.62	1486.97	1411.58	7.54	38.71	37	0	11998.8	7.71
p1155	Admission	95.8	140.83	77.52	2.63	0	780.85	0	0.41	4.36	1436.67	13.44	269.71	0	70.54	0	3.27	0	240	23.9	524	1480	67.25	290.2	2248.36	2379.33	30.52	71.66	33.9	0	61203.8	21.73
p1155	Admission	130.38	83.01	102.91	0.97	0.48	112.66	0	0	9.3	192.2908	0	263.01	0	47.19	0	3.8	0	370	22.23	698	2000	124	332.23	4998.85	1775.69	15.05	49.17	31.6	0	259462.8	21.17
p1155	D1	37.16	69.16	71.23	1.16	0	13.94	0	1.77	1.28	346.98	0	79.73	0	88.4	0	6.03	0	150	8.32	489	0	53.82	158.95	2061.91	941.39	7.19	30.84	28.2	61.99	393035.2	9.34
p1155	D3	43.31	28.71	100.27	0.37	0	21.43	0	0	0	50.8	0	22.73	0	8.94	0	3.8	0	190	11.24	495	500	69.3	210.64	1451.59	568.89	9.66	26.4	25.2	0	98663.7	11.38
p1155	D4	94.53	96.11	67.96	0.97	0	69.3	0	0	2.04	32.99	0	25.53	0	4.71	0	0.03	0	200	15.22	502	730	66.42	216.47	974.45	665.99	12.56	29.41	23.6	0	75786.1	9.57
p1155	D5	95.45	69.16	90.73	0.97	0	108.93	0	0	2.2	30.72	2.59	25.79	0	9.1	0	1.85	0	270	13.22	682	550	92.21	227.09	1318.05	912.19	16.55	31.2	24.9	0	61949.7	13.61
p1155	D6	83.06	72.7	79.05	0.97	0	115.65	0	0	1.02	25.38	0	18.45	0	8.3	0	0.5	0	100	5.5	0	0	41.93	259.73	1827.8	1536.76	17.64	42.86	27.6	0	72465.2	18.18
p1151	Admission	49.4	15.91	15.22	1.49	0.49	94.2	1.17	0	5.46	18.2	0	14.44	0	33.77	0	1.46	1.32	2480	1.5	1295.68	4390	162.27	494.46	462.97	1900.51	6.13	12.49	34.2	0	20453.8	6.62
p1151	Admission	35.97	7.68	13.48	2.42	0.49	37.62	1.46	0	5.34	8.24	0	15.88	0	30.96	0	2.33	1.61	3100	2.31	1408.03	5360	190.48	403.04	241.38	1486.7	6.13	8.22	26.6	0	75670.33	6.44
p1151	Admission	27.07	10.07	22.1	4.3	1.81	32.75	1.9	0	5.98	3.65	0	9.83	0	15.68	0	3.39	1.56	3290	3.77	1812.99	7400	262.58	267.72	132.56	1246.78	6.78	7.98	30.2	0	117173.6	7.95
p1151	D1	47.97	1.81	21.53	0.58	0	8.2	1.03	0	5.11	0	0	6.47	0	2.77	0	4.44	1.41	1540	1.5	1732.8	5240	202.87	252.22	208.23	585.79	4.79	14.22	30.6	0	159224.1	6.62
p1151	D2	62.38	8.74	12.3	1.49	0	0.33	1.31	0	5.11	15.98	0	12.16	0	2.77	0	1.64	1.32	360	2.31	1030.89	4050	70.82	658.44	411.27	1051.51	3.96	18.83	27.7	0	64737.67	8.56
p1151	D3	37.82	7.42	3.82	0	0	2.74	0.61	0	5.23	3.91	0	8.82	0	0	0	0.6	1.22	390	0.58	1069.21	3490	69.26	669.02	321.17	1180.4	3.96	17.38	20.5	0	25639.97	7.86
p1151	D4	46.01	9.8	10.53	1.49	0	11.08	0.89	0	5.11	0	0	8	0	1.53	0	0.6	1.41	460	0.84	960.62	2920	70.82	530.31	285.29	1197	4.52	12.27	19.1	0	23599.51	9.43
p1151	D5	41.25	5.29	15.22	0	0	5.4	1.1	0	5	0	0	9.51	0	0	0	0.26	1.32	460	0.84	1084.71	3630	82.68	541.38	265.23	1480.51	4.24	10.05	37.4	0	16465.14	8.13
p1150	Admission	39.51	3.69	34.36	0	0	0.33	1.98	0	5	9.38	0	13.61	0	183.99	0	0.09	1.22	0	17.22	0	730	0	341.84	475.84	982.74	7.16	30.09	21.6	0	18708.8	9
p1150	Admission	36.25	23.91	53.87	7.61	2.57	85.35	3.28	3.93	6.28	110.41	4.71	51.82	12.99	74.85	2.13	4.44	1.61	370	59.38	473	5170	30.88	195.49	716.19	1026.52	11.06	35.01	26.6	0	54426.6	12.68
p1150	Admission	34.59	10.07	33.26	1.26	0	23.21	2.2	0	5.23	193.53	0	41.98	13.15	34.33	0	0.26	1.41	380	56.52	0	4710	18.12	153.43	648.92	934.72	7.16	23.47	23.4	0	154943	9.09
p1150	D1	47.19	24.44	56.54	8.08	3.33	249.85	2.51	3.15	6.81	269.36	3.27	89.99	22.49	303.89	1.85	4.09	2.27	520	76.67	513	5810	43.1	214.02	1592.42	617.53	11.53	42.92	22.3	20.26	220333.4	13.43
p1150	D1	41.52	9.01	90.62	0.58	0	14.03	1.9	0	6.75	129.6	0	43.63	1.43	21.38	0	3.74	1.27	270	43.66	0	2320	17.45	554.92	961.71	1032.77	7.04	28.17	21.3	175.79	147250.5	12.34
p1150	D2	38.02	5.82	76.06	0	0	2.74	1.31	0	7.22	37.52	0	15.57	0	6.38	0	0.6	1.22	110	28.56	0	1100	0	465.52	517.52	1228.12	5.74	22.67	13.3	73.54	51785.1	10.29
p1150	D3	43.38	9.01	67.66	0	0	9.63	1.31	0.14	6.45	13.82	0	28.43	0	16.82	0	0	3.12	0	17.22	0	330	0	664.79	556.89	1569.22	7.16	31.61	24.5	36.46	61821.7	18.99
p1149	Admission	65.13	4.76	106.05	0	0.11	32.75	1.61	0.14	5.34	58.12	0	23.66	0	946.25	0	1.98	1.22	1720	22.36	930.94	800	104.48	1182.03	637.96	8323.59	8.79	46.17	69.4	0	5928.954	14.69
p1149	Admission	49.33	14.84	80.76	1.96	0	148.79	1.17	0	5.46	160.57	1.25	124.97	0	364.38	0	1.29	1.12	360	8.2	146.48	50	0	1490.12	2241.63	6026.59	18.64	121.83	43.9	0	96413.99	20.96

p1149	Admission	45.81	8.74	77.1	2.42	2.76	62.75	3.44	1.61	5.69	349.83	1.25	109.13	0	420.18	0	5.15	1.51	1610	15.17	708.48	900	99.87	1377.69	1545.41	9198.72	12	54.5	61.5	0	166388.2	25.18
p1149	D1	29.89	9.01	71.87	0	0.86	29.54	1.61	0.62	5.69	132.25	0	46.46	0	91.39	0	4.8	1.22	1470	17.32	697.46	880	98.48	1049.31	573.49	5229.13	8.42	26.03	64.4	36.97	163473	14.86
p1149	D1	27.87	3.69	78.67	0	0	17.05	0.54	0	5.06	99.59	0	33.66	0	36.01	0	2.68	1.03	2670	17.32	1058.29	2030	181.95	827.22	588.58	5295.77	6.91	21.47	70.5	0	135078.7	12.51
p1149	D2	38.83	8.48	83.36	0	0	26.36	1.68	0	6.39	59.04	0	32.77	0	52.71	0	2.68	1.41	2240	10.94	1007.35	2290	169.3	1235.77	949.57	4973.52	7.16	25.05	44.3	23.99	69140.91	22.91
p1149	D3	38.02	9.54	67.66	1.03	0	23.21	1.17	0	6.04	57.21	0	29.62	0	56.04	0	2.68	1.22	1020	6.87	512.26	870	86.06	1334.28	715.96	9390.55	7.67	14.43	44.6	36.46	32070.85	16.77
p1149	D4	48.75	13.25	79.19	5.24	1.62	32.75	2.89	0	6.28	66.64	0.1	43.93	0	110.68	0.24	7.11	1.71	1730	25.98	801.24	1190	120.11	1457.23	851.48	10983.76	8.05	19.44	32.4	115.59	70895	17.35
p1149	D5	42.45	6.89	82.32	0	1.24	26.36	1.03	0	5.58	24.7	0	25.46	0	41.03	0	3.74	1.37	1430	13.04	663.35	780	98.01	967.03	527.29	4766.27	6.52	17.38	29.9	189.23	96264.5	16.19
p1149	D6	42.58	5.29	80.76	0	0.11	17.05	0.89	0	5.58	29.31	0	21.46	0	42.15	0	3.74	1.41	900	6.87	396.52	300	55.11	802.74	560.89	3117.27	7.42	19.44	18	139.09	82535.77	12.68
p1148	Admission	34.04	14.84	34.36	2.89	1.24	35.99	2.05	0.14	6.16	34.51	1.25	20.15	0	56.04	0	3.04	2.12	0	0	0	0	0	202.75	597.05	727.21	7.42	24.26	37.4	0	17232.36	9.09
p1148	Admission	28.09	8.21	29.94	1.96	1.24	29.54	1.17	0.86	5.58	32.95	0	12.99	4.09	9.9	0	1.64	1.71	3000	21.16	875.12	1390	141.77	150.27	400.37	424.08	6.91	21.47	27.7	0	46828.23	6.71
p1148	Admission	28.6	19.63	37.11	4.3	2.76	45.86	2.2	1.11	6.75	51.49	1.77	18.84	2.94	20.24	1.16	3.04	2.32	1550	15.64	0	0	66.71	145.11	467.58	492.37	6.91	21.47	25.9	0	44356.32	9.09
p1148	D1	22.89	6.35	32.71	0	0	0	0.61	0.14	4.77	92.54	0	21.51	0	57.7	0	2.33	1.61	0	0	0	0	0	171.22	491.55	456.13	4.52	21.07	23.1	101.58	55306	5.35
p1148	D2	25.37	4.76	43.64	0	0	2.74	0.61	0.62	4.89	53.16	0	10.68	0	17.39	0	1.98	1.91	880	16.04	0	0	0	244.04	546.01	532.77	4.24	20.26	32.8	152.31	20509.94	7.33
p1148	D3	40.18	8.48	56.54	0	0	12.55	1.03	0	5.06	22.25	0	12.89	0	13.38	0	1.98	2.01	0	0	0	0	0	524.93	1016.42	1727.81	5.47	33.88	25.9	250.15	9389.359	12.25
p1148	D4	32.79	7.42	35.46	0	0	17.05	0.75	0	5.23	9.04	0	13.3	0	8.74	0	1.29	1.61	0	0	0	0	0	598.68	789.17	1236.41	4.79	23.87	32.8	201.25	7332.479	11.92
p1148	D5	32.09	8.74	40.93	0	0	14.03	1.03	0	5.11	5.64	0	10.63	0	0	0	0	1.81	0	0	0	0	0	505.5	589.07	1476.38	3.67	19.85	21.6	167.7	6929.648	11.32
p1146	Admission	34.18	14.84	23.23	6.18	3.33	92.42	1.9	1.61	6.63	33.25	3.76	12.83	13.3	32.65	1.58	4.44	1.91	460	54.7	0	5000	47.8	279.61	324.37	760.85	11.3	16.13	26.3	0	41550.56	11.83
p1146	Admission	28.81	6.89	11.72	1.03	0	17.05	0.75	0	5.34	27.91	0.1	14.44	3.3	15.68	0	2.33	1.22	450	34.2	78.3	3900	44.5	329.71	441.64	869.9	10.83	10.27	21.3	0	110365.1	9.17
p1146	D1	28.23	3.96	8.14	0	0	17.05	0.41	0	5.23	170.09	0	27.04	0	42.71	0	3.39	0.84	0	4.2	0	1200	0	208.63	501.68	725.1	11.06	17.8	19.8	0	74255.25	9.78
p1146	D1	26.78	0.99	13.48	0	0	11.08	0.34	0.62	5.34	93.23	0	19.09	0	51.6	0	2.68	1.12	0	0	0	300	0	214.45	529.37	600.61	12	12.27	19.8	0	96122.09	9.78
p1146	D2	40.18	6.35	3.18	0	0	14.03	0.54	0	5.92	54.36	0	19.19	0	41.59	0	3.39	1.12	0	0	0	200	0	496.33	892.37	1740.16	11.53	14.43	29.2	0	55625.22	15.36
p1146	D3	46.53	5.03	9.34	0	0	17.05	0.82	0	7.22	92.19	0.1	26.85	0	28.15	0	2.68	1.12	290	9.6	0	3700	69.1	363.57	865.96	1939.53	10.36	14.43	27	34.41	69796.49	13.43
p1146	D4	38.36	8.48	12.89	0	0	11.08	0.89	0	6.75	24.49	0	14.8	0	14.53	0	3.04	1.41	0	0	0	0	0	436.02	498.21	1406.15	12.69	14.43	15.9	22.93	51097.05	14.52
p1146	D5	59.99	6.89	9.94	0	0	20.11	0.75	0	7.34	25.5	0	16.24	0	19.67	0	4.09	1.81	180	13.8	0	2500	37.2	585.8	584.3	1546.54	11.53	14.43	14.8	34.92	44404.56	15.69
p1144	Admission	42.18	7.15	25.48	0	0	14.03	1.03	0	5.69	68.53	0	24.51	11.01	23.64	0	1.98	1.51	800	39.99	490	1840	86.87	291.15	629.41	547.63	7.29	23.47	55	0	28528.46	7.95
p1144	Admission	47.45	9.8	23.23	0.12	0	97.76	0.89	0	5.63	64.3	0.1	36.16	0	21.38	0	1.46	1.27	180	10.28	0	0	24.13	327.83	1127.21	623.88	10.12	43.83	89.5	0	20754.38	14.19
p1144	Admission	34.18	7.42	36.56	0	0	11.08	1.17	0	5.81	49.35	0	20.05	6.23	43.82	0	0	1.46	460	28.77	164	1180	48.45	275.86	656.31	374.78	7.16	27.39	23.4	0	37777.16	8.91
p1144	D1	30.81	6.35	29.94	0	0	23.21	1.46	0	5.92	96.76	0.1	22.56	0	54.93	0	2.51	1.51	330	16.74	99.11	350	38.03	263.72	708.09	415.52	8.91	25.05	26.3	0	25215.15	7.68
p1144	D2	27.94	5.82	47.43	0	0	0	0.34	0	5.81	39.25	0	13.04	0	8.15	0	0.6	1.22	0	0	0	0	0	315.93	725.46	269.04	7.42	14.43	18.7	56.5	26190.19	6.08
p1144	D3	81.91	27.39	63.44	3.59	2	104.92	1.83	4.19	7.34	14.15	4.23	16.34	0	15.68	0.24	1.64	2.12	80	7.51	0	0	13.03	251.81	986.31	295.04	7.92	26.22	16.9	39.51	14136.16	12.09
p1144	D4	36.32	6.89	39.29	0	0	5.4	1.24	0	5.75	17.88	0	8.76	0	3.99	0	0.94	1.71	0	0	0	0	0	482	414.07	366.19	6.91	15.28	15.9	1.35	10354.95	7.42
p1144	D5	35.15	2.08	40.93	0	0	44.2	0.82	0.62	5.46	18.52	0.7	40.66	0	14.53	0	0	1.22	0	0	0	0	0	335.88	428.54	549.75	13.6	33.69	25.6	43.55	8868.356	11.66
p1144	D6	36.11	0	45.81	0	0	5.4	0.61	0	5.11	10.62	0	10.05	0	12.22	0	0	1.17	0	0	0	0	0	340.72	368.76	585.79	5.87	22.67	20.5	27.15	9874.617	7.33
p1142	Admission	94.63	5.29	23.79	0	0	0	0.54	0	5.11	76.45	2.03	17.26	0	51.6	0	0	1.32	0	1.5	0	0	0	110.52	342.16	813.33	9.15	35.19	57.2	0	11881.81	10.12
p1142	Admission	200.38	2.62	32.16	0	0	0	0.48	3.66	5.06	47.77	0	28.63	0	13.38	0	1.98	1.22	0	0.58	0	0	0	163.95	764.57	596.38	8.42	39.45	60.8	3.29	25079.07	10.89
p1142	Admission	148.16	4.76	12.89	0	0	0	0.75	3.15	4.89	63.94	0	27.44	0	48.27	0	0.6	1.27	0	1.15	0	0	0	377.74	876.12	617.53	8.91	57.64	56.1	10.93	13316.04	16.19
p1142	Admission	94.08	6.89	15.22	0	0	2.74	0.75	0	5.23	80.68	0	23.66	0	63.24	0	0.6	1.32	0	1.88	0	0	0	823.54	876.95	1155.48	7.67	49.92	36.7	0	37038.2	16.1
p1142	D2	79.15	2.62	35.46	0	0	8.2	0.48	0.14	5.69	63.58	0	14.13	0	109.02	0	2.68	1.22	0	2.31	0	0	0	647.85	1082.18	974.39	9.15	44.19	31.7	33.38	12467.69	18.5
p1142	D3	49.01	2.62	38.75	0	0	9.63	0.34	0.62	5	20.91	0	8.55	0	14.53	0	0.43	1.03	0	0.58	0	0	0	679.57	518.74	1028.6	10.59	29.32	32	71.13	20272.3	13.35
p1142	D4	50.11	2.89	38.75	0	0	1.49	0.48	0	4.77	9.72	0	7.19	0	0	0	0	1.12	0	0.84	0	0	0	448.39	398.25	1005.67	5.2	21.47	33.1	5.12	12214.71	10.12
p114	Admission	189.5905	0	58.4053	38.6742	0	8953.135	0	54.3096	2.7201	340.09	18.1831	182.6207	0	75.065	66.0812	21.3965	0	0	0	0	0	0	19.2353	1953.504	45.6716	129.7361	649.7129	n/a	1040.554	5880.42	3.0189
p114	Admission	36.4779	132.853	85.9251	222.8352	84.7584	2356.858	35.5694	117.7761	74.3065	79.4609	0	31.988	1.29	0.4387	66.33	98.1961	51.8499	0	7.19	0	0	0	33.0574	318.7501	202.629	104.9272	242.0394	14.023	1125.592	22650.44	9.5162
p114	D3	50.8114	102.0149	95.8867	155.0003	14.1729	4602.008	25.7917	106.3164	46.5079	141.6809	444.2246	45.3159	10.21	15.6754	72.3559	83.6061	38.196	140	14.9	0	200	14.93	29.6171	802.6716	187.1805	127.1808	372.1656	17.004	1106.793	41741.72	12.9778
p114	D7	33.6417	79.6259	64.296	139.1844	0	4761.512	21.5328	73.0821	30.3951	47.3891	394.6552	19.5075	1.58	14.2911	64.3444	42.8042	0	0	0	0	0	0	18.2171	279.8253	162.2646	92.2572	273.3097	24.97	846.9533	34033.13	9.5162
p1139	Admission	46.4	1.54	33.26	2.42	0	11.08	0.61	0	5.23	44	0	11.68	0	44.93	0	0	1.12	6130	14.84	1761.49	5640	330.46	323.5	485.9	1884.07	5.34	13.58	51.4	10.36	40883.42	6.35
p1139	Admission	33.48	9.01	32.16	4.77	0	26.36	1.53	0.62	6.04	35.2	0.1	12.57	11.56	20.24	0	0.26	1.41	10630	45.34	2361.8	9740	462.49	191.71	269.45	1257.14	4.66	14.01	47.1	6.91	69789.6	5.16
p1139	Admission	31.8	6.35	25.48	2.89	0	8.2	1.31	0	5.23	33.93	0	11.47	34.46	7.56	0	0	1.41	7600	90.74	2119.34	9620	428.18	197.48	282.39	1076.49	4.52	13.58	48.9	0	84065.69	5.35
p1139	D1	44.83	5.03	44.18	1.73	0	8.2	0.89	0	5.34	33.64	0	9.41	3.69	8.74	0	1.29	1.12	4580	24.26	1252.21	2590	233.13	205.37	504.52	809.14	4.79	14.22	48.9	27.67	69801.81	5.25
p1139	D1	37.07	4.49	52.8	1.03	0	0.33	0.61	1.61	5.52	44.48	0	9.08	0	233.79	0	0	0.94	390	0	370.79	550	41.36	224.67	715.19	825.91	5.6	22.27	33.8	47.57	62373.85	5.07
p1139	D2	40.78	6.35	45.27	0.12	0	0.33	1.03	0	5.11	35.39	0	6.58	0	9.9	0	0.26	1.22	540	0	324.91	630	56.81	344.43	682.97	642.89	4.38	21.87	24.1	0	95326.65	7.15
p1139	D4	34.73	0.99	49.58	5	0	5.4	0.61	0	5.46	23.27	0	4.38	0	13.38	0	0.26	2.12	1050	6.61	534.43	500	66.15	408.2	717.61	798.65	6.13	14.65	23.4	0	55135.1	5.16
p1139	D6	120.62	7.95	73.44	0.58	0	23.21	1.03	1.11	6.16	18.1	0	11.26	N/A	6.38	0	0.6	1.12	560	N/A	324.91	590	46.3	450.47	849.44	1690.77	4.52	18.62	25.6	28.2	22572.92	6.62
p1138	Admission	30.25	5.29	22.1	0	0	15.53	0.82	1.61	5.69	40.2	0	9.14	0	46.05	0	0	1.61	0	10.59	0	180	0	433.22	377.16	4213.02	14.27	27	31	131.3	17396	16.19
p1138	Admission	36.93	33.59	77.1	9.03	5.83	90.65	3.91	10.04	6.99	29.11	6.56	9.89	27.22	50.49	3.27	3.74	2.53	1260	114	981	11880	121	245.48	328.11	2407	12.69	34.44	21.3	119.3	28899.2	18.17
p1138	D1	128.3	28.2	66.61	3.83	0	23.21	1.75	4.71	5.81	29.31	4.23	15.98	0	52.71	0.24	1.64	1.51	200	39.42	455	2510	0	410.86	1543.28	4289.23	13.94	55.2	32	99.71	24842.6	21.45
p1138	D2	30.32	7.42	80.23	0	0	17.05	0.75	0	5.81	15.55	0	11.42	0	37.68	0	0	1.17	0	15.22	0	440	0	681.19	513.18	3303.6	18.11	44.91	28.1	70.16	11353.3	21.94
p1137	Admission	144.16	10.33	20.96	0.12	6.99	55.94	5.03	0	5.34	57.57	2.78	30.7	46.63	122.24	0	3.04	3.39	4260	160	1331.55	3430	243.19	309.65	1122.61	3090.66	9.15	34.26	19.5	23.46	55846.86	12.85
p1137	Admission	69.36	15.37	32.16	2.42	4.29	263.54	4.87	0	5.81	38.67	4.47	26.25	64.82	55.49	0.76	2.68	2.85	4260	196	1584.7	7040	315.36	223.82	473.81	2619.92	5.6	15.71	19.8	30.28	130500.8	10.12
p1137	D2	64.75	4.23	59.2	0	0.3	14.03	4.39	3.41	5.11	61.4	0	151.55	0.5	32.65	0	5.15	2.32	800	12.49	348.69	230	41.99	288.23	937.31	1399.95	6.13	30.85	28.1	15.38	152055.1	12.59
p1137	D5	120.26	5.82	40.93	0.12	2	42.54	4.71	0	5.69	22.66	0.7	41.29	29.74	34.89	0	5.51	2.96	1160	85.47	716.63	1100	123.58	848.47	1087.26	5533.65	8.91	36.12	75.8	56.5	50170.17	19.16
p1137	D6	105.58	8.21	32.71	0	0.86	26.36	2.51	0	5.58	35.2	0	34.54	22.19	41.03	0	4.44	3.17	1800	47.04	958.37	1640	124.44	729.2	875.54	5663.35	9.4	27	75.8	31.32	63653.89	14.44
p1136	Admission	21.64	3.25	14.7	0.8	0	0	0	3.16	0.8	16.79	2.31	4.16	9.2	40.51	0	0	1.18	1230	34.99	206	1060	92.83	130.48	281.67	620.19	2.03	30.64	33.1	0	1098.837	4.05
p1136	Admission	28.19	11.5	56.73	13.32	6.27	60.42	3.66	6.25	1.68	16.62	6.84	6.59	5.64	3.52	1.75	6.93	6.37	760	20.73	31.03	490	58.01	112.91	109.09	703.77	9.9	40.26	36.2	0	50985.17	6.32
p1136	Admission	32.75	3.03	0	0.1	0	0	0	0	0.8	15.59	0	6.11	2.5	0	0	0	0.06	360	9.33	0	50	11.87	200.7	225.86	1529.72	1.59	28.58	30.3	0	38535.71	3.84
p1136	D1	32.38	14.53	68.58	19.5	11.38	119.52	6.55	10.84	2.78	37.72	9.39	6.59	12.21	13.31	6.24	8.34	9.4	960	34.99	116	1270	94.75	157.27	164.84	874.71	13.73	44.73	36.9	0	37810.83	8.58
p1136	D2	24.55	1.86	6.73	0	0	0	0	0	0.9	12.47	1.61	3.13	3.56	0	0	0.26	0	520	13.32	0	270	50.23	141.93	141.73	431.69	1.44	20.79	37.7	0	24088.61	3.64
p1136	D3	46.37	3.93	0.73	2.91	0	0	0	0	0.85	7.05	1.35	5.43	1.36	0.42	0	0.54	1.53	310	8.56	0	60	6.71	189.62	229.42	662.08	4.21	38.41	34.6	0	25597.38	5.73
p1136	D4	47.16	9.36	36	8.55	3.11	10.91	0	2.49	1.49	7.24	5.19	4.64	9.62	1.75	0	3.85	4.5	1010	43.37	248	1020	103	218.7	233.75	950.57	8.01	34.37	38.1	0	13067.85	6.81
p1135	Admission	50.29	3.81	11.46	18.62	0	49.01	0	2.27	0.61	56.96	2.95	6.79	0	213.22	0	0.26	7.72	0	7.11	0	0	0	227.77	351.01	553.91	2.82	21.68	33.1	0	10426.3	6.37
p1135	Admission	23.91	5.33	36.85	15.08	2.86	13.96	0.96	2.71	0.9	20.83	1.85	3.98	0	24.99	0	3.99	4.84	0	0	0	0	0	177.5	101.79	325.48	6.26	18.35	35.8	0	248519.1	5.83
p1135	D1	26.19	1.76	6.73	12.08	0	0	0	0	0.48	4.47	0	4.38	0	1.75	0	3.58	4.32	0	0	0	0	0	110.31	115.17	216.07	2.3	13	34.2	0	143731.6	4.3
p1135	D1	22.63	1.97	0	14.73	0	0	0	0	0.48	1.5	0	4.05	0	8.72	0	3.02	6.71	0	0	0	0	0	137.91	129.74	183.01	1.11	9.13	28	0	85093.35	3.28
p1135	D2	30.88	2.07	0	5.38	0	0	0	0	0.48	57.78	0	13.54	0	12.44	0	6.09	1.18	0	10.2	0	0	0	122.5	357.64	93.95	1.44	28.84	28.8	0	167402.2	6.26
p1135	D3	40.39	4.74	42.63	45	1.88	4.67	0	4.49	1.05	35.21	2.53	41.99	0	2.15	0	8.9	13.55	0	9.57	0	0	0	292.07	307.31	608.18	4.65	34.37	28	0	87748.66	8.47
p1135	D4	55.15	3.47	25.44	40.39	0.48	0	0	1.82	1.2	32.52	2.08	46.71	0	3.8	0	9.18	14.87	0	2.27	0	0	0	467.65	367.57	851.28	6.26	38.65	29.5	65.54	26012.75	9.03
p1135	D5	46.05	4.74	23.58	5.02	0.05	0	0	0	0.9	8.8	1.35	6.02	0	1.88	0	3.58	2.24	0	0	0	0	0	246.23	238.32	1427.48	5.47	28.05	37.7	0	21353.53	6.75
p1135	D6	68.85	11.36	72.89	18.79	4.86	77.41	3.37	7.57	1.68	14.73	7.15	9.5	31.56	3.11	0	9.18	9.81	3790	82.78	815	4150	183	272.06	216.69	1072.31	11.36	37.01	41.1	23.09	25596.17	12.36
p1134	Admission	165.07	3.36	0	2.56	0	298.58	0	3.82	1	431.59	0	94.43	0	387.7	0	0.4	1.89	0	0	0	0	0	203.76	4133.05	2966.89	3.3	48.62	43.7	43.41	12437.58	8.13
p1134	Admission	125.87	4.04	6.73	1.86	0	182.36	0	2.04	1.1	343.2	0.79	95.03	0	22.64	0	1.64	1	0	0	0	0	0	222.47	1577.65	2128.17	3.88	48.41	41.5	60.24	85889.03	10.97
p1134	Admission	113.83	2.6	0	1.15	0	40.4	0	0	0.85	332.41	0	72.33	0	37.5	0	2.74	0.26	0	4.27	0	0	0	320.95	1338.52	2203.5	3.54	45.61	41.1	78.47	88257.34	9.82
p1134	D1	114.55	2.92	5.43	2.91	0	0	0	0	1.1	289.69	7.15	39.02	0	63.01	0	3.02	1.27	0	7.11	0	0	0	444.13	1053.52	1272.28	6.45	62.17	35.8	80	69377.25	9.37
p1134	D4	27.8	3.81	19.76	4.85	0	0	0	1.37	0.97	171.75	5.19	11.37	0	10.43	0	3.71	0.91	0	0	0	0	0	282.84	195.01	798.15	4.54	30.89	21.9	48.87	53790.97	7.3
p1134	D5	45.89	8.07	65.67	14.2	6.65	68.93	4.54	15.82	2.72	51.02	14.34	11.11	0	9.43	1.75	6.65	5.61	0	2.74	0	0	0	408.54	191.67	770.58	10.47	40.71	21.1	62.74	54418.95	11.83
p1130	Admission	62.17	7.05	36	10.14	2.37	16.97	0.65	7.13	1.41	106.6	7.46	26.69	6.31	4.77	0.64	2.19	4.67	1130	68.54	1017	8880	145	1266.84	852.24	1264.97	5.47	42.07	79.5	99.96	31285.4	17.12
p1130	Admission	47.4	13.69	82.73	20.21	11.38	122.32	6.83	16.9	3.84	92.13	16.19	28.25	7.26	14.46	5.97	8.9	9.81	500	42.36	569	5170	39.22	1006.31	423.25	1486.79	12.98	46.04	63.9	94.85	29455.2	19.32
p1130	Admission	39.2	5.82	35.15	9.61	2.86	10.91	0.49	7.35	1.74	83.76	5.53	26.78	16.79	5.47	0.64	3.58	4.67	2070	109	1222	13910	219	1505.88	433.77	1473.83	6.83	38.88	67.6	84.87	33825.5	15.86
p1130	D1	36.78	10.42	64.93	14.55	8.21	109.73	4.54	12.36	3.4	132.48	16.31	28.25	10.69	8.58	1.9	7.49	8.9	720	60.33	620	7050	66.42	1081.99	435.29	1889.65	9.14	47.34	61.5	108.93	40908.2	18.71
p1130	D4	55.82	2.81	0.73	5.73	0	0	0	4.05	1.2	32.52	1.61	47.58	0	2.97	0	1.08	2.94	0	9.93	0	330	0	2070.88	797.86	2609.46	2.03	47.77	72.8	90.32	61033.5	19.69
p1130	D5	33.66	2.7	0.73	3.26	0	0	0	1.82	1	110.58	0	70.05	0	18.97	0	1.91	2.51	0	2.1	0	0	0	2345.3	819.83	2851.61	1.74	61.59	68.3	170.71	33238.2	20.06
p1130	D6	56.49	3.47	15.74	8.2	0	0	0	8.22	1.31	157.17	3.55	86.31	0	15.91	0	3.02	3.98	0	6.73	0	0	0	2589.14	855.52	2255.01	2.69	60.81	77.7	242.54	79992.3	27.48
p1130	D7	44.03	5.94	11.46	1.86	0	0	16.83	6.69	6.1	368.11	5.53	69	0	58.88	0	3.02	3.46	0	4.3	0	0	0	2467.09	1372.5	3663.76	8.28	49.47	78.4	392.75	368481.2	26.08
p1128	Admission	51.01	10.77	119.99	25.29	5.9	66.66	17.28	33.81	0	93.29	32.91	45.02	0	5.11	6.14	22.67	5.98	140	0	0	0	0	360.74	394.42	506.45	326.21	106.12	9.7	84.71	12297.3	13.31
p1128	Admission	26.67	0	6.95	4.28	0	0	0	0	0	102.38	0	34.74	0	1.93	0	0	0	140	0	0	0	0	512.39	315.87	584.3	155.31	38.65	9.4	0	23894.95	4.43
p1128	D1	40.76	12.64	80.81	13.45	2.19	77.79	11.37	22.29	0.09	136.31	25.6	41.5	0	6.26	6.14	14.25	5.24	0	0	0	0	0	343.34	345.72	695.21	1919.15	78.71	7.6	39.84	19277.96	11.39
p1126	Admission	50.18	62.78	208.58	55.21	52.11	695.27	33.46	53.35	4.67	77.38	54.21	43.92	0	31.68	20.54	59.19	22.12	0	5.53	0	0	0	345.88	377.02	357.21	41.23	75.03	147	94.59	41356.19	27.78
p1126	Admission	55.04	36.08	109.71	31.44	19.35	320.65	23.68	41.56	0.5	62.21	59.71	57.81	0	14	14.06	39.49	11.47	0	2.29	0	0	0	392.27	416.29	820.56	26.99	54.59	38.6	71.49	75613.61	29.4
p1126	Admission	50.89	36.76	121.67	30.78	20.88	269.3	26.16	30.6	0.42	51.63	43.32	35.96	0	15.07	9.51	40.95	10.82	460	27.48	721	520	58.84	388.11	358.65	373.4	26	49.36	41.5	55.72	108844.5	24.75
p1126	D1	66.52	12.01	2.79	12.58	0.31	77.79	8.26	6.38	0	47.35	5.23	56.83	0	12.31	0	15.13	1.11	0	2.89	0	0	0	591.47	726.34	1072.88	14.27	40.38	37.3	30.59	108890.6	19.28
p1126	D2	48.99	2.85	6.95	3.41	0	0	2.04	0	0	17.81	0	7.16	0	3.15	0	4.59	0	0	1.72	0	0	0	588.33	413.92	753.85	5.05	0	35.1	0	33801.1	10.33
p1126	D3	103.23	1.4	10.47	4.5	0	0	0.53	0	0	51.02	0	23.63	0	2.63	0	0.84	0	0	7.65	36.81	0	0	871.29	638.32	890.51	8.36	12.15	21.5	0	21643.75	11.56
p1126	D4	29.8	8.32	55	11.7	7.83	33.38	4.04	1.39	0.01	9.61	15.95	6.99	0	2.94	0	11.55	1.68	0	12.06	233	70	11.21	430.86	212.36	735.93	12.67	25.88	23.9	0	33051.35	10.5
p1126	D5	36.46	2.85	30.08	6.46	3.36	16.78	1.68	0	0	11.49	14.46	4.19	0	3.47	0	8.71	0.55	0	2.29	0	0	0	551.04	295.49	819.37	9.44	18.92	26	0	28622.11	9.97
p1126	D6	29.5	11.6	78.92	13.45	10.52	116.86	9.98	16.38	0.5	15.88	26.88	7.89	0	5	0	13.36	3.74	0	5.53	0	0	0	580.61	291.63	1191.93	18.45	33.54	55.4	13.57	38276.48	14.86
p1125	Admission	51.36	12.43	41.92	7.55	0	16.78	0	3.95	1.93	29.49	19.46	13.12	0	7.09	0	3.45	0.64	0	1.5	0	0	0	191.71	289.76	953.08	7.17	43.27	23.3	28.05	59872.66	5.95
p1125	Admission	48.75	12.43	30.08	6.02	0	25.08	0	0.47	0	82.54	8.93	18.98	0	14.27	0	6.72	0	0	1.32	0	0	0	262.69	470.52	691.61	5.82	64.95	19.1	33.96	151907.3	8.17
p1125	D1	35.35	10.36	37.31	6.46	0	0	0.53	0	0.58	41.25	13.94	14.23	0	9.27	0	7.73	0.26	0	0.84	0	0	0	512.83	644.66	567.33	7.79	64.95	17	28.05	58144.41	10.24
p1125	D3	29.5	13.05	30.08	5.59	0	41.69	4.67	0	4.15	5.82	10.36	7.95	0	9.89	0	5.67	0.55	0	0.58	0	0	0	826.4	438.27	1494.95	6.52	50.8	41.8	42.78	15955.01	9.07
p1125	D4	35.21	4.75	37.31	6.46	0	0	0	0	1.46	0	2.04	5.34	0	3.79	0	0	0	0	0.99	0	0	0	578.03	279.14	561.26	0	43.27	33.8	21.27	12803.96	6.7
p1125	D5	28.57	7.31	32.54	3.41	0	0.14	0	0	0.75	0	12.26	5.85	0	4.55	0	0	0.55	0	0.84	0	0	0	564.84	330.2	527.19	5.82	53.57	30.6	20.84	8477.887	6.88
p1124	Admission	40.5	5.33	2.79	3.85	0	50	0	7.97	0	343.42	0	88.84	0	577.41	0	0.84	0	100	0	0	0	0	545.99	1287.56	5494.43	3.17	36.86	37	38.16	14671.98	12.61
p1124	Admission	79.31	5.14	27.56	6.46	3.93	712.94	9.41	3.11	0	385.46	13.94	413.05	0	2546.56	2.73	12.46	0	110	0	0	20	0	765.8	3815.24	4022.2	14.27	55.92	30.6	55.72	121328.6	16.4
p1124	Admission	41.54	6.32	22.31	2.54	0	94.51	7.1	3.11	0	536.65	11.65	535.6	0	241.55	0	14.25	0	110	0	0	0	0	957.76	1717.55	2336.83	20.89	100.28	31.9	144.57	359895.7	18.86
p1124	D1	56.84	3.98	30.08	3.85	0	0	4.67	0	0.17	471.55	10.36	280.82	0	40.37	0	22.27	0	90	0	0	0	0	816.25	2994.13	2011.72	17.14	50.44	35.4	72.31	288565.4	14.69
p1123	Admission	108.12	17.5	59.16	8.21	0.31	83.36	9.98	20.82	0	45.27	8.14	29.47	0	89.21	0	15.13	2.06	0	0	0	0	18.69	245.04	1369.72	1014.25	25.24	111.56	21.5	102.82	5456.957	15.04
p1123	Admission	71.34	9.95	57.09	11.92	2.19	86.15	10.54	7.97	0	22.91	12.84	25.4	0	270.55	0	10.15	2.81	0	1.93	0	0	0	173.43	524.87	352.23	12.03	49.36	15	37.75	65127.49	7.71
p1123	Admission	38.37	15.37	59.16	10.39	0	88.93	12.2	14.88	0	22.53	10.36	30.91	0	53.05	0	16.86	3.56	0	3.63	0	0	0	125.85	464.34	698.8	19.08	66.12	11.9	91.3	127703.7	4.91
p1123	D1	43.46	32.01	78.92	15.64	6.73	161.76	13.29	17.12	0	29.37	20.26	16.31	0	13.22	0.34	28.99	4.86	90	6.83	0	0	12.06	135.01	317.72	252.76	22.87	55.26	10.8	117.58	369048.8	8.44
p1122	Admission	165.03	20.96	65.26	39.8	3.36	83.36	3.39	12.99	0.09	74.58	25.92	18.27	0	6.61	0	4.59	14.72	230	0	342.92	130	0	273.93	365.12	1272.1	17.14	71.23	30.6	75.62	25214.74	13.66
p1122	Admission	99.66	13.68	46.39	46.84	0.31	0.14	2.04	7.18	0	42.96	14.97	21.15	0	4	0	2.23	17.22	220	0	0	0	0	165.86	190.77	850.24	12.67	68.43	19.4	46.96	55270.32	8.53
p1122	Admission	110.75	14.95	64.26	25.29	5.62	27.84	2.73	3.11	0.37	50.21	16.88	19.74	0	6.85	2.06	4.03	6.26	180	0	0	0	0	163.72	314.24	770.56	15.93	50.8	18.8	28.05	75946.04	8.71
p1122	Admission	115.34	20.09	52.89	20.24	0.96	38.92	3.39	7.18	0	39.1	11.65	17.89	N/A	9.39	0	6.2	6.36	650	N/A	2975	0	0	287.03	518.47	928.32	14.65	53.57	15.3	48.63	54513.6	10.59
p1122	D2	72.34	3.6	0	41.56	0	0	0	0	0	76.3	0	18.54	0	4	0	0	12.87	140	0	0	0	0	1368.2	574.37	1371.64	5.82	52.2	17.3	34.8	63203.13	8.71
p1122	D3	89.17	5.14	13.68	34.3	0	0	0	0	0	52.83	0	14.72	0	3.36	0	4.59	9.89	140	0	0	0	0	2738	540.59	2216.3	7.79	46.39	9.7	116.77	103115.4	15.21
p1122	D4	57.95	8.93	32.54	7.77	0	22.31	4.04	0	0.29	1110.96	8.14	41.24	0	27.84	0	14.25	2.06	250	0	538.03	0	0	2142.02	939.77	1240.76	3.17	39.95	8.9	133.55	236266	9.07
p1122	D5	58.4	4.75	36.14	13.67	0	0	0.53	0	0.46	98.87	8.93	16.25	0	4.33	0	9.2	3.93	130	0	0	0	0	1785.64	402.78	878.68	8.92	45.63	0	160.89	129702.8	13.4
p1122	D6	54.02	11.6	87.35	32.54	6.73	72.22	11.65	8.36	1	48.17	18.63	10.97	0	4.89	0	18.55	10.45	320	0	0	0	0	529.38	237.4	553.97	16.46	52.89	1.08	100.76	68269.21	10.15
p1121	Admission	51.24	5.33	28.83	9.95	1.58	97.3	3.39	0	0	106.84	0	47.69	0	10.02	0	6.72	2.43	130	0.86	161.3	0	0	464.71	1271.42	904.7	9.18	10.88	13.9	0	121831.8	5.76
p1121	Admission	45.71	9.54	39.64	15.2	0	22.31	2.04	0	0	44.54	12.84	26.42	0	42.08	0	9.68	3.93	130	0	36.62	0	0	291.74	686.88	759.82	8.92	17.23	10.2	32.28	265174.2	10.06
p1121	D1	55.72	11.6	39.64	12.36	2.78	33.38	5.29	0	0	108.89	11.65	34.05	0	20.25	0	10.62	2.62	130	0	0	0	0	533.29	1235.88	442.68	13.09	15.39	15.3	8.37	679346.4	10.5
p1121	D4	40.5	3.98	34.95	4.93	0	27.84	0.53	0	0	54.92	5.23	17.4	1.35	12.7	0	9.68	0	940	17.1	608.72	300	36.2	1200.56	1272.7	1506.45	8.36	15.39	9.7	41.1	84840.39	11.39
p1121	D5	40.1	8.32	27.56	6.9	0	16.78	0.94	0	0.25	87.79	7.27	20.12	7.6	17.08	0	18.55	0.74	1120	31.47	677.76	500	71.43	1350.25	1076.55	1225.66	6.52	13.31	5.5	40.68	108239.2	10.86
p1121	D6	37.01	8.72	19.56	4.28	0	0	2.04	0	0.17	87.01	0	18.65	0	9.52	0	15.13	0.83	120	0	0	0	0	1138.01	1247.92	1211.71	7.17	0	3.5	85.12	107543.7	10.68
p1120	Admission	15.3	10.56	46.39	6.46	0.96	114.07	4.04	0.94	0	118.81	11.02	37.81	0	37.28	0	9.68	1.3	220	18.92	99.11	0	38.03	230.19	467.43	578.24	8.36	41.64	14.4	0	58719.82	4.43
p1120	Admission	15.73	2.85	16.69	1.46	0	11.25	0	3.11	0	120.08	0	49.58	0	17.22	0	0	0.16	0	1.93	0	0	1.46	285.51	962.09	790.82	5.82	54.92	10.5	9.24	163488.8	4.81
p1120	Admission	26.83	7.11	24.97	2.98	0	0	0	1.39	0	272.14	7.27	56.15	11.37	29.9	0	5.67	0	860	39.99	420	2150	91.09	302.75	1032.59	606.09	10.44	72.06	10.8	16.14	172138.2	10.06
p1120	D1	18.18	6.32	30.08	3.41	0	0.14	0	2.26	0	634.12	8.14	103.95	0	80.51	0	16.86	0	220	18.56	36.62	280	36.33	526.19	898.32	968.4	13.88	75.57	7.3	14.86	58219.58	11.21
p1120	D4	8.35	5.53	36.14	9.52	0	44.46	5.9	1.39	1.08	125.92	22.88	21.04	0	16.68	1.3	14.25	3.56	220	16.01	0	0	25.03	673.13	361.1	649.53	11.59	40.8	2.1	39.84	17496.43	7.8
p1120	D5	3.2	0.39	10.47	3.85	0	0	0	0	0	128.11	8.93	25.14	0	14.27	0	12.46	3.56	0	1.43	0	0	0	826.26	359.97	1476.55	5.05	24.63	2.8	22.97	23585.75	8.71
p1120	D6	6.78	4.36	32.54	4.72	0	16.78	0	3.11	0.66	76.75	8.93	19.09	0	14.14	0	3.45	2.99	0	0	0	0	0	532.71	392.18	981.34	8.92	35.47	1.7	56.55	32586.35	8.35
p1119	Admission	39.17	2.85	13.68	3.85	0	240.88	0	0	0	421.08	0	41.55	0	315.01	0	3.45	0.16	0	3.16	0	0	0	944.11	1210.1	1589.09	0	32.03	21.7	0	60289.3	2.44
p1119	Admission	23.84	9.54	22.31	7.33	0	604.27	0	3.95	8.06	831.32	2.04	270.54	0	94.06	0	12.46	0	0	2.62	0	0	0	1778.77	4876.48	2441.37	55.68	1403.67	16.4	19.99	312031	49.96
p1119	D1	10.32	2.12	6.95	0.81	0	0	0	0	0	8.12	0	10.19	0	77.58	0	7.73	0	0	15.22	0	1040	0	292.92	175.88	589.15	3.17	25.88	13	0.37	433554.4	0.93
p1119	D4	16.98	0.08	6.95	0.81	0	0	0	0	0	19.82	0	5.22	3.42	14.53	0	8.71	0	170	28.56	0	2700	0	357.28	316.17	234.92	0	21.22	7.6	0	86674.1	0.69
p1119	D5	25.69	2.12	19.56	2.54	0	0	0	0	0	30.08	0	7.1	0	19.14	0	9.68	0	0	8	0	380	0	587.47	396.98	275.6	0	35.47	5.2	0	83514.4	3.65
p1119	D6	43.84	5.14	22.31	3.85	0	27.84	1.32	3.11	0	19.02	2.04	11.3	0	19.83	0	20.22	0.74	0	2.62	0	0	0	592.9	404.44	368.42	6.52	43.27	4.2	0	60066.3	4.81
p1118	Admission	42.57	17.08	65.26	12.14	6.73	122.46	9.7	13.37	0.17	61.82	22.52	29.53	0	48.87	7.88	19.39	5.05	100	0	0	0	0	274.44	409.86	969.57	14.65	59.78	26.3	30.17	27207.03	12.09
p1118	Admission	45.71	31.12	114.88	20.46	20.88	257.92	19.62	23.75	1.93	41.04	31.83	27.17	0	26.38	13.85	33.94	11.38	100	0	0	0	0	310.01	368.23	592.78	25.62	75.57	17.3	52.38	52410.03	16.83
p1118	Admission	36.46	31.34	95.57	16.96	16.28	184.29	15.7	17.87	1.21	39.31	22.15	16.96	0	13.22	9.51	30.14	8.59	90	0	0	0	0	196.06	248.38	157.44	22.31	61.33	14.1	36.48	112713.2	12.09
p1117	Admission	44.09	18.36	53.95	83.02	9.98	142.09	10.54	11.08	0	38.66	10.36	38.34	0	24.21	2.06	14.25	50.51	0	6.78	559	990	0	981.41	341.8	1217.52	17.14	83.76	31.7	94.59	37285.16	4.72
p1117	Admission	45.34	38.8	90.11	103.79	16.79	349.28	20.38	22.29	1	39.75	23.23	35.69	0	18.59	12.59	28.6	49.77	0	9.57	503	810	0	665.35	206.38	647.12	26.74	88.85	22.8	107.74	98853.06	10.15
p1117	Admission	33.94	10.77	45.29	68.89	8.37	139.28	9.13	10.3	0	29.73	8.93	32.4	0	10.39	2.73	12.46	46.64	0	4.15	178	230	0	572.3	210.07	764.59	18.45	70.68	12.6	82.23	95622.7	4.23
p1117	D1	60.37	27.1	111.44	81.69	18.33	441.41	21.15	28.09	1.8	48.79	25.6	37.6	0	12.96	16.29	29.76	47.56	0	4.15	303	450	0	507.3	295.03	528.41	26.25	96.27	10.9	107.74	64722.34	13.48
p1117	D3	23.67	2.85	10.47	38.92	2.78	0	4.67	0	0	27.95	0	26.37	0	5.8	0	3.45	24.16	0	3.45	126	260	0	762.98	274.85	564.9	7.79	51.5	13	10.11	22120.35	3.45
p1117	D4	26.18	5.92	39.64	37.6	4.5	16.78	5.29	0	0	16.72	2.04	21.26	0	4.44	0	9.68	21.48	0	5.25	279	370	0	521.55	195.11	533.28	13.49	45.63	10.2	12.71	15679.1	3.35

p1117	D5	72.74	34.94	129.2	38.92	24.91	343.55	24.43	24.47	2.18	31.94	28.41	24.17	37.13	11.15	14.06	34.31	18.24	2260	106	1924	6040	266	377.44	264.2	319.7	28.65	73.42	18.6	49.46	14418.55	12.44
p1113	Admission	25.77	5.03	20.79	19.31	0	11.66	0	0.53	5.2	30.71	0	14.36	0	9	0	0	7.48	1210	4.27	385.22	0	17.53	189.91	418.38	1142.67	4.83	29.27	22.8	11.17	111964.2	4.19
p1113	Admission	23.6	9.92	38.82	22.42	2.19	39.13	0	5.78	2.38	18.01	2.17	6.38	0	2.32	16.71	0	7.48	0	0	0	0	0	96.03	166	461.1	9.6	25.77	15.1	18.63	160692.6	5.65
p1113	Admission	15.07	6.56	100.95	16.48	0	0	0	0	2.28	13.84	0	5.18	0	1.23	0	0	3.84	0	0	0	0	0	115.58	149.69	554.62	0	13.31	13.3	11.88	250574	2.27
p1113	D5	16.98	3.33	0	9.09	0	0	0	1.2	1.53	4.51	0	3.65	0	0	0	0	0	1640	3.23	334.27	0	51.83	250.11	210.4	588.77	0	9.81	6.4	0	36295.31	2.42
p1113	D6	19.22	4.1	11.59	45.2	0	0	0	9.03	8.79	3.42	0	7.84	0	0	6.11	0	14.17	0	0	0	0	0	424.67	230.9	526.63	0	21.37	5.7	0	49817.83	3.27
p1112	Admission	96.66	6.86	47.32	8.32	2.69	52.83	0	7.73	1.59	1323.01	1.6	75.92	0.07	363	2.97	3.19	1.45	1670	59.64	652.95	1870	118.13	182.89	3502.05	1563.2	8.31	58.03	26.7	39.98	16453.41	2.56
p1111	Admission	65.03	0	0	4.27	0.82	0	0	0	0.36	12.56	0	3.43	0	55.37	0	0	0	280	9.84	450	400	27.32	226.59	368.13	1881.29	6.85	23.08	75	0	7191.034	0.49
p1111	Admission	30.23	2.4	28.21	6.46	2.94	0	0	2.19	1.95	9.75	0	2.28	28.87	1.71	0	0	2.17	1310	64.07	1373	3060	187	109.13	96.88	668.68	11.7	12.08	22.8	0	40917.88	2.85
p1111	Admission	33.18	0.14	0	2.99	0	23.16	0	0	0.47	477.94	0	57.46	6.8	251.76	0	0	0	730	41.15	1005	1120	112	154.99	2221.65	936.14	1.66	31.16	11.9	0	67539.47	3.27
p1111	D1	41.18	0	0	0	0	0	0	0	0.2	34.17	0	7.6	0	0	0	0	0	0	2.29	0	0	0	157.41	336.81	414.35	0	0	12.3	0	280627.6	0
p1111	D1	16.98	0.31	0	9.54	0.09	0	0	0	0.42	28.76	0	6.11	0	0	0	0	1.34	0	3.52	0	60	0	231.88	254.58	436.71	4.53	0	16.1	0	259358	0.17
p1111	D2	16.24	0	0	7.7	0	0	0	0	0.82	24.34	0	5.15	0	0.87	0	0	2.65	360	14.31	362	290	38.84	428.38	247.03	332.1	2.72	3.38	9.9	0	92511.64	1.62
p1111	D3	20.35	0.14	0	10.46	0.09	0	0	0	1.18	2.96	0	1.84	0	0.75	0	0	0.76	0	10.95	248	180	16.36	555.3	238.66	438.94	3.14	0	8.87	0	52002.52	2.7
p1111	D4	24.57	1.77	16.37	6.31	1.19	12.82	0	2.52	1.5	3.86	0	1.47	0	2.08	0	0	0.37	0	5.53	0	0	0	444.03	199.95	573.87	8.69	0	9.5	7.08	40652.58	3.13
p1109	Admission	48.68	0	0	0	0	0	0	0	0.47	49.35	0	1.84	23.9	300.22	0	0	0	1680	87.99	515	2040	158	321.14	321.65	1379.55	0	20.78	39.5	0	21926.6	3.13
p1109	Admission	60.95	1.29	0.4	0.65	0	0	0	0	0.59	40.22	0	10.5	2.94	3.06	0	0	0	170	22.4	132	40	4.01	550.07	468.82	1476.39	0	0	39.2	0	110151.7	2.85
p1109	Admission	15.47	0	0	0	0	0	0	0	0.31	9.2	0	1.32	17.92	4.93	0	0	0	840	55.04	124	1140	87.71	647.88	119.88	375.87	0	0	35.6	0	144124.3	0.17
p1109	D1	38.02	0	0	0	0	0	0	0	0.15	13.76	0	5.8	16.75	2.94	0	0	0	1350	83.08	439	1740	145	786.55	370.12	1642.06	0	0	27.4	6.3	124236.4	1.69
p1109	D1	16.61	0	0	0	0	0	0	0	0.15	3.42	0	0	12.43	0	0	0	0	970	43.37	99.95	1020	90.27	366.45	144.67	757.35	0	0	13.7	0	92387.83	0.95
p1109	D2	28.67	0	0	0	0	0	0	0	0.31	7.32	0	0.17	12.65	0	0	0	0	1300	68.29	384	1690	129	371.44	283.7	1506.02	0	0	16.5	19.6	37975.19	1.17
p1109	D3	26.29	0.65	0	3.31	0	0	0	1.53	0.53	153.55	0	26	2.94	121.09	0	0	0	240	9.73	0	70	5.77	715.43	515.33	1847.1	0	19.56	41	17.32	143951.5	5.1
p1109	D4	16.61	0	7.35	3.47	0	0	0	0	0.47	40.29	0	9.64	8.37	0	0	0	0	690	25.75	31.03	600	52.17	503.71	335.64	989.33	0	0	38.1	1.88	118310.9	4.26
p1109	D5	18.72	0	12.58	2.01	0	0	0	1.53	0.5	43.7	0	3.51	10.04	0	0	0	0	620	28.27	0	810	55.41	480.39	247.26	826.33	0	0	21	3.79	48772.59	2.85
p1108	Admission	45.48	5.03	24.98	3.15	14.84	225.74	9.94	7.73	1.43	50.64	0	51.04	16.17	54.27	18.58	2.45	0	870	35.69	2646	5750	105	255.89	1036.36	1970.92	23.97	49.63	18.9	0	37714.16	3.84
p1108	Admission	33.07	2.55	0	4.27	5.72	50.54	3.72	1.7	3.43	90.18	0	30.12	10.69	49.33	5.48	0	0	490	14.16	1760	2550	69.72	310.05	829.77	1281.76	16.69	57.06	15.4	0	125448.3	4.26
p1108	Admission	28.55	2.71	0	2.01	5.98	50.54	4.31	1.2	5.13	106.15	0	34.52	9.63	99.92	3.84	0	0	560	15.12	1902	2890	70.32	343.66	633.91	1211.55	14.87	41.7	19.3	0	145830.8	3.56
p1108	D1	21.87	3.79	0	6.77	8.79	52.83	6.61	1.2	1.37	54.99	0	17.42	18.79	20.74	5.8	1.61	0	670	35.69	1945	3580	82.77	389.24	344.89	747.12	17.15	28.3	12.3	10.09	196323	4.4
p1108	D2	29.75	3.49	6.19	5.06	7.89	59.7	6.61	0.53	0.82	11.79	0	9.21	20.67	14.82	8.25	2.21	2.17	540	22.08	1737	3090	63.58	514.27	376.55	832.38	16.92	20.18	10.9	0.75	67825.63	4.54
p1108	D3	30.92	3.18	4.97	3.95	6.74	45.97	5.03	0	0.88	6.55	0	4.45	21.62	28.19	5.32	0.12	0	770	34.83	2293	5120	91.91	636.47	311.04	1283.65	15.74	23.64	8.1	17.65	47466.67	3.7
p1108	D4	36.39	4.41	3.67	5.53	11.48	71.19	7.17	0	1.18	6.94	0	6.89	25.23	27.38	8.25	1.13	0	1180	38.24	3304	7900	148	798.96	348.93	2534.78	20.86	29.27	13.3	6.69	38501.43	6.62
p1108	D5	26.81	3.79	3.67	3.95	9.56	50.54	5.47	0	0.94	16.73	0	8.91	29.24	23.65	5.8	0.79	0	590	29.64	1783	2650	62.33	419.8	260.86	1100.29	17.15	15.58	5.4	25.91	120090	3.27
p1108	D6	38.53	6.1	7.35	6.77	12.51	74.65	8.56	2.85	0.7	7.8	0	9.71	29.05	19.68	8.55	2.21	4.02	640	29.64	2005	3920	68.5	716.65	398.77	936.14	22.5	29.51	11.2	36.16	50594.08	5.79
p1106	Admission	82.32	35.46	195.78	30.06	40.01	321.25	32.74	74.26	20.36	89.49	9.13	38.77	28.93	38.15	79.04	64.15	10.39	1160	30.06	706	4780	73.49	362.03	316.17	1293.1	35.82	63.38	44.9	51.73	9470.36	22.36
p1106	Admission	71.71	38.11	198.62	38.81	44.61	405.96	34.74	83.85	23.42	81.02	14.73	40.07	37.15	25.78	80.84	73.41	12.88	1700	44.33	820	5950	96.24	328.84	248.69	1111.87	36.7	70.1	46.3	47.76	18503.95	23.59
p1106	Admission	66.69	28.93	183.32	27.42	33.32	316.05	29.98	76.11	20.78	77.96	7.59	40.5	32.34	18.76	86.01	55.58	11.72	1300	31.75	780	5290	72.11	286.8	180	948	37.29	67.16	29.5	37.93	31526.33	18.11
p1106	D1	58.03	25.68	145.52	28.45	28.74	277.44	24.41	45.03	12.91	43.64	11.93	23.99	35.23	15.48	54.33	34.87	11.86	1410	36.8	807	5330	80.3	312.64	156.29	1104.15	30.38	57.71	77.5	35.57	26236.74	16.4
p1106	D1	69.81	40.94	170.15	29.77	37.51	367.21	32.24	64.24	17.44	56	8.53	32.51	40.48	21.27	66.79	51.77	16.15	2390	50.15	877	5480	122	632.25	226.65	2735.95	37.69	71.55	28.5	54.27	26073.54	22.29
p1106	D2	56.42	16.32	110.45	14.83	22.88	182.59	16.64	40.26	11.28	33.75	1.89	24.77	27.95	10.8	55.84	35.02	4.79	1020	26.69	669	4470	59.5	533.52	163.06	2513.89	27.06	54.26	26.3	13.62	17241.55	13.55
p1106	D3	56.99	27.69	173.1	31.52	35.02	326.45	27.46	65.57	18.77	47.13	7.59	34.09	36.91	16.52	80.28	59.86	9.64	1230	35.96	784	5680	73.49	621.64	139.29	900.43	33.96	61.05	48.5	34.39	13670.36	17.71
p1106	D4	78.57	19.08	149.1	24.33	30.83	310.86	23.89	87.56	20.45	44.43	3.59	37.3	27.46	12.22	59.67	44.21	4.71	860	23.32	606	4030	58.07	490.18	217.82	2340.85	28.67	69.52	21	45.47	7766.362	19.68
p1105	Admission	83.21	4.72	0.4	0.65	0	0	0	0	0.76	149.04	0	12.87	0	71.46	0	0	0	0	17.22	0	730	0	361.44	699.14	1917.21	7.07	70.1	19.6	0	9589.9	8.93
p1105	Admission	25.37	5.33	10.57	2.17	0	0	0	0	0.82	62.63	0	11.82	14.82	9.64	0	0.12	0.03	410	61.6	457	4990	19.46	288.05	245.5	624.72	0	26.8	15.4	0	196398.6	3.7
p1105	D1	59.3	7.02	3.67	1	0	2.09	0	0	0.7	158.6	0	42.86	0	19.02	0	3.69	7.17	150	27.23	0	2120	0	349.02	779.28	1557.68	0.86	44.74	38.8	30.8	252432.2	10.48
p1104	Admission	53.96	13.27	54.11	10.15	6.49	76.96	2.21	12.26	2.28	11.87	8.37	7.13	0	4.18	2.8	4.82	6.37	120	0	0	0	0	247.85	185.63	364.43	16.92	42.08	56.1	14.98	6382.842	10.01
p1104	Admission	28.31	6.41	12.58	2.5	0	0	0	2.19	0.94	0	0	1.17	0	0	0	0.45	0.76	110	0	0	0	0	143.49	106.71	438.94	0.86	16.28	21.7	0	7601.33	3.13
p1104	Admission	23.32	1.77	0	0	0	0	0	0.18	0.31	0	0	0.03	0	0	0	0	0	280	0	0	0	0	164.58	108.53	487.48	0	16.28	20	0	20173.79	2.13
p1104	D1	67.89	8.09	6.19	1.34	0	4.56	0	5.13	0.7	6.55	0	2.86	0	0	0	0.79	0.64	180	0	0	0	0	285.56	298.05	517.97	4.21	32.75	15.1	0	12942.16	5.1
p1104	D4	67.89	7.78	23.33	3.95	0.45	9.32	0	3.01	1.12	27.16	0	5.6	0	0	0	1.73	1.34	1210	7.13	633.57	560	87.04	195.99	181.61	384.98	4.53	31.16	4.4	0	15101.08	5.93
p1104	D5	63.81	6.56	18.17	2.01	0	0	0	2.52	1.25	18.41	0	7.91	0	0	0	0.56	0	1710	0	602.34	410	71.95	447.15	241.56	633.12	0	33.2	10.9	0	9976.076	6.75
p1103	Admission	36.9	6.1	10.57	4.58	11.48	204.67	8.01	7.89	1.31	230.05	0	30.37	5.21	290.92	5.16	4.82	2.56	0	7.29	0	0	0	367.92	1503.43	1673.11	21.45	64.29	18.2	0	22566.51	8.86
p1103	Admission	28.8	5.33	0.4	2.66	9.69	89.71	6.32	1.7	1	174.18	0	26.37	5.59	24.58	5.8	4.82	0	0	2.89	0	0	0	414.93	446.4	683.23	15.74	41.31	14.7	0	110966.8	6.2
p1103	Admission	25.24	2.87	0	1	8.28	81.58	5.75	0.7	0.5	140.78	0	27.12	7.63	21.27	5.64	4.57	0	260	14.31	0	180	24.29	464.38	417.93	854.48	15.98	48.39	14	0	96225.74	7.44
p1103	D1	21.12	6.86	24.98	5.21	10.33	87.38	6.75	6.27	1.88	86.55	6.26	13.84	11.58	19.42	8.25	7	3.12	460	20.34	462	660	58.84	295.48	245.63	398.57	17.15	37.28	10.6	0	131509.7	6.89
p1103	D6	26.81	6.86	28.21	5.68	11.1	78.11	5.75	3.67	1.95	16.57	0	12.41	9.35	20.21	4.83	4.44	1.55	260	12.06	174	190	21.19	516.63	216.25	465.52	17.6	36.02	6.1	0.75	20790.19	9.61
p1102	Admission	62.58	18.31	56.76	12.88	12.13	141.55	7.45	13.07	1.95	82.13	16.17	12.34	0	77.2	5.16	6.49	9.18	0	0.74	0	0	0	165.86	239.9	926.24	21.06	67.6	33.1	46.33	17262.4	17.32
p1102	Admission	45.11	17.24	64.53	13.78	10.07	110.75	4.74	9.35	2.44	111.78	6.6	17.84	29.57	15.22	8.55	8.04	8.72	1410	147	1010	13360	145	187.81	353.88	352.94	17.6	50.85	22.1	32.6	61480.7	12.69
p1099	Admission	75.31	38.43	191.97	39.1	36.33	471.42	25.43	46.18	10.66	69.23	32.07	24.49	27.52	132.64	30.49	25.7	23.64	1350	88.46	904	10320	142	264.03	258.55	732.77	37.03	109.81	47.1	82.66	20577.6	27.48
p1099	Admission	73.74	14.79	82.97	8.47	5.72	75.8	4.31	12.59	2.51	77.78	6.94	33.72	25.75	8.1	6.89	5.21	4.02	1930	98.3	1360	10720	201	260.05	381.88	1018.7	14.22	65.51	55.7	11.88	57653.4	8.73
p1099	D1	41.66	19.08	108.79	21.24	18.59	163.17	11.03	19.88	5.86	34.87	17.56	11.49	25.75	14.17	21.47	10.15	10.54	890	69.79	624	6520	49.42	158.15	137.62	380.43	23.43	62.29	7.5	39.39	69176	11.29
p1099	D2	32.18	11.44	84.16	13.18	10.07	89.71	5.75	18.26	5.28	19.91	14.73	7.6	10.07	10.93	16.17	4.7	5.55	600	34.51	543	5810	33.5	173.99	124.84	418.83	18.05	42.85	8.1	18.95	48475.9	9.47
p1099	D3	52.63	22.14	106	16.93	12.13	122.54	8.29	18.9	5.06	39.13	11.66	10.64	10.07	9.64	11.74	10.15	9.79	600	34.51	543	5810	33.5	197.89	194.09	576	20.76	53.25	7.8	27.76	48475.9	10.82
p1099	D4	35.76	13.12	90.04	16.18	11.87	96.69	8.29	15.83	5.35	27.89	12.98	9.31	8.52	11.57	16.71	7.78	7.64	600	34.18	582	6050	30.14	217.26	118.73	537.43	19.02	50.5	6.8	22.16	23788.7	10.15
p1099	D5	31.61	8.39	74.52	5.84	3.95	50.54	2.21	15.18	3.09	45.49	5.19	11.1	0	12.35	8.7	5.21	2.84	0	1.15	0	0	0	382.4	143.97	186.47	13.69	62.45	6.8	18.63	22073.4	10.15
p1099	D7	41.56	19.69	121.35	25.37	20.15	189.93	14.66	29.14	7.5	29.2	21.69	15.14	6.31	19.94	21.99	12.01	12.08	480	17.89	0	3460	0	396.77	166.54	977.55	24.77	75.81	16.1	59.31	25500.7	17.32
p1096	Admission	134.59	23.07	89.46	19.16	12.9	257.12	3.42	13.24	3.15	70.8	0	14.33	73.2	18.36	4.51	12.01	9.94	1890	125	1742	16400	269	303.48	207.47	1953.04	20.96	52.74	62.2	44.89	16899.13	15.61
p1096	Admission	99	3.18	8.46	0.29	0	172.86	0	0	0.64	107.81	0	29.28	18.9	27.38	0	0.9	0	780	50.5	676	7900	122	299.95	336.71	2051.18	2.23	53.25	43.1	25.29	150585	12.29
p1096	Admission	103.13	4.57	6.19	0.29	0	13.98	0	0.7	0.76	93.27	0	27.78	7.6	4.68	0	3.94	0	290	36.5	184	4300	60.3	321.14	315.45	2042.29	7.29	41.7	42.8	2.39	184909.5	10.95
p1096	D1	128.47	7.78	18.17	5.37	0.45	32.29	0	2.19	1.31	171.85	0	54.42	23.9	65.06	0	6.23	0.22	1140	63.6	908	8400	144	298.87	761.32	1338.3	12.58	70.68	26.7	44.03	180015.3	14.09
p1094	Admission	43.26	6.86	37.35	22.12	3.69	32.29	0	2.68	2.78	42.1	3.37	11.95	0	2.94	0.64	3.69	9.41	0	19.67	0	970	0	122.51	120.85	396.31	12.44	46.59	21.78	7.08	46234.4	6.07
p1094	D1	43.45	11.44	51.42	12.58	4.71	50.54	0.77	5.46	2.54	21.46	7.91	7.64	0	3.43	2.07	5.72	7.95	0	24	0	1850	0	115.37	97.93	186.47	13.69	51.88	7.8	8.24	118533.2	6.62
p1094	D2	42.42	2.24	0.4	14.38	0	0	0	0	1.62	11.44	0	7.5	0	0	0	0.12	4.19	0	17.86	0	0	0	130.09	114.63	301.61	0	37.7	7.5	0	61536.7	3.27
p1094	D3	28.8	2.87	11.59	41.87	0.82	0	0	1.53	3.88	7.13	0	10.04	0	0	0	1.25	17.54	0	0	0	0	0	145.21	77.18	277.81	3.52	43.99	5.1	0	36471.3	3.84
p1094	D4	33.95	2.71	0	132.69	0	0	0	0	13.12	9.56	0	16.55	0	0	0	2.21	54.01	0	0	0	0	0	213.9	91.1	1092.56	5.65	63.99	6.1	0	35035.7	4.61
p1093	Admission	27.81	24.91	94.67	19.75	15.74	170.43	9.67	14.85	3.67	22.45	11.53	23.58	0	7.72	7.95	13.35	13.02	520	0	0	0	0	141.77	102.85	355.24	22.87	58.03	26.2	30.19	65468.33	14.42
p1093	Admission	17.69	5.95	8.46	3.31	0	37.99	0	10.97	0.7	15.5	8.53	80.96	0	0.29	0	2.33	3.66	110	0	0	0	0	204.92	208.43	765.51	6.15	73.27	60.9	3.79	76009.07	13.22
p1093	Admission	20.19	4.1	0	2.01	0	6.96	0	1.53	0.42	16.65	1.6	45.46	0	0	0	0.56	0.64	130	0	0	0	0	218.6	341.46	662.42	7.5	61.98	23.6	0	59309.89	8.66
p1093	D1	30.69	25.84	93.52	20.64	15.48	160.76	7.59	13.24	3.85	19.36	11.12	19.62	0	8.1	6.43	12.01	13.17	550	0	0	0	0	262.91	173.74	414.35	21.35	63.07	20.9	36.75	36116.59	13.82
p1093	D2	18.04	4.1	4.97	2.34	0	0	0	0.86	0.94	4.93	0	4.84	0	0	0	1.25	0.03	100	0	0	0	0	566.57	224.82	828.35	2.23	36.44	18.4	0	20503.83	5.79
p1093	D3	14.66	2.24	0	0	0	0	0	0	0.42	65.82	0	22.85	0	3.43	0	1.25	0	160	0	0	0	0	370.85	382.77	991.3	0	55.42	19.3	0	153700.5	8.46
p1093	D4	12.46	5.95	3.67	2.01	0	0	0	0	1.12	29.92	0	11.79	0	0.06	0	3.44	0.37	140	0	0	0	0	460.71	196.81	658.25	0	36.23	17.2	0	87343.27	7.71
p1093	D5	60.41	3.79	0	0	0	0	0	0.53	0.64	6.35	0	4.7	0	14.17	0	0	0	130	0	0	0	0	1052.96	258.2	1110.63	6.15	41.7	30	100.97	16170.35	28.77
p1092	Admission	121.01	24.67	127.13	24.66	73.14	421.4	37.6	14.64	2.56	44.54	11.61	26.25	36.68	199.56	15.03	63.11	3.8	230	45.08	0	810	25	100.4	425.4	201.51	60	59.09	107.6	196.62	21445.83	17.6
p1092	Admission	88.66	15.41	91.94	19.84	61.27	315.57	36.84	0.59	1.72	24.76	8.31	21.18	21.11	47.96	13.64	35.79	0	1670	20.21	1333.17	4480	200.22	69.32	211.04	214.27	46.65	46.15	80.4	175.47	178645.1	14.58
p1092	Admission	100.44	23.35	139.78	9.43	77.25	451.57	56.37	32.55	0.01	31.54	3.97	38.45	6.76	41.34	13.64	65.04	0	490	0	447.54	870	37.52	102.21	260.76	337.7	50.37	64.42	71	262.36	270880.7	22.92
p1092	D1	122.04	42.31	162.03	46.53	108.65	695.7	72.07	25.33	5.38	38.69	19.91	28.18	12.7	33.92	25.94	94.81	15.35	420	0	552.2	1860	41.36	118.77	263.53	267.02	67.28	84.87	64.7	259.77	315848.7	26.73
p1091	Admission	66.05	14.17	115.33	55.36	44.12	374.21	25.04	10.12	8.62	56.01	24.86	53.63	0	40.9	15.34	22.31	21.86	0	0	0	0	0	376.62	338.77	601.43	67.46	43.06	44.1	137.84	65293.2	16.29
p1091	Admission	20.4	0	4.09	6.97	2.24	4.4	0.96	0	0	28.98	5.2	28.95	0	5.27	1.2	0.77	0	0	0	0	0	0	419.66	224.1	611.29	17.93	22.36	35.4	43.59	88117.3	4.85
p1091	Admission	28.04	0.72	28.79	13.61	8.41	43.87	5.75	0	2.28	73.61	13.4	37.6	0	10.99	3.43	4	0	0	0	0	0	0	537.59	333.33	286.98	31.37	29.47	42.4	73.83	184334.8	8.37
p1089	Admission	73.39	15.16	98.24	67.54	28.33	270.07	16.56	6.4	5.64	39.93	19.74	22.19	0	181.12	18.55	24.07	24.14	30	0	0	0	0	206.59	389.86	313.26	55.56	42.86	26.7	80.62	6273.753	15.04
p1089	Admission	47.54	0.06	0	48.57	2.82	12.41	1.19	0	0.53	57.72	0	19.79	0	9.38	7.19	5.54	13.92	40	0	0	0	0	214.09	454.17	313.26	19.96	23.35	46.6	18.58	34679.92	5.82
p1089	Admission	27.79	1.06	9.24	46.19	1.08	0	1.64	0	0.84	49.7	0	17.73	0	15.27	6.71	5.54	8.35	320	4.95	0	60	0	134.03	265.3	307.83	18.97	19.26	22.5	14.53	48953.23	4.39
p1084	Admission	87.22	1.06	49.58	10.12	43.07	254.87	23.28	0	3.65	105.86	7.34	41.29	N/A	148.45	30.41	13.26	0	350	N/A	0	0	0	354.33	710.87	990.61	69.4	41.28	99.1	82.31	11339.58	6.41
p1084	Admission	47.2	0.01	37.15	9.43	31.51	271.96	18.95	0	2.7	78.36	0	42.81	20.67	88.76	28.77	11.18	0	120	0	0	0	0	324.38	567.94	607.71	54.3	15.96	29.1	53.87	62312.81	3.61
p1084	Admission	52.87	2.64	48.83	15.34	23.81	163.39	12.75	0	3.24	25.9	8.55	22.44	10.34	19.42	20.98	10.35	0	2730	2.61	995.29	1750	152.31	118.35	220.95	82.23	49	20.83	19.3	79.78	145159.8	6.02
p1084	D6	53.68	0.06	17.76	3.08	25.94	144.25	14.16	0	3.78	13.65	0	18.05	12.85	22.78	16.72	7.51	0	2940	6.05	1009.34	1450	165.01	379.66	302.54	467.61	44.7	15.09	21.7	50.77	20476.61	4.91
p1082	Admission	3199	>10000.00	3867.27	1666.28	2762.04	10116.41	1036.69	5031.28	804.26	511.26	89.49	1624.17	50.55	188.15	1402.61	12293.73	625.3	65750	228.55	20995	166050	3960	3653.34	646.53	>50000.05	246.04	>59188.86	49.1	2076.72	31808.2	585.84
p1082	Admission	4228.3	>10000.00	3999.27	1319.31	2246.45	7569.79	799.93	2876.15	334.79	426.36	71.17	2201.2	53.25	157.53	1850.88	22472.1	432.84	36250	138.4	17340	211800	3560	6458.28	532.01	>50000.05	247.94	47315.65	44.5	1670.18	53743.41	335.03
p1082	Admission	228.3	27.471	399.27	19.31	246.45	569.79	9.93	276.15	4.79	426.36	71.17	201.2	128.95	157.53	180.88	18.9142	432.84	49600	371.7	21040	155800	3635	458.28	532.01	968.6284	247.94	71.7763	44.5	670.18	64020.01	335.03
p1082	D1	2482.05	4.71	3747.48	1836.67	16.7549	63.21	842.4	3794.08	640.72	445.69	70.5	1465.54	24.95	221.05	2020.85	8707.68	563.3	24450	109.2	18010	120950	2725	6520.78	556.62	327.34	242.52	45	30.4	1782.47	44163.36	613.52
p1082	D6	1394.62	3.2	3301.58	1090.3	1689.27	8311.53	750.46	23.61	412.7	294.97	63.21	226.47	51.45	160.89	1299.73	8.14	471.32	49700	134.75	16500	192200	3605	11313.64	481.58	781	235.46	52.67	77.8	1363.42	36282.85	369.49
p1080	Admission	68.27	2.07	33.87	16.38	6.75	55.56	4.27	0	1.06	43.67	6.83	14.22	0	50.18	8.94	5.15	0.43	0	2.29	0	0	0	287.37	379.44	425.28	31.7	43.06	21.3	49.43	0	6.8
p1080	Admission	52.23	10.08	73.65	29.12	22.48	211.11	12.75	0	3.78	80.59	12.81	20.92	0	37.84	12.4	14.95	10.51	0	2.89	0	0	0	243.46	225.84	212.45	49	46.53	20.9	63.51	17929.64	12.42
p1080	Admission	47.2	5.12	59.8	32.89	17.92	165.3	13.35	0	2.7	69.03	12.61	17.73	0	20.47	15.18	12.84	7.84	0	2.89	0	0	0	225.02	173.21	164.97	49	45	18.4	70.41	88370.76	10.33
p1080	D1	42.53	2.64	33.87	18.46	9.78	47.77	5.33	0	1.28	28.98	3.97	14.59	0	3.37	11.77	8.32	0.66	0	3.52	0	0	0	224.19	195.5	153.99	34.25	33	11.9	47.64	80838.74	6.15
p1080	D3	34.11	0.33	13.68	16.38	1.67	0.3	3.84	0	0.37	26.89	0	13.85	0	9.98	7.51	5.54	0	0	1.72	0	0	0	769.05	344.2	342.22	20.44	26.95	67.7	47.64	58957.81	6.28
p1080	D5	39.08	0.33	17.76	13.26	3.95	38.01	2.31	0	0.99	24.45	0	14.83	0	8.39	11.15	4.96	0	0	1.72	0	0	0	517.72	279.23	407.25	26	35.14	14.02	37.65	14573.11	6.02
p1080	D6	64.92	0.06	13.68	28.78	5.08	28.21	3.84	0	3.38	14.01	2.13	16.31	0	8.39	13.64	5.93	0	0	6.22	0	90	0	653.27	268.39	216.09	25.21	31.7	12.4	57.84	7144.896	5.37
p1080	D7	56.35	0.98	13.68	8.73	5.08	82.69	3.19	0	1.72	16.75	5.76	72.02	0	6.43	4.1	4.76	0	0	1.72	0	0	0	602.7	525.7	740.14	30.36	46.34	21.7	85.68	10697.52	8.63
p1078	Admission	81.35	3.04	37.15	21.22	8.41	84.62	5.96	0	0.91	17.92	5.76	18.05	0	35.87	4.1	7.11	2.1	70	1.15	0	0	0	201.25	392.84	230.66	31.7	93.06	66	25.43	9903.519	6.87
p1078	Admission	74.32	3.44	43.48	20.53	11.15	86.55	6.38	0	2	32.98	7.83	17.92	0	19.42	4.1	9.94	5.77	630	2.31	0	230	18.76	360.92	581.54	263.38	49.92	113.58	26.7	43.13	34532.82	9.94
p1078	D1	68.41	6.54	64.04	45.85	15.5	178.68	12.34	0	3.24	31.76	12.22	16.93	0	16.92	7.35	14.53	11.17	670	2.76	268.09	370	39.08	283.18	420.62	204.24	53.45	119.42	18	77.66	40922.06	10.59
p1078	D1	79.08	0	0.63	14.3	0	0	0	0	0.2	64.33	0	12.88	0	2.26	0	2.88	0	250	6.67	0	10	0	385.71	572.04	244.3	13.13	53.04	23.8	19.07	34502.37	3.54
p1078	D2	61.75	0	9.24	9.43	0	0	1.19	0	0.2	17.25	4.6	7.74	0	0	0	3.62	0	100	1.15	0	0	0	452.1	450.01	215.18	15.69	55.91	14.4	16.57	18077.46	4.65
p1078	D3	56.65	0.48	6.8	13.61	1.67	2.37	0	0	0.99	10.66	0.54	6.69	0	0.85	0	4.38	0	70	2.31	0	0	0	320.78	368.78	290.61	15.69	53.4	23.4	23.01	19127.91	4.78
p1076	Admission	94.29	15.53	127.13	47.21	33.62	323.15	20.33	8.74	7.46	41.3	22.6	21.05	16.36	37.4	13.33	25.4	30.54	1070	80.25	345.69	660	51.32	345.35	289.65	376.56	58.01	62.54	31.3	108.84	28562.16	17.34
p1076	Admission	40.06	5.55	56.93	20.53	11.42	109.68	6.8	0	2.97	21.64	7.34	11.31	49.35	12.81	6.06	11.39	10.84	730	157	150.24	480	33.41	164.91	200.31	206.98	34.87	25.77	21.7	45.84	78485.83	7.72
p1076	D1	59.54	11.02	86.82	34.26	23.01	201.58	15.76	0	5.9	34.41	15.27	17.67	0	17.34	10.83	17.95	17.86	50	0	0	0	0	160.42	226.48	133.82	50.59	39.68	10.4	62.2	151665.2	10.98
p1076	D1	27.53	0.19	4.09	5.21	2.24	0	1.64	0	0.2	20.85	0	10.76	4.71	4.31	1.02	4.38	0	250	24.7	0	20	0	309.14	248.13	180.51	1.78	14.8	8.5	17.58	182397.2	4.33
p1076	D2	37.07	0.19	5.49	4.5	0.18	0	0.73	0	0.28	8.26	0	11.06	0	9.98	0	4	0	20	0	0	0	0	394.18	268.33	978.16	5.8	15.38	45	17.07	58748.95	4.2
p1076	D5	53.36	2.45	27.04	10.12	5.63	28.21	2.97	0	1.21	2.23	1.69	10.7	0	3.37	2.42	5.15	0	40	0	0	0	0	913.9	277.16	549.4	21.38	27.18	22.1	39.49	23469.23	7.59
p1074	Admission	59.69	0.98	16.76	5.21	7.86	24.28	3.84	0	1.43	11.86	2.54	156.86	0	0	7.67	7.51	0	120	0	0	0	0	231.49	300.77	385.59	56.39	19.79	74.6	57.4	88695.99	4.72
p1074	Admission	48.57	0.64	11.52	0.92	3.95	20.33	3.41	0	0.53	21.08	0	207.27	0	1.72	6.06	5.54	0	120	0	0	0	0	291.08	512.16	445.1	31.04	18.73	82.2	63.51	96730.16	5.24
p1074	Admission	47.2	0.64	6.8	3.79	3.38	0	2.31	0	0.99	19.8	0.54	135.55	0	2.63	4.26	4	0	120	0	0	0	0	331.11	492.9	609.5	67.28	23.84	58.1	73.41	141082.3	6.8
p1074	D1	58.94	0.81	15.75	5.92	8.41	34.1	3.84	0	2	31.32	5.2	159.7	0	5.07	4.92	7.71	0	120	0	0	0	0	307.62	387.86	324.13	35.47	22.36	37.7	44.04	180908.2	6.22
p1074	D3	27.02	0.33	0	8.03	3.38	0	2.97	0	1.72	16.75	0.54	130.65	0	18.17	4.43	7.51	0	120	0	0	0	0	1073.95	360.61	1289.69	21.83	18.19	3.3	66.11	164352.6	8.63
p1074	D4	34.97	0.81	11.52	11.52	5.08	8.42	4.27	0	2.21	12.77	2.54	186.5	0	36.09	5.41	9.13	0	120	0	0	0	0	1398.06	389.81	3030.05	23.99	16.53	0.5	156.78	138375.8	9.02
p1074	D5	59.39	0.98	6.8	5.21	3.38	16.38	1.64	0	1.86	19.56	0	275.84	0	77.96	6.06	6.72	0	120	0	0	0	0	1510.34	640.63	3146.26	30.36	19.79	12	193.18	110364.1	8.5
p1074	D6	46.5	0.64	0.63	5.92	2.82	4.4	3.41	0	2.56	16.67	1.69	207.57	0	256.02	4.1	5.54	0	120	0	0	0	0	1108.9	424.08	1522.32	36.07	25.05	26.7	147.74	138658.8	6.22
p1067	Admission	63.71	5.99	30.58	2.41	0.49	4289.39	1.54	3.17	1.06	423.36	0	95.7	0	948.93	0.62	1.17	0.09	60	0	0	0	0	347.3	1860.75	2740.85	9.42	48.03	51.9	0	12138.5	8.06
p1067	Admission	41.19	2.76	45.35	2.04	0	1084.35	0.87	2.23	1.18	240.25	0	72.46	0	40.78	0	0.15	0	210	0	202.5	20	0	278.03	1494.74	1986.73	12.25	43.29	20.9	6.02	104247	9.29
p1067	Admission	43.66	2.51	35.17	0.56	0	221.95	0.54	0.32	1.01	421.05	0	99.45	0	212.08	0	0.26	0	70	0	0	0	0	265.7	2640.66	874.1	38.97	148.33	22.1	108.64	202770.1	14.17
p1065	Admission	65.91	1.88	0.63	30.84	0	793	2.53	0	0.37	323.46	8.78	131.08	0	168.1	0.66	4.76	0	20	0	0	0	0	846.54	1203.05	2891.04	53.87	31.7	20.5	91.53	46352.16	8.37
p1065	Admission	55.41	0.48	6.8	17.07	0.49	216.83	0.96	0	0	191.91	5.48	105.9	0	110.61	0	2.88	0	20	0	0	0	0	705.53	840.44	2921.65	42.94	22.86	28.5	145.77	139863.9	7.72
p1065	Admission	42.53	0.06	4.09	7.67	0.49	28.21	0	0	0	111.08	2.54	93.57	0	66.34	0	5.15	0	40	7.53	0	0	0	512.53	714.72	2839.43	41.11	22.36	29.7	226.93	208705.3	6.93
p1065	D1	61.9	0.19	6.8	10.12	1.08	0	1.19	0	0.2	36.39	3.64	43.61	0	9.78	2.08	5.15	0	20	0	0	0	0	556.29	1007.99	757.11	40.3	39.28	39.9	132.26	219563.4	5.37
p1065	D1	51.25	0.19	0	9.43	0	0	0	0	0	34.77	0	41.49	0	7.4	0	5.54	0	90	6.87	0	0	0	750.45	953.41	677.55	21.83	37.44	31.7	131.06	211134.5	5.24
p1065	D2	45.8	0.01	0.63	7.32	0	0	0.25	0	0	19.07	0	26	0	2.81	0	6.32	0	180	7.53	0	0	0	778.28	827.36	662.34	21.83	28.34	12.4	89.03	122902.3	5.17
p1065	D3	41.4	0	0	6.97	0	0	0.25	0	0	17.09	1.69	20.23	0	3.37	0.29	5.15	0	110	7.53	0	0	0	767.08	587.41	506.29	19.47	26.01	5.1	69.12	99196.27	3.54
p1065	D4	80.34	0	0	10.82	0	0	0	0	0	16.84	0	28.69	0	1.37	0.29	4.38	0	10	0	0	0	0	778.94	792.8	665.92	18.97	34.72	4	84	67061.34	6.02
p1065	D5	45.08	0.06	0	5.92	0	0	0.25	0	0	20.77	0	25.62	0	2.63	0.66	4.76	0	50	0	0	0	0	1091.81	720.4	922.98	26.77	42.27	8.5	78.51	53285.74	7.98
p1065	D6	41.02	0.48	2.54	10.82	0	0	0.25	0	0.53	15.39	0	28.44	0	1.9	0.29	4.76	0	100	7.53	0	0	0	1240.05	680.8	1053.73	27.52	34.93	4.4	71.7	42360.47	7.19
p1063	08h	77.74	3.02	106.49	1.85	0.86	30.79	0.38	0.19	1.26	21.79	0	5.52	0	6.87	1.21	2.85	0	0	0	0	0	0	188.22	166.66	1016.46	8.97	9.71	33.3	17.8	14174.9	5.08
p1063	12h	55.58	32.4	42.5	3.54	7.19	61.51	4.8	10.16	1.61	69.1	0	14.72	0	19.91	1.98	3.83	1.51	0	20.59	0	0	0	690.58	639.75	1145.93	17.91	80.85	14.4	55.16	50714.4	8.95
p1063	16h	63.98	0	9.68	0	0	0	0	0	0.77	7.93	0	3.84	0	3.13	0	0	0	0	0	0	990	0	129.88	202.01	2203.13	0	0	34.5	0	12801.4	1.29
p1063	D3	67.83	1.76	118.87	0.56	0.1	23.12	0	0	1.52	28.21	0	9.94	0	6.12	0.23	3.09	0	0	0	0	0	0	1062.69	510.52	1050.17	10.69	10.67	39.5	40.59	10367	11.92
p1063	D4	99.01	2.64	157.4	0.2	3.33	41.03	0.38	1.02	2.05	16.13	1.94	10.48	0	8.92	1.79	6.56	0	0	0	0	0	0	1754.01	552.25	1051.58	15.4	10.19	30.5	96.71	9113.3	15.02
p1062	Admission	55.98	17.68	57.65	7.72	6.36	161.53	4.45	2.85	1.96	30.86	3.05	7.6	0	26.99	3.14	2.85	1.03	0	37.14	0	2250	0	293.14	522.74	555.27	15.24	42.65	42.4	9.48	15746.6	7.26
p1062	Admission	59.31	13.03	36.66	2.41	4.35	46.15	3.84	2.23	1.18	26.12	3.73	12.86	0	30.34	1.6	1.77	0	0	37.46	0	1570	0	408.89	521.82	1590.73	24.43	175.76	22.5	49.44	44241.8	12.9
p1062	Admission	75.21	1.76	81.77	0.93	0.86	23.12	0.38	0	1.35	29.24	0	6.04	0	9.85	1.98	1.65	0	0	0	0	0	0	168.62	274.7	2490.84	10.69	12.53	22.1	6.02	7741.4	6.99
p1062	D3	51.76	11.82	80.55	2.04	2.64	35.91	1.7	11.55	1.18	54.09	0	13.4	0	11.71	0.42	3.83	2.06	0	24.94	0	0	0	531.22	420.95	1374.86	12.25	37.24	33.7	69.9	28319	8.74
p1062	D4	62.42	17.37	76.85	0	4.01	84.57	2.04	0.59	1.52	20.61	3.05	42.43	0	7.61	2.56	3.58	0	0	0	0	0	0	342.4	605.62	2038.42	14.4	56.86	17.4	117.84	19759.2	13.39
p1062	D5	72.8	20.71	77.47	0	6.03	76.88	2.89	0.73	1.87	18.59	5.32	27.67	0	10.97	1.6	4.81	0	0	0	0	0	0	270.43	756.85	1169.96	16.84	59.53	24.5	136.73	15064.7	11.01
p1062	D6	82.03	21.19	82.98	3.54	5.7	51.27	3.07	0.45	2.23	14.38	1.09	9.94	0	11.71	1.21	6.06	2.62	0	25.88	0	1380	0	376.25	661.4	1883.72	16.99	55.22	15.5	59.59	20119.8	9.84

p1061	Admission	75.29	2.51	17.33	2.04	0	74.32	0.54	2.38	1.26	90.06	0	56.77	0	101.03	0.02	4.32	0	0	0	974	780	42.92	844.9	1119.3	2450.17	9.85	6.06	17.4	22.82	34679.92	11.15
p1061	Admission	43.66	3.02	35.17	2.6	2.29	51.27	1.04	3.97	1.26	27.74	0	31.77	0	77.77	0.82	6.06	0	0	7.13	778	800	45.6	373.77	441.96	3866.32	7.54	49.1	13.2	38.87	131471.5	9.84
p1061	Admission	52.4	0.39	11.7	0	0	775	0	3.81	0.77	22.73	0	758.54	15.06	55.71	1.79	2.24	0	0	0	0	0	0	219.09	692.01	2378.04	3.2	0.52	14	60.54	149448.9	6.92
p1061	Admission	51.34	6.13	52.27	7.34	10.62	140.99	4.1	16.84	2.27	55.97	6.51	21.89	9.28	31.46	6.97	11.91	0.4	330	28.76	817	1200	59.83	161.79	400.11	3108.89	10.69	97.1	14.7	55.39	143416.4	15.31
p1061	D1	16.46	3.28	11.7	1.67	0	20.56	0.87	1.77	0.93	45.56	0	42.76	0	19.91	0.23	4.81	0.4	290	9.21	580	330	34.04	817.95	328.91	1832.41	11.87	30.38	17.8	38.65	334639.2	13.25
p1061	D2	43.9	0.6	22.52	0	0	35.91	0	0	0.93	25.07	0	28.08	0	3.88	0.23	3.58	0	0	0	291	0	0	289.85	364.58	2564.82	0	0	8.1	65.78	42126.35	7.12
p1061	D4	38.6	1.76	30.58	2.79	0.86	28.23	0.63	2.54	1.18	107.78	0	18.64	0	9.85	0.62	5.43	0.4	0	0	0	0	0	753.26	287.17	796.5	4.34	0.52	2.2	47.87	52173.8	8.06
p1061	D5	48.74	1.28	25.82	2.04	0.1	43.59	1.7	0	1.1	286.18	0	49.23	0	30.53	0.02	6.81	3.41	0	0	0	0	0	591.09	656.4	959.08	4.68	3.66	5.6	140.41	192296.2	9.97
p1057	Admission	90.1	1.76	25.01	0	0	349.87	3.07	0	1.18	273.54	1.09	81.51	0	119.09	0.62	0.94	0	220	2.66	0	60	35.05	181.94	1796.68	785.45	10.06	52.95	26.1	0	158216	13.89
p1056	Admission	57.95	10.03	52.95	4.67	2.98	48.71	6.03	7.1	1.26	23.32	0	7.6	0	13.58	2.37	1.88	0	1370	13.75	340.76	700	60.41	176.67	314.77	1086.7	7.03	40.71	21.7	11.57	8095.417	8.13
p1056	Admission	48.07	11.82	65.5	10.6	9	107.64	6.21	17.92	2.32	11.93	6.21	11.47	0	20.28	6.59	4.81	0.72	1850	31.03	605.35	1040	93.02	118.44	184.54	385.21	13.17	40.05	29.7	23.01	42665.65	13.18
p1056	Admission	53.85	5.16	29.02	0.2	0.1	455.9	4.63	7.1	0.81	13.4	0	128.31	0	19.17	0	0.94	0	190	6.87	0	500	27.87	165.51	344.12	617.92	7.28	73.41	25.3	39.3	53535.98	7.39
p1056	D1	54.06	10.33	65.5	11.56	10.95	115.33	6.03	19.36	2.41	21.55	7.08	10.4	11.99	23.64	7.54	6.06	0.56	2360	39.65	673.46	1170	109.48	123.48	243.46	475.35	12.06	37.24	23.7	30.31	78950.96	13.89
p1056	D3	66.95	7.41	45.35	0.93	0.86	28.23	4.45	3.49	1.01	30.17	0	13.25	0	16.74	0	4.07	0.88	600	12.34	121.15	590	51.65	391.14	433.54	269.15	7.54	47.09	16.7	35.89	90224.13	7.52
p1056	D4	48.07	8.27	48.84	2.41	1.23	33.35	4.63	4.13	1.26	39.16	0	13.9	16.03	18.42	0.52	3.58	0.56	1680	33.91	414.02	780	70.68	259.02	346.47	1312	8.03	41.36	12.1	24.74	63142.89	7.93
p1056	D6	89.65	5.16	46.75	0	0	18	3.07	1.32	1.52	21.2	0	11.82	0	9.48	0	3.09	0.56	160	0	0	460	21.25	348.55	421.03	503.74	8.51	38.07	10.6	53.09	29555.33	6.19
p1055	Admission	45.32	2.26	32.9	1.67	5.03	455.9	1.7	3.81	1.52	87.82	2.51	20.46	0	36.68	1.21	5.31	0.09	0	6.87	0	350	0	562.5	18.36	5325.63	18.65	35.05	32.5	0	33223.53	15.31
p1055	Admission	23.79	1.52	19.1	3.54	2.98	48.71	4.28	0	1.26	32	0	12.32	0	9.85	0.42	2.85	1.82	0	0	0	450	6.32	478.61	15.11	2593.57	15.73	20.92	30.1	0	70534.23	9.15
p1055	Admission	31.02	2.01	25.82	1.67	3.67	35.91	5.33	0	1.48	56.08	0	14.56	0	13.2	0.42	4.94	2.06	0	0	0	410	0	557.31	19.89	1945.47	19.94	18.93	22.9	0	74190.23	9.42
p1055	D1	28.85	2.01	20.83	0.93	4.01	33.35	1.04	3.17	2	457.07	0	20.62	0	21.77	0.82	5.43	0	720	11.64	0	600	31.47	322.65	16.23	557.99	9.85	25.12	12.9	0	27876.86	12.62
p1055	D2	21.85	1.28	14.58	0.2	4.01	38.47	2.21	0	2.23	353.36	0	25.37	0	21.77	0.62	10.62	0	540	0	0	590	24.95	574.23	12.13	333.04	12.99	12.08	8.8	0	20699.15	11.22
p1055	D4	14.54	2.51	46.75	3.16	3.16	38.47	3.58	1.32	1.43	62.42	0	10.63	0	9.85	1.01	4.57	1.82	500	15.89	32.01	700	36.09	424.35	13.06	329.04	15.07	18.93	6.6	6.02	42677.85	11.92
p1055	D6	25	4.62	52.27	8.1	4.69	47.43	6.12	3.17	2.14	40.46	0	10.4	0	13.2	1.79	6.56	3.25	970	9.56	152.3	680	35.74	420.13	15.95	291.74	20.22	24	10.6	6.6	10298.78	16.53
p1054	Admission	102.03	13.95	81.77	12.33	13.52	537.85	5.33	17.92	3.06	138.96	7.36	22.97	67.9	55.15	8.88	5.81	2.3	1110	204	833.22	3850	142.2	116.02	463.86	499.68	20.08	53.26	24.5	22.06	21565.64	16.74
p1054	Admission	54.57	1.52	42.5	0.2	0	225.81	0.71	0.45	1.1	113.85	0	23.09	0	42.64	0	0	0.96	120	0	0	0	0	195.35	512.33	1566.14	8.03	26.05	15.9	30.92	229410.4	8.74
p1054	Admission	51.45	2.26	49.53	0.93	0	51.27	0.54	0.73	1.14	156.61	0	21.77	10.88	22.52	0	0	0.72	560	19.92	408.96	760	32.99	172.18	543.97	1233.74	7.54	36.07	15.9	48.54	388359.6	10.11
p1053	04h	63.52	5.58	38.14	4.67	2.64	232.25	1.7	8.11	1.35	66.12	3.4	9.49	11.99	49.17	2.56	1.41	0.09	540	33.87	458	690	82.26	111.41	286.5	403.99	7.28	27.33	27.3	87.14	17613.14	10.8
p1053	08h	47.62	2.01	22.52	0	0	0.1	0.07	0	0.85	30.97	0	9.49	0	2.76	0	0	0	0	11.21	0	0	21.31	122.43	217.41	307.71	0	11.61	30.1	169.7	55031.97	4.62
p1053	16h	45.79	3.28	27.43	3.16	0.86	28.23	0.79	2.85	1.18	27.28	0	8.66	7.74	4.25	0.82	0.94	0	460	30.52	340	650	75.14	105.6	212.51	250.58	0	19.33	16.3	155.05	93057.21	8.06
p1053	D1	60.64	2.26	33.66	2.04	0	10.33	0.54	1.92	1.01	38.15	0	8.81	7.74	10.97	0.42	1.41	0	370	19.57	191	450	56.13	169.33	322.28	193.74	3.98	25.86	14	216.85	39852.18	6.79
p1053	D2	52.4	1.52	38.88	0	0	0.1	0.23	0	0.77	13.52	0	4.35	0	2.01	0	0.26	0	0	7.96	0	0	10.56	253.5	202.8	378.51	0	15.61	7.7	265.06	23395.19	5.01
p1053	D3	46.94	1.52	39.61	0.2	0	5.21	0.07	0	0.77	22.26	0	5.96	12.54	0.51	0	2.12	0	560	29.68	423	920	87.89	161.79	184.73	412.05	1.72	15.61	12.5	254.67	54312.9	6.06
p1053	D4	73.72	1.76	42.5	0.56	0	12.89	0.38	0.45	1.52	12.3	0	11.63	8.78	1.26	0	1.88	0	500	29.26	376	770	74.42	608.93	410.31	1880.79	7.54	22.47	10.2	202.9	19052.31	9.42
p1053	D5	44.85	1.52	32.13	0.93	0	2.66	0.07	0.45	1.43	2.4	0	5.63	0	0	0	0.04	0	0	4.15	0	0	6.67	444.12	283.13	928.38	4.34	16.46	14.7	245.65	18143.22	5.4
p1053	D6	100.21	3.55	54.98	2.41	0	23.12	0.71	2.54	1.78	5.29	0	11.47	0	0.88	0.62	0.94	0	0	11.21	0	0	16.06	537.02	475.98	775.78	7.28	24.94	14.7	272.92	9495.178	10.8
p1051	Admission	99.08	3.68	53.63	0.2	0	12.89	0.87	2.85	0.93	16.55	0.6	12.09	0	19.17	0	0	0	0	0	0	0	0	411.06	670.24	2595.08	11.29	44.41	27.7	21.11	8494.439	10.25
p1051	Admission	48.74	5.72	91.38	2.79	1.23	38.47	1.87	9.47	1.26	27.86	3.73	12.01	0	6.87	1.21	1.41	0.09	0	0	0	0	0	197.3	238.48	110.94	12.99	34.03	17	12.57	50754.34	9.9
p1051	Admission	35.99	1.05	25.82	2.23	0.49	28.23	2.89	2.23	0.85	7.67	0	4.42	33.66	13.2	0.42	2.12	1.59	3010	102	2039	6830	356	222.43	109.04	850.5	8.03	26.23	35	7.48	32668.9	3.67
p1051	D1	46.71	10.03	85.4	7.72	6.03	79.44	3.24	11.55	1.96	12.97	5.32	8.81	0	9.29	4.77	3.09	0.72	0	2.77	0	0	0	140.73	227.93	343.72	18.21	33.69	8.4	1.07	55233.79	11.36
p1051	D1	41.44	2.76	64.21	0.2	0	7.77	0.71	0	0.85	7.29	0	4.79	0	8.73	0	0	0	0	0.99	0	0	0	290.81	222.8	656.19	4.68	17.29	15.9	0	48672.67	4.75
p1051	D2	41.69	5.16	71.86	3.54	0.49	30.79	2.04	5.1	1.18	2.26	0	5.74	0	5.75	1.6	1.41	0.17	0	0.99	0	0	0	103.64	152.31	331.7	2.27	19.73	8.8	0.88	141963.3	5.34
p1051	D3	44.62	1.76	64.21	0	0	0.1	0.54	0.19	0.62	0	0	3.48	0	4.63	0	1.17	0	0	0	0	0	0	88.85	110.44	200.33	0	11.15	9.5	0.88	80686.4	2
p1051	D4	70.86	3.02	38.14	0.2	0	5.21	0.54	4.78	0.69	0	4.7	11.55	0	4.07	0	2.61	0	0	0	0	0	0	89.15	179.22	246.61	10.27	30.2	12.1	9.01	65087.13	6.59
p1051	D5	90.84	2.26	62.91	0	0	2.66	0.38	0	0.62	17.03	0	6.71	0	2.2	0	1.65	0	0	0	0	0	0	174.55	356.11	353.08	1.02	24.94	9.5	0	39456.73	5.01
p1050	Admission	106.85	12.12	56.32	10.02	6.69	264.45	2.21	10.51	1.87	1126.05	6.79	110.07	28.93	40.22	4.1	2.85	2.93	1860	81.5	481	3580	191	245.67	1711.62	701.43	13.88	69.62	22.5	0	71443.08	23.02
p1050	Admission	301.34	13.8	34.42	5.43	36.26	2917.08	1.04	0	0.77	5575.08	0	808.83	0	222.8	0	2.61	8.34	930	21.64	244	670	74.86	1741.58	3762.9	1203.95	18.5	127.17	27.3	0	160247.8	36.17
p1050	Admission	212.54	11.82	50.91	3.73	5.7	1963.35	0.23	0	0.77	5113.59	0	267.6	0	222.22	0	3.09	4.04	640	15.81	363	610	55.19	3082.44	3579.56	1116.29	16.84	45.2	32.5	3.46	168302.2	25.55
p1050	D1	42.56	9.15	65.5	4.67	2.64	110.2	1.54	5.43	1.48	302.51	1.94	23.77	11.09	16.93	2.37	2.73	1.75	1460	64.95	336	2500	138	642.34	528.97	261.19	11.87	35.05	17.4	21.11	169108.6	14.45
p1050	D1	45.9	8.27	56.32	0	0	58.95	0.23	0	0.69	240.05	0	23.85	9.47	22.89	0	2.85	1.67	1570	57.58	268	1980	117	1317.81	666.65	426.83	7.28	37.08	16.7	17.26	166814.2	13.6
p1050	D2	44.38	7.7	45.35	0.93	0	25.68	0.23	0	0.85	141.12	0	28.56	14.8	12.46	0	1.17	1.82	2390	71.04	410	2960	161	854.26	588.13	665.78	5.32	21.31	15.9	3.84	149366.2	7.32
p1050	D3	42.19	7.41	56.32	0	0	10.33	0.38	0	0.77	43.43	0	20.54	2.01	3.51	0	0.26	1.03	890	28.38	67.17	1070	80.3	633.11	487.55	471.3	3.98	23.62	12.5	16.72	133780.6	8.33
p1050	D4	42.69	5.99	47.45	0	0	12.89	0.23	0	0.77	22.38	0	7.53	1.38	4.25	0	0.48	1.98	560	25.01	0	900	56.63	251.33	341.92	271.81	2.76	17.29	6.3	5.18	84004.66	5.4
p1048	Admission	97.51	7.12	57.65	8.49	29.66	2195.75	2.55	4.78	5.12	383.67	17.2	326.08	17	381.11	12.31	29.68	3.25	0	2.13	0	0	0	188.68	7061.38	2869.07	50.15	99.54	52	0	15511	9.9
p1048	Admission	115.9	5.99	76.24	4.11	27.5	507.9	2.04	5.43	4.82	349.17	13.74	305.31	26.74	62.99	8.12	25.54	2.93	450	19.57	285	770	83.67	386.8	3535.74	1973.46	48.5	157.03	49.5	5.88	140574.7	11.5
p1048	Admission	129.34	2.76	53.63	0	7.85	323.8	0.71	2.85	2.23	392.08	4.06	801.06	7.74	582.76	2.56	16.84	0	0	9.57	87.69	0	17.84	606.42	4435.26	2910.43	51.28	151.87	39.9	9.01	358668.6	15.67
p1048	D1	155.76	3.15	75.62	0.2	9.65	224.52	1.54	1.92	2.87	589.28	4.7	685.05	6.73	219.73	3.14	23.42	0.56	0	0	0	0	0	1439.9	6545.88	2786.57	41.02	152.11	37.8	24.16	379351.9	17.47
p1048	D3	76.6	3.55	97.26	0.93	12.88	197.51	2.55	2.23	5.02	170.18	5.92	118.84	18.14	47.12	3.91	21.04	2.46	400	22.1	291	480	54.63	1587.64	4045.4	4667.52	30.04	74.39	39.5	43.21	91824.5	16.89
p1048	D3	54.87	3.55	85.4	2.79	10.62	268.32	3.07	2.23	4.42	116.77	5.47	79.25	20.42	80.58	3.14	19.99	4.04	300	12.03	191	200	40.08	1391.73	3479.09	4485.95	30.04	64.77	41.5	32.76	107169.9	12.48
p1048	D4	69.13	4.35	93.74	10.02	12.56	182.08	4.1	4.94	5.12	49.23	5.62	59.74	20.99	33.7	3.33	15.01	16.08	0	7.96	0	0	19.58	1747.51	2389.88	2736.28	44.94	82.89	47.8	36.74	60174.18	16.6
p1048	D5	77.58	7.41	97.26	6.58	24.55	359.99	3.24	16.84	7.77	37.64	16.86	53.56	22.71	54.21	10.4	31.7	10.89	0	9.57	0	0	21.31	1004.53	1471.2	1177.44	39.44	64.63	35.4	115.66	46718.23	16.53
p1048	D7	72.47	2.76	84.19	7.34	19.54	341.89	1.45	0.19	5.22	23.38	11.05	37.63	22.71	45.26	6.78	25.28	9.29	0	3.45	0	0	8.65	912.89	916.25	4925.34	36.47	38.07	20.5	22.06	42556.84	9.29
p1047	Admission	20.34	4.16	6.07	11.14	14.95	58.16	8.76	6.9	3.95	9.98	9.8	9.04	0	19.95	6.95	7.57	1.55	0	0	0	950	0	133.35	163.34	329.3	30.06	14.17	48.8	35.67	16249.8	5.3
p1047	Admission	12.28	1.32	0	3.81	5.81	29.05	4.51	2.94	2.35	15.57	7.71	10.46	0	12.84	4.34	6	0	0	0	0	0	0	91.9	198.44	329.3	13.24	6.87	33.8	30.08	23426.3	3.05
p1047	Admission	14.09	1.97	0	4.51	5.81	32.13	4.6	2.13	3.06	27.81	7.17	11.06	0	13.98	4.92	7.57	0	0	0	0	0	0	99.23	242.25	224.91	20.64	10.76	21.8	32.33	34872.9	3.3
p1047	D1	16.64	0.42	0	0	1.84	17.62	2.82	0.36	1.82	31.88	3.96	6.42	0	14.17	2.71	5.6	0	0	0	0	0	0	163.5	216.46	279.98	3.38	2.63	10.8	45.59	24520.6	2.11
p1047	D1	10.92	1.11	0	0	3.57	16.96	2.46	1.35	2.24	25.47	5.18	6.74	0	11.89	2.9	6.9	0	0	0	0	0	0	198.89	273.65	351.07	13.24	5.82	6.8	37.89	25785.1	2.8
p1047	D2	13.33	0	0	0	0.64	2.12	1.59	0	1.67	12.32	3.96	5.85	0	10.37	2.61	4.8	0	0	0	0	0	0	209.24	298.69	269.97	0	8.12	14.6	26.13	16959.4	2.99
p1045	Admission	56.81	2.79	0	6.78	4.14	33.35	4.42	0.14	2.43	9.98	4.27	31.08	0	10.75	5.11	4.49	0	0	0	0	0	0	240.46	283.12	938.73	18.15	16.03	15.2	55.91	32173.3	5.56
p1045	Admission	57.46	3.93	0	10.09	9.09	52.38	6.22	2.13	3.42	9.09	6.05	26.46	0	13.41	6.75	5.04	1.16	0	57.63	0	3170	0	194.07	250.09	976.3	21.42	18.48	24.9	63.97	35799.7	6.42
p1045	Admission	43.18	3.32	0	6.61	4.7	40.6	5.32	1.61	2.98	6.71	5.18	18.17	0	11.51	6.56	4.65	0	0	43.37	0	3190	0	194.87	221.7	522.83	21.42	16.44	13.4	42.85	41647.7	4.71
p1045	D1	46.8	0.16	0	0.29	0	16.31	1.59	0	1.38	15.29	0.15	28.39	0	6.63	1.28	3.58	0	0	43.37	0	3410	0	504.44	348.09	1606.38	13.24	18.88	16.7	37.61	46872.2	5.89
p1040	Admission	71.22	0.29	0	2.23	6.36	38.2	1.59	0.14	2.59	125.57	7.71	22.76	0	317.69	3.09	6.41	0	20	2.4	0	0	0	200.09	1096.39	1056.15	17.71	9.67	94.2	50.77	19480.77	3.62
p1040	Admission	57.53	0.42	0	1.35	8.01	38.2	1.59	0	2.35	131.14	7.98	22.44	0	23.85	3.38	7.57	0	30	2.94	0	0	0	335.7	905.2	860.43	13.24	5.82	94.3	55.37	85552.55	2.89
p1040	Admission	36.16	0.04	0	1.35	7.73	25.93	1.76	0.84	2.43	128.54	5.76	18.25	0	43.68	3.28	8	0	320	7.62	0	0	0	292.04	573.55	629.05	16.33	6.98	141.7	68.24	112454.1	3.17
p1040	D1	24.69	1.97	0	6.61	11.77	91.78	4.78	2.13	3.06	330.65	8.51	31.45	0	49.52	5.2	7.57	0	110	9.97	0	0	0	134.67	475.15	259.11	21.42	8.12	71.9	90.14	123953.8	3.94
p1040	D2	12.55	0	0	0	2.43	32.74	1.68	0	1.97	61.35	4.58	16.16	0	26.59	2.8	7.57	0	310	11.19	0	40	0	151.05	292.07	147.33	10.81	5.11	17.9	75.14	131012	2.73
p1040	D3	32.22	0.1	0	0	3.57	33.35	1.24	0	2.05	45.15	6.05	12.65	0	19.18	2.51	7.83	0	130	3.52	0	0	0	536.07	537.83	305.05	13.24	8.34	30.8	77.26	52086.28	3.94
p1040	D4	13.84	0.29	0	0.47	2.71	26.56	1.76	0	2.43	15.69	4.58	7.36	0	18.21	2.61	6.74	0	260	4.13	0	0	0	541.84	279.3	475.5	17.26	10.76	10.6	103.16	22074.52	3.11
p1040	D5	14.72	0.35	0	0.29	1.84	25.3	2.29	0	2.35	12.73	5.47	6.42	0	15.71	2.51	4.88	0	310	6.88	0	0	0	603.17	304.14	462.84	17.71	10	19.1	87.26	14683.48	4.2
p1040	D6	114.02	3.09	48.29	12.35	14.95	100.51	8.67	8.37	5.74	17.99	9.8	7.5	0	25.61	5.01	9.72	2.16	420	18.53	0	60	0	655.93	537.94	1031.53	29.77	34.24	23.2	154.06	16852.59	10.93
p1039	Admission	100.04	1.68	10.44	0.82	16.26	442.67	1.94	7.19	4.12	109.75	12.28	55.14	18.66	2278.52	9.86	10.08	0	0	4.88	0	420	5.8	214.78	1144.73	1460.69	37.07	34.8	45.3	37.33	12015.74	5.04
p1039	D1	13.07	0.29	5.38	0	12.3	67.29	1.76	1.09	3.71	316.72	10.81	110.22	24.45	59.04	5.98	12.68	0	0	0	0	0	0	261.42	548.26	575.42	30.06	9.23	32.3	107.57	376158	5.3
p1039	D1	16.29	0.97	0	2.76	12.3	74.05	3.17	4.47	2.9	379.23	8.25	107.26	39.9	95.87	5.98	9.03	0	480	33.07	315	5470	49.92	94.28	448.53	209.9	24.34	5.82	31.9	88.83	426131.6	3.75
p1039	D2	21.94	0.97	8.65	1.35	19.13	58.74	3.26	2.94	3.87	183.17	12.28	58.05	33.08	37.28	6.56	11.86	0	240	19.09	201	2180	34.44	176.16	612.55	304.21	36.32	8.01	33.3	93.02	280582.4	5.49
p1039	D3	15.08	0.42	0	0	10.97	48.88	1.41	0.36	3.91	65.01	9.55	25.8	34.03	33.3	5.4	11.14	0	210	14.22	114	1710	23.56	414.76	651.11	725.77	26.35	6.06	24.2	75.94	202943.6	5.1
p1039	D4	26.07	0.29	0	0	18.61	87.94	1.59	0.36	4.29	53.1	13.71	29.29	33.72	50.12	6.66	14.34	0	190	15.61	107	1550	22.5	423.7	743.65	988.55	36.57	17.57	20.5	111.18	144988.6	8.45
p1039	D5	15.81	0.29	11.59	2.76	18.09	88.49	1.76	1.35	4.41	80.97	11.67	29.67	28.93	57.01	5.78	13.05	0	0	0	0	420	3.85	320.19	637.73	994.69	35.55	9.01	15.6	139.36	182485	6.09
p1039	D6	28.84	0.9	0	3.11	23.26	105.92	2.64	2.4	4.92	45.19	14.64	33.58	34.83	49.92	7.14	14.34	0.18	0	0	0	520	5.8	253.84	617.3	543.17	39.98	11.84	15.6	152.54	118885.6	5.69
p1039	D7	37.44	0.69	0	2.76	22.23	78.53	3	1.61	4.88	29.17	12.52	30.29	30.53	44.68	6.75	13.97	0	0	0	0	420	4.31	251.56	651.55	477.19	36.06	9.89	16.3	92.75	82752.02	4.32
p1038	Admission	37.6	14.99	72.72	21.21	19.13	79.64	8.94	38.69	4.75	20.84	9.8	30	52.59	23.85	38.28	7.57	4.36	3010	133	2014	7860	320	172.86	265.45	381.26	37.32	14.17	38.9	78.32	21411.73	5.59
p1038	Admission	30.23	10.64	57.97	24.33	13.89	62.75	7.03	33.57	4.46	16.21	9.55	26.95	32.51	14.17	43.06	6	3.41	1610	66.43	1144	5140	205	104.12	125.96	327.63	35.55	9.45	25.8	67.71	32600.7	4.39
p1038	Admission	34.69	13.81	62.64	7.31	11.77	60.46	4.33	35.55	3.71	18.47	6.89	35.43	26.74	16.28	43.75	5.12	0	1990	75.52	1767	7090	271	248.7	276.26	879.53	32.07	11.2	17.9	45.59	38881.09	3.81
p1038	D1	44.14	10.31	87.99	8	3.57	357.88	1.59	36.04	2.74	17.91	5.18	223.78	0	12.27	50.14	4.49	0	460	34.28	959	1700	69.37	221.45	226.26	2138.36	31.79	13.54	35.8	56.99	38340.18	2.99
p1038	D2	35.9	10.4	37.23	4.16	5.26	38.2	2.29	22.88	3.3	21.19	5.18	30.41	5.74	14.56	46.95	4.8	0	610	24.63	559	2150	103	322.73	214.78	852.62	27.95	7.89	10.4	42.85	25441.23	3.11
p1036	Admission	36.42	2.12	10.44	9.74	12.3	54.12	5.32	2.4	3.14	23.47	9.8	12.57	40.58	51.94	6.17	6.99	0	1480	81.4	1170	4360	204	419.92	283.77	2018.41	21.42	12.48	70.5	55.37	46310.61	5.23
p1036	Admission	14.47	0.83	0	13.92	7.46	33.35	3.88	1.87	2.62	26.76	7.03	9.71	44.31	23.85	3.47	7.07	0.18	1300	93.81	1062	4050	186	339.65	155.73	1610.96	27.32	7.21	49.7	57.26	110848.7	4.39
p1036	D1	15.21	0	0	4.16	9.09	24.04	1.59	0	2.82	40.25	7.98	10.98	21.56	29.35	4.14	8.08	0	720	37.2	581	1860	109	307.83	171.29	718.04	19.01	3.65	27.6	43.67	176395.1	3.11
p1036	D1	26.46	3.02	23.29	11.83	12.84	41.79	5.95	5.74	3.95	15.21	9.8	8.49	54.86	19.37	7.43	6.74	0.78	1220	96.51	1015	4720	181	191.65	120.21	560.13	28.56	9.89	25.8	69.84	134228.8	4.2
p1036	D2	19.25	0	0	2.06	5.26	28.43	1.76	0	2.35	6.13	5.18	4.93	16.44	13.79	3.09	6.41	0	620	42.16	469	1340	97.63	174.92	167.03	321.77	11.46	0.68	9.1	30.64	43618.84	2.42
p1035	Admission	21.83	3.17	0	8.35	8.01	85.73	5.86	3.49	2.74	53.37	4.88	9.19	28.5	26	5.11	4.42	0.71	900	71.3	645	6700	96	45.33	318.77	199.06	19.84	13.12	27.4	29.52	18984.08	4.71
p1033	Admission	50.09	2.27	0	2.06	13.63	901.88	3	3.63	3.54	97.59	10.56	65.42	6.8	503.58	10.64	9.99	0	700	16.5	1110	4500	122	1114.06	1135.19	3740.98	30.06	30.95	33.1	109.38	19741.87	6.88
p1033	Admission	25.88	0.55	0	0	3	30.9	1.59	0.6	2.51	43.2	6.05	34.84	1	43.28	5.4	7.24	0	140	4.1	251	1400	26.1	631.23	493.35	1747.08	18.15	7.44	20.1	178.2	205329.5	4.2
p1032	Admission	57.96	0.04	3.13	1.18	0	831.95	2.11	3.08	1.67	164.92	2.29	54.92	0	102.32	2.51	2.7	0	510	11.64	0	230	0	261.8	2618.56	1506.97	10.81	38.97	19.5	26.7	15257.51	5.1
p1032	Admission	87.96	1.11	16.44	2.94	0	1788.89	2.11	4.18	2.28	542.85	4.58	269.37	0	71.5	2.51	4.73	0	160	10.94	0	10	0	623.99	3617.08	2411.9	27	51.62	14.4	63.97	113262.2	10.14
p1032	Admission	67.31	1.39	18.47	0	0.64	427.62	2.2	2.8	2.35	488.01	3.64	208.54	0	68.01	2.71	5.12	0	80	10.25	0	0	0	892.84	3133.77	1371.27	23.63	31.9	14.8	49.41	146754.3	10.48
p1032	D1	28.93	0.35	0	0	0	55.86	2.11	0.14	2.05	366.11	3.32	109.23	0	69.45	2.71	5.76	0	2570	32.47	925.03	2700	138.04	3017.9	1164.62	2400.33	15.35	12.05	16.9	96.41	171655.4	4.65
p1032	D1	20.24	0.55	0	1	0	50.05	1.76	9.7	1.82	204.83	2.29	92.11	0	65.56	2.51	6.08	0	700	10.94	150.24	390	27.9	3840.42	1107.08	6170.95	10.14	9.45	18.7	95.1	160044.5	6.22
p1032	D2	22.56	0.76	4.67	1.35	0	32.74	1.68	2.4	1.82	60.52	3.15	105.29	0	213.12	1.75	5.12	0	100	0	0	10	0	2897.61	2389.36	5300.82	27	27.52	14	87	119217.1	7.28
p1032	D3	21.41	0.04	0	0	0	38.2	1.76	0.6	1.75	18.82	2.64	73.6	0	23.07	1.75	4.96	0	1270	23.09	471.37	970	71.19	1279.11	1284.37	2364.7	0	5.82	15.6	47.23	155978.2	4.58
p1030	Admission	25.19	6.43	17.46	14.61	19.13	64.46	9.4	9.55	4.88	9.9	8.77	9.26	0	20.73	7.53	7.57	5.31	4570	37.82	1236.01	4520	243.69	305.45	158.62	315.08	28.87	15.41	35.6	54.29	26532.13	6.45
p1030	Admission	25.48	3.32	9.86	10.79	14.42	47.12	6.22	5.17	3.67	12.41	9.68	8.75	0	15.9	6.46	6.66	0.48	2610	8.18	774.53	2170	128.47	281.29	177.88	360.29	25.69	12.27	25.8	36.23	38199.16	5.49
p1030	Admission	17.45	0.62	0	1	0	15.65	2.11	0.36	2.09	18.23	3.15	7.72	0	8.87	2.32	4.03	0	1320	0	323.07	510	41.44	427.47	203.12	929.13	5.96	8.79	27.8	33.16	29506.3	4.32
p1030	D1	23.18	4.55	17.97	13.57	12.84	50.05	7.12	6.46	3.71	10.31	7.98	8.86	0	16.86	6.66	6	2.94	500	3.81	0	40	0	265.57	161.79	501.68	26.35	15.41	10.6	48.86	21222.4	6.65
p1030	D1	36.07	0.42	0	0	0	60.46	1.59	0	1.75	115.2	2.29	43.5	0	25.42	1.75	4.18	0	170	0	0	0	0	810.85	443.73	440.09	3.38	8.46	8.1	109.89	189450.7	4.91
p1030	D2	13.07	0.29	0	1	0	15.65	2.11	0.6	1.6	11.2	2.64	7.5	0	7	1.75	3.06	0	240	0	0	0	0	278.68	111.02	320.1	0	4.02	11.4	123.53	55762.69	2.7
p1030	D3	16.99	0	0	0	0	12.97	1.76	0	1.67	22.72	2.64	6.21	0	9.24	1.47	3.13	0	110	0	0	0	0	654.49	190.83	507.6	0	1.27	5.3	94.06	27419.61	2.8
p103	Admission	16.7704	892.9064	333.1899	128.4613	353.0149	0	104.111	93.4818	67.5462	674.3461	266.569	9.3674	14.38	158.6652	99.237	232.3841	106.9265	260	33.36	0	750	21.1	18.1141	271.672	409.5506	124.3566	2725.176	15.763	1350.669	318677.2	115.129
p103	D1	29.3232	829.8932	302.4969	148.7838	289.6676	531.4426	108.3319	116.1173	62.5078	400.6529	200.0569	24.2194	24.44	110.8497	66.7532	188.4072	242.9098	500	43.36	263	1330	48.53	27.4199	427.7923	407.9203	212.9873	2676.034	17	1222.937	199883.7	87.5496
p103	D3	31.2294	916.6299	373.4575	198.697	470.1083	3884.258	261.3385	214.7819	102.1864	322.1177	272.651	18.7825	25.02	88.7902	108.7368	266.9128	320.5738	480	42.28	263	1500	58.01	75.8203	594.8344	544.9114	346.7406	3131.078	21.6	1450.798	97795.01	298.4361
p103	D7	21.5785	553.3035	287.4875	140.2277	318.6723	0	98.0508	63.8208	50.0666	516.3669	159.2029	15.0428	2.63	61.571	75.8776	156.06	195.4199	160	7.8	0	90	16.41	154.068	461.6007	361.6297	172.9568	1554.355	9	1554.298	48713.88	135.4545
p1028	Admission	110.55	0.29	0	2.06	0	711.88	2.29	1.74	2.12	269.91	4.58	125.35	0	349.41	1.94	4.18	0	1570	11.59	709.48	1310	74.93	247.36	2478.67	2815.24	0	34.15	30.3	119.94	38305.04	5.72
p1028	Admission	33.29	0.29	0	0	0	108.08	1.41	2.8	1.46	83.21	2.29	87.92	0	30.14	1.18	3.43	0	60	0	0	0	0	258.58	866.93	837.03	0	24.82	31.6	116.59	317134.8	3.75
p1028	Admission	43.58	0.16	0	0.29	0	29.67	1.59	6.32	1.82	300.89	2.29	116.98	0	52.14	1.18	4.34	0	20	0	0	0	0	349.29	1710.03	1205.7	10.81	37.49	33.6	242.11	418094.8	6.85
p1026	Admission	84.44	2.94	0	4.51	4.7	285.44	4.51	3.77	2.51	66.36	3.96	16.32	10.47	66.79	3.47	4.42	0.18	3260	97.74	921.68	2990	160.84	207.06	325.29	1776.68	14.33	12.9	39.7	61.29	17648.23	4.55
p1022	Admission	28.84	0.1	0	2.41	0	19.57	2.64	0	1.75	10.79	4.88	11.81	0	21.7	1.94	4.18	0	480	4.84	0	450	15.95	111.47	312.89	167.36	0	16.65	11	28.96	54237.44	2.24
p1022	Admission	21.73	1.04	0	3.81	0	20.86	2.82	0.97	1.97	6.46	3.32	8.34	23.78	10	2.9	4.18	0	440	117	0	1460	39.25	49.22	189.2	73.53	5.96	13.12	12.8	23.86	168487.4	2.3
p1022	Admission	78.41	6.722	20.89	8.97	3.872	29.39	4.13	7.576	1.55	91.316	2.536	32.696	0	31.482	1.814	7.258	5.4	420	34.39	245.97	460	10.85	320.2668	535.098	709.2598	8.618	32.772	48.5014	47.836	103613.4	11.496
p1022	D1	68.26	2.42	0	4.51	0.32	29.67	3	1.35	2.31	94.14	4.88	27.11	0	23.07	2.9	6.25	0	0	0	0	250	0	180.26	741.79	223.24	10.14	35.92	24	35.67	29643.5	3.65
p1020	Admission	44.29	10.15	52.82	8.7	18.09	93.97	4.6	36.37	4.37	112.48	12.04	31.58	7.47	65.56	36.88	9.29	1.32	5510	30.73	1489.81	6850	275.59	99.93	289.76	235.75	58.42	22.66	15	51.58	21445.83	4.71
p1020	Admission	25.38	8.19	44.43	5.21	12.84	55.86	3.35	29.65	3.95	34.86	9.93	25.97	6.58	21.51	43.75	8.77	0	1350	27.19	847.12	3040	107.31	54.39	122.48	151.51	47.03	56.43	12.2	81.48	95677.16	3.46
p1020	Admission	23.08	8.11	42.07	5.56	14.95	42.39	3.53	34.89	3.71	33.01	9.8	24.34	2.46	19.18	40.47	9.81	0.04	1710	19.18	766.39	2520	77.95	36.7	112.08	112.2	40.68	11.2	9.9	110.15	119097	2.95
p1020	D1	26.27	8.35	44.43	2.76	10.17	38.2	3.17	28.19	3.3	27.3	8.77	20.51	3.91	14.75	35.69	7.24	0	2000	19.52	797.05	2420	106.37	31.91	117.79	87.86	36.06	9.23	3.2	104.46	87936.99	2.61
p1020	D1	65.4	8.19	55.04	1.35	8.55	38.8	1.68	31.28	3.38	88.64	7.17	37.54	4.27	22.68	36.29	8.77	0	1770	19.52	847.12	2630	118.75	137.28	522.91	297.53	31.79	26.94	4	75.14	37551.02	3.68
p1020	D2	39.44	5.4	42.07	1.53	8.55	36.39	1.41	24.8	3.06	110.93	6.62	30.33	0	23.46	32.71	8.08	0	1010	6.52	608.16	1600	64.97	71.57	269.08	126.44	29.47	10.98	2.7	136.81	53644.89	2.24
p1020	D4	54.92	7.39	56.51	4.69	9.09	42.98	2.82	29.32	3.26	36.87	7.98	27.28	7.11	18.02	37.68	9.46	0.04	1820	20.21	904.42	3350	134.17	145.92	261.88	151.51	31.5	9.01	3	155.07	20800.48	2.61
p102	D1	37.3393	364.7487	312.189	150.1318	246.4259	741.6573	56.4686	56.469	53.9121	3784.941	132.4785	132.3563	5.71	40.1847	64.2385	117.6526	174.1079	130	16.24	0	200	11.4	67.6972	2684.206	316.1567	147.1897	1700.299	24.2	1661.149	336253.3	70.8126
p102	D5	45.7974	336.3	312.189	134.3535	273.7995	1198.72	52.0946	57.6934	45.9737	596.9432	137.8864	15.7826	4.71	17.4242	48.9075	146.2437	178.7086	140	11.59	0	150	12.08	154.7737	3193.03	345.8875	158.7264	1618.833	23	1545.708	86512.89	82.5772
p1019	Admission	34.87	3.09	19.46	19.13	6.91	76.29	7.4	6.03	3.22	40.55	6.33	12.5	0	14.36	9.37	5.28	4.67	0	27.15	0	1850	0	11.03	279.26	93.75	30.06	9.89	35.2	54.29	56475.4	4.45
p1019	Admission	28.37	4.7	19.46	18.43	9.9	50.05	8.03	5.46	3.67	26.64	6.48	15.15	0	20.34	12.79	7.15	5.15	0	17.86	0	1070	0	16.95	316.41	72.69	31.22	10.76	30.5	53.75	86279.9	5.36
p1019	Admission	30.41	4.7	16.44	17.05	10.17	55.86	8.76	6.61	4.08	29.83	8.38	16.59	0	22.09	9.18	7.57	3.88	0	44.03	0	3460	0	33.73	425.77	85.33	30.06	13.12	26.8	59.68	80500.9	5.46
p1019	D1	20.88	3.32	14.34	19.48	4.14	44.76	6.58	8.07	3.34	13.53	7.03	11.17	0	16.09	7.33	7.24	3.81	0	27.46	0	2000	0	31.61	173.4	127.27	25.69	10.76	10.2	62.36	76733.1	4.26
p1019	D2	26.07	0.42	0	13.4	0	11.61	2.64	0.48	1.79	11.44	2.29	7.5	0	10.94	1.37	4.26	0.78	0	0	0	1160	0	0	165.29	165.7	16.33	4.27	6	37.89	23076.3	2.55
p1018	Admission	28.53	4.57	10.85	5.61	7.42	50.98	4.5	6.59	2.82	9.46	9.26	8.68	5.95	28.1	8.07	9.06	2.55	750	23.74	0	500	20.5	174.52	462.35	197.17	0	19.9	12.354	22.59	26890.32	3.52
p1018	Admission	21.69	5.49	14.25	9.82	10.97	69.39	6.16	11.16	3.23	25.17	11.5	7.17	22.59	12.33	12.05	11.07	3.61	1240	73.1	471.72	1370	62.41	236.03	306.88	216.2	0	8.58	11.283	25.18	128734.7	4.38
p1018	Admission	14.36	3.98	0	2.66	4.95	37.51	3.82	0	2.48	10.18	7.18	4.89	47.4	9.72	5.54	7.93	2.03	1140	105	453.91	2420	67.72	130.09	250.51	188.1	0	0	11.93	20.41	105653.3	1.34
p1018	D2	19.63	2.6	0	0	1	17.1	2.46	0	2.11	10.65	4.5	7.9	0	7.14	3.76	6.98	0.56	0	0	0	290	0	401.98	454.95	1146.2	0	14.66	21.093	31.96	34441.03	4.64
p1013	Admission	42.35	6.88	23.39	12.7	10.13	100.99	6.56	14.24	3.77	29.27	11.5	7.48	38.85	96.49	14.52	10.89	4.56	1050	74.34	935	4050	147	79.78	216.32	156.06	0	10.19	20.174	31.12	40695.32	3.35
p1013	Admission	20.74	2.7	0	3.49	0	7.6	1.56	0	2.82	31.02	0	15.27	0	25.7	0	8.22	2.97	0	12.03	87.69	160	31.31	92.94	238.62	187.27	0	0	14.011	33.64	87891.53	0
p1013	Admission	17.93	3.12	0	3.65	1	18.81	2.46	0	3.08	30.69	2.67	17.7	6.23	24.26	1.55	9.61	3.03	520	23.79	446	1160	72.98	74.84	240.17	166.72	0	17.08	14.817	40.45	153680	0.72
p1005	Admission	12.13	4.68	0	4.96	10.97	97.67	3.25	7.81	2.89	12.91	7.83	13.14	0	47.93	42.76	11.61	1.98	4070	0	2830	26480	359	199.67	219.47	668.63	0	5.98	24.867	21.28	23426.3	2.08
p1004	Admission	25.76	3.65	7.6	9.98	2.25	861.05	2.91	0	2.04	180.18	6.73	51.83	33.07	271.44	2.33	6.88	3.18	4510	80.3	601	3040	184	252.37	810.49	718.51	0	31.52	70.349	18.2	12398.36	1.46
p1004	Admission	19.21	2.73	0	0	0	481.03	1.67	0	1.63	77.58	0	54.1	3.23	38.11	0	6.5	0.56	660	10.92	0	370	43.37	194.87	681.68	578.57	0	5.04	12.565	17.31	122860.6	0
p1004	Admission	14.97	2.53	0	2.33	0	29.04	1.67	0	1.54	22.64	0	45.25	0	9.87	0	6.2	0.98	0	3.51	0	0	0	177.08	453.41	594.39	0	2.97	11.93	48.77	146971	0
p1004	D1	15.73	3.65	0	6.43	2.86	30.74	3.25	0	2.01	11.72	5.04	14.68	15.32	5.94	3.06	8.03	1.98	1610	34.69	185	1320	92.3	142.25	290.37	319.15	0	5.98	11.5	50.77	138331	0.21

p1004	D1	13.1	2.46	0	0	0	13.66	2.01	0	1.54	36.7	0	26.92	13.75	16.68	0	8.87	0.98	1750	34.27	235	1250	105	241.44	645.16	418.8	0	6.88	8.073	33.64	122443	0.34
p1004	D2	17.5	2.6	2.66	0.65	0	23.94	1.9	0	1.59	23.64	0	11.24	0	8.05	0	8.87	1.5	0	3.51	0	0	0	284.78	756.71	358.14	0	12.3	8.794	39.84	73137.48	1.12
p1004	D3	15.28	2.84	0	0	0.34	40.88	2.57	0	1.7	9.4	0	6.5	2.01	6.54	0	9.15	1.92	490	13.34	0	240	34.12	246.54	376.23	293.86	0	4.04	11.065	28.38	36129.53	0.72
p1004	D4	13.73	2.8	3.89	2.16	0.34	54.34	2.23	0	2.06	6.65	1.5	7.85	5.56	14.65	0	8.12	2.4	480	18.3	36.16	320	38.79	397.3	376.47	345.4	0	10.19	8.556	41.07	25267.14	2.08
p1004	D5	12.3	2.98	15.87	1.83	1.32	42.57	2.23	0	1.92	24.1	2.67	6.5	4.41	9.41	0	8.5	1.82	610	10.92	0	240	29.32	178.22	250.39	198.82	0	3.78	2.593	49.57	30945.53	1.34
p1004	D7	17.65	3.36	23.03	4.31	1.63	46.78	2.91	0	1.97	22.13	5.54	200.27	2.01	8.2	0.65	7.93	2.66	280	4.86	0	0	0	225.62	332.27	213.71	0	1.77	1.781	62.67	23841.54	0.88
p1002	Admission	30.48	3.51	0	12.7	4.95	124.15	3.19	0	2.36	19.59	8.04	12.67	0	184.26	3.92	10.34	4.03	210	0	276.51	390	7.16	161.02	257.39	1268.22	0	18.75	27.851	83.59	8213.423	3.65
p1002	Admission	15.43	3.22	0	8.53	2.86	30.74	1.9	0	2.16	25.85	4.77	15.08	0	7.14	3.41	8.5	5.51	0	0	0	310	0	243.99	325.45	562.79	0	15.71	21.821	65.02	30779.13	3.62
p1002	Admission	22.9	3.26	0	21.75	3.76	32.43	3.31	0	2.41	32.98	4.22	17.78	0	12.02	4.42	10.61	7.84	0	0	0	290	0	365.7	434.32	1047.7	0	30.23	21.457	113.82	45267.92	6.47
p1002	D1	17.93	5.18	15.07	16.21	8.42	86.88	4.84	8.97	3.61	44.61	11.14	10.72	0	27.46	7.78	10.8	6.57	1280	0	510.85	1000	60.41	160.58	239.87	252.8	0	17.08	19.06	62.28	26071.69	6.04
p1002	D1	7.95	3.01	0	7.88	2.25	27.34	1.78	0	2.87	28.96	2.31	10.38	0	10.17	2.52	9.15	3.55	250	0	0	410	0	312.03	231.58	411.08	0	18.09	24.867	63.65	20009.56	1.79
p1002	D5	20.33	2.87	0	8.53	3.76	38.35	2.46	0	2.26	5.3	5.29	8.78	0	11.4	2.52	11.52	3.82	0	0	0	310	0	316.49	191.87	357.29	0	12.85	9.494	55.16	26594.17	1.82
p1001	Admission	60.78	10.95	56.85	28.99	28.85	225.94	12.12	34.18	7.33	16.51	25.08	129.58	39.5	373.71	30.68	19.75	152.16	740	52.6	746	6300	102	124.34	358.81	199.65	0	18.26	30.277	47.16	24056.39	11.84
p1001	Admission	61.07	3.19	0	22.38	10.13	63.55	2.23	0	3.56	6.91	15	319.94	13.8	47.27	17.26	13.21	391.81	130	10.5	0	2000	31	160.58	446.66	182.33	0	9.4	15.215	19.53	41425.6	1.64
